# Ever-Adapting RND Efflux Pumps in Gram-Negative Multidrug-Resistant Pathogens: A Race against Time

**DOI:** 10.3390/antibiotics10070774

**Published:** 2021-06-25

**Authors:** Martijn Zwama, Kunihiko Nishino

**Affiliations:** 1 SANKEN (The Institute of Scientific and Industrial Research), Osaka University, Ibaraki, Osaka 567-0047, Japan; 2 Graduate School of Pharmaceutical Sciences, Osaka University, Suita, Osaka 565-0871, Japan

**Keywords:** pathogens, multidrug resistance, RND, evolution, efflux pump, adaptation

## Abstract

The rise in multidrug resistance (MDR) is one of the greatest threats to human health worldwide. MDR in bacterial pathogens is a major challenge in healthcare, as bacterial infections are becoming untreatable by commercially available antibiotics. One of the main causes of MDR is the over-expression of intrinsic and acquired multidrug efflux pumps, belonging to the resistance-nodulation-division (RND) superfamily, which can efflux a wide range of structurally different antibiotics. Besides over-expression, however, recent amino acid substitutions within the pumps themselves—causing an increased drug efflux efficiency—are causing additional worry. In this review, we take a closer look at clinically, environmentally and laboratory-evolved Gram-negative bacterial strains and their decreased drug sensitivity as a result of mutations directly in the RND-type pumps themselves (from *Escherichia coli*, *Salmonella* *enterica*, *Neisseria gonorrhoeae*, *Pseudomonas aeruginosa*, *Acinetobacter baumannii* and *Legionella pneumophila*). We also focus on the evolution of the efflux pumps by comparing hundreds of efflux pumps to determine where conservation is concentrated and where differences in amino acids can shed light on the broad and even broadening drug recognition. Knowledge of conservation, as well as of novel gain-of-function efflux pump mutations, is essential for the development of novel antibiotics and efflux pump inhibitors.

## 1. Introduction

Antimicrobial resistance (AMR) undermines our ability to treat infectious diseases, as pathogenic microorganisms become insensitive to our developed antibiotics [1]. Resistance to multiple antibiotics is called multidrug resistance (MDR) and is one of the major concerns in human health worldwide, a trend seen in clinically significant pathogenic organisms [2]. AMR can be caused by alterations in drug targets or the inactivation or alteration of antibiotics [3,4,5]. Notably, compared to these single factors contributing to the resistance of a single class of antibiotics, MDR can be caused by reduced permeability of bacterial membranes [6] and by the over-expression of multidrug efflux pumps alone, in both Gram-negative and Gram-positive bacterial cells [6,7,8]. These efflux pumps can be acquired from plasmids and horizontal gene transfer [7,9], and mutations in the regulatory network can significantly increase the expression of both acquired and intrinsic efflux pumps in clinical strains [3]. This over-expression is one of the main reasons for MDR [10]. In Gram-negative bacteria, efflux pumps belonging to the resistance-nodulation-division (RND) superfamily are one of the major contributors to MDR in clinical pathogens today [10,11]. These efflux pumps can recognize and expel many different classes of antibiotics, including macrolides, β-lactams, aminoglycosides, quinolones, dyes and detergents [12]. It is important to note that these membrane proteins have intrinsic multidrug recognition properties; however, they have been around before the clinical usage of antibiotics, and it has been shown that RND pumps play critical physiological roles in the survival and fitness of bacterial cells [13] and in cell metabolism [14], and that the multidrug recognition ability has been around since ancient transporters [15]. RND pumps form tripartite efflux systems, enabling the export of antibiotics directly to the outside of the cell [16]. The significance of the over-expression caused by increased transcription of the pump operons by mutations in their regulatory genes and proteins (e.g., the AraC family, TetR family and two-component systems (TCS)) [17,18] has been well established for most clinical pathogenic bacteria [3,17,18,19,20,21,22,23,24,25,26,27].

Alarmingly, in recent years, mutations within RND-type exporters themselves have been reported to enhance MDR by an increased efflux activity of the pump proteins. This worrying gain-of-function development adds significantly to the over-expression challenges already set by pathogenic Gram-negative bacteria, as the emergence of amino acid substitutions increases the minimum inhibitory concentrations (MICs) of antibiotics used to treat the pathogenic infections by two-fold or more. This review aims to summarize this recent development in the MDR field for a selection of pathogens: *Escherichia coli*, *Salmonella enterica* subsp. *enterica*, *Neisseria gonorrhoeae*, *Pseudomonas aeruginosa*, *Acinetobacter baumannii* and *Legionella pneumophila*. In addition, we try to summarize phylogenetic connections between efflux pumps in terms of amino acid differences (variation) and conservation within the transmembrane (motor) domain and the periplasmic (drug efflux) domain by analyzing 135 homotrimeric RND multidrug efflux pumps. These insights help us guide the development of novel antibiotics and efflux pump inhibitors.

## 2. Structure of RND-Type Multidrug Efflux Pumps

Before discussing the RND efflux pumps from different Gram-negative pathogens, we will briefly summarize our current knowledge of arguably the most studied RND pump called AcrB, from *Escherichia coli* (AcrB-Ec). More elaborate and detailed reviews regarding the structure and the mechanism of AcrB-Ec and other multidrug transporters can be read elsewhere [12,28,29,30,31,32]. In short, the first crystal structure of an RND-type multidrug efflux pump (AcrB-Ec) was solved in 2002 [33], paving the way for concise structure–function analysis after previous meticulous biochemical analysis of this efflux pump before this crystal structure was available, e.g., [34,35]. Since then, several research groups have obtained crucial information about AcrB-Ec, and other members of the RND superfamily, by solving crystal structures, analyzing biochemical data, performing molecular dynamics simulations and, more recently, obtaining electron microscope (EM) images of innate conformations of the pumps and even of the entire tripartite complexes. Examples of crystal and EM structures of RND-type multidrug efflux pumps besides AcrB-Ec are MexB from *P. aeruginosa* (MexB-Pa) [36,37,38,39], AdeB from *A. baumannii* (AdeB-Ab) [40,41] and MtrD from *N. gonorrhoeae* (MtrD-Ng) [42,43], which we discuss further in this review.

**Figure 1:**
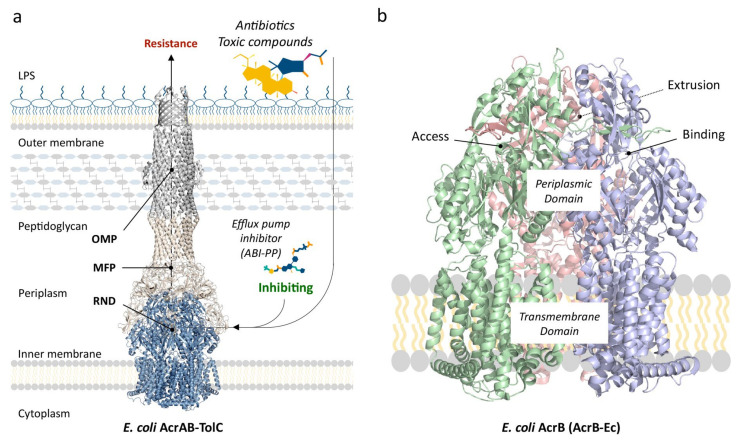
Structure of AcrAB–TolC-Ec and AcrB-Ec. (**a**) The structure of the tripartite complex AcrAB–TolC from *Escherichia coli* (PDB accession code 5O66 [48]). Antibiotics and other toxic compounds enter through the outer membrane and are captured by the RND efflux pump and consequently pumped out of the cells. ABI-PP is an efflux pump inhibitor (EPI), stopping the pump from functioning. (**b**) Structure of AcrB-Ec. Green shows the access monomer, blue the binding monomer and red the extrusion monomer (PDB accession code 3AOD [51]). Abbreviations: OMP, outer membrane protein; MFP, membrane fusion protein; RND, resistance-nodulation-division protein.

To summarize, RND multidrug efflux pumps are homotrimeric proteins embedded in the inner membrane of Gram-negative bacterial cells and couple with six membrane fusion proteins (MFP). (Among RND multidrug efflux pumps, there are also heteromultimeric pumps [30]; however, this review focuses on the homotrimeric group of pumps). There have been debates on whether the RND pump itself directly, or indirectly through MFPs, couples to the outer membrane protein (OMP) tunnel [44,45], which lies embedded in the outer membrane, and how many proteins of each three of the segments (RND, MFP and OMP) comprise the tripartite complex [46,47] (Figure 1a). However, there has been a growing consensus that one RND efflux pump trimer couples with six MFPs, and that this hexameric MFP tunnel interacts and forms a complex with three OMP monomers by relatively weak tip-to-tip interactions. This consensus is guided mainly by the elucidation of the structures of the entire tripartite complexes of AcrAB–TolC (*E. coli*) and MexAB–OprM (*P. aeruginosa*), obtained by EM imaging [16,38,48,49,50].

**Figure 2:**
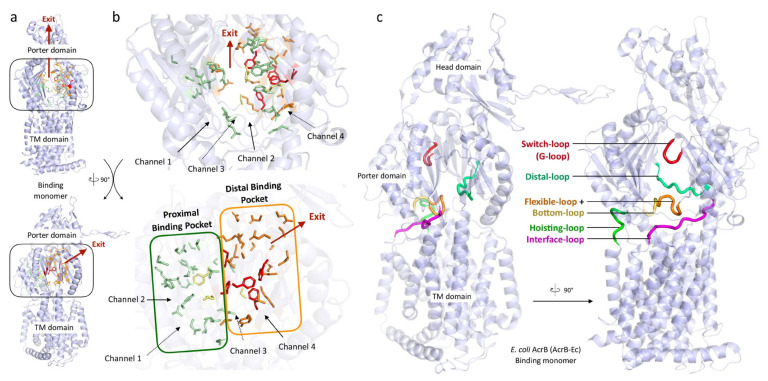
The structure, domains and loops of the RND monomers. (**a**) Side views of the entire protomer of AcrB-Ec. (**b**) The porter domain with the highlighted proximal (PBP) and distal binding pockets (DBP) and their drug-interacting residues. Arrows roughly indicate channels (dashed arrows indicate behind the image). Colors: orange, DBP; green, PBP; yellow, between DBP and PBP; red, Phe residues in the hydrophobic pit. (**c**) Side views of the flexible loops. The cartoon representation is transparent, allowing one to view all loops and residues in their entirety. PDB accession code 3W9H [36].

The RND efflux pump itself (Figure 1b) consists of three monomers forming a homo-trimer, each showing one of three distinct conformations called access, binding and extrusion [52] (or alternatively loose (L), tight (T) and open (O) [53]), when actively pumping substrates. The trimer oscillates between these three states, from access to binding to extrusion and back to access, and this movement is called the “functionally rotating mechanism” [52]. Throughout this cycle, drugs move through one of the protomers of the pump by a peristaltic motion in the porter domain (Figure 2) [53]. There are two distinct drug-binding pockets within each monomer: a deep distal binding pocket (DBP) and a proximal binding pocket (PBP) [51,54] (Figure 2a,b) separated by the switch-loop [54] (sometimes referred to as the G-loop in the literature [30,55,56]) (Figure 2c). The flexible hoisting-loop enables the significant conformational changes in the porter domain [57]. As shown in Figure 2c, there are several other distinct functional loops within the monomers. Crystal structures of efflux pump inhibitor (EPI) ABI-PP bound to AcrB-Ec and MexB-Pa show the existence of a hydrophobic pit or trap (or inhibitor binding pit) [36], rich in phenylalanine residues. Other EPIs (MBX inhibitors) bound to AcrB-Ec (porter domain only) have also been crystallized [58]. It is hypothesized that large drugs, such as erythromycin and rifampicin, bind strongly to the PBP in the access monomer, and smaller drugs, such as minocycline and doxorubicin, bind strongly to the DBP in the binding monomer [51]. However, the large surfactant molecule LMNG (lauryl maltose neopentyl glycol) was recently found to be bound to the DBP of MexB-Pa in the binding monomer [37]. This was also the case for erythromycin, bound in the hydrophobic pit of MtrD-Ng [43], overlapping the ABI-PP binding location in AcrB-Ec and MexB-Pa [36]. Additionally, smaller molecules such as doxorubicin and ethidium have been found to be present in the PBP of AcrB-Ec [54] and AdeB-Ab [41], respectively, besides being found in the DBP. Molecular dynamics simulations have shown that, depending on the molecular properties, pump substrates move within the pockets and have preferred binding sites [55].

## 3. Conservation among RND Efflux Pumps Highlights Important Domains

In this section, we provide an overview of conservation distilled from the comparison of numerous RND multidrug efflux pumps. We previously analyzed about 400 RND genes from Gram-negative gammaproteobacteria [15] (including heavy metal efflux pumps (HME) [59,60], heteromultimeric MdtBC-like pumps [59,61,62] and others such as TriC-like efflux pumps [63,64]). For this review, we specifically selected from that database 133 homotrimeric multidrug efflux pump genes and added the sequences of MtrD-Ng and AdeG-Ab, converted the DNA sequences to amino acid sequences and performed protein multiple sequence alignment [65] on a total of 135 pumps. We also zoomed into 19 better studied and defined efflux pumps from *E. coli* (AcrB-Ec, AcrD-Ec and AcrF-Ec), *Salmonella enterica* (AcrB-Sa, AcrD-Sa and AcrF-Sa), *N. gonorrhoeae* (MtrD-Ng), *P. aeruginosa* (MexB-Pa, MexD-Pa, MexF-Pa, MexI-Pa, MexQ-Pa, MexW-Pa and MexY-Pa), *A. baumannii* (AdeB-Ab, AdeG-Ab and AdeJ-Ab), *L. pneumophila* (LpeB-Lp) and *H. influenzae* (AcrB-Hi). The sequences of all 135 pumps are provided in Supplementary Data S1.

### 3.1. Conservation Heat Maps Show Distinct Areas of Importance and Adaptation Flexibility

**Figure 3:**
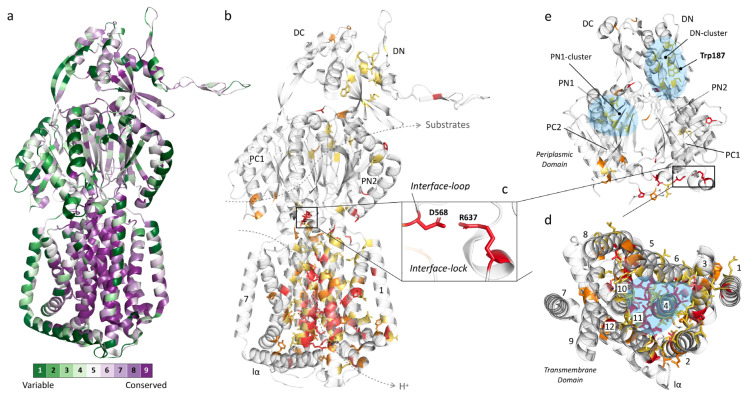
Heat maps of conservation, based on 135 sequences of RND multidrug efflux pumps. (**a**) Side view of AcrB-Ec showing conservation of the monomer, analyzed by using ConSurf [67,68]. Conservation is relative (most conserved “category 9” (dark purple) ranges from 50% to 100% identically conserved depending on the residue; Table 3 and Table S1). (**b**) Manual conservation heat map based on Clustal Omega [65]. Residues can be found in Table 2. (**c**) The “interface-lock” between D568 from the “interface-loop” and R637 from the PC1 subdomain. (**d**) Top-down view of the transmembrane domain. (**e**) Outside-in view of the periplasmic domain. Dashed lines indicate located at the back (for PN1 and PN2). Coordinates from high-resolution AcrB-Ec (using DARPin inhibitors, PDB accession code 4DX5 [54]). Colors: (**a**) Green, variable regions; purple, conserved regions. (**b**–**e**) Red, fully conserved among all 135 pumps; light red, conserved among the 19 selected pumps while also highly conserved in all 135 pumps; orange, fully conserved in 19 pumps; yellow, highly conserved in all 135 pumps; blue, conserved hydrophobic clusters.

We created two heat maps derived from 135 efflux pumps, each counting, on average, 1043 amino acids which make up one monomer of the trimeric RND pump. The first map is automatically created, based on HMMER homology [66], by using ConSurf [67,68] after first performing multiple sequence alignment by Clustal Omega [65] (Figure 3a). The second map was manually created after using the same Clustal Omega output, based on the following criteria (Figure 3b–e): (A) fully (identically) conserved among all 135 pumps (32 residues, red), (B) conserved among the selected 19 pumps while also highly conserved among all 135 pumps (13 residues, light red), (C) fully conserved among the selected 19 pumps (33 residues, orange) and (D) highly conserved among all 135 pumps (58 residues, yellow). This second map focuses solely on the most conserved residues because the highest conserved residues found by ConSurf are relative and include residues that are between 50% and 100% identically conserved. The residue conservation analysis (including percentages and variability per residue) can be found in Supplementary Table S1. Note that there are a total of 71 highly conserved residues (58 yellow and 13 light red residues) among the total 135 pumps. Additionally, note that there are 78 fully conserved residues among the selected 19 pumps, where these include not only the 33 depicted in orange but those in the red and light red color categories, too, by definition. We included this light red category partly because this includes residues that we know to be crucial for the function of the pump (e.g., AcrB-Ec’s D408 and K940, part of the proton relay pathway). These residues are different in only a few (two and three, respectively) of the total 135 pumps (the “K940R” mutation in MexB-Pa resulted in a fully active pump [34]). Nonetheless, we can clearly see distinct areas of conservation and areas where there is basically no conservation (high variability). Conservation suggests specific residues to play an essential role in the functioning of the pumps, may it be for proton relay, remote conformational coupling, stability or flexibility of the pump, stability of the trimeric complex, etc.

Figure 3a,b show the conservation heat maps for homotrimeric RND multidrug efflux pumps, based on the 135 pump sequences. Table 1 lists the conservation in specific subdomains (based on the AcrB-Ec sequence), Table 2 lists the conserved residues (also based on AcrB-Ec, Figure 3b), and Table 3 lists other highly conserved residues found by ConSurf (Figure 3a). More heat map images can be seen in Supplementary Figures S1 and S2 and ConSurf heat map images in Figure S3. From these overviews, it is visible that the primary conservation is found in the transmembrane domain (TM domain), mainly in two TM helices: TM4 (with the D407 and D408 residues) and TM11 (which contains R971), with complete or high conservation of 63.6% and 39.4%, respectively (Table 1). These residues in these helices (D407, D408 and R971) play crucial roles in the proton transfer and, therefore, energy consumption by the pumps [33,69,70,71].

In TM4 (Table 2, green background), which is composed of 33 amino acids, six residues are completely conserved (using AcrB-Ec numbering: N391, L400, V406, D407, I410 and E414), three are conserved in the selected 19 pumps and highly conserved in all 135 (D408, A409 and V412), four are additionally conserved in the selected pumps (I402, G403, N415 and R418) and eight are highly conserved among all pumps. As for TM11 (Table 2, blue background), five residues are fully conserved (R971, R973, M977, T978 and P988), two are conserved in the selected pumps and highly conserved in all (A963 and A981), two additional residues are conserved in the selected pumps (P974 and G985) and four are highly conserved in all pumps. As seen in Table 2 and as mentioned before, D408 (TM4) and K940 (TM10)—which form salt bridges with D407, and provide the energy transduction in the pumps [12]—are not conserved in sequences from two to three organisms (Table 2 and Table S1). However, we know from experimental data that these residues are crucial to the function of the pump (shown with asterisks (*) in Table 2, more at Discussion (Section 6)). TM7 and lα (both almost entirely green in Figure 3a) are significantly variable (merely 3.7% and 0% conserved, respectively, Table 1). A multitude of other conserved residues in the TM domain form hydrophobic patches and clusters where these residues come together, especially between TM4, TM5 and TM6 (highlighted in blue in Figure 3d and Figure S1). TM numbering and locations can be seen in Figure 3d and Figure S1.

### 3.2. Conservation in the Periplasmic Domain

From Figure 3 and Table 1, Table 2 and Table 3, we can conclude that the TM domain is significantly conserved, while the periplasmic domain (with the porter and head subdomains) is significantly variable (Figure 3e, Figures S2 and S3). As specific included transporters (such as AcrD) have a significantly different drug recognition spectrum, this was partly expected. However, efflux pumps with similar drug spectra also do not show stringent conservation, which we will discuss further in the next section (Section 4). Conserved or highly conserved residues which do exist in the periplasmic domain are not located in the binding pockets but, alternatively, probably provide flexibility to the loops (e.g., P36, P119, G171, P318, P565 (interface-loop), G619 (switch-loop)) and structure (Figure 3e and Table 1; Table 2). Interestingly, it is in the porter domain where the only two interacting fully conserved charged residues in the entire protomer of all 135 pumps are located: D568 and R637 (on the PC1 subdomain), shown in Figure 3b,c,e. D568 is located on a semi-conserved loop (Table 1) we here named the “interface-loop”, as it lies in between the TM domain and the porter domain, and we therefore assigned the name “interface-lock” to the residues’ interaction. This loop is also the most conserved among all loops (Table 1 and Table S1 and Figure 3). Future experimental data are needed to explain the function of these conserved interacting residues. Another fully conserved residue is N298, for which it has been shown that mutagenesis to a bulky tryptophan (N298W) inhibits the AcrB-Ec efflux ability significantly for all tested compounds [72]. N298 has also been found to be implicated in the binding of carboxylated β-lactams, fusidic acid and DDM in recent crystal structures and biochemical analysis of AcrB-Ec [73]. This same study found that the N298A mutation significantly negatively affected the carboxylated β-lactam MICs (also seen in binding differences in drug-bound crystal structures), but not the erythromycin MICs. On the other hand, our recent mutation N298W significantly negatively affected the MICs of all tested compounds [72]. We discuss the carboxylated β-lactams’ conserved drug recognition further in Section 4.6. The three residues (N298, D568 and R637) are even conserved in phylogenetically distanced [15] TriC (data not shown). Two other highly conserved hydrophobic cores (named here the “DN-cluster” and “PN1-cluster” in Figure 3e, highlighted in blue) are present in the porter domain, consisting of hydrophobic residues probably stabilizing the subdomains by holding together the β-sheets (Figure 3e and Figure S2, blue). The PN1-cluster in AcrB-Ec comprises I45, V61, I65 and V127, and the DN-cluster comprises M184, V203, I207, L251, L262, V265 and V771 (Table 2).

An interesting highly conserved tryptophan (W187), partly facing the periplasm, is also observed (Figure 3e and Figure S2, Table 2). According to a cryo-EM structure of AcrAB–TolC, this residue lies between two AcrA-Ec monomers and seems not to be in the direct vicinity of AcrA-Ec (PDB accession code 5O66 [48]). Being close to the arm of the adjacent monomer (pinning through the head domain of the following monomer), it is likely important for the stabilization of the trimer complex, possibly interacting with the neighboring P223 (Table 3, Figure S2) from the other protomer. This P223 residue is also conserved among all pumps, except for the MexI/W-clustered pumps (including LpeB-Lp and AcrB-Hi), where the arm seems to be around six amino acids shorter than the other pumps (data not shown), and, interestingly, MexY-Pa. W187 is present in all analyzed transporters, except for two pumps from *Legionella pneumophila*, being Phe (“MexI/W”) or Thr (LpeB-Lp), see Table 2. These two pumps also do not have the P223 residue on their truncated MexI/W-like arms. More images of the heat map of the periplasmic domain can be found in Figures S1–S3. A recent phylogenetic study found that—similar to our recent study on ancient AcrB-Hi (which is close to, or belongs to, the MexI/W cluster) [15]—MexI and MexW form a phylogenetic cluster, in between the Mdt-like cluster (including MdtB and MuxB) and the Acr cluster (including AcrB and MexB) [62]. It would be interesting for future research to study this distinct cluster’s members in more detail (see Discussion).

### 3.3. Partly Conserved Residues in the Binding Pockets

For this review’s conservation heat map (Figure 3b–e and Table 1 and Table 2), there is a thin line between a residue being classified as fully conserved (red), highly conserved (light red/orange/yellow) or even not listed at all, e.g., when there are only one or two exceptions among the 135 sequences. There may be a few more functionally important relatively conserved residues, which is why we also analyzed the pumps by ConSurf, of which the most conserved residues (corresponding to the darkest purple category in Figure 3a and Figure S3) are listed in Table 3. Other residues of interest can be found in Table S1 (listing all aligned residues, including their conservation percentages and alternative substitutions). Despite no residues in the binding pockets being fully conserved according to the multiple sequence alignment, a few residues are partly conserved among most efflux pumps within the drug-binding regions, we which will discuss further below and in Section 4 and Section 6.

Table 4 lists the loop sequences of the 19 selected pumps, and Table 5 and Table 6 compare their DBP and PBP residues (including conservation among 135 pumps), based on the AcrB-Ec amino acids and their numbering. Fully conserved residues, as compared to AcrB-Ec, are highlighted with a green background. Two of the most conserved residues in Table 5 (DBP) are AcrB-Ec’s F178 and F628 (~70% and ~74% conserved in 135 pumps), located in the hydrophobic pit. F178 is sometimes replaced with Trp (MexY-Pa, MexQ-Pa, LpeB-Lp; red background), Tyr (in AcrD) or Leu (MexW-Pa). F628 is different only in the MexI/W-like proteins, namely MexI/W-Pa, LpeB-Lp and AcrB-Hi, as Gly, Val or Ile, respectively. Another clearly visible relatively conserved residue in the DBP is Y327 (~81% conserved among 135 pumps), which in all 135 pumps is replaced occasionally by Phe (in ~12% of the pumps), by Ile in LpeB-Lp and very rarely by charged residues (see Table S1). This residue has been shown to be part of a postulated entrance channel between the PC2 and PN1 groove, specific for carbonated β-lactams [73], where compounds from the TM1/TM2 interface (fusidic acid, cloxacillin, piperacillin and other carboxylated drugs) translocate via TM2 to this entrance channel [74]. Q176 in the DBP is also party conserved in a selection of pumps (~59% conserved among 136 pumps, including AcrB-Ec, AcrF-Ec, MexB-Pa, MexD-Pa, AdeB-Ab, AcrB-Hi and more, see Table 5). This “Q176D” residue in MexY-Pa contributes to the high number of negatively charged residues in the DBP, possibly explaining aminoglycoside recognition (Section 4.2). A list provided below shows a few occasionally conserved residues for eight well-studied pumps. From this list and Table 5 and Table 6, it is visible that compared to AcrB-Ec, MexD-Pa and AcrB-Hi have the least conserved residues of the eight pumps (16 and 17 in both pockets, respectively), and MexB-Pa has the most (31 residues in both pockets). In the PBP, highly conserved residues are L674 and G675 (~72% and 88% conserved, respectively), conserved for most clades of transporters, including AcrD-Ec, while not strictly for the MexI/W-like pumps (MexI/W-Pa, LpeB-Lp and AcrB-Hi, see Table 6). These residues are located at the lower cleft entrance of Channel 2 of the PBP, on the partly conserved flexible-loop (or F-loop, e.g., AcrB-Ec PAIVE**LG**T, AcrD-Ec PAISG**LG**S and AdeB-Ab PAIDE**LG**T, bold underlined, Table 4). This loop’s initial proline (P669) (underlined) also seems to be conserved (~93% conserved among 135 pumps). Mutating the L674 residue to Trp in AcrB-Ec showed decreased drug efflux [51]. On this flexible-loop, another residue (I671 in AcrB-Ec) is partly conserved as a Leu, Ile or Val (~58% conserved among 135 pumps, Table 6), which has been shown to be implicated in drug selectivity of small substrates [75]. A comparison between all the loops (flexible-loop, switch-loop, distal-loop and interface-loop) is provided in Table 4. The switch-loop between the PBP and the DBP consists of seven amino acids, of which one Gly is fully conserved among 19 pumps (G619 in AcrB-Ec) and ~72% conserved among 135 pumps. Mutating this Gly to Pro in AcrB-Ec inhibits the pump, showing the importance of this loop’s flexibility [51]. Despite the observation that the switch-loop is located near erythromycin in the PBP of the access monomer [51], the loop seems unnecessary in the binding of erythromycin, as erythromycin was found in the same location in both Ala-substituted loop mutants and loop-deleted mutants of AcrB-Ec [76]. S824 is also conserved in most of the 19 pumps (Table 2 and Table 6, and as seen in the list below), although this residue is far from drug-binding residues in the crystal structures quite deep into the PC2 subdomain, and it is not clear if this residue is important for drug recognition. Interestingly, substitutions of this residue (S824I in LpeB-Lp and S821A in MtrD-Ng) are found in drug-resistant strains (see Section 5). The three highest conserved residues in the PBP are G675 (~88% conserved among 135 pumps), T91 (~70% conserved) and Q569 (~69% conserved). As most residues within the pockets of the pumps are relatively variable while, simultaneously, the TM domain is highly conserved, it underpins just how versatile the adaptations of these pumps may be in regard to substrate recognition, and probably in substrate recognition optimization based on specific physiological functions these pumps play within the natural environments of each bacterial species. Later in this review, we will discuss the differences in substrate recognition and binding pocket residues and other properties to help explain the differences between the pumps in the porter domain.

We here list the conserved residues in the DBP compared to AcrB-Ec, among seven additional characterized efflux pumps, in order to provide a quick overview. Bold underlined text highlights conserved in all eight pumps (including AcrB-Ec), and italic underlined text highlights conserved in six out of eight pumps:

- AcrD-Ec F610, *F628*, S180, E273, D276, *G288*, G290, K292 and **Y327**
- MexY-Pa F615, *F628*, S46, E130, S135, *G288*, K292, **Y327**, M573 and V612
- MexB-Pa *Phe-pit*, V139, Q176, S180, I277, A279, *G288*, K292, **Y327**, V571, V612 and R620
- MexD-Pa *Phe-pit*, E130, Q176, S180, E273, I277, *G288* and **Y327**
- AdeB-Ab F136, *F178*, F617, F628, S46, E130, S134, Q176, **Y327** and M573
- MtrD-Ng *Phe-pit* (except F610), S134, L177, E273, *G288*, **Y327**, M573 and V612
- AcrB-Hi *F178*, S46, S128, S135, Q176, E273, N274, A279 and **Y327**

Additionally, the same is conducted for the residues in the PBP. Bold text means present in six out of eight pumps, and italic underlined text highlights conserved in five out of eight pumps:

- AcrD-Ec **S79**, *T91*, *Q577*, **I671**, **L674**, **G675**, D681, R717, N719 and E826
- MexY-Pa T91, Q569, M575, N667, **L674**, **G675**, D681 and **S824**
- MexB-Pa **S79**, *T91*, Q569, *Q577*, M662, F664, F666, E673, *L694*, G676, D681, R717, N719, E826 and L828
- MexD-Pa Q577, **I671**, **L674**, **G675** and **S824**
- AdeB-Ab **S79**, *T91*, Q569, *Q577*, **I671**, E673, **L674**, **G675**, T676 and **S824**
- MtrD-Ng **S79**, Q569, *Q577*, **I671**, E673, **L674**, **G675**, R717 and **S824**
- AcrB-Hi **S79**, *T91*, F666, N667, **I671**, **S824** and E826

## 4. Binding Pocket Differences Help Understand Drug Recognition Spectra

There are several clades of distinct efflux pumps among the homotrimeric RND multidrug efflux pumps with distinct or divergent efflux properties. As mentioned before, we previously analyzed about 400 efflux pump genes and found clades for several groups of pumps, which could be clustered into AcrB/AcrF, AcrD, MexB, MexD/MexY, AdeB, MexF/MexQ and MexI/MexW [15]. Among these pumps, drug recognition can slightly or significantly differ [91]. However, interestingly, phylogenetically distant and ancient AcrB from *H. influenzae* (AcrB-Hi) can export the same compounds as AcrB-Ec (including macrolides, β-lactams and dyes), but it exports bile salts significantly less efficiently [15]. Additionally, ABI-PP could not inhibit AcrB-Hi [15], while it inhibited AcrB-Ec completely [15,36]. Other classes of drugs may also be exported by one pump, but not by another. These include aminoglycosides and monobactams [92]. To further investigate the differences between several transporters, we compared the aforementioned 19 pumps by looking at their amino acids, the hydrophobicity of the pockets and the number of charged and hydrophobic residues, in order to help understand different drug specificities.

### 4.1. Differences in the Hydrophobic Trap of the Distal Binding Pocket

**Figure 4:**
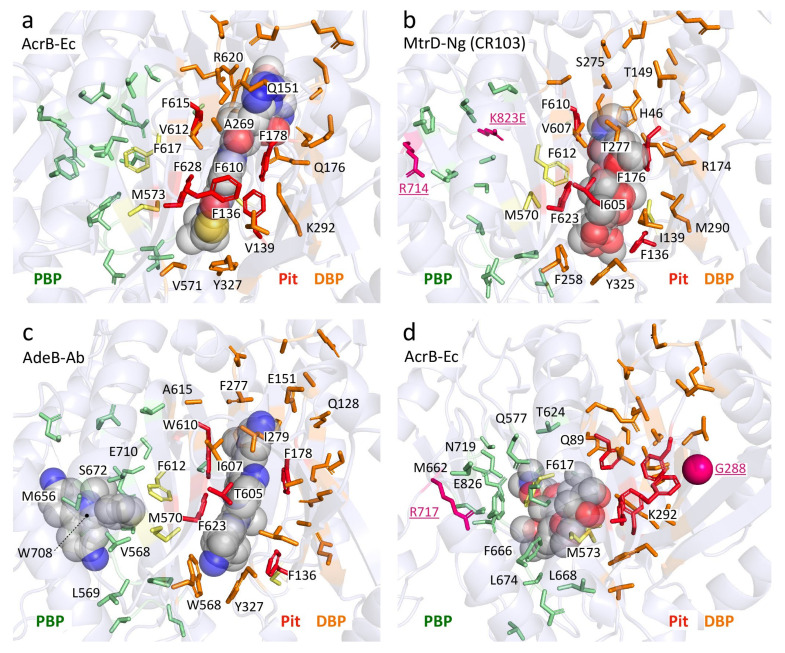
Drug-bound structures of AcrB-Ec, MtrD-Ng and AdeB-Ab**.** (**a**) ABI-PP bound in the binding monomer of AcrB-Ec (PDB accession code 3W9H [36]). (**b**) Erythromycin bound in the binding monomer of “CR103” MtrD-Ng (PDB accession code 6VKT [43]). (**c**) Ethidium bound in the binding monomer of AdeB-Ab (PDB accession code 7KGG [41]). (**d**) Erythromycin bound to the access monomer of AcrB-Ec (PDB accession code 3AOC [51]). A front view of all four structures can be found in Figure S4. Colors: green sticks show the PBP; orange sticks show the DBP; red sticks show the hydrophobic pit; pink highlights recurring substitution locations in clinical strains.

Table 5 showed the aligned residues within the DBP. These 30 residues in the DBP were selected for comparison based on drug-bound structures and MD simulations, namely: minocycline- and doxorubicin-bound AcrB-Ec [52], erythromycin- and rifampicin-bound AcrB-Ec [51], doxorubicin- and minocycline-bound AcrB-Ec [54], ABI-PP-bound AcrB-Ec and MexB-Pa [36], molecular simulations of multiple drugs to AcrB-Ec [55], a mutation study in AcrB-Ec [75] and ampicillin- and erythromycin-bound MtrD-Ng [43]. The DBP includes the hydrophobic pit, or inhibitor binding pit, which is a phenylalanine-rich pit in, e.g., AcrB-Ec [36,58] and MexB-Pa [36]. For most of the 19 selected pumps (excluding AcrD), these pit residues are hydrophobic residues, except for a Thr in AdeB-Ab; however, this residue has a hydrophobicity between Tyr and Trp, according to the hydrophobicity scale used in this review, based on transmembrane helix insertion [93]. The inhibitor binding pits in MexI-Pa, MexW-Pa, AcrB-Hi and LpeB-Lp (“MexI/W cluster”) are significantly different when compared to pumps such as AcrB-Ec, MexB-Pa and MexY-Pa (“Acr cluster”). LpeB-Lp is the least conserved compared to AcrB-Ec when looking at the residues in the DBP and PBP (9 out of the 53 residues, Table 5 and Table 6). Interestingly, the differences in AcrB-Hi (16 out of 53 residues conserved) do not contribute to a fundamentally altered drug efflux spectrum; we showed that AcrB-Hi has a similar spectrum to AcrB-Ec (including macrolides, dyes and β-lactams) [15]. Drug-bound structures of AcrB-Ec, AdeB-Ab and MtrD-Ng show the different amino acids mentioned in Table 5, while all have drugs bound at the same location in the DBP, where different subsets of amino acids interact with the drug molecules (Figure 4 and Figure S4). Figure 4a shows ABI-PP bound to AcrB-Ec, tightly bound in the narrow pit [36]. In the same location, we can see much bulkier erythromycin bound in MtrD-Ng (Figure 4b) [43], where the pit seems to be somewhat wider than for ABI-PP-bound AcrB-Ec. Figure 4c shows two ethidium molecules bound to AdeB-Ab’s DBP and one in the PBP [41].

From the residues in the DBP shown in Table 5 and Figure 4, two conclusions can be drawn: 1) the hydrophobic pit (and DBP) of all transporters contains hydrophobic residues (except for AcrD, which is discussed later on), partly explaining the similar drug recognition spectra and binding structures of the different pumps, and 2) the rest of the DBP is largely not conserved, the only exception being Y327 (see the written list above). As mentioned before, Y327 has been shown to be implicated in carboxylated β-lactam recognition [73]. When looking only at residues as a recognition factor, a few residues are selectively conserved in and near the hydrophobic pit (such as F136, F178, F628 and Y327) and play a role in drug recognition efficiency, and they can also be seen in Figure 4. However, converting AcrB-Ec’s Phe residues to Ala did not disrupt the substrate export completely, although the MIC values were usually significantly lower, and the most profound effect was found for F610A [94]. This mutation is believed to alter subtle properties in the pit, resulting in inefficient drug export rather than directly disrupting drug binding [95]. Interestingly, another transporter from *H. influenzae* (AcrB-Hi) only has the F178 residue conserved, and the F136 residue is a Gly, all while this transporter can export the same compounds as AcrB-Ec very efficiently when expressed in *E. coli* cells (when analyzing the protein by homology modeling, the F610 residue may even be a charged Glu) [15]. As seen for AcrB-Hi and other MexI/W proteins (Table 5), alignment causes gaps in areas of interest (binding pockets and the extruded arm too), and actual crystal or EM structures would potentially give interesting new insights into the mechanism of these pumps and actual structural differences between these transporters and other well-defined pumps (such as AcrB-Ec, MexB-Pa, MtrD-Ng and AdeB-Ab). The conserved Y327 interacts with ABI-PP in AcrB-Ec [36] (Figure 4a), erythromycin in MtrD-Ng [43] (Figure 4b) and several substrates in MD simulations [55]. A recent ethidium-bound AdeB-Ab structure shows ethidium interacting with this conserved Tyr, too (Figure 4c). Y327 is located in the lower vicinity of the hydrophobic pit and the DBP (Figure 4), and in AdeB-Ab next to a Trp (W568, which is V571 in AcrB-Ec). This is AdeB-Ab’s second Trp in the DBP, together with W610 (which is F615 in AcrB-Ec) on the switch-loop, both interacting with an ethidium molecule [41] (Figure 4c, Table 5). More on Y327 in Section 4.6

### 4.2. Differences between Distal Binding Pockets Explain Aminoglycoside Selectivity

The biggest outliers in terms of DBP conservation are the AcrDs (AcrD-Ec and AcrD-Sa), where the pit consists of Asn, Ser, Pro, Tyr and two Phes (Table 5). Thus, this pit is significantly more hydrophilic than the pits of the other transporters [96]. Table 7 shows the hydrophilicity (based on the sum of the residues calculated from [93]). AcrD has a DBP hydrophilicity value of around 39–40 kcal mol^−1^ (Table 7, green), while the DBPs of AcrB-Ec, MexY-Pa, AcrB-Hi, MtrD-Ng and AdeB-Ab are significantly more hydrophobic (25.6, 27.0, 17.3, 16.9 and 21.7 kcal mol^−1^, respectively, Table 7). AcrD’s significantly hydrophilic pit (in combination with the many differences in the residues themselves) can explain why AcrD-Ec exports aminoglycosides, while many other drugs (e.g., erythromycin, ciprofloxacin, tetracycline and many more drugs which are substrates of AcrB-Ec) are poorly exported or not exported at all [35,97,98,99]. AcrD-Ec also has the ability to export, e.g., monobactams, which AcrB-Ec cannot, and this phenotype can be explained by differences in the PBP (Table 6), which will be explained in more detail in Section 4.4.

While the differences in the DBP (both residues and hydrophobicity) explain both aminoglycoside recognition and the inability to export many other drugs by AcrD-Ec, they do not explain a similar phenomenon between MexY-Pa and MexB-Pa [100]. These two pumps are phylogenetically closer to each other than AcrB-Ec and AcrD-Ec [15,100,101], and both show similar hydrophilicity in the DBP of around 26–27 kcal mol^−1^ (Table 7). MexB-Pa and MexY-Pa both have a broad substrate range (especially when compared to AcrD-Ec), including erythromycin, tetracycline, chloramphenicol and more. However, interestingly, MexY-Pa has the ability to also export aminoglycosides [56]. Table 7 shows the number of charged (K, R, D, E) and hydrophobic (I, L, F, V, C, M) residues in both the DBP (from Table 5) and PBP (from Table 6). As seen in Table 7, there is a striking difference in the number of negatively charged residues between MexB-Pa and MexY-Pa in the DBP. MexB-Pa has five positively charged residues (3xK, 2xR, Table 7, orange) and only one negatively charged residue (1xD), while MexY-Pa harbors mainly negatively charged residues (3xE, 3xD, Table 7, green), with only one positively charged residue (1xK). These differences were also observed in computer simulations, where more charged residues are accounted for [102], and a recent study comparing the two pumps in more detail [101]. This significantly negatively charged DBP could explain why MexY-Pa has the ability to export aminoglycosides besides having a broad substrate range (possible by also having a hydrophobic pit), offering a different explanation than for AcrD-Ec.

### 4.3. Bulky Tryptophan in the Inhibitor Binding Pit Prevents Inhibition

Another critical difference (for inhibitor design) between MexY-Pa and MexB-Pa is the presence of a bulky Trp in MexY-Pa, which explains why the inhibitor ABI-PP is not inhibiting this pump [36]. This bulky tryptophan (represented by F178 in AcrB-Ec) is also present in MexQ-Pa and LpeB-Lp (Table 5, red background). Fairly recent studies indicate that LpeB-Lp is an upcoming efflux pump over-expressed in a selection of clinical strains of *L. pneumophila* (the “Paris strain”) causing macrolide resistance [103,104]. This fuels the urge for the development of novel universal efflux pump inhibitors which have overcome this bulky Trp hindrance. Two pumps (AdeB-Ab and MexI-Pa) have a Trp in AcrB-Ec’s F615 location (located on the switch-loop, Table 4 and Table 5); however, these residues possibly do not interfere much with the space of the pit because it is located on the flexible-loop and is located at the “top” of the pit, rather than deeper into the pit itself (Figure 4c, W610 in AdeB-Ab interacting with an ethidium molecule).

### 4.4. Specific Amino Acids in the Proximal Binding Pocket Explain β-Lactam Selectivity

Table 6 compared the residues in the PBP. As mentioned, AcrD-Ec and MexY-Pa can both export aminoglycosides (while AcrB-Ec and MexB-Pa cannot), explained by the differences in hydrophobicity or the number of negatively charged residues in the DBP. However, AcrD-Ec can also effectively export both monobactams (such as aztreonam) and anionic β-lactams (carbenicillin and sulbenicillin). AcrB-Ec cannot export aztreonam and can only weakly export carbenicillin and sulbenicillin [99]. Also, while MexY-Pa and AcrD-Ec can both export aminoglycosides, MexY-Pa is unable to export carbenicillin and sulbenicillin [105,106]. Three residues of interest are AcrB-Ec’s respective Q569, I626 and E673. These residues are charged Arg (R568 and R625) and Gly (G672) in AcrD-Ec and AcrD-Sa. These three residues are implicated in monobactam (aztreonam) and anionic β-lactam (carbenicillin and sulbenicillin) selectivity in AcrD-Ec, and substitution of these residues in AcrB-Ec (Q569R/I626R/E673G) as a triple mutant adds or increases the efflux ability of AcrB-Ec for these three β-lactams [99], providing an explanation to why AcrD-Ec can export aztreonam, carbenicillin and sulbenicillin, while MexY-Pa and AcrB-Ec cannot (or only weakly) [99,100,105]. There are more differences between these efflux pumps, such as the ability of MexB-Pa to efflux imipenem, meropenem, carbenicillin and sulbenicillin, which is not recognized by MexY-Pa [101,105]. Perhaps the differences in charged and hydrophobic residues account for these specificities (Table 5, Table 6 and Table 7), as MexB-Pa does not have AcrD’s Arg and Gly residues in the PBP. Additionally, it is interesting to note that despite the significant substrate specificity differences between AcrB-Ec and AcrD-Ec, both transporters can export certain β-lactams (e.g., nafcillin) and SDS very effectively [99]. Perhaps similarities (10 residues in the list above) in the PBP explain this phenomenon. However, even less conserved MexD-Pa (five conserved residues in the PBP) also has the ability to export nafcillin [105]. A short discussion regarding certain β-lactam (including nafcillin) export abilities by many phylogenetically distinct and distanced efflux pumps is provided in Section 4.6.

### 4.5. Adaptation through Amino Acids and Hydrophobicity Alterations May Increase Activity

Another interesting PBP difference presented in Table 6 is the presence of a third Trp in AdeB-Ab (W708), which is a charged Arg in AcrB-Ec (R717), located at the entrance of the PBP. This Trp interacted with a third ethidium molecule in a recent cryo-EM structure [41] (Figure S4c). This R717 location is also a hotspot for RND pump mutations in clinical strains (Table 6, and Figure 4b,d in pink) which we will discuss later (Section 5). AdeB-Ab seems to be unique in having two Trp in the DBP and one in the PBP. Just one other transporter in the list of 19 pumps (LpeB-Lp) holds three Trp residues, but in different locations (compared to AcrB-Ec: F178W (same as MexY-Pa, mentioned before) in the DBP, and M662W and F664W at the entrance (Channel 2) of the PBP). We recently found that ethidium efflux is enhanced by double Trp mutations (T37W/A100W) at the Channel 3 entrance in AcrB-Ec [72]. Table 7 shows differences in hydrophobicity of the DBP. AdeG-Ab has the least hydrophilic pocket (∆G = 10.2 kcal mol^−1^), compared to AcrB-Ec (25.6 kcal mol^−1^), MexB-Ec (26.2 kcal mol^−1^) and AcrD-Ec (39.6 kcal mol^−1^). Interestingly, there is a significant difference in the number of both charged (K, R, D, E) and hydrophobic (I, L, F, V, C, M) residues between AcrB-Ec and AcrB-Hi (Table 7, yellow). AcrB-Ec has five charged residues, while AcrB-Hi only has one. Additionally, AcrB-Ec has 12 hydrophobic residues, while AcrB-Hi merely has seven. The same goes for the number of hydrophobic residues in the PBP between AcrB-Ec and AcrB-Hi (nine vs. five, respectively). At the same time, as mentioned before, the efflux spectrum of these transporters is almost the same (with the exception of bile salts) [15]. In our previous study, we determined the expression levels of AcrB-Ec and AcrB-Hi in *E. coli* cells to be similar, while AcrB-Hi could export most drugs less effectively with a several-fold lowering of the MICs of certain drugs (such as methicillin and cefcapene pivoxil), a similar MIC for other drugs (such as ethidium or cloxacillin) and, interestingly, a significantly lower ability to export bile salts (including deoxycholic acid) [15]. Perhaps, looking at the presented data in this review, AcrB-Ec (and other evolved transporters) has adapted to have both more charged and hydrophobic residues to increase drug efflux efficiency and accommodate physiologically relevant compounds. For example, AcrB-Ec, AcrD-Ec and MtrABC-Ec have been shown to be involved in enterobactin export [107]. Other differences obtained through evolution may be the Pro (“P223”) on the arm (absent in the truncated arms of AcrB-Hi and MexI/W-like pumps) and the Trp residue (“W187”) in the DN subdomain, possibly enhancing the stability of the trimer. The differences in hydrophobicity, number of charged residues (specifically positively and negatively charged residues), number of hydrophobic residues and the volume of the pockets can help us understand the substrate recognition differences and the differences in the efficiencies of the export of specific drugs between different pumps.

### 4.6. Conserved Residues May Partly Explain Conserved Drug Specificities

Comparison of substrate specificities between MexD-Pa, MexY-Pa and MexB-Pa shows that among these pumps, many classes of antibiotics are recognized and exported (including quinolones, macrolides and tetracycline), with distinct differences between them (e.g., for imipenem, carbapenem, carbenicillin, sulbenicillin, ceftazidime, meropenem and more) [105], even though these three pumps differ significantly in pocket residues (Table 5 and Table 6). Between MexB-Pa, MexY-Pa and MexD-Pa, the conserved pocket residues are G290, F615, F628 and Y327 in the DBP, and L674 and G675 in the PBP.

Between six pumps, namely, AcrB-Ec, AcrD-Ec, MexB-Pa, MexD-Pa, MexY-Pa and AcrB-Hi, we compared the substrate specificities for a selection of drugs and drug classes listed in Table 8. We found that one class of antibiotic was exported by all six pumps, namely, cloxacillin, oxacillin and/or nafcillin (second-generation narrow-spectrum penicillins) β-lactams. This may also include first-generation penicillins (such as benzylpenicillin) or fourth-generation extended-spectrum β-lactams (such as piperacillin), but these were not tested for all pumps. Therefore, we could at least conclude the second generation to be widely exported (Table 8, green). The six pumps have different hydrophobic properties in the PBP and DBP (Table 7) and, within the binding pockets (DBP and PBP), only have one conserved residue among them: Y327 in the DBP. The overlap in the substrate range may be partly explained by this residue, as well as the aforementioned fully conserved N298 (outside the pockets, near the Channel 3 entrance). A recent study found that a Y327A mutation (postulated to be implicated in a novel substrate entrance Channel 4) caused a decrease in drug resistance against carboxylated β-lactams such as dicloxacillin and oxacillin, and that the N298A mutant of AcrB-Ec decreased drug binding for the specific compounds seen in the crystal structures and is reflected in the MIC data [73]. Another study found that these drugs (in addition to fusidic acid) are translocated via a TM1/TM2 groove [74]. We found one of the implicated TM residues (I337) to be highly conserved within the analyzed 135 pumps (Table 1 and Table 2), and a recent study found the mutation I337A to have the largest impact among the tested mutations for the MICs of specific compounds (for fusidic acid, oxacillin, etc.) and hardly for erythromycin [74]. As mentioned above, it would be interesting to determine the structure of phylogenetically distanced AcrB-Hi-like pumps to further understand the recognition determinants and pocket residues. Other differences besides residues in binding pockets (such as differences in the volume of the pockets, movements of loops, distances between loops, interactions with substrates to residues and the importance of water within the pockets and channels) between different pumps are investigated by molecular dynamics to try to explain specific differences between these pumps and their mechanisms (which cannot always be readily understood by only comparing the residues within the pockets) [96,101,102,108,109].

## 5. Recent Mutations in RND Multidrug Efflux Pumps Cause Enhanced Drug Resistance

As seen in Figure 3 and Table 1 and Table 2, the conservation of RND multidrug efflux pumps is mainly present in the transmembrane domain and indicates that the porter domain is flexible to adapt to changes in the environment of the bacterial cells, explaining divergences in drug recognition spectra between different pumps. RND transporters are known to be promiscuous transporters, as they can recognize and transport a large number of structurally different compounds [12]. These substrates are surrounded by a multitude of residues and loops in two voluminous binding pits (Table 4, Table 5, Table 6 and Table 7) and enter the pump through a multitude of channels [51,72,73,74,75,110]. It is fascinating that these efflux pumps can expel not only a wide range of drugs but also differ significantly in their amino acid composition within the binding pockets, while between these pumps, the substrate recognition spectrum is highly conserved (with the exceptions of some drugs, such as monobactams and aminoglycosides, and divergent efficiencies). Differences and evolved properties in binding pockets described above may have given transporters a more efficient export ability. In this last section, we will describe novel amino acid substitutions in RND multidrug efflux pumps, which have been arising recently in clinically, environmentally and laboratory-evolved strains. Previously displayed Table 5 and Table 6 partly identify the location of the mutations in the DBP and PBP, highlighted by asterisks (*). An overview of recent mutations found in different pumps from different organisms can be found in Table 9. These recently spreading mutants significantly further enhance the efflux ability of intrinsically expressed efflux pumps (gain-of-function mutations) and have already proven to be a major problem in treating severe infections. Our over-usage and misusage of antibiotics have been putting extreme selective pressure on bacterial pathogens, causing an uprise of these mutated, highly efficient RND efflux pumps.

### 5.1. AcrB-Sa Mutants Cause Fluoroquinolone (G288) and Macrolide (R717) Resistance

**Figure 5:**
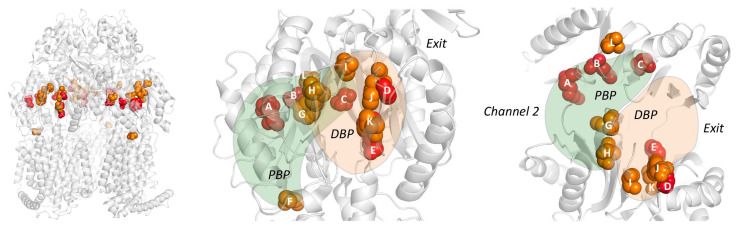
Upcoming gain-of-function mutations in RND-type efflux pumps. Resistant clinically, environmentally and laboratory-evolved strains show an alarming rise in gain-of-function mutations in the binding pockets of homotrimeric RND multidrug efflux pumps. Residues are shown as spheres. From left to right: view of the whole AcrB-Ec trimer, side view and top-down view. Red spheres show the most recurring amino acid substitution (by nonsynonymous mutations in the genes) numbered by letters (A–E), being G228D (AcrB-Sa, “D”), G287A/S (MexY-Pa, “D”), G288C/M/S (AcrB-Ec, “D”), G288S (AdeJ-Ab, “D”), R717Q/L (AcrB-Sa, “A”), R714C/G/H/L (MtrD-Ng, “A”), K823D/E/N (MtrD-Ng, “B”), F136L (AdeJ-Ab, “E”) and K79A/T (MexY-Pa, “C”). Orange shows mutations found in resistant strains (letters F–L), potentially increasing drug resistance; however, the direct effects of the specific mutations have not yet been significantly investigated, or the effect is not clear. These include F178S (“J”), A562V (“F”) and M626V (“H”) (MexB-Pa), S824I (“L”) (LpeB-Lp), V139F (“K”), A279T (“I”) and F617L (“G”) (AcrB-Ec) and S821A (“L”) (MtrD-Ng). The mutations P319L and M78I (AcrB-Sa, Table 9) are not shown, as they are both not present in the binding pockets. Colors: green, proximal binding pocket (PBP); light orange, distal binding pocket (DBP). PDB accession code 3W9H [36].

Blair et al. (2015) reported a mutation in the AcrB-Sa efflux pump found in a post-therapy *Salmonella* Typhimurium clinical isolate, which caused a fatal infection. Table 9 lists recent mutations found in bacterial strains causing increased MDR. The *Salmonella* residue substitution was G288D in AcrB-Sa, a novel mutation causing fluoroquinolone ciprofloxacin resistance (MIC 32- to 64-fold increase pre- vs. post-therapy) [80]. In the same study, concerning the G288D mutation on AcrB-Sa-expressing plasmids, antimicrobial MICs were also increased for other antimicrobials, e.g., chloramphenicol and tetracycline (although doxorubicin export was decreased, also when the mutation was conferred in AcrB-Sa expressed in *E. coli*). Computer simulations in the same study demonstrated that the charged Asp residue protruded through to the hydrophobic pit, altering the hydrophobicity and causing steric clashes with residues in this pit, changing their conformation (especially F178 and Q176), and increased the radius of gyration of the DBP by roughly 10% [80]. Figure 4d shows the location of G288 in AcrB-Ec (shown as a pink ball in the DBP) which is also highlighted in Table 5 and shown in Figure 5. Interestingly, the G288 mutation has also been found in AcrB-Ec, MexY-Pa and AdeJ-Ab (explained in Section 5.3, Section 5.4, Section 5.5). The G288 residue is somewhat conserved, as seen in Table 5 (~50% Gly and ~20% Ala, Table S1). However, interestingly, “G288” is substituted by more bulky residues in the MexI/W-clustered transporters (being Val (~6%), Tyr (~4%) or His (~1.5%)). Additionally, for this reason, and as mentioned before, it would be interesting to study these members in more detail in future research.

In recent years, other AcrB-Sa mutants have been observed, causing untreatable infections in Nepal, Bangladesh, India and Pakistan, in both *Salmonella* Typhi [85,86,87,88] and *Salmonella* Paratyphi A [85,88], summarized in Table 9. These clinical isolates are resistant to azithromycin (macrolide) by the mutations R717Q and R717L in AcrB-Sa. Hooda et al. (2019) identified 13 azithromycin-resistant *Salmonella* strains (12 Typhi, 1 Paratyphi A) from around 1000 hospital isolates from Bangladesh, with MIC values between 32 and 64 μg ml^−1^, with the first strain isolated in 2013 [85]. The 12 *Salmonella* Typhi AcrB-Sa genes had an SNP at R717 substituted with a Glu (R717Q), and the *Salmonella* Paratyphi A AcrB-Sa had an R717L mutation. Both mutations in AcrB-Sa showed a decrease in erythromycin and azithromycin (both macrolides) susceptibility. Similarly, Iqbal et al. (2020) described azithromycin-resistant *Salmonella* Typhi strains from Pakistan. The isolates cause severe problems during treatment, as extensively drug-resistant (XDR) *Salmonella* Typhi has left azithromycin as one of the last treatment options. Here, too, the R717Q mutation in AcrB-Sa was identified as the reason for this resistance [86]. *Salmonella* Typhi isolates from Nepal harboring the R717L mutation have been described by Duy et al. (2020), also responsible for azithromycin resistance. They note that none of the analyzed strains had an acquired AMR gene. Importantly, the authors also described that these mutants had divergently emerged in both Nepal and Bangladesh among the so-called H58 lineage, suggesting that selective pressure caused by treating typhoid fever with azithromycin resulted in these resistant strains independently [111]. Katiyar et al. (2020) analyzed two azithromycin non-susceptible strains (from 133 clinical isolates from patients with typhoid fever) from India, which both had the R717Q mutation in AcrB-Sa [87]. Another recent study by Sajib et al. (2021) predicted that the R717 mutation first occurred somewhere in 2010. They also described a *Salmonella* Typhi isolate from the United Kingdom harboring the AcrB-Sa R717Q mutation. In the same study, the authors analyzed 2519 *Salmonella* Typhi isolates and 506 *Salmonella* Paratyphi A isolates from Bangladesh, of which 104 isolates were azithromycin-non-susceptible. Of these, 32 *Salmonella* Typhi and 6 *Salmonella* Paratyphi A isolates had a significantly high azithromycin MIC (>32 mg ml^−1^). All of these 32 highly resistant Typhi isolates had the R717 mutation (29 R717Q and 3 R717L), and five Paratyphi A isolates had the R717Q mutation [88]. It is clear that the spontaneous and divergent emergence of the “R717 mutations” in AcrB-Sa should raise great concern for the treatment of typhoid fever by macrolides.

Lastly, recently, P319L and M78I/P319L mutants of AcrB-Sa have been found in *Salmonella* ssp. strains isolated from pork, swine, chicken and duck from Guangdong, Shandong, Hubei, Henan (China), causing increased MICs for multiple substrates, of which the most noticeable are fluoroquinolones (enrofloxacin and norfloxacin), but also for erythromycin and other substrates. These two residues are not located in the binding pockets of AcrB-Sa, but more on the outside of the monomers. The authors argued that the P319L residue might increase the export efficiency by altered interaction with AcrA [112].

### 5.2. MtrD-Ng Mutations (R714, K823) by Mosaic Patterns Causes Macrolide Resistance

Recently, mutations in the multiple transferable resistance (mtr) efflux pump from *N. gonorrhoeae* (MtrD-Ng), acquired by mosaic-like patterns in the alleles, have become an increasing concern in azithromycin (macrolide) resistance [120] (Table 9). Mosaic patterns arise in *N. gonorrhoeae* acquiring and recombining donor DNA from *Neisseria* spp. (*N. meningitidis* and *N. lactamica*), resulting in multiple mutations in both the repressor (*mtrR*) and efflux pump (*mtrCDE*) genes, and are found worldwide [121]. It has been extensively studied that (as for most other pathogens) increased resistance in clinal strains can be a result of mutations in the regulatory network (e.g., MtrR or MtrA) [122,123,124]. However, mutations in the efflux pump MtrD-Ng itself (instead of by direct mutations in the 23S rRNA target gene [125]) cause significantly elevated MICs (azithromycin > 2 µg/mL) and are relatively new. Recently, Wadsworth et al. (2018) analyzed 1102 isolates and noticed an increase in mosaic patterns at the *mtrCDE* region, with the highest diversity in the *mtrD* gene [113]. Four residue mutations between isolates were found in MtrD-Ng: I48T (DBP), G59D, K823E (PBP) and F854L. Additionally, in 2018, Rouquette-Loughlin et al. studied eight clinical strains from 2014 and found that mutations directly in the MtrD-Ng protein accounted for an increased azithromycin resistance, which could not be explained only by mutations in the promoter region or in the regulatory network. They identified two mutations, namely, K823E and S821A [24], both on the PC2 subdomain in the PBP of MtrD-Ng. Cryo-EM structures of MtrD-Ng (from transformant “CR103” by [24]) holding the two K823E and S821A mutations were solved recently [43], and the same study identified several other mutations in this pump, including R714G. Both single mutations, K823E and R714G, resulted in an increase in MICs for several substrates (azithromycin, erythromycin and polymixin B) [43]. Additionally, Ma et al. (2020) analyzed 4852 global *N. gonorrhoeae* genomes. Of these, 12 contained the mutation R714H/L/C, and seven contained the mutation K823E/N [89]. They did not observe mutations at positions 74, 669, 821 and 825, as found (and tested) by others [24,43].

Interestingly, the R714H/L/C mutations [43,89] correspond to the R717L/Q mutations discussed before (Section 5.1) present in AcrB-Sa of *Salmonella* clinical isolates from Bangladesh, Pakistan, India and Nepal (Table 9). The location of these mutations (at R714, K823 (and S821)) are all in the PC2 subdomain and face the PBP (Table 6 and Figure 5), which explains the increase in the MICs for macrolides, but, e.g., not for other drugs such as penicillin, ampicillin, ethidium bromide and crystal violet [43]. Although the S821A mutation showed no increase in the MICs of the tested compounds [43], a similar mutation (S824I) was found in the LpeB-Lp efflux pump in *L. pneumophila* clinical isolates from China [90]. However, direct MICs were not determined for this strain, nor was the effect of the mutation determined. It is interesting that these mutations are seen within pumps from different organisms (Table 9), that the Ser residue is highly conserved among the 19 analyzed efflux pumps (represented by an Ala in AcrD-Ec/Sa and MexB-Pa, similar to the S821A mutation in MtrD-Ng) and that the K823E mutation is similar to E826 in wild-type AcrB-Ec/Sa and other pumps (Table 6 and Table S1).

### 5.3. AdeJ-Ab Mutations (G288, F136) Cause Increased Drug Resistance

Mutations in the previously described G288 location (Section 5.1) have also been found in multiple studies on *A. baumannii* clinical isolates. Hawkey et al. (2018) investigated carbapenem-resistant *A. baumannii* isolates from burn wound sites of a 2013 patient to investigate resistance evolution [77]. They analyzed the collected 20 strains from this patient in addition to strains from three other patients (one before and two after the admission of the main investigated patient). All collected strains were multidrug-resistant (to, e.g., aminoglycosides, fluoroquinolones and more); however, they showed variations in meropenem resistance (MICs ranging from 2 to >32 µg mL^−1^). All strains which first showed an elevated MIC of ≥8 µg mL^−1^ contained the mutation G288S on the RND pump AdeJ (the authors mentioned AdeB, but we believe it to be AdeJ after checking the sequences). A later strain harbors mutation F136L, with an MIC of 8 µg mL^−1^, and later isolated strains with this F136L mutation also contained a mutation, A515V, in the penicillin-binding protein (PBP3) FtsI, further increasing the MICs (≥32 µg mL^−1^). After being administered meropenem, the patient was treated with colistin (a last-resort polymyxin treatment) [77]. As explained before, the G288 residue is located near the hydrophobic pit in the DBP, and the G288S mutation possibly altered the drug-binding properties of the pit to meropenem. This can also explain the mutation of F136L, which is also located in the pit, possibly increasing the binding of carbapenems to the pit, although we are not sure of the precise mechanism of these alterations. The F136 location is the least conserved residue of the six Phe residues (with AcrB-Ec as a reference), being a Leu in MexF-Pa and AdeG-Ab, and Ile in MexQ-Pa and MexY-Pa (see Table 5).

Similarly, a recent study by Santos-Lopez et al. (preprint, 2020) investigated the roles of selective pressure by antibiotic treatment of *A. baumannii* laboratory-evolved strains under increased cephalosporin (ceftazidime) and carbapenem (imipenem) concentration conditions. Growth under ceftazidime resulted in mutation in AdeJ, causing resistance to both ceftazidime and imipenem in 16 of the 18 strains (the other two harboring mutations in the *adeIJK* regulatory protein AdeN, or a PBP instead). The mutation in AdeJ found in replicates was G288S. Additionally, other mutations seen in AdeJ were, e.g., F136L, F136S, Q176K, Q176R and A290T, all in the DBP [78]. Q176 in AcrB-Ec interacts with ABI-PP in one of the crystal structures [36], and the G288D mutation in AcrB-Sa alters the Q176 conformation [80]. The recurring F136L and G288S mutations in AdeJ further suggest that these substitutions are significant gain-of-function mutations in this efflux pump.

### 5.4. Mutations in MexY-Pa (K79, G287) Increase Aminoglycoside Resistance

Greipel et al. (2016) studied 361 isolates of people suffering from cystic fibrosis (CF) by analyzing the genome sequences. The isolates came from multiple EU countries (including Germany, Sweden and the Netherlands) [84]. They found 85 nonsynonymous mutations in the *mexY* gene. In two isolates, the G287S mutation was present [84], which is similar to the G288 mutations in AcrB-Sa/Ec [80] and AdeJ-Ab [77]. They also described a “Q175E” mutation in 327 isolates, similar to the Q176 location in AdeJ-Ab and AcrB-Ec mentioned before; however, in wild-type MexY-Pa, this residue is E175 (Table 5), and thus it may be possible that the other 34 strains have an E175Q mutation in the DBP. However, we cannot confirm this. It is an interesting location as mutations here have also been seen in AdeJ-Ab (Section 5.3), and the Q176 residue is one of the somewhat conserved residues among the 19 selected transporters shown in Table 5 (this residue’s conformation is altered in the G288D gain-of-function mutant of AcrB-Sa according to MD simulation (Section 5.1) [80]). Another potentially interesting mutation found in MexY-Pa in the same study was S48N (similar to S48 in AcrB-Ec), located in the DBP, close to the exit. A list of the total 85 nonsynonymous mutations can be found in [84]. Direct MIC measurements looking at the effect of the mutations (G287S, E175Q and S48N) have not been performed. However, the yet again recurring G287S (“G288”) mutation is a worrying find. López-Causapé et al. (2017) sequenced and analyzed 28 strains from 18 patients with CF from Spain and Australia, isolated between 1995 and 2012 [82]. They found mutations in more than 100 genes related to AMR. Besides mutations in repressor MexZ, and genes *gyrA* and *fusA1*, they found mutations in the RND-type efflux pumps, including MexY, MexB and MexW. MexY and MexB had the most different numbers of mutations (nine) in many isolates (8 for MexB and 19 for MexY). One of the most recurring mutations was G287A in MexY (similar to G287S mentioned above), seen in three isolates. In MexB, the F178 location was mutated in one strain (F178S). Strains with the MexY (G287A) mutation had a significantly higher MIC for tobramycin compared to other isolates, although this was due to many different other mutations in multiple genes for different isolates, and the effect of the G287A and F178S mutations was not directly observed. However, their analysis by comparing the median MICs for strains with or without a particular mutation suggested an increase in multiple drugs for mutations in MexY (e.g., imipenem, aztreonam, meropenem and tobramycin). For MexB mutations, a similar increase in MICs was observed; however, this was noticeably more significant compared to MexY for aztreonam and meropenem. The complete overview of mutations can be found in [82].

In a recent study, Wardell et al. (2019) showed that 4 out of 13 laboratory-evolved strains, under tobramycin growth conditions, harbor mutations in MexY-Pa, which did not occur in meropenem or ciprofloxacin selected strains. Three of these four had the mutation G287S (the same as the MexY-Pa mutations found by [84]), and one had the mutation K79T [83]. Besides the recurring G287 mutation, K79T catches our attention, as a mutation in the same location (K79A) was found by experimentally evolved MexY-Pa by selective pressure under aminoglycosides. In that study, the K79A mutant caused a significantly higher MIC for aminoglycosides paromomycin, neomycin and spectinomycin [56]. As the K79T mutation was found in a strain with decreased tobramycin susceptibility [82], it is likely that this PBP location mutation increases the substrate recognition of aminoglycosides by MexY-Pa. The same study [83] also found MexY-Pa mutations in 140 out of 558 (25%) clinical isolates and in 15 out of 172 (8.7%) environmental isolates (although specific mutations were not mentioned), again highlighting the significant variability and frequency of mutations in MDR pump genes. Another recent study by Colque et al. (2020) studied 14 clinal isolates from CF patients from Denmark who suffered long-term infections by *P. aeruginosa* between 1991 and 2011. They found two mutations in MexB-Pa (five times in M626V, and once in A562V, both in the PBP) and six in MexY-Pa (although none in a binding pocket) [5]. In MexB-Pa, the M626V mutation is inside the PBP, while A562V is directed to Channel 1 of the monomer. Similarly, multiple mutations in *P. aeruginosa* RND-type pumps (MexY, MexB, MexD, MexK, MexI, MexQ) were found in an MDR clinical isolate (“PA154197”) from Hong Kong [126]. In both these studies, the direct effects of the mutations were not determined.

### 5.5. Experimentally Obtained Mutations in AcrB-Ec (V139, A279, G288)

Cudkowicz et al. (2019) and Langevin et al. (2020) examined the evolution of mutations in *E. coli* and AcrAB–TolC specifically, respectively, under chloramphenicol growth conditions, and both studies observed the V139F mutation in AcrB-Ec [114,115]. This mutation was also seen by Hoeksema et al. (2019) when analyzing the effects of mutations in genes related to AMR, specifically the role of these mutations in the resistance to a second antibiotic after a first antibiotic gave rise to a specific mutation (where V139F was found in strains resistant to tetracycline, which previously acquired resistance to amoxicillin, enrofloxacin or kanamycin) [116]. This Val residue (V139) is located in the hydrophobic pit in the DBP. It is not clear how this mutation exactly enhances the efflux ability of AcrB-Ec, and if the mutation causes increased MICs for multiple drugs and therefore acts as a significant gain-of-function mutation. The recurrence of this mutation, however, makes it a noteworthy one.

Schuster et al. (2014) found a G288S mutation in most of their evolutionarily evolved strains (after in vitro random mutagenesis of the AcrB-Ec gene), along with G288M, G288C and A279T (also in the hydrophobic pit of the DBP). The MIC data for G288S and A279T (single and double) did not indicate a gain-of-function mutation for the tested compounds (even a decrease in MICs for novobiocin and chloramphenicol) [118]. The A279T mutation was also obtained by researchers who optimized AcrB for the export of styrene and alpha-olefins. Out of eight variants, seven contained A279T and five contained F617L [119]. On the other hand, a G288C mutation was found to be the most recurring in another study by Soparkar et al. (2015) when trying to regain the export ability in the loss-of-function mutation F610A in AcrB-Ec (of which the gene was located on a plasmid, transformed into AcrB-deficient cells). They found G288C to be the most effective suppressor alteration, occurring five independent times. The introduction of G288C in AcrB (F610A) increased the MICs for erythromycin, novobiocin, minocycline, nalidixic acid and SDS (when compared to AcrB (F610A)) [79]. The “G288” mutation is the most recurring mutation with the most alternative amino acid substitutions, as seen in Table 9.

## 6. Discussion

In this review, we provided a conservation analysis of homotrimeric RND-type multidrug efflux pumps, including a more detailed view of 19 selected pumps (which have been better studied). We also looked at the conservation and variation among a selection of pumps to try to summarize, explain and understand the substrate specificities of some pumps, based on specific residues, hydrophobic and hydrophilic residues, in both pockets. The analysis showed that among all efflux pumps, the TM domain was significantly conserved, while the porter domain was largely variable, except for some interesting residues, including the residues D568 from the “interface-loop” and R637 from the PC1 subdomain. Certain residues within the binding pockets were conserved between some pumps, but not all, despite the pumps having a similar efflux spectrum. Interestingly, the least conserved pump (AcrB-Hi) compared with AcrB-Ec can expel the same compounds. We hypothesize that changes in the number of hydrophobic and hydrophilic residues in the pockets may enhance drug efflux and may specifically enhance the efflux of physiologically relevant toxic compounds, such as bile salts. As for the TM domain, the three residues forming salt bridges—playing a crucial role in the proton translocation and therefore energy consumption—are D407, D408 and K940 (numbering based on AcrB-Ec) in most of the 135 analyzed pumps. There were, however, three noticeable outliers for the K940 residue, namely, where the Lys was an Arg residue (for the organisms *I. loihiensis*, *C. japonicus* and *T. turnerae*). This “K940R” residue was also created in MexB-Pa back in 2000 by Guan et al., which resulted in a fully active pump [34], indicating that this region, however critical for the function of the pump, is still slightly flexible by substitutions.

As for the conservation in the porter domain, the conserved residues of interest were the aforementioned “interface-lock” D568 and R637 (both 100% conserved among 135 pumps), N298 (located at the vestibule and close to Channel 3, also 100% conserved), P223 (99% conserved (excluding gaps); present in all analyzed pumps, except for MexI/W members and, interestingly, MexY-Pa (Gly)), W187 (98.5% conserved; possibly stabilizing trimer formation, present in all analyzed pumps, except for two MexI/W-like pumps from *L. pneumophila*) and, to a lesser extent, but still significantly, Y327 (81% conserved; in the hydrophobic pit in the DBP, linked to the recognition of carboxylated drugs), and those partly conserved but noticeable were F178 and F628 (70% and 74% conserved; in the hydrophobic pit), L674 and G675 (72% and 88% conserved; part of the bottom and flexible (F-) loops in the PBP) and the somewhat less conserved Q577 (69% conserved; in the PBP). It is interesting to note that these residues are—as an example—not present in AcrB-Hi (except for F178 and Y327), which has a similar substrate range to AcrB-Ec. We also found it interesting that among the selected transporters, certain carboxylated drugs (including cloxacillin, oxacillin and nafcillin) were exported by all (Table 8), perhaps partly explained by the Y327 residue. A list of all residues with their conservation percentages and alternative substitutions can be found in Table S1. We found that members of the MexI/W cluster (including AcrB-Hi and LpeB-Lp) consistently showed distinct differences when compared to their RND multidrug efflux pump colleagues. These differences are the truncation of (the tip of) the arm, the lack of the conserved P223 on this arm, the gaps after sequence alignment (e.g., in the hydrophobic pit (“F610”) and other parts of both the PBP and DBP), the lack of highly conserved F628 in the pit, the lack of conserved L674 on the F-loop in the PBP and, additionally (although only seen in two pumps from *L. pneumophila*, including LpeB-Lp), the conserved W187 substitutions (being a Phe or a Thr). It would be interesting for future research to obtain structures and biochemical data (in addition to AcrB-Hi [17]) of members of this cluster. In particular, the macrolide resistance causing LpeB-Lp pump (gene lpp2880 from clinically relevant *L. pneumophila* str. Paris [103,104]) not only showed the distinct abovementioned MexI/W characteristics but was also an outlier within this cluster, being the only pump lacking both the conserved W187 and Y327 (Table 2) and showing the most gaps after multiple sequence alignment (Table 5; Table 6).

Besides comparing drug recognition and conservation among pumps, we looked into the rise in recent adaptations of the RND pumps occurring in fairly recent multidrug-resistant clinical strains. We found several noticeable recurring amino acid substitutions present in clinically, environmentally and laboratory-evolved strains. Firstly, G288D (AcrB-Sa), G288C/S/M (AcrB-Ec), G288S (AdeJ-Ab) and G287A/S (MexY-Pa), mutations just outside the hydrophobic pit of the DBP, changed—and usually enhanced—MICs for certain drugs (fluoroquinolones in *Salmonella* Typhimurium, aminoglycosides in *P. aeruginosa*, carbapenems in *A. baumannii* and multiple drugs in *E. coli*). In AdeJ-Ab, another noticeable mutation was F136L, decreasing susceptibility to meropenem. Other worrying mutations are R717L/Q (AcrB-Sa in *S.* Typhi and *S.* Paratyphi A), and R714C/G/H/L and K823D/E/N (MtrD-Ng), mutations in the PBP of the RND efflux pump, increasing the MICs for macrolides (such as azithromycin) considerably (possibly also the mutation S821A, recurring as S821I in LpeB-Lp from *L. pneumophila* strains from China). The mutations K79A/T (MexY-Pa) were independently observed by different research groups in laboratory-grown resistant strains. Lastly, V139F (AcrB-Ec), an amino acid located in the hydrophobic pit of the DBP, was found in multiple studies. Other mutations found in clinically, environmentally and laboratory-evolved strains can be found in Table 9. We note that there are likely more mutations and studies regarding mutations in RND multidrug efflux pumps not mentioned in this review.

Additionally, it is possible that the mentioned gain-of-function mutations cause increased MDR, rather than an increased resistance to one class of antibiotics, as many papers reviewed in this review article did not test for multiple classes of drugs but found mutations in the pumps after observing specific resistance in clinical strains (e.g., for carbapenems, fluoroquinolones, macrolides or aminoglycosides). Examples where MICs increased for multiple drugs as a result of a specific mutation are: AcrB-Sa (G288D), increasing the MICs for chloramphenicol, ciprofloxacin (fluoroquinolone) and tetracycline [80]; AcrB-Ec (G288C), increasing the MICs in an F610A background for erythromycin (macrolide), novobiocin, minocycline, nalidixic acid (quinolone) and SDS [79]; MtrD-Ng (R714G), increasing the MICs for azithromycin, erythromycin (macrolides), ethidium and polymyxin B [43]; and MtrD-Ng (K823E), increasing the MICs for azithromycin, erythromycin (macrolides) and polymyxin B [43]. Additionally, the “G288” mutation emerges in different pumps from different organisms, of which strains are resistant to a variety of drugs (including macrolides, fluoroquinolones, aminoglycosides and carbapenems), pointing to an increase in MDR by one gain-of-function mutation. Additionally, a combination of the mutations mentioned in this review may potentially increase MDR, which may result from increased use of alternative antibiotic treatments.

These recent adaptive mutations are worrying, as commonly used antibiotics to treat infections caused by these pathogens are rendered ineffective, and last-resort antibiotics are used (which have more or worse side effects or may not always be an option in underdeveloped regions in the world). An example is the use of colistin for *A. baumannii* infections resistant to carbapenems. Especially worrying are cases where extensively drug-resistant (XDR) pathogens leave a specific class of antibiotics as a last option, after which this XDR pathogen becomes resistant to this antibiotic too, by mutations in the RND pump, noticeably azithromycin resistance in *S.* Typhi strains in India, Nepal, Bangladesh and Pakistan. It is particularly worrying that these mutations—besides being spread through transfer—seem to be appearing independently in different locations, and in different organisms and pumps, further indicating that the misusage and over-usage of antibiotics put extreme selective pressure on these pathogens, giving rise to not only mutations in genes part of expression regulatory pathways but also gain-of-function mutations in the efflux pumps themselves, leaving us with last-resort antibiotics, or worse, when a pump increases (or potentially gains) resistance to the last treatment options.

We hope that this review can help increase our understanding of the mechanisms of drug recognition by RND multidrug efflux pumps and help the development of novel antibiotics and efflux pump inhibitors needed to treat the increasingly spreading and evolving pathogenic bacteria.

## Figures and Tables

**Table 1:** Conservation and similarity between 135 MDR RND-type efflux pumps.

Domain/Subdomain/Loop		Sequence (Based on AcrB-Ec)	Residue Count	Conserved or Highly (%)
**Transmembrane Domain**	TM1	**F**ID**RP**IF**A**WVIAIIIMLAG**G**LAILK**L**	26	6/26 (23.1%)
	TM2	**T**PFVKI**SI**HEVVKTLV**EA**IIL**V**FL**V**MYLF**L**	30	8/30 (26.7%)
	TM3	RATL**IP**TIAV**PV**V**L**L**G**TFAV**L**AA	23	7/23 (30.4%)
	TM4	**N**T**L**T**M**FGM**VL**A**IGLLVDDAIVVVEN**VE**R**V**M**AEE	33	21/33 (63.6%)
	TM5	**P**KE**A**TRKSMGQ**I**QGA**LV**GIAMV**L**S**AV**FV**P**MAFF	33	9/33 (27.3%)
	TM6	**G**AI**Y**RQ**F**SIT**I**VS**A**MAL**S**VLV**AL**I**L**T**P**ALCATM**L**	34	11/34 (32.4%)
	TM7	GRYLVLYLIIVVGMAYLFVRL	21	0/21 (0%)
	TM8	QAPSLYAISLIVV**FL**C**L**AAL**Y**	21	4/21 (19.0%)
	TM9	I**P**FS**V**MLVV**P**LGVIGALLAATFR	23	3/23 (13.0%)
	TM10	D**V**YFQ**V**GLLTTI**GL**SA**K**NA**ILI**V**E**F**A**KDLMDK	32	10/32 (31.3%)
	TM11	GLIE**A**TLDAVRM**RLRPI**L**MTS**L**A**FIL**G**VM**PL**VI	33	13/33 (39.4%)
	TM12	SGAQNAV**G**TG**V**MG**G**MVTA**T**VLAIFFV**P**VFFVVVRRR	36	5/36 (13.9%)
	lα	GFFGWFNRMFEKSTHH**Y**TDSVGGILRS	27	1/27 (3.7%)
**Periplasmic Domain**	PN1	PPAVT**I**SASYPG**A**DAKTVQDT**V**TQV**I**EQNMNGIDNLMYMS**S**NSDSTGTVQI TLTFESGTDADIAQVQVQNKLQLAMPLL**P**QEVQQQG**V**SVEK/PRLERYNG	100	7/100 (7%)
	PN2	VVGVINTDGTMTQEDISDYVAAN**M**KDAISRTS**G**VGDVQLFGSQ/GENYDII AEFNGQPASGLGIKLATGA**N**ALDTAAAIRAELAKMEPFF**P**SGLKIVYPYDT	101	4/101 (4.0%)
	PC1	GVFMTMVQLPAGATQERTQKVLNEVTHYYLTKEKNNVESVFAVNGFGFA**G** RGQNTGIAFVSLKDWAD**R**PGEENKVEAITMRATRAFSQIKDAMVFAF	97	2/97 (2.1%)
	PC2	**G**FDFELIDQAGLGHEKLTQARNQLLAEAAKHPDMLTSVRPNGLED/SPRLE RYNGLP**S**MEILGQAAPGK**S**TGE**A**MELMEQLASKLPTGVGYDWTGMSY**Q**	98	5/98 (5.1%)
	DN	YA**M**RI**W**MNPNELNKFQLTPVD**V**ITA**I**KAQNAQVAA**G**QLGGTPPVKGQQLN ASIIAQTRLTSTEEFGKIL**L**KVNQDGSRVL**L**RD**V**AKIELG	90	8/90 (8.9%)
	DC	TPQFKIDIDQEKAQAL**G**VSINDINTTLGAAWGGSYVNDFIDRGRVKK**V**YVMS EAKYRMLPDDIGDWYVRAAD**G**QMVPFSAFSSSRWEYG	89	3/89 (3.4%)
**Loops**	Switch-loop	GFGFA**G**R	7	1/7 (14.3%)
	Distal-loop	EKSSSSFLM	9	0/9 (0%)
	F + Bottom-loop	PAIVELGTAT	10	0/10 (0%)
	Hoisting-loop	ERLSGNQ	7	0/7 (0%)
	Interface-loop	PSSFL**P**D**ED**QG	11	3/11 (27.3%)

Bold underlined, fully conserved between 135 pumps; bold, conserved in 19 selected pumps and highly conserved in all 135. Abbreviations: TM, transmembrane helix; F, flexible.

**Table 2:** Conserved residues based on 135 RND-type efflux pumps.

Domain		135 Conserved	19 Conserved + 135 Highly	19 Conserved	135 Highly Conserved
Transmembrane		P9, G23, P373, N391, L400, V406, D407, I410, E414, V452, P455, F470, S481, P490, L888, I943, L944, R971, R973, M977, T978, P988, G1010, P1023	A347, V351, I367, S389, D408 *, A409, V412, A430, L449, L937, K940 **, A963, A981	F5, Y327 ***, E346, P368, G378, I402, G403, N415, R418, P427, G461, G464, L497, Y527, P898, P906, G936, E947, A949, P974, G985, A995, G1004, T1015, P1023	R8, A12, L30, T330, S336, I337, V354, L359, V374, L376, L383, L393, M395, V399, L404, L405, V411, V413, M420, I438, L442, V443, A451, Y467, I474, A477, A485, L486, L488, F885, L886, Y892, V901, V925, V929, I945, L972, I975, S979, L989, V1007
Periplasmic	Porter	P36, A52, G171, N298, P318, D568, R637	-	P119, P565, G619, G679, S836, A840	I45, V61, I65, S80, V127, M162, E567, S824, Q865
	Head	G217	-	G740, G796	M184, W187 ****, V203, I207, L251, L262, V265, V771

Analysis after Clustal Omega alignment, based on the categories described in this article (red, light red, orange and yellow). Numbering in accordance with AcrB-Ec. * Exception: D→N in *Marinobacter hydrocarbonoclasticus* and *Alkalilimnicola ehrlichii*; ** exception: K→R in *Idiomarina* *loihiensis*, *Cellvibrio* *japonicus* and *Teredinibacter* *turnerae*; *** exception: 25/135 pumps (mostly Y→F), including Y→I in LpeB-Lp from *L. pneumophila* (the only exception among the 19 selected pumps); **** exception: W→F in “MexI/W” and W→T in LpeB-Lp from *L. pneumophila*. The heat maps of these residues can be seen in Figure 3b–e, Figures S1 and S2 (red to yellow). Underlined, mentioned explicitly in the article text. A green background highlights the conserved residues in TM4, and the blue background highlights the conserved residues in TM11.

**Table 3:** Additional highly conserved residues.

>90%	80 ≤ 90%	70 ≤ 80%	50 ≤ 80%
G51(NS), Y77(FST), G86(NS), L118(MP), P119(G), G179(AS), R185(N), N211(RS) P223(G), F246(LVY), D264(HNQS), A266(G), V340(GIT), T343(ASV), P358(CL), R363(KN), P368(ATN), G387(DN), E602(Q), Q774(IMRS), R780(DL), A890(GISTV), E893(GN), F948(VY), G994(DS)	I6(TV), F94(AILM), S144(ADFN), V172(AIMT), A279(GST), T394(NS), A401(CSV), V416(ACIM), A457(GSV), T473(ASV), N820(ADLMQS), A889(CSV), V905(AIL), G911(AS), N941(HST), V946(ACFIT), G996(AS)	N109(ADS), V122(AIST), D156(EHNS), R168(AG KQST), Q210(EHNSY), A299(ELPST), L350(ACIV), Q360(AGHR), F396(ALV), M435(ACLSTV), V448(AIT), F453(LY), L492(IMQ), Q469(ES), Q928(DEKLMV), T933(ALMV), A942(G), F982(LMT)	A16(NST), M355(ITV), T365(AIMSV), V372(AI), T431(ASV), S434(AGST), I445(MSTY), I446(FLM(V)), S471(AT(CG)), S375(ACSV(T)), M478(IMTV(A)), T489(S(K)), V884(AI), L891(Q(M)), D924(N(S)), R1000(Q(KL))

Residues were chosen from the relatively highest conserved category after analysis by ConSurf [67,68], and conservation ranges between 50% and 100% (Table S1), excluding the residues mentioned already in Table 2. Residues in brackets (AA) indicate alternative residues among the 135 pumps. Double brackets at “50 ≤ 80%” ((AA)) indicate < 1% occurrence. Numbering and amino acid labeling as in AcrB-Ec. The conservation heat map based on these residues can be seen in Figure 3a and Figure S3 (dark purple). Underlined, mentioned explicitly in this article.

**Table 4:** Loop sequences of 19 selected transporters.

Transporters	Flexible-Loop	Distal-Loop	Interface-Loop	Switch-Loop
AcrB-Ec	PAIVELGT	EKSSSSFLM	PSSFL**P**DE**D**QG	GFGFA**G**R
AcrB-Sa	PAIVELGT	EKSSSSFLM	PSSFL**P**DE**D**QG	GFGFA**G**R
AcrF-Sa	PAIVELGT	EKSSSSFLM	PSSFL**P**DE**D**QG	GFSFS**G**Q
AcrF-Ec	PAIVELGT	EKSSSSYLM	PSSFL**P**EE**D**QG	GFSFS**G**Q
MexB-Pa	PSVLELGN	TKAVKNFLM	PEAFV**P**AE**D**LG	GFNFA**G**R
AcrD-Ec	PAISGLGS	RKTGDTNIL	PTSFL**P**LE**D**RG	GSGPG**G**N
AcrD-Sa	PAISGLGS	RKTGDTNIL	PTSFL**P**QE**D**RG	GSGPG**G**N
MexY-Pa	PPLPDLGS	EKAADSIQL	PQAFL**P**EE**D**QG	GFSLY**G**D
MexD-Pa	PPINGLGN	EQTSAGFLL	PEAFV**P**AE**D**LG	GFSFS**G**Q
AdeB-Ab	PAIDELGT	EASSSGFLM	PTAFM**P**EE**D**QG	GWGFS**G**A
AdeJ-Ab	PAMPELGV	TKSGASFLQ	PSSFL**P**EE**D**QG	GFSFT**G**V
MtrD-Ng	PPILELGN	SKARSNFLM	PTSFL**P**TE**D**QG	GFSFS**G**S
MexQ-Pa	PPVPGLGT	QKTSPDILM	PPGFV**P**MQ**D**KY	GLSVN**G**F
MexF-Pa	PPVPGLGT	DKASPDLTM	PTGFV**P**QQ**D**KQ	GLSIN**G**F
AdeG-Ab	PPVMGLGT	LKSSPTLTM	PGGFV**P**AQ**D**KQ	GLSIN**G**F
MexI-Pa	AALPGST–	SSGETTAVA	KRELA**P**TE**D**QA	TWIIN**G**T
LpeB-Lp	PGVDDAG–	QRK–SNGLP	SHETA**P**KE**D**RG	RLTFI**G**D
AcrB-Hi	PEIDTGE–	SSG–GSGIM	SSELT**P**NE**D**KG	GMSIA**G**A
MexW-Pa	PSLPGTG–	EAADASALM	KKELA**P**EE**D**QG	AFQIN**G**Y

Bold underlined, fully conserved. A bar (–) indicates a gap in the sequence alignment.

**Table 5:** Overview of residues of interest in the DBP area of 19 selected RND-type MDR transporters, and conservation among 135 pumps.

	Position	136	178	610	615	617	628	46	89	128	130	134	135	139	151	176	177	180	273	274	276	277	279	288	290	292	327	571	573	612	620
Pump																															
AcrB-Ec		F	F	F	F	F	F	S	Q	S	E	S	S	V	Q	Q	L	S	E	N	D	I	A	G **	G	K	Y	V	M	V	R
Conservation (%)		49	70	50	60	52	74	38	38	18	27	40	32	50	22	59	44	34	55	31	29	10	40	49	43	33	81	29	24	44	19
AcrB-Sa		F	F	F	F	F	F	S	Q	S	E	S	S	V	Q	Q	L	S	E	N	D	V	A	G ***	G	K	Y	V	M	V	R
AcrF-Sa		F	F	F	F	F	F	S	T	S	E	S	S	V	Q	Q	L	A	E	N	N	V	A	G	G	K	Y	V	L	V	Q
AcrF-Ec		Y	F	F	F	F	F	S	T	S	E	S	S	V	Q	Q	L	A	E	N	N	V	A	G	G	K	Y	V	L	V	Q
MexB-Pa		F	F	F	F	F	F	Q	T	R	T	K	N	V	K	Q	V	S	Q	D	S	I	A	G	A	K	Y	V	F	V	R
AcrD-Ec		N	Y	F	S	P	F	T	S	T	R	D	T	T	K	D	A	S	E	K	D	Y	S	G	G	K	Y	M	T	T	N
AcrD-Sa		N	Y	F	S	P	F	T	T	T	R	D	T	T	K	D	A	S	E	K	D	Y	S	G	G	K	Y	M	T	T	N
MexY-Pa		I	W	Y	F	L	F	S	S	Y	E	D	S	I	A	E	T	A	S	E	G	F	S	G ****	A	K	Y	D	M	V	D
MexD-Pa		F	F	F	F	F	F	T	E	Q	E	A	G	I	T	Q	F	S	E	S	N	I	S	G	A	Q	Y	Y	V	I	Q
AdeB-Ab		F	F	T	W	F	F	S	E	Q	E	S	G	L	E	Q	S	A	Q	A	N	F	I	A	A	Q	Y	W	M	I	A
AdeJ-Ab		F *	F	F	F	F	F	A	S	T	T	A	S	V	D	Q	V	G	D	N	Q	F	S	G *	A	K	Y	V	M	V	V
MtrD-Ng		F	F	I	F	F	F	H	S	T	S	S	N	I	T	R	L	A	E	D	S	S	T	G	A	M	Y	F	M	V	S
MexQ-Pa		I	W	V	L	V	F	T	I	V	Q	P	D	V	P	V	V	A	D	A	A	L	S	A	Q	I	Y	Y	V	F	F
MexF-Pa		L	F	V	L	I	F	R	T	T	D	P	D	V	M	Q	L	L	N	Q	A	L	S	A	P	F	Y	Y	V	F	F
AdeG-Ab		L	F	V	L	I	F	R	T	T	L	P	T	V	M	G	L	S	S	Q	G	L	S	A	P	F	Y	Y	I	F	F
MexI-Pa		A	F	–	W	I	G	T	V	E	S	T	T	Y	I	Q	T	G	A	A	E	T	A	H	G	F	Y	A	L	–	T
LpeB-Lp		G	W	–	L	F	V	S	Q	E	Q	S	N	F	F	E	V	–	D	N	Q	M	V	V	S	N	I	L	G	–	D
AcrB-Hi		G	F	–	M	I	I	S	T	S	S	G	S	Y	S	Q	V	A	E	N	N	S	A	V	A	N	Y	A	I	–	A
MexW-Pa		A	L	–	F	I	G	T	T	Q	E	A	S	Y	N	E	I	N	A	S	D	A	S	Y	G	K	Y	I	F	–	Y

Asterisks: * (F→L) and (G→S) found in AdeJ from *Acinetobacter baumannii*—in experimentally evolved and clinical strains from Australia implicated in meropenem resistance [77,78]; ** (G→C) found in experimentally evolved AcrB from *Escherichia coli*—increases efflux, especially for erythromycin [79]; *** (G→D) found in AcrB from *Salmonella* Typhimurium—in experimentally evolved and clinical strains from the UK, implicated in ciprofloxacin resistance [80,81]; **** (G→A/S) found in MexY from *Pseudomonas aeruginosa*—in clinical strains mainly from the EU and Australia, implicated in tobramycin resistance [82,83,84]. Colors: green background, conserved residues compared to AcrB-Ec; red background, bulky Trp (potentially) inhibiting inhibitor (EPI) effectiveness. The first six separated columns show the Phe residues (as in AcrB-Ec) in the “hydrophobic pit”. Note: *Salmonella* AcrB, AcrD and AcrF alignments based on *Salmonella* Typhi str. CT18 genes STY0519 and STY2719 and *Salmonella* Typhi str. LT2 gene STM3391, respectively. LpeB-Lp alignment based on *Legionella pneumophila* str. Paris gene lpp2880. Conservation based on 135 pumps, and further details (regarding alternative residues and the conservation percentages) can be found in Table S1.

**Table 6:** Overview of residues in the PBP area of 19 selected RND-type MDR transporters, and conservation among 135 pumps.

	Position	79	91	569	575	577	624	626	662	664	666	667	668	671	673	674	675	676	681	717	719	824	826	828
Pump																								
AcrB-Ec		S	T	Q	M	Q	T	I	M	F	F	N	L	I	E	L	G	T	D	R ***	N	S	E	L
Conservation (%)		55	70	69	26	61	29	25	16	49	56	31	23	58	32	72	88	37	36	49	43	52	43	
AcrB-Sa		S	T	Q	M	Q	T	I	M	F	F	N	L	I	E	L	G	T	D	R	N	S	E	L
AcrF-Sa		S	T	Q	M	Q	S	M	L	F	F	N	M	I	E	L	G	T	D	R	N	S	E	L
AcrF-Ec		S	T	Q	M	Q	A	M	F	I	F	N	M	I	E	L	G	T	D	R	N	S	E	Q
MexB-Pa		S	T	Q	Q	Q	S	M **	M	F	F	A	P	V	E	L	G	N	D	R	N	A	E	L
AcrD-Ec		S	T	R	S	Q	V	R	R	I	S	S	P	I	G	L	G	S	D	R	N	A	E	V
AcrD-Sa		S	T	R	S	Q	V	R	R	F	S	S	P	I	G	L	G	S	D	R	N	A	E	V
MexY-Pa		K *	T	Q	M	M	S	M	T	Y	M	N	S	L	D	L	G	S	D	M	A	S	N	E
MexD-Pa		E	V	L	D	Q	A	L	T	M	V	S	P	I	G	L	G	N	A	M	E	S	R	V
AdeB-Ab		S	T	Q	S	Q	V	V	E	M	V	L	P	I	E	L	G	T	S	W	E	S	S	A
AdeJ-Ab		S	Q	Q	L	Q	A	I	Y	M	L	Q	L	M	E	L	G	V	N	R	E	S	N	Q
MtrD-Ng		S	S	Q	S	Q	M	M	F	I	V	V	P	I	E	L	G	N	S	R ****	G	S	K ****	S
MexQ-Pa		S	T	K	I	Q	A	V	F	G	F	P	P	V	G	L	G	T	K	M	S	S	D	S
MexF-Pa		S	T	K	F	Q	S	I	Y	A	F	P	P	V	G	L	G	T	R	F	S	T	E	N
AdeG-Ab		Q	T	K	F	Q	A	I	Y	A	F	P	P	V	G	L	G	T	K	F	S	S	D	N
MexI-Pa		S	T	Q	A	K	A	F	S	F	F	Q	L	L	G	S	–	–	P	D	D	A	T	Q
LpeB-Lp		T	T	R	Y	P	–	S	W	W	T	G	L	V	D	A	G	–	E	N	D	S *****	T	H
AcrB-Hi		S	T	K	I	N	S	L	S	S	F	N	I	I	T	G	–	–	P	N	D	S	E	S
MexW-Pa		T	S	Q	M	N	A	I	Q	F	F	N	L	L	G	T	G	–	P	D	D	S	I	S

Asterisks: * (K→T) found in MexY from *Pseudomonas aeruginosa*—experimentally evolved strain, implicated in tobramycin resistance [83]. Additionally, K79A in MexY has increased aminoglycoside (paromomycin) resistance [56]; ** (M→V) found in MexB from *Pseudomonas aeruginosa*—clinical strains from Denmark [5]; *** (R→L/Q) found in AcrB from *Salmonella* Typhi and *Salmonella* Paratyphi A—clinical isolates from Bangladesh, Nepal, India and Pakistan, known to cause azithromycin resistance [85,86,87,88]; **** (R→C/G/H/L) and (K→D/E/N) found in MtrD from *Neisseria gonorrhoeae*—clinical isolates from India, the USA and the EU, known to cause azithromycin resistance [24,43,89]; ***** (S→I) found in LpeB from *Legionella pneumophila*—spring water isolates from China [90]. Colors: green background, conserved residues compared to AcrB-Ec. A bar (–) indicates a gap after sequence alignment for the specific AcrB-Ec position. Note: *Salmonella* AcrB, AcrD and AcrF alignments based on *Salmonella* Typhi str. CT18 genes STY0519 and STY2719 and *Salmonella* Typhi str. LT2 gene STM3391, respectively. LpeB-Lp alignment based on *Legionella pneumophila* str. Paris gene lpp2880. Conservation based on 135 pumps, and further details (regarding alternative residues and the conservation percentages) can be found in Table S1.

**Table 7:** Charged and hydrophobic residues in the binding pockets.

	Proximal Binding Pocket (PBP)					Distal Binding Pocket (DBP)				
Transporters	+	-	Sum	HP	Hydrophilicity (kcal mol^−1^)	+	-	Sum	HP	Hydrophilicity (kcal mol^−1^)
AcrD-Ec	4	2	6	5	27.07	4	4	8	3	39.56
AcrD-Sa	4	2	6	5	27.35	4	4	8	3	39.24
MexY-Pa	1	3	4	7	23.16	1	6	7	8	26.97
MexB-Pa	1	3	4	7	25.75	5	1	6	12	26.19
AcrB-Sa	1	3	4	9	20.54	2	3	5	12	25.93
LpeB-Lp	1	3	4	2	24.35	0	4	4	11	25.87
AcrB-Ec	1	3	4	9	20.54	2	3	5	12	25.64
MexW-Pa	0	2	2	7	20.14	1	3	4	6	23.23
MexD-Pa	1	3	4	9	18.59	0	3	3	11	22.80
AcrF-Ec	1	3	4	8	23.49	1	2	3	11	22.25
AdeB-Ab	0	3	3	7	18.65	0	3	3	9	21.69
AcrF-Sa	1	3	4	9	21.36	1	2	3	12	21.25
AdeJ-Ab	1	2	3	8	23.74	1	2	3	13	18.62
MexF-Pa	2	1	3	6	20.77	1	2	3	15	18.27
AcrB-Hi	1	2	3	5	21.41	0	1	1	7	17.30
MtrD-Ng	2	1	3	8	20.60	1	2	3	12	16.92
MexI-Pa	1	2	3	5	21.57	0	2	2	6	15.47
MexQ-Pa	2	1	3	7	19.95	0	2	2	15	13.59
AdeG-Ab	2	1	3	6	22.82	1	0	1	15	10.16

Colors: green, positive contribution to aminoglycoside recognition; orange, negative contribution or difference explaining aminoglycoside non-recognition; yellow, AcrB-Hi’s low charged and hydrophobic residue count (and hydrophilicity, orange), possibly explaining lower substrate export efficiency compared to AcrB-Ec (including the significantly low bile salt MICs). Hydrophobicity based on [93]. Abbreviations and symbols: number of positively charged residues (+), negatively charged residues (-) and hydrophobic residues (HP). Definitions: positively charged, K and R; negatively charged, D and E; hydrophobic, I, L, F, V, C and M residues.

**Table 8:** Substrate specificities of six different pumps.

Pump	EM	NOV	Tet	Qui	NSP	CAR	SUB	Bile	AG	AZT
AcrB-Ec	✓	✓	✓	✓	✓	✓	✓	✓	X	X
AcrD-Ec	X	✓	X	X	✓	✓	✓	✓	✓	✓
MexB-Pa	✓	✓	✓	✓	✓	✓	✓	n/a	X	✓
MexD-Pa	✓	✓	✓	✓	✓	X	X	n/a	X	X
MexY-Pa	✓	X	✓	✓	✓	X	X	n/a	✓	X
AcrB-Hi	✓	✓	✓	✓	✓	n/a	n/a	✓ *	X	X

Comparison between AcrB-Ec, AcrD-Ec, MexB-Pa, MexD-Pa, MexY-Pa and AcrB-Hi for several antibiotics or classes of antibiotics. A check mark (✓) indicates recognition of the substrate by the pump, and a cross (X) indicates no recognition. Green highlights a substrate for all six pumps. Asterisk (*) indicates just slightly exported. Abbreviations: EM, erythromycin; NOV, novobiocin; Tet, tetracyclines; Qui, quinolones (e.g., ciprofloxacin, norfloxacin, enoxacin (fluoroquinolones), nalidixic acid (quinolone)); NSP, (second-generation) narrow-spectrum penicillin β-lactams (e.g., cloxacillin, oxacillin and nafcillin); CAR, carbenicillin; SUB, sulbenicillin; Bile, bile salts (cholic acid, deoxycholic acid); AG, aminoglycosides; AZT, aztreonam (monobactam); n/a, not available. Overview created from references [15,35,56,97,99,101,102,105].

**Table 9:** RND mutations in recent clinically, environmentally and experimentally evolved strains.

Organism	Pump	Mutations	Country	Resistance	References
*Salmonella enterica*	AcrB	G288D *	UK	Ciprofloxacin (fluoroquinolone)	[80,81]
		P319L *	China	Multiple (fluoroquinolones)	[112]
		P319L/M78I *	China	Multiple (fluoroquinolones)	[112]
		R717Q *	Bangladesh, Pakistan, India	Azithromycin (macrolide)	[85,86,87,88]
		R717L *	Bangladesh, Nepal	Azithromycin (macrolide)	[85,88,111]
*Salmonella* Paratyphi *A*	AcrB	R717L *	Bangladesh	Azithromycin (macrolide)	[85]
		R717Q *	Bangladesh	Azithromycin (macrolide)	[88]
*Neisseria gonorrhoeae*	MtrD	R714H *	Europe, Russia	Azithromycin (macrolide)	[89]
		R714L *	USA	Azithromycin (macrolide)	[89]
		R714C *	USA	Azithromycin (macrolide)	[89]
		R714G *	Experimentally	Azithromycin (macrolide)	[43]
		K823N *	Canada	Azithromycin (macrolide)	[89]
		K823E *	USA, India, Canada	Azithromycin (macrolide)	[24,89,113]
		K823E/S821A *	USA	Azithromycin (macrolide)	[24]
		K823D *	USA	(N/D)	[24]
*Pseudomonas* *aeruginosa*	MexB	A562V	Denmark	(N/D)	[5]
		M626V	Denmark	(N/D)	[5]
		F178S	Australia	(N/D)	[82]
	MexY	K79T *	Experimentally	Tobramycin (aminoglycoside)	[83]
		K79A *	Experimentally	Paromomycin (aminoglycoside)	[56]
		**G287A**	Australia, Spain	High tobramycin MIC isolates	[82]
		**G287S**	Europe (and other), *Experimentally*	Tobramycin (aminoglycoside)	[83,84]
*Escherichia coli*	AcrB	V139F	Experimentally	(N/D)	[114,115,116,117]
		**G288S/M** /C *	Experimentally	(N/D)	[118]
		**G288C** *	Experimentally (frequent mutation)	Multiple (especially erythromycin)	[79]
		A279T	Experimentally	(N/D)	[118]
		A279T, F617L	Experimentally (frequent mutation)	(Increased 1-Hexene tolerance)	[119]
*Acinetobacter baumannii*	AdeJ	F136L *	Australia	Meropenem (carbapenem)	[77,78]
		G288S *	Australia	Meropenem (carbapenem)	[77,78]
*Legionella pneumophila*	LpeB	S824I (I911L, G1158W, F1124 insert)	China	Azithromycin (macrolide)	[90]

Bold underlined indicates the recurring “**G288**” mutation in AcrB-Ec, AcrB-Sa (*Salmonella* Typhimurium) [80,81], MexY-Pa and AdeJ-Ab. Bold indicates the “**R717**” mutation recurring in AcrD-Sa (*Salmonella* Typhi and Paratyphi A) [85,86,87,88,111] and MtrD-Ng [43,89]. Asterisk (*) indicates direct measured increased MICs.

## Data Availability

Data is contained within the article or Supplementary Materials.

## Supplementary Materials

The following are available online at https://www.mdpi.com/article/10.3390/antibiotics10070774/s1, Figure S1: Additional images of the TM domain, Figure S2: Additional images of the porter domain, Figure S3: Additional images of the ConSurf output, Figure S4: Front view of the drug-bound structures of AcrB-Ec, MtrD-Ng and AdeB-Ab, Table S1: Overview of residue conservation based on 135 MDR-type RND pumps, Data S1: Sequences of 135 MDR-type RND pumps.
