# Supplementary material for: Ever-Adapting RND Efflux Pumps in Gram-Negative Multidrug-Resistant Pathogens: A Race against Time
Source: Antibiotics (Basel). 2021 Jun 25;10(7):774. doi: 10.3390/antibiotics10070774 (PMC8300642; doi:10.3390/antibiotics10070774)
Supplement: Supplementary file 1 [file antibiotics-10-00774-s001.zip › Data S1.pdf]

Data S1: Sequences of 135 MDR-type RND pumps.

>AcrB_E_coli
MPNFFIDRPIFAWVIAIIIMLAGGLAILKLPVAQYPTIAPPAVTISASYPGADAKTVQDTVTQVIEQNMNGIDNLMYMSSNS
DSTGTVQITLTFESGTDADIAQVQVQNKLQLAMPLLPQEVQQQGVSVEKSSSSFLMVVGVINTDGTMTQEDISDYVAA
NMKDAISRTSGVGDVQLFGSQYAMRIWMNPNELNKFQLTPVDVITAIKAQNAQVAAGQLGGTPPVKGQQLNASIIAQ
TRLTSTEEFGKILLKVNQDGSRVLLRDVAKIELGGENYDIIAEFNGQPASGLGIKLATGANALDTAAAIRAELAKMEPFFPSG
LKIVYPYDTTPFVKISIHEVVKTLVEAIILVFLVMYLFLQNFRATLIPTIAVPVVLLGTFAVLAAFGFSINTLTMFGMVLAIGLLVD
DAIVVVENVERVMAEEGLPPKEATRKSMGQIQGALVGIAMVLSAVFVPMAFFGGSTGAIYRQFSITIVSAMALSVLVALILT
PALCATMLKPIAKGDHGEGKKGFFGWFNRMFEKSTHHYTDSVGGILRSTGRYLVLYLIIVVGMAYLFVRLPSSFLPDEDQG
VFMTMVQLPAGATQERTQKVLNEVTHYYLTKEKNNVESVFAVNGFGFAGRGQNTGIAFVSLKDWADRPGEENKVEAIT
MRATRAFSQIKDAMVFAFNLPAIVELGTATGFDFELIDQAGLGHEKLTQARNQLLAEAAKHPDMLTSVRPNGLEDTPQFK
IDIDQEKAQALGVSINDINTTLGAAWGGSYVNDFIDRGRVKKVYVMSEAKYRMLPDDIGDWYVRAADGQMVPFSAFSS
SRWEYGSPRLERYNGLPSMEILGQAAPGKSTGEAMELMEQLASKLPTGVGYDWTGMSYQERLSGNQAPSLYAISLIVVFL
CLAALYESWSIPFSVMLVVPLGVIGALLAATFRGLTNDVYFQVGLLTTIGLSAKNAILIVEFAKDLMDKEGKGLIEATLDAVR
MRLRPILMTSLAFILGVMPLVISTGAGSGAQNAVGTGVMGGMVTATVLAIFFVPVFFVVVRRRFSRKNEDIEHSHTVDHH

>AcrF_E_coli
MANFFIRRPIFAWVLAIILMMAGALAILQLPVAQYPTIAPPAVSVSANYPGADAQTVQDTVTQVIEQNMNGIDNLMYMS
STSDSAGSVTITLTFQSGTDPDIAQVQVQNKLQLATPLLPQEVQQQGISVEKSSSSYLMVAGFVSDNPGTTQDDISDYVA
SNVKDTLSRLNGVGDVQLFGAQYAMRIWLDADLLNKYKLTPVDVINQLKVQNDQIAAGQLGGTPALPGQQLNASIIAQ
TRFKNPEEFGKVTLRVNSDGSVVRLKDVARVELGGENYNVIARINGKPAAGLGIKLATGANALDTAKAIKAKLAELQPFFP
QGMKVLYPYDTTPFVQLSIHEVVKTLFEAIMLVFLVMYLFLQNMRATLIPTIAVPVVLLGTFAILAAFGYSINTLTMFGMVLA
IGLLVDDAIVVVENVERVMMEDKLPPKEATEKSMSQIQGALVGIAMVLSAVFIPMAFFGGSTGAIYRQFSITIVSAMALSVL
VALILTPALCATLLKPVSAEHHENKGGFFGWFNTTFDHSVNHYTNSVGKILGSTGRYLLIYALIVAGMVVLFLRLPSSFLPEE
DQGVFLTMIQLPAGATQERTQKVLDQVTDYYLKNEKANVESVFTVNGFSFSGQAQNAGMAFVSLKPWEERNGDENSA
EAVIHRAKMELGKIRDGFVIPFNMPAIVELGTATGFDFELIDQAGLGHDALTQARNQLLGMAAQHPASLVSVRPNGLEDT
AQFKLEVDQEKAQALGVSLSDINQTISTALGGTYVNDFIDRGRVKKLYVQADAKFRMLPEDVDKLYVRSANGEMVPFSAF
TTSHWVYGSPRLERYNGLPSMEIQGEAAPGTSSGDAMALMENLASKLPAGIGYDWTGMSYQERLSGNQAPALVAISFV
VVFLCLAALYESWSIPVSVMLVVPLGIVGVLLAATLFNQKNDVYFMVGLLTTIGLSAKNAILIVEFAKDLMEKEGKGVVEAT
LMAVRMRLRPILMTSLAFILGVLPLAISNGAGSGAQNAVGIGVMGGMVSATLLAIFFVPVFFVVIRRCFKG

>AcrD_E_coli
MANFFIDRPIFAWVLAILLCLTGTLAIFSLPVEQYPDLAPPNVRVTANYPGASAQTLENTVTQVIEQNMTGLDNLMYMSS
QSSGTGQASVTLSFKAGTDPDEAVQQVQNQLQSAMRKLPQAVQNQGVTVRKTGDTNILTIAFVSTDGSMDKQDIADY
VASNIQDPLSRVNGVGDIDAYGSQYSMRIWLDPAKLNSFQMTAKDVTDAIESQNAQIAVGQLGGTPSVDKQALNATIN
AQSLLQTPEQFRDITLRVNQDGSEVRLGDVATVEMGAEKYDYLSRFNGKPASGLGVKLASGANEMATAELVLNRLDELA
QYFPHGLEYKVAYETTSFVKASIEDVVKTLLEAIALVFLVMYLFLQNFRATLIPTIAVPVVLMGTFSVLYAFGYSVNTLTMFA
MVLAIGLLVDDAIVVVENVERIMSEEGLTPREATRKSMGQIQGALVGIAMVLSAVFVPMAFFGGTTGAIYRQFSITIVAAM
VLSVLVAMILTPALCATLLKPLKKGEHHGQKGFFAWFNQMFNRNAERYEKGVAKILHRSLRWIVIYVLLLGGMVFLFLRLP
TSFLPLEDRGMFTTSVQLPSGSTQQQTLKVVEQIEKYYFTHEKDNIMSVFATVGSGPGGNGQNVARMFIRLKDWSERDS
KTGTSFAIIERATKAFNQIKEARVIASSPPAISGLGSSAGFDMELQDHAGAGHDALMAARNQLLALAAENPELTRVRHNG
LDDSPQLQIDIDQRKAQALGVAIDDINDTLQTAWGSSYVNDFMDRGRVKKVYVQAAAPYRMLPDDINLWYVRNKDGG
MVPFSAFATSRWETGSPRLERYNGYSAVEIVGEAAPGVSTGTAMDIMESLVKQLPNGFGLEWTAMSYQERLSGAQAPAL
YAISLLVVFLCLAALYESWSVPFSVMLVVPLGVIGALLATWMRGLENDVYFQVGLLTVIGLSAKNAILIVEFANEMNQKGH
DLFEATLHACRQRLRPILMTSLAFIFGVLPMATSTGAGSGGQHAVGTGVMGGMISATILAIYFVPLFFVLVRRRFPLKPRPE

>MexB_P_aeruginosa
MSKFFIDRPIFAWVIALVIMLAGGLSILSLPVNQYPAIAPPAIAVQVSYPGASAETVQDTVVQVIEQQMNGIDNLRYISSES
NSDGSMTITVTFEQGTDPDIAQVQVQNKLQLATPLLPQEVQRQGIRVTKAVKNFLMVVGVVSTDGSMTKEDLSNYIVSN
IQDPLSRTKGVGDFQVFGSQYSMRIWLDPAKLNSYQLTPGDVSSAIQAQNVQISSGQLGGLPAVKGQQLNATIIGKTRL
QTAEQFENILLKVNPDGSQVRLKDVADVGLGGQDYSINAQFNGSPASGIAIKLATGANALDTAKAIRQTIANLEPFMPQG
MKVVYPYDTTPVVSASIHEVVKTLGEAILLVFLVMYLFLQNFRATLIPTIAVPVVLLGTFGVLAAFGFSINTLTMFGMVLAIGL
LVDDAIVVVENVERVMAEEGLSPREAARKSMGQIQGALVGIAMVLSAVFLPMAFFGGSTGVIYRQFSITIVSAMALSVIVA
LILTPALCATMLKPIEKGDHGEHKGGFFGWFNRMFLSTTHGYERGVASILKHRAPYLLIYVVIVAGMIWMFTRIPTAFLPDE
DQGVLFAQVQTPPGSSAERTQVVVDSMREYLLEKESSSVSSVFTVTGFNFAGRGQSSGMAFIMLKPWEERPGGENSVFE
LAKRAQMHFFSFKDAMVFAFAPPSVLELGNATGFDLFLQDQAGVGHEVLLQARNKFLMLAAQNPALQRVRPNGMSDE
PQYKLEIDDEKASALGVSLADINSTVSIAWGSSYVNDFIDRGRVKRVYLQGRPDARMNPDDLSKWYVRNDKGEMVPFN
AFATGKWEYGSPKLERYNGVPAMEILGEPAPGLSSGDAMAAVEEIVKQLPKGVGYSWTGLSYEERLSGSQAPALYALSLL
VVFLCLAALYESWSIPFSVMLVVPLGVIGALLATSMRGLSNDVFFQVGLLTTIGLSAKNAILIVEFAKELHEQGKGIVEAAIEA
CRMRLRPIVMTSLAFILGVVPLAISTGAGSGSQHAIGTGVIGGMVTATVLAIFWVPLFYVAVSTLFKDEASKQQASVEKGQ

>MexY_P_aeruginosa
MARFFIDRPVFAWVISLLIVLAGVLAIRFLPVAQYPDIAPPVVNVSASYPGASAKVVEEAVTAIIEREMNGAPGLLYTKATSS
TGQASLTLTFRQGVNADLAAVEVQNRLKIVESRLPESVRRDGIYVEKAADSIQLIVTLTSSSGRYDAMELGEIASSNVLQAL
RRVEGVGKVETWGAEYAMRIWPDPAKLTSMNLSASDLVNAVRRHNARLTVGDIGNLGVPDSAPISATVKVDDTLVTPE
QFGEIPLRIRADGGAIRLRDVARVEFGQSEYGFVSRVNQMTATGLAVKMAPGSNAVATAKRIRATLDELSRYFPEGVSYNI
PYDTSAFVEISIRKVVSTLLEAMLLVFAVMYLFMQNFRATLIPTLVVPVALLGTFTVMLGLGFSINVLTMFGMVLAIGILVDD
AIIVVENVERLMAEEGLSPHDATVKAMRQISGAIVGITVVLVSVFVPMAFFSGAVGNIYRQFAVTLAVSIGFSAFLALSLTPA
LCATLLRPIDADHHEKRGFFGWFNRAFLRLTGRYRNAVAGILARPIRWMLVYTLVIGVVALLFVRLPQAFLPEEDQGDFMI
MVMQPEGTPMAETMANVGDVERYLAEHEPVAYAYAVGGFSLYGDGTSSAMIFATLKDWSERREASQHVGAIVERINQ
RFAGLPNRTVYAMNSPPLPDLGSTSGFDFRLQDRGGVGYEALVKARDQLLARAAEDPRLANVMFAGQGEAPQIRLDIDR
RKAETLGVSMDEINTTLAVMFGSDYIGDFMHGSQVRKVVVQADGAKRLGIDDIGRLHVRNEQGEMVPLATFAKAAWTL
GPPQLTRYNGYPSFNLEGQAAPGYSSGEAMQAMEQLMQGLPEGIAHEWSGQSFEERLSGAQAPALFALSVLIVFLALAA
LYESWSIPLAVILVVPLGVLGALLGVSLRGLPNDIYFKVGLITIIGLSAKNAILIIEVAKDHYQEGMSLLQATLEAARLRLRPIVM
TSLAFGFGVVPLALSSGAGSGAQVAIGTGVLGGIVTATVLAVFLVPLFFLVVGRLFRLRKAPRTGNSPQIPTEQA

>MexD_P_aeruginosa
MSEFFIKRPNFAWVVALFISLAGLLVISKLPVAQYPNVAPPQITITATYPGASAKVLVDSVTSVLEESLNGAKGLLYFESTNNS
NGTAEIVVTFEPGTDPDLAQVDVQNRLKKAEARMPQAVLTQGLQVEQTSAGFLLIYALSYKEGAQRSDTTALGDYAARN
INNELRRLPGVGKLQFFSSEAAMRVWIDPQKLVGFGLSIDDVSNAIRGQNVQVPAGAFGSAPGSSAQELTATLAVKGTLD
DPQEFGQVVLRANEDGSLVRLADVARLELGKESYNISSRLNGTPTVGGAIQLSPGANAIQTATLVKQRLAELSAFFPEDM
QYSVPYDTSRFVDVAIEKVIHTLIEAMVLVFLVMFLFLQNVRYTLIPSIVVPVCLLGTLMVMYLLGFSVNMMTMFGMVLAI
GILVDDAIVVVENVERIMAEEGISPAEATVKAMKQVSGAIVGITLVLSAVFLPLAFMAGSVGVIYQQFSVSLAVSILFSGFLA
LTFTPALCATLLKPIPEGHHEKRGFFGAFNRGFARVTERYSLLNSKLVARAGRFMLVYAGLVAMLGYFYLRLPEAFVPAEDL
GYMVVDVQLPPGASRVRTDATGEELERFLKSREAVASVFLISGFSFSGQGDNAALAFPTFKDWSERGAEQSAAAEIAALN
EHFALPDDGTVMAVSPPPINGLGNSGGFALRLMDRSGVGREALLQARDTLLGEIQTNPKFLYAMMEGLAEAPQLRLLIDR
EKARALGVSFETISGTLSAAFGSEVINDFTNAGRQQRVVIQAEQGNRMTPESVLELYVPNAAGNLVPLSAFVSVKWEEGP
VQLVRYNGYPSIRIVGDAAPGFSTGEAMAEMERLASQLPAGIGYEWTGLSYQEKVSAGQATSLFALAILVVFLLLVALYES
WSIPLSVMLIVPIGAIGAVLAVMVSGMSNDVYFKVGLITIIGLSAKNAILIVEFAKELWEQGHSLRDAAIEAARLRFRPIIMTS
MAFILGVIPLALASGAGAASQRAIGTGVIGGMLSATFLGVLFVPICFVWLLSLLRSKPAPIEQAASAGE

>MexQ_P_aeruginosa

MTFPRFFIDRPIFAIVLSVLMMIGGIVSFFQLPLSEYPAVTPPTVQVTTAYPGANPDVIAQTVATPLEQAITGVEGMLYMSS
QSATDGRMILTITFDQHIDPDMAQIQVQNRVSRVLSRLPDEVQRQGVVTQKTSPDILMVVHLLSPEQRYDPLYISNYAYL
QVRDELLRLPGISDVVVWGAGEYSMRLWLDPDLIAARGLTAGEVIAAVREQNVQVAAGAVGQAPDSTAAFQVTVNTL
GRLSDEEQFGDIIVRTGADGQVTRLRDVARIEMGGDAYALRSLLDGEPAVALQIIQSPGANALDTAEAVRATVARLEGNF
PAGLSARIAYDPTVFVRASLQTVATTLLEAILLVVVVVVLFLRSWRASLIPLMAVPVSLVGTFAVMHLMGFSLNTLSLFGLVL
SIGIVVDDAIVVVENVERHIENGEPPLQAARRAMDEVTGPIMAITSVLAAVFIPTAFLSGLQGEFYRQFALTIAISTILSALNSL
TLSPALAGLLLRPRPAGGAVAGRFQRLLQVLGRPLRNAPEAYGNAVRKVVRVSGLALVVYGGLLGLTWVGFQAVPPGFV
PMQDKYYLVGIAQLPNGASLERTDAVVRQMSRIGLDEPGVESVVAFPGLSVNGFVNVPNAAVMFFMLDPFESRTSADL
GALAIAGRLQARFASIPDGFLGVFPPPPVPGLGTIGGFKMQVEDRGGAGLEALARQTQVLMMKATESGQLGGLMTSFDI
NAPQLEVVVDRTKVKSQGVRLADVFEALQVYLGSLYINDFNRFGRTYKVTAQADAPHRMQAEAIGRLQVRNAAGAMLP
LSSFVTVTPSSGPDRVIHYNGYPSADISGGALPGVSSGQAVALMERLAGEVLPEGMTFEWTDLTYQQKLAGNSALFIFPLC
VLLAYLILAAQYNSWLLPLAVLLIVPMCLLSAIAGVWLVGGDNNVFVQIGLVVLVGLAAKNAILIVEFARTLEAEGARALEA
VVEACRLRLRPILMTSLAFIAGVVPLVMASGAGAEMRQAMGVAVFAGMLGVTLFGLFLTPVFYVLVRALAARLERRAGGS
LAHLEGGRP

>MexF_P_aeruginosa
MNFSQFFIQRPIFAAVLSLLILIGGAISLFQLPISEYPEVVPPTVVVRANFPGANPKVIGETVASPLEQAITGVENMLYMSSQS
TSDGKLTLTITFALGTDLDNAQVQVQNRVTRTEPKLPEEVTRLGITVDKASPDLTMVVHLTSPDNRYDMLYLSNYAVLNV
KDELARLDGVGDVQLFGLGDYSLRVWLDPNKVASRNLTATDVVNAIREQNRQVAAGTLGAPPAPSDTSFQLSINTQGR
LVTEEEFENIIIRAGANGEITRLRDIARVELGSNQYALRSLLNNKPAVAIPIFQRPGSNAIEISNLVREKMAELKHSFPQGMDY
SIVYDPTIFVRGSIEAVVHTLFEALVLVVLVVILFLQTWRASIIPLAAVPVSLIGTFAVMHMLGFSLNALSLFGLVLAIGIVVDD
AIVVVENVERNIGLGLKPVEATKRAMREVTGPIIATALVLCAVFIPTAFISGLTGQFYRQFALTIAISTVISAFNSLTLSPALAAV
LLKGHHEPKDRFSVFLDKLLGSWLFRPFNRFFDRASHGYVGTVNRVLRGSSIALLVYGGLMVLTYFGFSSTPTGFVPQQDK
QYLVAFAQLPDAASLDRTEAVIKQMSEIALAQPGVADSVAFPGLSINGFTNSPNSGIVFTPLKPFDERKDPSQSAGAIAAA
LNAKYADIQDAYIAIFPPPPVQGLGTIGGFRLQIEDRGNQGYEELFKQTQNIITKARALPELEPSSVFSSYQVNVPQIDADID
REKAKTHGVAISDIFDTLQVYLGSLYANDFNRFGRTYQVNVQAEQQFRLEPEQIGQLKVRNNLGEMVPLASFIKVSDTSG
PDRVMHYNGFITAELNGAPAAGYSSGQAQAAIEKLLKEELPNGMTYEWTELTYQQILAGNTALFVFPLCVLLAFLVLAAQ
YESWSLPLAVILIVPMTLLSAITGVILAGSDNNIFTQIGLIVLVGLACKNAILIVEFAKDKQEEGMDRVAAVLEACRLRLRPILM
TSIAFIMGVVPLVISTGAGAEMRHAMGVAVFSGMIGVTFFGLLLTPVFYVLIRRFVENREARRAANDKGLPEVHA

>MexW_P_aeruginosa
MAFTDPFIRRPVLASVVSLLIVLLGMQAFSKLVIREYPQMENALITVTTLYAGANAETIQGYITQPLQQSLASAEGIDYMTSV
SRQNYSTISIYARIGANTDRLVTELLAKSNEVKSQLPPDAEDPVLQKEAADASALMYISFYSEQMNNPQITDYLSRVIQPKL
ATLPGIAEAEILGNQVFAMRLWLDPVKMAAFGVTAGEINQAVQQYNFLAAAGEVKGQLVVTSVNASTDLKSPQAFAAIP
VKTDGDRRVLMGDVARVELGAASYDAISSFNGIPSVYIGIKGTPSANPLDVIKEVRAKMPELEEQLPPNLKVSIAYDATRFI
QASIDEVVKTLGEAVLIVIVVVFLFLGAFRSVLIPVVTIPLSMIGVLFFMQAMGYSINLLTLLAMVLAIGLVVDDAIVVVENIHR
HIEEGKPPFEAALEGAREIAVPVVSMTITLAAVYAPIGFLTGLTGALFKEFAFTLAGAVIISGIVALTLSPMMCSRLLRHEENPS
GLAHRLDLIFEGLKQRYQRALHGTLDTRPVVLVFAVLVLALIPVLLMFTKKELAPEEDQGIVFLMTNSPQTANLDYLNRYTA
EFEGIFRSFPEYYSAFQINGYNGVQAGIGGMLLKPWDEREKSQMELLHAVQAKLNEIPGVQIFAFNLPSLPGTGEGLPFQF
VLNTANDYESLLQVAQRVKQRASESGKFAFLDLDLAFDKPELVVDIDREKAAQMGVSMQDLGVALASLLGEGEINRFTID
GRSYKVIAQVERPYRDNPGWLGSYYVKSRNGQLVALSTLIETHERARPRQLNQFQQLNSAIISGFPIVSMGEAIETVQQIA
REEAPRGFAVDYAGASRQYVQEGSALLVTFGLALAIIFLVLAAQFESFRDPLVIMVTVPLSICGALIPLFLGVSSLNIYTQVGL
VTLIGLISKHGILIVEFANQLRHEQGLGRREAIEQAAAIRLRPVLMTTAAMVLGVIPLILATGAGAVSRFDIGIVIATGMSVGT
LFTLFVLPCIYTLVARPDAPPGVTQAANAH

>MexI_P_aeruginosa

MTFTDLFVRRPVLALVVSTLILLLGLFSLGKLPIRQYPLLESSTITVTTEYPGASADLMQGFVTQPIAQAVSSVEGIDYLSSTSV
QGRSVVTIRMLLNRDSTQAMTETMAKVNSVRYKLPERAYDSVIERSSGETTAVAYVGFSSKTLPIPALTDYLSRVVEPMFSS
IDGVAKVQTFGGQRLAMRLWLDADRLAGRGLTASDVAEAIRRNNYQAAPGMVKGQYVLSNVRVNTDLTNVDDFREM
VIRNDGNGLVRLRDVGTVELGAAATETSALMDGDPAVHLGLFPTPTGNPLVIVDGIRKLLPEIQKTLPPDVRVDLAYETSRF
IQASIDEVVRTLVEALLIVVLVIYLCLGSLRSVLIPVATIPLSMLGAAALMLAFGFSVNLLTLLAMVLAIGLVVDDAIVVVENVH
RHIEEGKSPVAAALIGAREVAGPVIAMTITLAAVYTPIGLMGGLTGALFREFALTLAGAVIVSGVVALTLSPVMSSLLLQAHQ
NEGRMGRAAEWFFGGLTRRYGQVLEFSLGHRWLTGGLALLVCISLPLLYSMPKRELAPTEDQAAVLTAIKAPQHANLDYV
ELFARKLDQVYTSIPETVSTWIINGTDGPAASFGGINLAAWEKRERDASAIQSELQGKVGDVEGSSIFAFQLAALPGSTGGL
PVQMVLRSPQDYPVLYRTMEEIKQKARQSGLFVVVDSDLDYNNPVVQVRIDRAKANSLGIRMQDIGESLAVLVGENYV
NRFGMEGRSYDVIPQSLRDQRFTPQALARQFVRTQDGNLVPLSTVVRVALQVEPNKLIQFDQQNAATLQAIPAPGVSM
GQAVAFLDDVARGLPAGFSHDWQSDSRQYTQEGNTLVFAFLAALVVIYLVLAAQYESLADPLIILITVPLSICGALLPLALG
YATMNIYTQIGLVTLIGLISKHGILMVEFANELQLHERLDRRAAILRAAQIRLRPVLMTTAAMVFGLVPLLFASGAGAASRFG
LGVVIVSGMLVGTLFTLFVLPTVYTLLARNHAEVDKSPRSRQLAEADLLVNKA

>AdeJ_A_baumannii
MAQFFIHRPIFAWVIALVIMLAGILTLTKMPIAQYPTIAPPTVTIAATYPGASAETVENTVTQIIEQQMNGLDGLRYISSNSA
GNGQASIQLNFEQGVDPDIAQVQVQNKLQSATALLPEDVQRQGVTVTKSGASFLQVIAFYSPDNNLSDSDIKDYVNSSI
KEPLSRVAGVGEVQVFGGSYAMRIWLDPAKLTSYQLTPSDIATALQAQNSQVAVGQLGGAPAVQGQVLNATVNAQSL
LQTPEQFKNIFLKNTASGAEVRLKDVARVELGSDNYQFDSKFNGKPAAGLAIKIATGANALDTAEAVEQRLSELRKNYPTG
LADKLAYDTTPFIRLSIESVVHTLIEAVILVFIVMFLFLQNWRATIIPTLAVPVVVLGTFAVINIFGFSINTLTMFAMVLAIGLLV
DDAIVVVENVERVMSEDHTDPVTATSRSMQQISGALVGITSVLTAVFVPMAFFGGTTGVIYRQFSITLVTAMVLSLIVALTF
TPALCATILKQHDPNKEPSNNIFARFFRSFNNGFDRMSHSYQNGVSRMLKGKIFSGVLYAVVVALLVFLFQKLPSSFLPEED
QGVVMTLVQLPPNATLDRTGKVIDTMTNFFMNEKDTVESIFTVSGFSFTGVGQNAGIGFVKLKDWSKRTTPETQIGSLIQ
RGMALNMIIKDASYVMPLQLPAMPELGVTAGFNLQLKDSSGQGHEKLIAARNTILGLASQDKRLVGVRPNGQEDTPQY
QINVDQAQAGAMGVSIAEINNTMRIAWGGSYINDFVDRGRVKKVYVQGDAGSRMMPEDLNKWYVRNNKGEMVPFS
AFATGEWTYGSPRLERYNGVSSVNIQGTPAPGVSSGDAMKAMEKIIGKLPSMGLQGFDYEWTGLSLEERESGAQAPFLY
ALSLLIVFLCLAALYESWSIPFSVLLVVPLGVIGAIVLTYLGMIIKGDPNLSNNIYFQVAIIAVIGLSAKNAILIVEFAKELQEKGE
DLLDATLHAAKMRLRPIIMTTLAFGFGVLPLALSTGAGAGSQHSVGFGVLGGVLSATFLGIFFIPVFYVWIRSIFKYKPKTINT
QEHKS

>AdeB_A_baumannii
MMSQFFIRRPVFAWVIAIFIIIFGLLSIPKLPIARFPSVAPPQVNISATYPGATAKTINDSVVTLIERELSGVKNLLYYSATTDTS
GTAEITATFKPGTDVEMAQVDVQNKIKAVEARLPQVVRQQGLQVEASSSGFLMLVGINSPNNQYSEVDLSDYLVRNVVE
ELKRVEGVGKVQSFGAEKAMRIWVDPNKLVSYGLSISDVNNAIRENNVEIAPGRLGDLPAEKGQLITIPLSAQGQLSSLEQ
FKNISLKSKTNGSVIKLSDVANVEIGSQAYNFAILENGKPATAAAIQLSPGANAVKTAEGVRAKIEELKLNLPEGMEFSIPYD
TAPFVKISIEKVIHTLLEAMVLVFIVMYLFLHNVRYTLIPAIVAPIALLGTFTVMLLAGFSINVLTMFGMVLAIGIIVDDAIVVVE
NVERIMATEGLNPKDATSKAMKEITSPIIGITLVLAAVFLPMAFASGSVGVIYKQFTLTMSVSILFSALLALILTPALCATILKPI
DGHHQKKGFFAWFDRSFDKVTKKYELMLLKIIKHTVPMMVIFLVITGITFAGMKYWPTAFMPEEDQGWFMTSFQLPSDA
TAERTRNVVNQFENNLKDNPDVKSNTTILGWGFSGAGQNVAVAFTTLKDFKERTSSASKMTSAVNTSMANSTEGETMA
VLPPAIDELGTFSGFSLRLQDRANLGMPALLAAQDELMAMAAKNKKFYMVWNEGLPQGDNISLKIDREKLSALGVKFSD
VSDIISTSMGSMYINDFPNQGRMQQVIVQVEAKSRMQLKDILNLKVMGSSGQLVSLSEVVTPQWNKAPQQYNRYNGR
PSLSIAGIPNFDTSSGEAMREMEQLIAKLPKGIGYEWTGISLQEKQSESQMAFLLGLSMLVVFLVLAALYESWAIPLSVMLV
VPLGIFGAIIAIMSRGLMNDVFFKIGLITIIGLSAKNAILIVEFAKMLKEEGMSLIEATVAAAKLRLRPILMTSLAFTCGVIPLVIAS
GASSETQHALGTGVFGGMISATILAIFFVPVFFIFILGAVEKLFSSKKKISS

>AdeG_A_baumannii

MNISKFFIDRPIFAGVLSVLILLAGLLSVFQLPISEYPEVVPPSVVVRAQYPGANPKVIAETVASPLEESINGVEDMLYMQSQ
ANSDGNLTITVNFKLGIDPDKAQQLVQNRVSQAMPRLPEDVQRLGVTTLKSSPTLTMVVHLTSPDNRYDMTYLRNYAV
LNVKDRLARLQGVGEVGLFGSGDYAMRVWLDPQKVAQRNLTATEIVNAIREQNIQVAAGTIGASPSNSPLQLSVNAQG
RLTTEQEFADIILKTAPDGAVTRLGDVARVELAASQYGLRSLLDNKQAVAIPIFQAPGANALQVSDQVRSTMKELSKDFPS
SIKYDIVYDPTQFVRASIKAVVHTLLEAIALVVVVVILFLQTWRASIIPLLAVPVSIIGTFALMLAFGYSINALSLFGMVLAIGIVV
DDAIVVVENVERNIEAGLNPREATYRAMREVSGPIIAIALTLVAVFVPLAFMTGLTGQFYKQFAMTIAISTVISAFNSLTLSPA
LAALLLKGHDAKPDALTRIMNRVFGRFFALFNRVFSRASDRYSQGVSRVISHKASAMGVYAALLGLTVGISYIVPGGFVPA
QDKQYLISFAQLPNGASLDRTEAVIRKMSDTALKQPGVESAVAFPGLSINGFTNSSSAGIVFVTLKPFDERKAKDLSANAIA
GALNQKYSAIQDAYIAVFPPPPVMGLGTMGGFKLQLEDRGALGYSALNDAAQNFMKAAQSAPELGPMFSSYQINVPQL
NVDLDRVKAKQQGVAVTDVFNTMQIYLGSQYVNDFNRFGRVYQVRAQADAPFRANPEDILQLKTRNSAGQMVPLSSL
VNVTQTYGPEMVVRYNGYTSADINGGPAPGYSSSQAEAAVERIAAQTLPRGIKFEWTDLTYQKILAGNAGLWVFPISVLL
VFLVLAAQYESLTLPLAVILIVPMGILAALTGVWLTAGDNNIFTQIGLMVLVGLACKNAILIVEFARELEMQGATAFKAAVE
ASRLRLRPILMTSIAFIMGVVPLVTSTGAGSEMRHAMGVAVFFGMIGVTFFGLFLTPAFYVLIRTLNSKHKLHSAAVHEAPL
ASPHDH

>AcrB_H_influenzae
MYEEIRMKFTDIFIRRPVLAVSISLLMIILGLQAISKLAVREYPKMTTTVITVSTAYPGADANLIQAFVTSKLEESIAQADNIDY
MSSTSAPSSSTITIKMKLNTDPAGALADVLAKVNAVKSALPNGIEDPSVSSSSGGSGIMYISFRSKKLDSSQVTDYINRVVK
PQFFTIEGVAEVQVFGAAEYALRIWLDPQKMAAQNLSVPTVMSALSANNVQTAAGNDNGYYVSYRNKVETTTKSVEQL
SNLIISSNGDDLVRLRDIATVELNKENDNSRATANGAESVVLAINPTSTANPLTVAEKIRPLYESIKTQLPDSMESDILYDRTI
AINSSIHEVIKTIGEATLIVLVVILMFIGSFRAILIPILAIPISLIGVLMLLQSFNFSINLMTLLALILAIGLVVDDAIVVLENIDRHIKA
GETPFRAAIIGTREIAVPVISMTIALIAVYSPMALMGGITGTLFKEFALTLAGAVFISGVVALTLSPMMSSKLLKSNAKPTWME
ERVEHTLGKVNRVYEYMLDLVMLNRKSMLAFAVVIFSTLPFLFNSLSSELTPNEDKGAFIAIGNAPSSVNVDYIQNAMQPY
MKNVMETPEVSFGMSIAGAPTSNSSLNIITLKDWKERSRKQSAIMNEINEKAKSIPEVSVSAFNIPEIDTGEQGPPVSIVLKT
AQDYKSLANTAEKFLSAMKASGKFIYTNLDLTYDTAQMTISVDKEKAGTYGITMQQISNTLGSFLSGATVTRVDVDGRAY
KVISQVKRDDRLSPESFQNYYLTASNGQSVPLSSVISMKLETQPTSLPRFSQLNSAEISAVPMPGISSGDAIAWLQQQATD
NLPQGYTFDFKSEARQLVQEGNALAVTFALAVIIIFLVLAIQFESIRDPMVIMISVPLAVSGALVSLNILSFFSIAGTTLNIYSQ
VGLITLVGLITKHGILMCEVAKEEQLNHGKTRIEAITHAAKVRLRPILMTTAAMVAGLIPLLYATGAGAVSRFSIGIVIVAGLSI
GTIFTLFVLPVVYSYVATEHKPLPVFDENKTTH

>AcrB_Salmonella_Typhi
MPNFFIDRPIFAWVIAIIIMLAGGLAILKLPVAQYPTIAPPAVTISATYPGADAKTVQDTVTQVIEQNMNGIDNLMYMSSNS
DSTGTVQITLTFESGTDADIAQVQVQNKLQLAMPLLPQEVQQQGVSVEKSSSSFLMVVGVINTDGTMTQEDISDYVAA
NMKDPISRTSGVGDVQLFGSQYAMRIWMNPTELTKYQLTPVDVINAIKAQNAQVAAGQLGGTPPVKGQQLNASIIAQT
RLTSTDEFGKILLKVNQDGSQVRLRDVAKIELGGENYDVIAKFNGQPASGLGIKLATGANALDTATAIRAELKKMEPFFPPG
MKIVYPYDTTPFVKISIHEVVKTLVEAIILVFLVMYLFLQNFRATLIPTIAVPVVLLGTFAVLAAFGFSINTLTMFGMVLAIGLLV
DDAIVVVENVERVMTEEGLPPKEATRKSMGQIQGALVGIAMVLSAVFIPMAFFGGSTGAIYRQFSITIVSAMALSVLVALIL
TPALCATMLKPVAKGDHGEGKKGFFGWFNRLFDKSTHHYTDSVGNILRSTGRYLLLYLIIVVGMAYLFVRLPSSFLPDEDQ
GVFLTMVQLPAGATQERTQKVLDEVTDYYLNKEKANVESVFAVNGFGFAGRGQNTGIAFVSLKDWADRPGEKNKVEAI
TQRATAAFSQIKDAMVFAFNLPAIVELGTATGFDFELIDQAGLGHEKLTQARNQLFGEVAKYPDLLVGVRPNGLEDTPQF
KIDIDQEKAQALGVSISDINTTLGAAWGGSYVNDFIDRGRVKKVYVMSEAKYRMLPDDINDWYVRGSDGQMVPFSAFSS
SRWEYGSPRLERYNGLPSMEILGQAAPGKSTGEAMAMMEELASKLPSGIGYDWTGMSYQERLSGNQAPALYAISLIVVFL
CLAALYESWSIPFSVMLVVPLGVIGALLAATFRGLTNDVYFQVGLLTTIGLSAKNAILIVEFAKDLMDKEGKGLVEAMLEAV
RMRLRPILMTSLAFMLGVMPLVISSGAGSGAQNAVGTGVLGGMVTATVLAIFFVPVFFVVVRRRFSRKSEDIEHSHSTEHR

>AcrF_Salmonella_Typhi

MANFFIRRPIFAWVLAIILMMAGALAIMQLPVAQYPTIAPPAVSISATYPGADAQTVQDTVTQVIEQNMNGIDNLMYMS
STSDSAGSVTITLTFQSGTDPDIAQVQVQNKLQLATPLLPQEVQQQGISVEKSSSSFLMVAGFVSDNPNTTQDDISDYVA
SNIKDSISRLNGVGDVQLFGAQYAMRIWLDANLLNKYQLTPVDVINQLKVQNDQIAAGQLGGTPALPGQQLNASIIAQT
RLKDPEEFGKVTLRVNTDGSVVHLKDVARIELGGENYNVVARINGKPASGLGIKLATGANALDTATAIKVKLAELQPFFPQ
GMKVVYPYDTTPFVKISIHEVVKTLFEAIILVFLVMYLFLQNIRATLIPTIAVPVVLLGTFAVLAAFGYSINTLTMFGMVLAIGLL
VDDAIVVVENVERVMMEDNLSPREATEKSMSQIQGALVGIAMVLSAVFIPMAFFGGSTGAIYRQFSITIVSAMALSVLVAL
ILTPALCATLLKPVSAEHHEKKSGFFGWFNTRFDHSVNHYTNSVSGIVRNTGRYLIIYLLIVVGMAVLFLRLPTSFLPEEDQG
VFLTMIQLPSGATQERTQKVLDQVTHYYLNNEKANVESVFTVNGFSFSGQGQNSGMAFVSLKPWEERNGEENSVEAVI
ARATRAFSQIRDGLVFPFNMPAIVELGTATGFDFELIDQGGLGHDALTKARNQLLGMVAKHPDLLVRVRPNGLEDTPQFK
LDVDQEKAQALGVSLSDINETISAALGGYYVNDFIDRGRVKKVYVQADAQFRMLPGDINNLYVRSANGEMVPFSTFSSA
RWIYGSPRLERYNGMPSMELLGEAAPGRSTGEAMSLMENLASQLPNGIGYDWTGMSYQERLSGNQAPALYAISLIVVFL
CLAALYESWSIPFSVMLVVPLGVVGALLAASLRGLNNDVYFQVGLLTTIGLSAKNAILIVEFAKDLMEKEGRGLIEATLEASR
MRLRPILMTSLAFILGVMPLVISRGAGSGAQNAVGTGVMGGMLTATLLAIFFVPVFFVVVKRRFNRHHD

>AcrD_Salmonella_Typhi
MANFFIDRPIFAWVLAILLCLTGALAIFSLPVEQYPDLAPPNVRITANYPGASAQTLENTVTQVIEQNMTGLDNLMYMSS
QSSGTGQATITLSFIAGTDPDEAVQQVQNQLQSAMRKLPQAVQDQGVTVRKTGDTNILTIAFVSTDGSMDKQDIADYV
ASNIQDPLSRVNGVGDIDAYGSQYSMRIWLDPAKLNSFQMTTKDVTDAIESQNAQIAVGQLGGTPSVDKQALNATINA
QSLLQTPQQFRDITLRVNQDGSEVKLGDVATVELGAEKYDYLSRFNGNPASGLGVKLASGANEMATAKLVLDRLNELAQ
YFPHGLEYKIAYETTSFVKASIIDVVKTLLEAIALVFLVMYLFLQNFRATLIPTIAVPVVLMGTFSVLYAFGYSINTLTMFAMVL
AIGLLVDDAIVVVENVERIMSEEGLTPREATRKSMGQIQGALVGIAMVLSAVFVPMAFFGGTTGAIYRQFSITIVSAMVLSV
LVAMILTPALCATLLKPLHKGEQHGQRGFFGWFNRTFNRNAERYEKGVAKILHRSLRWILIYVLLLGGMVFLFLRLPTSFLP
QEDRGMFTTSIQLPSGSTQQQTLKVVEKVENYYFTHEKNNIMSVFSTVGSGPGGNGQNVARMFVRLKDWDARDPTTG
SSFAIIERATKAFNQIKEARVFASSPPAISGLGSSAGFDMELQDHAGAGHDALMAARDQLIELAGKNSSLTRVRHNGLDD
SPQLQIDIDQRKAQALGVSIDDINDTLQTAWGSSYVNDFMDRGRVKKVYVQAAAKYRMLPDDINLWYVRNKDGGMV
PFSAFATSRWETGSPRLERYNGYSAVEIVGEAAPGVSTGTAMDVMESLVHQLPGGFGLEWTAMSYQERLSGAQAPALY
AISLLVVFLCLAALYESWSVPFSVMLVVPLGVIGALLATWMRGLENDVYFQVGLLTVIGLSAKNAILIVEFANEMNQKGHA
LLDATLYASRQRLRPILMTSLAFIFGVLPMATSTGAGSGSQHAVGTGVMGGMISATILAIFFVPLFFVLIRRRFPLKPRPE

>AcrB_K_pneumoniae
MPNFFIDRPIFAWVIAIIIMLAGGLSILKLPVAQYPTIAPPAISITAMYPGADAETVQNTVTQVIEQNMNGIDHLMYMSSNG
DSTGTATITLTFESGTDPDIAQVQVQNKLALATPLLPQEVQQQGISVEKASSSFLMVVGVINTNGTMNQDDISDYVAAN
MKDPISRTSGVGDVQLFGSQYAMRIWMDPNKLNNFQLTPVDVISALKAQNAQVAAGQLGGTPPVKGQQLNASIIAQT
RLTNTEEFGNILLKVNQDGSQVRLRDVAKIELGGESYDVVAKFNGQPASGLGIKLATGANALDTANAIRAELAKMEPFFPS
GMKIVYPYDTTPFVKISIHEVVKTLVEAIILVFLVMYLFLQNFRATLIPTIAVPVVLLGTFAVLAAFGFSINTLTMFGMVLAIGLL
VDDAIVVVENVERVMAEEGLPPKEATRKSMGQIQGALVGIAMVLSAVFIPMAFFGGSTGAIYRQFSITIVSAMALSVLVALI
LTPALCATMLKPIQKGSHGATTGFFGWFNRMFDKSTHHYTDSVGNILRSTGRYLVLYLIIVVGMAWLFVRLPSSFLPDEDQ
GVFLSMAQLPAGATQERTQKVLDEMTNYYLTKEKDNVESVFAVNGFGFAGRGQNTGIAFVSLKDWSQRPGEENKVEAI
TARAMGYFSQIKDAMVFAFNLPAIVELGTATGFDFELIDQGGLGHEKLTQARNQLFGMVAQHPDVLTGVRPNGLEDTP
QFKIDIDQEKAQALGVSISDINTTLGAAWGGSYVNDFIDRGRVKKVYIMSEAKYRMLPEDIGKWYVRGSDGQMVPFSAFS
TSRWEYGSPRLERYNGLPSLEILGQAAPGKSTGEAMALMEELAGKLPSGIGYDWTGMSYQERLSGNQAPALYAISLIVVFL
CLAALYESWSIPFSVMLVVPLGVVGALLAATFRGLTNDVYFQVGLLTTIGLSAKNAILIVEFAKDLMEKEGKGLIEATLEAVR
MRLRPILMTSLAFILGVMPLVISSGAGSGAQNAVGTGVMGGMVTATILAIFFVPVFFVVVRRRFSKKSEDIEHSHQVEHH

>AcrD_K_pneumoniae

MANFFIDRPIFAWVLAILLCLTGALAILSLPVEQYPDLAPPNVRITANYPGASAQTLENTVTQVIEQNMTGLDNLMYMSS
QSSATGQATITLSFTAGTDPDEAVQQVQNQLQSAMRKLPQAVQNQGVTVRKTGDTNILTLAFVSTDGSMDKQDIADY
VASNIQDPLSRVNGVGDIDAYGSQYSMRIWLDPAKLNSYQMTTKDVTDAISSQNAQIAVGQLGGTPSVDKQALNATIN
SQSLLQTPEQFRNITLRVNQDGSEVTLGDVATVEMGAEKYDYLSRYNRQPASGLGIKLASGANEMATAERVINRLNELA
QFFPHGLEYKVAYETTSFVKASITDVVKTLLEAILLVFLVMYLFLQNFRATLIPTIAVPVVLMGTFAVLYACGYSINTLTMFAM
VLAIGLLVDDAIVVVENVERIMSEEGLSPREATRKSMGQIQGALVGIAMVLSAVFVPMAFFGGTTGAIYRQFSITIVAAMVL
SVLVAMILTPALCATLLKPVKPGESHERTGFFGWFNRTFNRSASRYETFVGKILHRSLRWMLIYVLLLGGMVFLFLHLPTSFL
PLEDRGMFTTSVQLPSGSTQQQTLKVVQKAEDYFLNNEKQNVESVFATVGSGPGGNGQNVARMFVRLKDWDQRDPQ
TGTSFAIIERATKAFNQINEARVIASSPPAISGLGSSAGFDMELEDHAGKGHDALMAARDTLLELAGKNPLLTRVRHNGLD
DSPQLQVDIDQRKAQALGVSIDDINDTLQTAWGSSYVNDFMDRGRVKKVYVQAAAKYRMLPDDINLWYVRNSSGTM
VPFSAFATSRWETGSPRLERYNGYSAVEIVGEAAPGISTGTAMDMMEKLAAQLPTGFGLEWTAMSYQERLSGAQAPALY
AISLLVVFLCLAALYESWSVPFSVMLVVPLGVIGALLATWMRGLENDVYFQVGLLTVIGLSAKNAILIVEFANELNEKGQDL
LSATLSACRQRLRPILMTSLAFIFGVLPMATSTGAGSGSQHAVGTGVMGGMISATVLAIFFVPLFFVLVRRRFPLKERPQ

>AcrF_K_pneumoniae
MSKFFIHRPVFAWVLAIIMMIAGGLAILQLPIAQYPTIAPPAVAISATYPGADAQTVQDTVTQVIEQNMNGIDNLMYMSS
TSDSAGSVTITLTFKSGTDPDIAQVQVQNKLQLATPLLPQEVQQQGISVEKSSSSFLLVAGFISDNPTTTQDDISDYVASNV
KDPISRLNGVGDVQLFGAQYAMRVWLDGNLLNKYNLTPVDVINALQVQNDQIAAGQLGGTPALKGQQLNASIIAQTR
LKDPQEFGKVTLRVNADGSVVHLKDVARIELGGENYNVVARINGKPASGLGIKLATGANALDTATAIKAKLAELQPYFPQ
GMKVVYPYDTTPFVKISIHEVVKTLFEAIILVFLVMYLFLQNMRATLIPTIAVPVVLLGTFAVLSMFGYSINTLTMFGMVLAIG
LLVDDAIVVVENVERVMVEEKLSPKEATEKSMSQIQGALVGIAMVLSAVFVPMAFFGGSTGAIYRQFSITIVSAMALSVLV
ALVLTPALCATLLKPASAEHHEKKGFFGWFNARFDQSVNHYTNSVSGILRGTGRYLVIYLLIVVGMAVLFMRLHTSFLPDE
DQGVFLTMIQLPSGATQERTQKVLDTVTDYYLHNEKANVESVFTVNGFSFSGQGQNSGMAFVSLKPWEARSGDENSVE
SIIKRATVAFSQIKDAMVFPFNMPAIIELGTATGFDFELIDQGGLGHTALTQARNQLLGMVKQHPDQLVRVRPNGLEDTP
QFKLDVDQEKAQALGVSLSDINETISAALGGYYVNDFIDRGRVKKVYVQADAHFRMLPSDINNMYVRSANGEMVPFSAF
VTSRWIYGSPRLERYNGLPSMEILGEASPGKSTGEAMALMETLASKLPSGIGYDWTGMSYQERLSGNQAPALYAISLIVVF
LCLAALYESWSIPFSVMLVVPLGVIGALLAATLRGLNNDVYFQVGLLTTIGLSAKNAILIVEFAKDLMEKEGKGIIEATLEASR
MRLRPILMTSLAFILGVMPLVISHGAGSGAQNAVGTGVMGGMLTATLLAIFFVPVFFVVVRRRFTRHAE

>AcrB_E_ictaluri
MAKFFIDRPIFAWVLAIIMMLAGALAIMSLPVAQYPTVAPPAVTISASYPGADAATVQNSVTQVIEQNMNGIDNLMYMS
STSDSAGNASITLTFEAGTDPDIAQVQVQNKLQLAMPSLPQEVQQQGVSVDKASSSILLVAGFISEDGSLSQDDIADYVA
SNIKDPLSRTQGVGSVQLFGSQYAMRIWLDPNKLNKYNLTPVDVVSQIKVQNNQIAGGQLGGTPPVPGQQLNASIIVQ
TRLKDPTEFGKILLKVQQDGSRVLLRDVARVELGAENYGTIARYNGKPAAGIAVKLATGANALDTAKAVKTELARLAPYFP
ASLKTVYPYDTTPFVQLSIDEVVKTLLEAIVLVFVVMYLFLQNIRATLIPTIAVPVVLLGTFAVLEIVGFSINTLTMFGMVLAIGL
LVDDAIVVVENVERVMSEEGLPPKEATKKSMEQIQGALVGIAMVLSAVFVPMAFFGGSTGAIYRQFSITIVSSMVLSVLVA
MILTPALCATMLKPIAKGDHGVQTGFFGWFNRLFEKSTHHYTDSIGRILRGTGRYLVIYLLIVVGMGLLFIRLPTSFLPEEDQ
GVLLTMAQLPPGATQERTNNVLKQVSHYYLTKEKDNVESVFTVSGFGFSGQGQNNGLAFVSLKPWDERKGDQNKVTAI
IQRAAVAFSKINDAMVYPFNLPAIVELGTASGFDFELIDQANLGHEKLTEARNQLLGMAAQHPDLLVGVRPNGLEDTPQF
KLDIDQEKAQSLGVSLADVNQTISAALGGAYVNDFIDRGRVKRVYVQADAPFRMLPSDIDNWYVRSSNGQMVPLSAFS
SSRWEYGSPRLERYNGLPSMEILGEAAPGKSTGEAMALMETLVAKLPAGVGYDWTGMSYQERLSGNQAPALYAISLIVVF
LCLAALYESWSIPFSVMLVVPLGVVGALLATSLRGLTNDVYFQVGLLTTIGLSAKNAILIVEFAVDLMHKEGKGLVEATLEAV
RMRLRPILMTSLAFILGVMPLVISNGAGSGAQNAVGTGVMGGMVSATILAIFFVPVFFVVVRRRFGRRSEDLEHSRPVEPH
NK

>AcrF_A_hydrophila

MARFFIDRPIFAWVIALVIMLAGSLAIIKLPVAQYPSIAPPAVGISASYPGASAKTVEDSVTQIIEQNMTGLDHLLYMSSQSD
SSGRVSVTLTFQPGTDPDIAQVQVQNKLQQAMSLLPQEVQQQGIRVQKTSSSFLMVAAFISKDGAMTNDDLADYVVA
NIKEPLSRLDGVGDITLFGSQYSMRVWLDPNKLNRVQMTPGDVQAAIKAQNTQVAFGKLGGTPAVSDQRFTATIMGQT
RLSTVEEFNDILLRVNQDGSKVRLKDVARVELAGESYDAEALYNGQSTAAVAIKLATGANALDTAEKVRGKLNELSTYFPA
NMEIVYPYDTTPFVKISIEEVVQTLVEAIFLVFCVMYLFLQNFRATLIPTIAVPVVLLGTFGVMSAFGFSINTLTMFGLVLAIGL
LVDDAIVVVENVERLMSEEDLSPLEATRKSMTQITGALVGIALVLSAVFVPMAFFGGSTGAIYRQFSLTIVSAMVLSVLVALI
LTPALCATLLKPIKHGEFGAKRGFFGWFNRAFDASANRYQSGVRKVVKQGVRYGIIYAAMLAVLAILFMRMPTSFLPEED
QGVIMSMVQLPVGATKQRTEVVLADMRDYFMKNEKDNVDSVLTVAGFSFAGSGQNSGMAFIKLKDWKERNTPDRSA
NAIIGRAMGYLFSIKEAQVFAFNLPPIPELGTATGFDFFLQDRAGVGHDKLMAARNQLLGMAAQDPNLVRVRPNGMED
TPQLDIKIDYEKALAQGLSISDINSTLSSAWGSAYVNDFVDRGRVKKVYLQADAPFRMNPEDLKLWYVRNSAGQMVPFS
AFASTDWSFGSPRLERYNGVPAMEIVGEAAPGKSTGDAMAAIEQMVKQLPEGIGIEWTGLSYQERQAGSQAPALYAISLL
VVFLCLAALYESWSIPFSVMLVVPLGVLGAIVAATLRGLENDVYFQVGLLTTIGLSAKNAILIVEFAKELYDRGMGLSEAVVE
AARLRLRPILMTSLAFILGVLPLVISTGAGASSRNAIGTGVMGGMISATVLAIFFVPLFFVLVMRYFTKHSTKQARLAHAEEIK
HD

>AmrB_A_hydrophila
MPQFFIHRPVFAWVIALFIVLAGVIAIPKLPISHYPSVAPVSVTLYATYSGATPQTLNDAVVSLIERELSGVKNLLYFESSVDA
SGSAQITATFKPGTDPELAQMDVQNRIKAVEPRLPQAVRQNGLQVESASSGFLMMVGMTSPDGRFEEVALNDYMARN
IVQELRRIDGVGRVQLFGAEQAMRIWVDPGKLAAYGLTISEVAQAIEQQNVQIAPGRLGDEPALPGQRLTVPLTVEGQLT
TPEAFAAIVLRAGTDGAKLVLGDVARVELGAQSYAFANRENGVAATSAAIQLSPGANAVRTADAVRTRLAQLSPTLPAG
MAYSIPFDSAPFVKVSIEKVIHTLLEAMVLVFLVMFLFLQNLRYTLIPAIVAPIALLGTFAVMLLAGFSINSLTMFGMVLAIGIIV
DDAIVVVENVERLMASRGLSPREATSQAMKEISGAVIGITLVLTAVFIPMAFGGGSVGVIYQQFTLSMAVSILFSAFLALTLT
PALCATLLRPISPDHHEKRGFFGAFNRGVERLTAGYGARVTSLVGRSGRVMATFAALCAVLAIAATQLPSSFLPDEDQGYF
MTSIQLPTGATSERTQAVIKEFEAHVASREALDTNLVVQGFSFSGAGPNAAMAFTMLKDWDQRHGATAAEEAELAQAA
MANSHEGTVMSLMPPTIDELGTASGFTLFLQDRANRGEAALLAAQAQLLALAANSKVVSDVYPDSLPPGQSVHLEINRQ
KAEAMGLSFTSVSSTLSAAMGSLYVNDFPNDGHMQQVILQADASARMQLDDVLGLRVRNASGGMVPLREVVTPVWR
ESPQQMMRFDGFPAVRIAGGAAPGISSGAAMAEMERLVAQLPPGFAVAWTGQSLQERQSAAQAPLLMLLSGLVVFLV
LAALYESWSIPLSVMLVVPLGLLGAVAAVMLRGMPNDVFFKVGMITIIGLSAKNAILIVEFARQLHGEGRSLLDAAVTAARL
RLRPILMTSLAFTLGVVPLMLASGASAETQHAIGTGVFGGMISGTLLAIFFVPVFFVFIIGAQERLQRWQARRAEQRGSQ

>MexFQ_A_hydrophila
MFSKFFIERPIFASVISIIIVLGGLAAMRSLPIEQYPQITPPVVSVTAFYPGATPEVISQTVAAPLEQQINGVERMLYMQSGSA
SNGQMNLNVYFEIGTDPDQATINVNNRVSAAMAQLPEEVKKQGVTVKKKSTSILQVVTLQSPDGTFDTTYLSNYALLNII
DELKRIPGVGDTTLFGGTDYAMRIWLRPDRLAQLELTPSDVIGAIREQNTQFAAGKIGAQPTTAPIDFTYTVQTKGRLEDV
REFQNIIIRSMPDGSKIRVKDVARVELGGKDYDLLAKANGKPAIGIATYLQPGANAVAVADQVHATMLRLKERFPQDIEYQ
IPYDTTEFVKISIEEVVHTLFEAIVLVFVVVYLFLQNFRATLIPCIAVPVSLIGTFAGMLVLGFSINLLTLFGMVLAIGIVVDDAIV
VLENVERIMQEKKCSPKEAAILAMQEVSGPVVAIVLVLCAVFLPVAFMGGMTGVMYKQFAITVAVSVAISGLVALTLSPAL
CAVLLKEGHHKPARFFVWFNNAFDGLTRRYVRGVAFLNRRVGVAFTIIAFILVSIYGLLMKVPGELVPNEDQGYLLSAVML
PDAASLTRTQAVANDFDQMAMANPNVKDVITFSGFDILSNAIISNSGISFITLKDWSERQGAGQDSFSLAKTFQGMSLM
GLADGFVASFNPPPITGMSTTGGLEAYLQNRSTGNAQAFSAEVQRFLEAAKQRPEFASVTSTYRANVPQVYLDLDREKA
KALGLPINAVFDTMQATFGQVYVNDFNQFGRTYRVQLQSEADYRAKKSDIRNVYVRSDKGEMIPLSSLVTVRDATGPEL
VERFNIFQAAKIMAQPAPGYSSGQAIAALEAVADESLGNDAKLEWTGSAYQEKAAAGSAGTAFGFGIVMIFLILAAQYER
WSLPFAVITAVPFALFGALLATWLVGLTNNVYFQIGLVTLVGLAAKNAILIVEFAVMKHEEGMSLIESALEAARLRFRPIVMT
SLAFILGCVPLVTSSGAGAASRHALGWPVIGGMLAATFIAIFFIPLFFRLIMRRSERAGGKTAQPKS

>AcrB_A_hydrophila

MRFTDIFIKRPILAISISFLIALLGVQAVFKMQVREYPEMTNTVVTVSTSYYGASADLIQGFITQPLEQAIAQADNIDFMTSST
QLGSSTITAYMKLNTNPNAALSDILAKVNSVRSQLPKESEDPSVTMSTGSTTAVLYIGFTSPELNASQITDYLERVIKPQLFT
VGGVSKVDMYGGTKYALRVWLDPAKMAAFNLTAADVMSVLNSNNYQSATGQATGYFTLYNGNAETQVNTTEELERL
VVATRDGKVIRLADIAKVSLEKSHDTYRATANGREAVVLAINAAPTANPINIAHDVLEMLPSLKSNMPSTMEANVLYDSTV
AINESISEVIKTIAEAAAIVLVVITLFLGSFRAVIIPIITIPLSLIGVVMVMQMFGFSINLMTLLAMVLAIGLVVDDAIVVLENVDR
HVKEGEEPFRAAIIGTREIAVPVIAMTVTLAAVYAPIALMGGITGSLFKEFALTLAGAVFVSGIIALTLSPMMCAKMLKANEEP
GKFESKVHHLLDRMTVRYDAMLHAVMQKRIVVVLFAVIVFASLPMLFKFIPSELAPNEDKGVMAVLGTAPSNVNLDYIQG
TMEQVNKVLDEQPEVAFSQVFSGVFNANQAFGIASMVPWSQREASQKEVLERVGKLVENVPGMSITTFQFPELPGASS
GLPIQFVITTPNNFESLYQVASEILTAAQGSGRFVYSELDLQYDSATMKIKIDKDKAGAYGITMQDIGITLGTMMSDGYVN
RIDLDGRSYEVIPQVERKYRLNPESIKGYYVRAADGQSIPLGSLISIEVVGEPRALPHFNQLNSATIGAVLMPGTTMGNAIA
WLDATASEKLPQGYRYDYMGEARQFVTEGSALYATFALALAIIFLVLAIQFESARDPLVIMVSVPLAISGALIALAWGAATM
NIYSQVGLITLVGLITKHGILICEVAKEEQLLHGRNRMEAVMAAAKVRLRPILMTTAAMIAGLIPLLYATGAGAAQRFSIGIVI
VAGLAIGTLFTLFVLPVIYTYLASEHKPLPVFDESIPPKAKGSH

>AcrB2_C_burnetii
MKLSEICIRQPVLAIVLSLILVVLGVVAFQRLELRFFPKLELPILTVATTYDGASAQLMESQVTTQIENALSGVDHIASISSTSYP
GSSYVTVLFQLGGNFEEEASAVRDKVFAIRDQLPPNANPPTITVGTKGNPVLGIGFIDPQKSSADIRDYVERAVQPALRQL
SGVGEVDILGASDYAMRIWLNASKMVAHQVTVTDVKNALTANNIYFPAGSIQEPKRNYSIVSHTQLKDATAFGNIIIKNN
DGGTVRLNDIGHAELGYRSLYEAPMRINGQNGIELLIEPLQEANPVTVAHEVKQALATIQKNLPPGMQASVNYDASTFLK
SSIEETFYAMGEAVLLVIFVVFLFLGSARAASIPIITIPVSLIAVFAFIKLFGFTINTMSLLGIVLAIGLVVDDAIVMLENIHRHIEEG
MTPFQAAIKGSREISFAIIAMALTLVAVYAPVGFVQGFTAELFKEFAFTLAAAVVISAFIALTLSPMMCSRILLPRTKDSRLTIII
DRAFSQLTGFYQRFLKFALNKRRLFIAGLLAIAGVGYVLFISLPSELIPKEDIGLIQVSVTSPSGASLNYTNQYAEQVEKIIQQN
PAVASVMSQVSTTSVNIRVTLKPWGERRETTAQVIANLNPKLAAIPGIDATAYLPDVVNYGLQGNDIDLNFMTAGDYTD
LLDPINKMMKLLKHYPGVTDLHTNLKYDTQQYAITIKRDLAAVLGVNIQDIADTVSAMMSGNHWTDVQAGNKSYEVIV
QMDKQDLTDFNALKKLYVHSNNKTDANKGNSIPVSSLIDLTPTVGQGSFTHFNRFRSGTISARLAPGYSESQVIDYIKAHV
PFVMTSDVHYAFSGKAAQFLQSSKSMIGIILMSLIFIYLVLSAQFGSFLDPFVILLAVPLCIVGGLLSLKLTGGTLSIYSQIGLVT
LIGMISKHGILITQFINDLRKQGMAMQEAILRGAMIRLRPVLMTTLAMVFGALPLALASGPGSIGRHQIGWVIVGGLLFGTF
FSLIVVPIAYSYLGRFKKR

>AcrB_C_burnetii
MNISEICIKRPVLALVLSLVLVVVGIMGFNDLNTRFFPKFEQNVINISTAYPSASAKLVESSITTPLEEGISGVQGIDTIISQSFQ
GESDIKLTLDYGVNVYEVANQIRDKVEQARVQLPTNINAPVVQVGWGNMELMDVGFTAPNENPKAIRDYLERNVIDQIE
QIPGIADVEVAGANQYAMRIWLDPQKMAARGLSVNDVQMAIQNSNVELPAGEIKGSSINYPITAETKLNTADQFNNIIV
KNDNGQVIRIKDIGQAQLDDDSADQSIVTLDGQPGVLLSINNATDANPLDAAKNVNHFLNQIRPHLPNGMKIVPSFDIS
AYMHGSIHEVYISIGIAILCVILVIFLFLGRLRTVAIPIATIPVCLIASFGAMYLLGFSINVITLLALVLSIGLVVDDAIVMLENIYRHI
ENGEKPLIAAIKGSKEITFPVIAMTITLAAVYAPIGLMQNQAANIFRSFAFTLAGAVLISGFVALSLSPMMCSRFLKAQGGEP
KGYSRIIETIYERMAQGYQKILAAILNKRILVVFAAVLIAVGGFFLAKNIPMAFVPQEDMGFVIAAVSTSSGSSINTINRGLEQ
VASIFKQAPAVASVVTVADTNAKAFDSVFAMLKPFKDRKQSASQVADVINQKIKQIPGLNAAAFAPSFGGSMQHQLEFY
LMGDTYSDLYAVSQKLINQLGNYPGLRVIQSNMRFDSQQYNMTVNRDIAGQLQANIKDIDNTMAALYGGSKISTFDAN
GKTYNVYLQAQENDLHSLASINKFYVNNAQNKLIPLSNLISIKPILSQVVLPHYNHLRAAQMSAQLAPGYNLGTVVNYLQ
TELPQLLPANVKYAFTGQAHDLLTSNNSMGLIFLLAMVFIYLVLAAQFESFIDPFIILLAVPLSIVGAMASLKLIGGTLNIYTDI
GLVTLIGLVSKHGILITQFANRLQKEGLAMREALLKAASIRLRPILMTTAAMVFGALPLLFSSGGSAQSRHQIGIVIIGGLIFGT
FFSLVVVPVAYSYAYKFKLFLKRFKTTDEMSLSGK

>MexIW_C_burnetii
MASSKFTDKFIIRPVLAIVVSLFIFILGLRAITEMEVRQYPKMNNTLITISTAYPGAPASLIEGFITTRLEKSIAGAEGIDYMTSTST
QGVSTINVFIKLNFDPNVAFTDIMSKVAQVQNDLPRESQLPVIQKLTGTQTALMYISFSSDQMSSEQISDYLNRVVQPKIE

TVFGVSQAEILGGQTFAMRVWLNTKRMAALNVTPTDVTQALINNNFQSAAGQTKGDFITLYIKAKTDIGDKQAFENLVIK
EVNGTLIRIRDIGRVELGAEDYDSSVYMNGKSAIFVAVNTTPTANPLSVIANVKKILPDLEKEYPPSLHSKVVYDATEYIQASI
YEVIRTIFEATLIVIVVIFLFLGSIRTVVIPVITIPLSLIGVCFLMWFLGYSLNLLTLLSMVLAIGLVVDDAIVVVENVYRHIEEGMD
GFHAAIRGAREIAFPIISMTLTLAAVYAPIGFMTGLTGALFTEFAFTLAGAVIVSGIIALTLSPMMCSKFLNQNIGKQRYVQFI
DRSFERLKQFYSRRLHGVLNHRFLMVLLAVAVLASCFIFYMNTTSELAPEEDQGALFVQAQAPQYANIDYVEAYSKQYEK
YFKKLPSMANYFMINGFGTVNTVLAAMIMKPWDQRQQSQKQAQAILQPELNTVAGLQTVVFPLASLPGGGTGLPVQF
VITSIAPFNKIYLATEQIVEEAKRSGLFIFVDSELKYNNPQLNVIIDRNKAGDLGVTMQSLSQILAYSLGGNYINRFAMMGQS
YKVIPQLSRLFRLNPSNLEQIYVNTAGGQLIPLSTLVKMNITTQPNLLSHFQQLNSAQIDAVLAPGHTMGEALTYLEQTAN
KVLARDMSYDFGGQMRQFVQEGQALVVTFFLAIIIIYLVLAAQFESFRDPFVILISVPMSICGALIFLNLGLATINIYTQIGLVT
LIGLISKHGILMVEFANKLQEEEGLSVREAIEKSAAIRLRPILMTTAAMVLGVVPLILATGAGAVSRFDIGLVIATGMLIGTMFT
LFVVPTMYLLIAKKHQPLRKVDE

>MexFQ_M_hydrocarbonoclasticus
MLRTFIDRPIFASVISIIITLGGALALLGLPVEQYPNVVPPQVVVDGRFPGASADVISDSVVAPLEQEINGVDDMIYMESSAT
DSGSFRVTVSFEIGTDPDQATINVNNRVQQALARLPQTVRDQGLKVESRSTSILQVITLSSPDNSMDVVEISNYALLNVLD
ELVRLPGIGDASLFGAQDYSMRIWLRPDKLAQFELTPADVAGALRAQNAQFAAGRIGAEPAPEGQAFTFSVSAPGRLTSP
EEFGEVILRSDDQGASLRLKDVARIELGAQNYDFAAIYNGGATVPMGVYLQPGANALDAAEAVNEAMKEIADRFPEGLEY
NVPYDTTRFIDISIQEVISTFIVAVLLVVVVTFLFLQHFNATLIPLIAIPVSLVGTFAGMQAMGFSVNLLTLFGLILAIGVVVDN
AIIIMENAERLIKEEGMSPYNAAVTTVSQVSGAVVASTLVLVAVFAPVAFLGGLSGELYRQFAITIAVSVVISGVVALTLTPA
MCALLLGHEGKQLTVFRWFDAGFDKVTNGFSAVVDWLLRHAVIGVLLFAAMIGGTVFLLNKMPSGLVPQEDQGFVLTA
YSLPATSSLSRTEGTRDELAQNMLQLPEVKDVVAFAGFDIISSALRTNSGVAFVTLEDWAEREGEGQGAADIANKIMGIGF
GMPEAFVIAFTPPPIQGLSTTGGVEGYIQARGGRTPAEIKAMADRFTQAANQRPELTNVRVTLDTGIPRYQAIVDREKAQ
AAGVPVDQIFTTMQSTFGGLYVNDFTLQGRNWQVTLQSEGEFRSHPDDLRKVFVRSSYGEMIPLSSLVELERVSGPDILN
RFNVYPAAKLLADPTPGHTTGEALTALQEVANQQFDRNTLLGWTGEAYQLQDSADSGTLAFGMGLLLVFLILAAQYER
WGLPVAVATAVPFGVFGAALASLLRGFPNDIYFQVGLLVLIGLAAKNAILIVEFAAQNRQTGMSSFEAASTAARQRFRAIM
MTALTFIVGSLPLAFSTGAGAVSRQEIGTVVVGGMLVASTLALFFVPLFYKLIEDLADWRRKRKAA

>MexFQ2_M_hydrocarbonoclasticus
MISRVFIQRPKFAFVISILITLAGALALRILPVNMYPDMAPPQVQVTATLPGASAQVVEEAVIRPVEQQLNGVEGMIYMESS
ASSDGTAKITLTFESGTDTDIAQVNVQNRVALAEPFLPDEVRRQGVIVSKQTGNMLLGINLTSSRDDLDGVFLSNYASNN
LVEALGRVAGVGSAEVMGAKDYSMRVWLNPRRMAALDVTVAEVAAALKEQNQIVAAGKLGQSPVPAGQQFVYSIQTR
ERLSDPEEFGQTILRSRPDGGFVRLRDVGRIELGSRSYTSTAKLNNRDTAFLVIYQLPDANALDVAERVKALVESASASFPEG
VEYSVLYDTTEFISRSISEVVVTLFQAVGLVILVVFLFLQNWRATLIPSIAIPVSLIGTFAVMSVLGYSINITITLFGLVLAIGIVVD
DAIVVIENVERILKEEALPIREAVSKAMAEVTGPVIATTLVLLAVFVPVAFMPGITGKLYQQFSVTISVAVIISSVNALTLSPALC
VALLGRGGDQSVRWLRPFETLITRSTGGYSKAVAELLRKGMLVGLVLLVVLVAGGGLFKAVPSAFVPPEDQGFLFVDVQL
PDAASLERTDAVLEKVTDMVLEDPAVTDFITVSGFSLLGGAASNNGLGIVMLKDWEDRETPELGLGSVIPRLYSKLWTVSE
AQVMVFNPPPIPGLGTSAGFDYRLQDNLGRDVSELAQVMNGLIFQANQHPALSRVYSTYRANIPQYLLEVDRNKAKAL
GIQLSDIFSTLQTQLGSLYVNDFTRFGKNYRVLLQAEGEYRQRPEDLTHYFVRNQEGDMVPLTTLASLEPVLGPSSIQHFN
LKRSVTLSGEPAPGFASGDAINAMEALSESLPQGYEFSWSGQSLQEIQAGNLAAVIFVLALVFVYLFLVAQYESWSLPAAV
LGAVPLALAGAMAGLWLVGLANNIYAQVGLVLLIGLSTKTAILIVEFAMQLRASGLSTAEAAQKAAVLRFRAVLMTALSFV
LGVLPLVFASGAGAASRVSLGITVLSGMVAATILGTLLVPVFYQWIQSIRDRRMASGQGAL

>MexIW_M_hydrocarbonoclasticus
MKFTDIFVHRPVLATVVSLLILLIGARAAMEMEIRQYPELESTTVTVSTAYPGASSDLIKGFITTPLQQAIAEASGIDYLTSTSS
QSVSTIQAKMILNYDANDALAEIQAKVASQRNVLPAEAEDPVITSTTGDSTALMYIAFYSTELDVPQITDYLTRVVQPKLQ
ALAGVGKAELLGRTFALRIWLDPERLAAVDMTPVEVAAKLRANNYQAAVGKTRGKYTEVSLTSDTDIADPDAFRNLVVK
QSNGTLIRLQDIARVELGSQTYDSLALYKGQPATYVAIELAPGANPLTVAELVKAELPDIESQLPSGLNVRLAYDASDFIDD

SINEVIQTLLEAMIIVLVVVFLCLGSVRASVVPSVAVPLSLIGGAFVMLMFGFSLNLLTLLSMVLAIGLVVDDAIIMVENVHR
HIEQGESRFDAAINGAREMATPIIAMTTTLVAVYAPIGFMGGLVGSLFTEFAFTLAGTVVISGIVALTLSPMLSAKVLKPHGD
PGRFEKVVEKSFNGLANGYKAALASLMQTKSVVVFFAFVVLGSIYFMLMMSQNELAPTEDQGILFYQGLGPETSTLDYLY
DHGQEVQNRMSEVPGYSEDFMILGITSPNAVFGGFKLAPWSEREITQFEVQPKLDAELKKVTGLQTAVFPRPSLPGSGGG
LPFQFVITTGGSYEQLDQVADEMLGKAMQSGNFMFLKKSINFDKPVTRINVDRDRVADLGLSMQDVGQSLASMLSGGY
INRFNMEGRSYQVIPQVDDQFRPSKEALQDYYIRAGNGELVPLSSVVSFTEEVEPSNRTQFNQQNSLTLEGVVMPGVAA
GTAMEFMEQTASDVFPQGFSYDYTGETRQLATQGSALMVTFFLSLLVIYLVLAAQFESWRDPIIILVSVPMSVAGAMAFIV
LGFATMNIYTQVGLITLIGVVSKNGILIVEFANQLQKERGLNKVDAVIEAAAIRLRPIIMTSLALIFAMVPLLIAIGPGAESRFAI
GLTISAGLGIGTLFTVFVLPAFYILLARDHHGSAADEYGNDEESSQVPAGNH

>AcrB_C_koseri
MPNFFIDRPIFAWVIAIIIMLAGGLAILKLPVAQYPTIAPPAVTISATYPGADAKTVQDTVTQVIEQNMNGIDNLMYMSSNS
DSTGTVQITLTFESGTDADIAQVQVQNKLQLAMPLLPQEVQQQGVSVEKSSSSFLMVVGVINTDGTMTQEDISDYVAA
NMKDAISRTSGVGDVQLFGSQYAMRIWMDPTKLNNFQLTPVDVINAIKAQNAQVAAGQLGGTPPVKGQQLNASIIAQ
TRLTSTEEFGKILLKVNQDGSQVRLRDVARIELGGENYDIIAKFNGQPASGLGIKLATGANALDTANAIRAELKKMEPFFPS
GMKIVYPYDTTPFVTISIHEVVKTLVEAIILVFLVMYLFLQNFRATLIPTIAVPVVLLGTFAVLSAFGFSINTLTMFGMVLAIGLL
VDDAIVVVENVERVMSEEGLPPKEATRKSMGQIQGALVGIAMVLSAVFIPMAFFGGSTGAIYRQFSITIVSAMALSVLVALI
LTPALCATMLKPVAKGSHGSTKGFFGWFNNMFEKSTHHYTDSVGNILRSTGRYLLLYLIIVVGMAYLFVRLPSSFLPDEDQ
GVFLSMAQLPAGATQERTQKVLDEMTDYYLTKEKDNVESVFAVNGFGFAGRGQNTGIAFVSLKDWADRPGDENKVDAI
TGRAMGAFSQIKDAMVFAFNLPAIVELGTATGFDFQLIDQGGLGHEKLTQARNQLFGEVAKHPDLLVGVRPNGLEDTPQ
FKVDIDQEKAQALGVSISDINTTLGAAWGGSYVNDFIDRGRVKKVYVMSEAKYRMLPEDIGNWYVRGSDGQMVPFSAF
SSSRWEYGSPRLERYNGLPSMEILGQAAPGKSTGEAMAMMEELASKLPSGIGYDWTGMSYQERLSGNQAPALYAISLIVV
FLCLAALYESWSIPFSVMLVVPLGVIGALLAATFRGLTNDVYFQVGLLTTIGLSAKNAILIVEFAKDLMDKEGKGLIEATLEAV
RMRLRPILMTSLAFILGVMPLVISSGAGSGAQNAVGTGVMGGMVTATVLAIFFVPVFFVVVRRRFSRKSEDIEHSHSVEHH

>AcrF_C_koseri
MANFFINRPIFAWVLAIILMMAGALAIMQLPVAQYPTIAPPAVAISATYPGADAQTVQDTVTQVIEQNMNGIDNLMYM
SSTSDSSGSVTITLTFQSGTDPDIAQVQVQNKLQLAMPLLPQEVQQQGISVEKSSSSFLLVAGFVSDNKNLTQDDISDYV
ASNVKDTISRTSGVGDVQLFGAQYAMRIWLDSNALNKYQLTPLDVINQLKTQNNQIAAGQLGGTPSIPGQQLNASIIAQ
TRLKTPEEFGRITLKVNQDGSMVHLKDVARIELGGENYNMVTKINGQAATGLGIKLATGANALDTAAAIKAKLAELQPFFP
QGLKVVYPYDTTPFVKISIHEVVKTLFEAIILVFLVMYLFLQNIRATLIPTIAVPVVLLGTFAILAAFGFSINTLTMFGMVLAIGLL
VDDAIVVVENVERVMVEEKLPPKEATQKSMSQIQGALVGIAMVLSAVFVPMAFFGGSTGAIYRQFSLTIVSAMALSVLVA
LILTPALCATLLKPASADHHEKKGGFFGWFNTLFEKSVGHYSNSVSGILRKTGRYLLLYVLIVGGMAILFLRLPTSFLPEEDQG
VFMTMVQLPAGATQTRTQQVLDQIQNYYLNQEKANVESVFTVNGFSFSGQGQNSGIVFISLKPWEERPGEENGVEAIV
ARATRAFSQIKDGLVFPFNLPAIIELGTATGFDFELIDQANLGHTELTKARNQLLGMIKEHPDLLVRVRPNGLEDTPQFKLD
VDQEKAQALGVSLSDINQTISTALGGTYVNDFIDHGRVKKVYVQADAPFRMLPGDINNLYVRSANGEMVPFSTFSSARW
IYGSPRLERYNGMPSMELLGEAAPGRSTGEAMALMESLAAKLPNGIGHDWTGMSYQERLSGNQAPALYAISLIVVFLCLA
ALYESWSIPFSVMLVVPLGVVGALLAASLRGLNNDVYFQVGLLTTIGLSAKNAILIVEFAKDLMEKEGRGLIEATLEASRMRL
RPILMTSLAFILGVMPLVISHGAGSGAQNAVGTGVMGGMLTATLLAIFFVPVFFVVVRRRFNRHHD

>AcrD_C_koseri
MANFFIDRPIFAWVLAILLCLTGTLAILSLPVEQYPDLAPPNVRITANYPGASAQTLENTVTQVIEQNMTGLDNLMYMSSQ
SSGTGQATVTLSFVAGTDPDEAVQQVQNQLQSAMRKLPQAVQNQGVTVRKTGDTNILTIAFVSTDGSMDKQDIADYV
ASNIQDPLSRVNGVGDIDAYGSQYSMRIWLDPAKLNSYQMTAKDVTDAIESQNAQIAVGQLGGTPSVDRQALNATINA
QSLLQTPEQFRDITLRVNQDGSEVKLGDVATVEMGAEKYDYLSRFNGNPASGLGVKLASGANEMETAKLVLNRLDELAE
YFPHGLEYKVAYETTSFVKASIIDVVKTLLEAIALVFLVMYLFLQNFRATLIPTIAVPVVLMGTFSVLYAFDYSINTLTMFAMVL
AIGLLVDDAIVVVENVERIMSEEGLSPREATRKSMGQIQGALVGIAMVLSAVFVPMAFFGGTTGAIYRQFSITIVAAMVLSV

LVAMILTPALCATLLKPLHKGEHHGQKGFFGWFNRTFNRNAERYEKGVAKILHRSLRWILIYVLLLGAMVVLFLRLPTSFLP
QEDRGMFITSVQLPSGSTQQQTLKVVQEVEKYYFTQEKDNVMSVFSTVGSGPGGNGQNVARMFVRLKDWDERNATT
GTSFAIIERATKAFNKIKEARVYASSPPAISGLGSSAGFDMELQDHAGAGHDALMAARDRLIELAAQDSALTRVRHNGLD
DSPQLQIDIDQRKAQALGVSIDDINDTLQTAWGSSYVNDFMDRGRVKKVYVQAAAKYRMLPDDINLWYVRNKDGGM
VPFSAFATSRWETGSPRLERYNGYSAVEIVGEAAPGVSTGTAMDVMESLVRQLPTGFGLEWTAMSYQERLSGAQAPALY
AISLLVVFLCLAALYESWSVPFSVMLVVPLGVIGALLATWMRGLENDVYFQVGLLTVIGLSAKNAILIVEFANEMNEKGHDL
LDATLHACRQRLRPILMTSLAFIFGVLPMAISNGAGSGSQHAVGTGVMGGMISATILAIYFVPLFFVLVRRRFPLKPKPE

>MexDYB_C_koseri
MFSRFFVRRPVFAWVIAILIMLAGILAIKTLPVAQYPDVAPPSVKISATYTGASAETLENSVTQVIEQQLTGLDNLLYFTSTSS
SDGSVSIDVTFEQGTDPDTAQVQVQNKVQQAESRLPSEVQQSGVTVVKSQSNFLLIAAVYDKSDRASSSDIADWLVSN
VQDPLARVEGVGSLQVFGAEYAMRIWLDPAKLASYSLMPSDVQSAIEAQNVQVSAGKIGALPSPDTQQLTATVRAQSR
LQTVEQFKKIIIKSQSDGAVVRIQDVARVEMGSEDYTAIAKLNGHPAAGVAVMLAPGANALDTATLVKDKVAEFKRDMP
QGYDVAYPKDSTEFIKISVEDVVQTLFEAIILVVCVMYLFLQNLRATLIPALAVPVVLLGTFGVLALFGYSINTLTLFAMVLAIG
LLVDDAIVVVENVERIMRDEGLPAREATEKSMGEIAGALVAIALVLSAVFLPMAFFGGSTGVIYRQFSVTIISAMLLSVVVAL
TLTPALCGSILSHTAPHKKGFFGIFNRFYSRTEQGYQRGVLHVLRRSVSVMGVYALLCGAMALAMWKLPGSFLPTEDQGE
IMVQYTLPAGATASRTAEVNRQVTDWFLNKEKANTDVIFTVDGFSFSGSGQNAGMAFVSLKNWSERKGEENTAQAIAL
RASKELGAIRDATLFAMTPPSVEGLGQSNGFSFELMANGATDRDTLLQMRNQLIAQANQSDILHAVRANDLPQMPQL
QVDIDSNKAVSLGLGLSDVTSTLSSAWGGTYVNDFIDRGRVKKVYIQGDSEFRSSPADLGKWFVRGSDNSMTPFSAFATT
RWEYGPESLVRYNGSAAYEIQGENASGFSSGAAMDKMEALANSLPAGTTWAWSGLSLQEKLASGQAMSLYAISILVVFL
CLAALYESWSVPFSVILVIPLGLLGAALAAWMRDLNNDVYFQVALLTTIGLSSKNAILIVEFAEAAVAEGYSLSRAALRAAQ
TRLRPIIMTSLAFIAGVTPLAIATGAGANSRVAIGTGIIGGTLTATLLAIFFVPLFFVLVKHLFTRKSRRQE

>AcrB_S_flexneri
MPNFFIDRPIFAWVIAIIIMLAGGLAILKLPVAQYPTIAPPAVTISRFYPGADAKTVQDTVTQVIEQNMTGIDNLMYMSSNS
DSTGTVQITLTFESGTDADIAQVQVQNKLQLAMPLLPQEVQQQGVSVEKSSSSFLMVVGVINTDGTMTQEDISDYVAA
NMKDAISRTSGVGDVQLFGSQYAMRIWMNPNELNKFQLTPVDVITAIKAQNAQVAAGQLGGTPPVKGQQLNASIIAQ
TRLTSTEEFGKILLKVNQDGSRVLLRDVAKIELGGENYDIIAEFNGQPASGLGIKLATGANALDTAAAIRAELAKMEPFFPSG
LKIVYPYDTTPFVKISIHEVVKTLVEAIILVFLVMYLFLQNFRATLIPTIAVPVVLLGTFAVLAAFGFSINTLTMFGMVLAIGLLVD
DAIVVVENVERVMAEEGLPPKEATRKSMGQIQGALVGIAMVLSAVFVPMAFFGGSTGAIYRQFSITIVSAMALSVLVALILT
PALCATMLKPIAKGDHGEGKKGFFGWLNRMFEKSTHHYTDSVGGILRSTGRYLVLYLIIVVGMAYLFVRLPSSFLPDEDQG
VFMTMVQLPAGATQERTQKVLNEVTHYYLTKEKNNVESVFAVNGFGFAGRGQNTGIAFVSLKDWADRPGEENKVEAIT
MRATRAFSQIKDAMVFAFNLPAIVELGTATGFDFELIDQAGLGHEKLTQARNQLLAEAAKHPDMLTSVRPNGLEDTPQFK
IDIDQEKAQALGVSINDINTTLGAAWGGSYVNDFIDRGRVKKVYVMSEAKYRMLPDDIGDWYVRAADGQMVPFSAFSS
SRWEYGSPRLERYNGLPSMEILGQAAPGKSTGEAMELMEQLASKLPTGVGYDWTGMSYQERLSGNQAPSLYAISLIVVFL
CLAALYESWSTPFSVMLVVPLGVIGALLAATFRGLTNDVYFQVGLLTTIGLSAKNAILIVEFAKDLMDKEGKGLIEATLDAVR
MRLRPILMTSLAFILGVMPLVISTGAGSGAQNAVGTGVMGGMVTATVLAIFFVPVFFVVVRRRFSRKNEDIEHSHTVDHH

>AcrD_S_flexneri
MANFFIDRPIFAWVLTILLCLTGTLAIFSLPVEQYPDLAPPNVRVTANYPGASAQTLENTVTQVIEQNMTGLDNLMYMSS
QSSGTGQASVTLSFKAGTDPDEAVQQVQNQLQSAMRKLPQAVQNQGVTVRKTGDTNILTIAFVSTDGSMDKQDIADY
VASNIQDPLSRVNGVGDIDAYGSQYSMRIWLDPAKLNSFQMTAKDVTDAIESQNAQIAVGQLGGTPSVDKQALNATIN
AQSLLQTPEQFRDITLRVNQDGSEVRLGDVATVEMGAEKYDYLSRFNGKPASGLGVKLASSANEMATAELVLNRLDELA
QYFPHGLEYKVAYETTSFVKASIEDVVKTLLEAIALVFLVMYLFLQNFRATLIPTIAVPVVLMGTFSVLYAFGYSVNTLTMFA
MVLAIGLLVDDAIVVVENVERIMSEEGLTPREATRKSMGQIQGALVGIAMVLSAVFVPMAFFGGTTGAIYRQFSITIVAAM
VLSVLVAMLLTPALCATLLKPLKKGEHHGQKGFFAWFNQMFNRNAERYEKGVAKILHRSLRWIVIYVLLLGGMVFLFLRLP
TSFLPLEDRGMFTTSVQLPSGSTQQQTLKVVEQIEKYYFTHEKDNIMSVFATVGSGPGGNGQNVARMFIRLKDWSERDS

KTGTSFAIIERATKAFNQIKEARVIASSPPAISGLGSSAGFDMELQDHAGAGHDALMAARNQLLALAAENPELTRVRHNG
LDDSPQLQIDIDQRKAQALGVDIDDINDTLQTAWGSSYVNDFMDRGRVKKVYVQAAAPYRMLPDDINLWYVRNKDG
GMVPFSAFATSRWETGSPRLERYNGYSAVEIVGEAAPGVSTGTAMDIMESLVKQLPNGFGLEWTAMSYQERLSGAQAP
ALYAISLLVVFLCLAALYESWSVPFSVMLVVPLGVIGALLATWMRGLENDVYFQVGLLTVIGLSAKNAILIVEFANEMNQK
GHDLFEATLHACRQRLRPILMTSLAFIFGVLPMATSTGAGSGGQHAVGTGVMGGMISATILAIYFVPLFFVLVRRRFPLKPR
PE

>MexB_A_vinelandii
MSGFFIDRPIFAWVIALVIMLAGGLSILGLPINQYPSIAPPAVSISVTYPGASAQTVQDTVVQVIEQQLNGIDNLRYITSDSN
SDGNMTITATFEPGTDADIAQVQVQNKLQLATPLLPQEVQQQGIRVTKSVRNFLLIVGFVSEDGSMDRNDLANYIVSNI
QDPLSRAKGVGDFQVFGSQYAMRIWLDPARLNNFQLTPVDVRNAVESQNVQVSSGQFGGLPAVPDQQLSATIIGKTRL
QTAEQFRDILLKVNGDGSQVRLDDVAEVGLGGENYSITSTYNGRPAAGMAIRLATDANALETAQAVRDTLAELEPFFPPG
VKVVYPYDTTPVISASIEEVVKTLGEAIVLVFLVMYLFLQNVRATLIPTLAVPVVLLGTFGVLAAFGFTINTLTMFAMVLAIGLL
VDDAIVVVENVERVMVEDGLPPREATRKSMGQIQGALVGIALVLSAVFVPMAFFGGSTGVIYRQFSITIVAAMALSVLVAL
IFTPALCATLLKPVSRGEHHRKRGFFGWFNRSFERSVRGYERGVRQVLGRKLPYLLLYLAIVALMVWLFSRIPTAFLPDEDQ
GVLYAQVQTPSGSSAERTQLVVDEMRNYLLTEEGHLVNSVFTVTGFNFAGRGQSSGLAFISLKPWAERTGEEDDVFALA
QRAQQRFAGFRDALAFAYAPPAVMELGNASGFDVFLQDRAGVGHEALSAARDQFLARAAKNPVLSRVRANGLRDEPQ
YRLLIDDELARAYGVPLADINSSISIAWGASYVNDFIDRGRVKKVYLQGEPKARMTPEDLGKWFVRNAAGTMVPFSAFAN
GEWTYGPPKLARYNGVSAMEILGEPAPGYSSGEAMAEVERIAEELPPGIGYAWTGLSYEERLSGSQAPALYALSLLVVFLCL
AALYESWSIPFSVMLVVPLGVIGALCFTSLRGLSNDVYFQVGLLTTVGLSAKNAILIVEFAKDLHEQGMALPEAAVEACRM
RLRPIIMTSLAFILGVVPLAVSSGAGAGAGSQHAIGTGVIGGMLTATVLAIFWVPLFYVLVSSLFGDRRPEAADRGEVP

>MexB2_A_vinelandii
MSRFFIDRPIFAWVIALVLMLGGGLAILKLSVTQYPNISPPAVQISVTYPGASAQTVQDTVVQVIEQQLNGLDGLRYISSVS
NADGSLQIIVTFEQGTNPDIAQVQVQNKLQLATPMLPEEVQRQGIRVVKYQVNFMMILGLVSTDGRMNGFDMGNYIA
SNLQDPISRVYGVGDFQVFGSQYAMRIWLDPEKLNNYQLTPQDVTSAISAQNVQVSSGQLGGLPAARGVQLNATVIGK
TRMRTADEFKEILLKVNRDGSQVRLKHVAEVGLSAENFSIASKYNGMPAAGMALRLAPGANLLESVAAVRAKLDELKPYL
PEGVEVVYPYETAPMVSASIEAVVHTLFEAIVLVFLVMYLFLQNFRATLIPTLAVPVVLLATFGVLFACGFTINVMTMFAMV
LAIGLLVDDAIVVVENVERIMVEEGLSPKEATRKSMGQISGALVGIALVISAVFLPMAFFGGSAGVIYRQFSITIISAMSFSVLI
ALIFTPALCATLLKPVAKGAHHEKRGFFGWFNRTFERSADGYQRGVGGITRRRWSFMLIYLLLVGGVAYLFLRLPGSFLPNE
DQGTMFVQIQLPPNSSAERTEAVLAETRDYLLKDEGDTVASVFSVNGFNFAGRGQSSGLLFINLKPFSERPRPEQDIFALA
QRAAVRYARIKDANVIPVVPPAVREMGNATGFDLFLQDYGGLGHEALMAARNQLLGMAAKEPTLTMVRPNGMNDEA
QYQVIIDDEKARALQVSIEDINETMSVAWGSSYVNDFIDRGRVKKVYVQGKQDSRVGPEDFDKWYVRNAEGGMVPFSA
FASGQWIYGSPKLERYNGISSVEVLGQPAPGYSTGDAMAVMNRLAAQLPSGIGIAYTGLSYEELAAGSQAPALYALSLLIV
FLCLAALYESWSIPFSVMLVVPLGVLGAVGATLLRGLSNDVFFQVGMLTTMGLAAKNAILIVEFAKDLYEHQGRSLMQAA
SEAARLRLRPIIMTSIAFALGVLPLATATGPGSGSQHSIGTGVLGGTLAATFLAIFFVPLFYVVVVGLFERQKDKAAETEGVM
A

>MexFQ_A_vinelandii
MSLSHYCIERPIFASVISIVITLAGAVAMFNLPVAQYPDITPPQITVSATYPGADAEVVANNVAAPIEQQVNGADDMIYMN
SSSSATGNMTLNVFFEIGTDPSLAQVDVQNRVNLALPQLPSAVQSQGIQVQKKSSAFMMVIAVYSPDERYDSTYIANYA
NLYILDAIKRIPGANQASIFGTPDYAMRIWLKPDRMAQLKITAADVQQAVANQNQQFAVGRIGQSPTGEAVEQSFAVTT
KGRLTEPEEFENIIIRAASGGAAIVRLRDVGRAELGQKDYSLRSTYQGKPATLMAVYQQPGANALDVSDAVRTTLAEMEKS
FPAGIEYKIVMDTTAFTRTSIAEVVHTFFEALVLVVAVVYLFLQSLRATLIPVLAVPVSIVGTFIGMTALGFSINMLTLFGMVLA
IGIVVDDAIVVIENVERNMQVHRLPPREAAKRAMDEVAGPVVAIVLVLCAVFVPVAFMGGITGQLYKQFAITIAISVVISGL
VALTLSPALAAILIKPAHGRKRGFFRWFERNFERMTEGYSRAVAFMIKRFLLALLLFAGMVVLTVLMAQRIPGAFLPPEDQG

YLLGAVIMPDAASLDRTGEVGRAVSDYFMRDEAVEGVAIVNGYSLLDSQNKNNAGTFFVGFKDFEERYKDDESIRTQSAP
AVIAAAGQAFSRVREGLVVPVNPPSIPGLGTTGGMEVWVQNKGDGSVGQLAGIVNDFVAKARQRPEIGAITATFNAASR
QLLADVDREKAETLGVPVEDVYSTMQTMFGSLYVSQFNKFSRLWQVILQAEPTYRLRVEDLQQIYVRNRGGEMIPLKALL
STRYVTGPDLLTRFNNFPAVKITANAAPGFSSGQALAALETLAAQNMSGDYALALSGEAFEEKKAGGTSAQVFVFGLIMV
FLILAAQYEKWTLPVGVLLAVPFALFGALLAILLRGLVNDVYFQIGLTMLVALAAKNAILIFEFAVLNREKGMSVFDAAMTA
ARERLRPIVMTSLAFILGCVPLAIAVGASENSRHSIGTGVIGGMLAATVIAVFFIPLFYYLIERLSEKSGTAKQEAPPAEPAPLG
GDLSGVPQRRREDD

>MexFQ2_A_vinelandii
MNFSQFFIHRPIFAAVLSLLTLIGGAIALFQLPISEYPEVVPPTVVVRANFPGANPKVIGETVASPLEQAIVGVEGMLYMSSQ
ATSDGKLTLTITFALGTDLDNAQVQVQNRVTRTMPTLPTEVQRLGVTVDKASPDLTMVVHLTSPDERYDMLYLSNYATL
NVKDELARLDGVGDVQLFGLGNYSLRVWLDPHRVASRGLTAMDVVDAIREQNRQVAAGSLGAPPAPGDTGFQLSINT
QGRLVSEEEFENIIVRAGEDGEITRLKDIARIELGSSQYALRSQLNNRPAVAMPVFQRPGSNAIDISDAVRARMAELKQSFP
QGVDYSIVYDPTVFVRGSIEAVVHTLFEAIVLVVLVVILFLQTWRASIIPLAAVPVSLIGTFAVMHLFGFSLNALSLFGLVLAIGI
VVDDAIVVVENVERNIALGKPPLEATRQAMQEVTGPIVATALVLCAVFVPTAFISGLTGQFYQQFALTIAISTVISAFNSLTL
SPALAAVLLKDHHAPKDRFSRVLDRLLGGWLFGPFNRLFERASHGYVATVRHVLRGSAIALLVYGGLMGLTWLGFSSTPT
GFVPPQDKQYLVAFAQLPDAATLDRTDAVIQRMSEIAGRHPGVADTVAFPGLSINGFTNSPNSGIVFTPLKSFEERQDPSL
SADAIAAELNRRFAEIQDAYIAIFPPPPVQGLGTIGGFRVQVQDRGGLGYEELYRQVQNVIAKSRTVPELAGLFTSYQVNV
PQVDADIDREKAKTHGVAISDIFDTLQVYLGSLYANDFNRFGRTYQVNVQAEQRFRLEPAQIGQLKVRNNRGEMVPLST
FVRIEGSAGPDRVMHYNGFLTAEINGAAAPGYSSGQAEAAMERLLREELPGGMTFEWTELTYQQILAGNTAIFVFPLCVLL
AFLVLAAQYESWGLPLAVILIVPMTLLSAIVGVRIAGGDNNVFTQIGLIVLVGLACKNAILIVEFAKDRQAEGLDRIAAVLEA
CRLRLRPILMTSIAFIMGVVPLVLSSGAGAEMRHAMGVAVFSGMIGVTLFGLLLTPVFFVLVRAFVERREARRNRQALEAQ
G

>AcrF_P_asymbiotica
MPKFFIDRPIFAWVIAIITMLAGLLAIMKLPVAQYPTIAPPAISISATYPGADATTVQNTVTQVIEQNMNGIDNLVYMSSNS
DSAGNMNITLTFEAGTDPDIAQVQVQNKLQLAMPLLPQEVQQQGIHVDKSTNAFLMVAGFTSEDGSMSQEDIADYVG
ANVKDPLSRVTGVGDAMLFGNQYAMRIWLNPDKLINYKMTTLDVINAIKAQNNQIAAGQLGGTPPVPGQRLNASIIAQ
TRLTSPEEFSKILLRVNTDGSQVRLKDVAKVELGSESYNLIGRFNGKPAAGIGIKLGTGANALDTSKAVKEALKNMEPFFPH
GLKIVYPYDTTPFVKISIHEVVKTLIEAIMLVFVVMYLFLQNFRATLIPTIAVPVVLLGTFAILAAFGYSINTLTMFAMVLAIGLL
VDDAIVVVENVERVMQEEGLSPKEATKKSMGQIQSALVGIAMVLSAVFVPMAFFGGSTGAIYRQFSITIVSAMVLSVLVAL
ILTPALCASMLKPIAKGSHGKQTGIFGWFNRLFDKSTHHYTDSVGRILRNTGRYLVIYVLLVAGMAVMFTRLPSSFLPEEDQ
GVLLTMVQLPAGATQEQTQKVLDEVNHYFSTEEKDRVVSVFTVSGFGFSGQGQNTGLAFMSLKDWSERPGAENKVPAI
VARASAHFQKIKEGMVFAFNVPAIVELGSATGFDFELIDKANLGHDKLTEARNQLLGMIQQRPDMLVGVRPNGQEDTP
QYRVRIDQEKAETLGVSIADINSTLSTMFGSHYVNDFIDRGRVKKVYIQSEAPYRMLPSDIYKLYVRNKQGEMVPFSAFAD
MSKEPWTYGSPRLERYNGLPSMEILGEAAPGKSTGDAMALMEELASQLPNGIGFDWTGMSYQERLSGNQAPALYALSLI
VVFLCLAALYESWSIPFSVMLVVPLGIVGALLATTFRGLDNDVYFKVGLLTTIGLSAKNAILIVEFAKDLMEKEGKGLIEATLD
AVRMRLRPILMTSLAFMLGVIPLVLSNGAGSGSQNAVGTGVLGGMIAATSLAIFFVPVFFVVIKRRFSKKVEEIEPASESH

>AcrB_Y_enterocolitica
MAKFFIDRPIFAWVIAIIIMLAGSLAIMKLPVAQYPTIAPPAISISANYPGADATTVQNTVTQVIEQNMNGIDNLLYMSSSSD
SSGNVQLTLTFNSGTDPDIAQVQVQNKLQLAMPLLPQEVQQQGVSVEKSSSSFLMVAGFISEDGTMKQEDIADYVGSN
VKDPISRTAGVGDVQLFGSQYAMRIWMDPHKLNNYGLTPVDVINAIKVQNNQVAAGQLGGTPPVPGQELNSSIIAQTR
LTNAEEFSQIMLKVNTDGSQVRLKDVAIVQLGAENYNIIARYNGKPAAGIGIKLATGANALNTSAAVKAELAKLQPFFPAG
LKVVYPYDTTPFVKISINEVVKTLVEAIILVFLVMYLFLQNFRATLIPTIAVPVVLLGTFAILSAFGYSINTLTMFGMVLAIGLLVD
DAIVVVENVERVMQEEGLPPKEATKKSMEQIQGALVGIAMVLSAVFIPMAFFGGATGAIYRQFSITIVSAMVLSVLVALILT
PALCATMLKPIAKGEHGPKTGFFGWFNRMFEKSTHHYTDSVGNILRSTGRYLVIYLAIVIGMGVLFLRLPTSFLPEEDQGVF

LTMVQMPAGATQERTQKVLDQVTDYYLDKEKDVVNSVFTVNGFGFSGQGQNTGLAFVSLKNWDERPGEQNKVPAIV
ARASAAFSQIKDGLVFAFNLPAIVELGTATGFDFQLIDQGDVGHQKLTEARNQLLGMAAQHPDMLVGMRPNGLEDTPQ
FKVEVDQEKAQALGVAISDINTTLGSAMGGSYVNDFIDRGRVKKVYVQADAPFRMLPSDIDKWYVRNNAGQMVSFATF
SSAKWEYGSPRLERYNGLPSMEILGQAAPGKSTGEAMDLMQELASKLPSGIGYDWTGMSYQERLSGNQAPALYAISLIVV
FLCLAALYESWSIPFSVMLVVPLGVVGALLAASLRGLNNDVYFQVGLLTTIGLSAKNAILIVEFAKDLMDKEGKGLVESTLEA
VRMRLRPILMTSLAFILGVLPLVISSGAGSGAQNAVGTGVMGGMITATVLAIFFVPVFFVVVRRRFSRKNEDVEHAHAVD
HKVK

>AcrD_Y_enterocolitica
MANFFIDRPIFAWVLAIILCLTGALAISTLPVEQYPNLAPPNVRISASYPGASAQTLENTVTQVIEQSMTGLDNLLYMSSQS
SNSGSASITLTFQAGTNPNEAMQQVQNQLQSAIKKLPQDVQQQGVSVSKSGDNTLMMVAFVSTDGSMDKQDIADYV
ASNLQDPLSRIEGVGSIDAFGSQYAMRIWLDPNKLNNYQLTTQDIVTAIQSQNSQIAVGQLGGTPSVDNQALNATINA
QSQLQTPEQFREITLRVNQDGSLVTLGDVAKIEMGAEKYDYLSRFNGQPASGMSIKLASGANELQTDELVKARIAELTPFF
PHGLEAKIAYETTPFVKASIKDVVKTLLEAILLVFLVMYLFLQNFRATLIPTIAVPVVLLGTFAILSAFGFSINTLTMFAIVLAIGLL
VDDAIVVVENVERVMSEEGLDPREATRKSMSQIQGALVGIALVLSAVFIPMAFFGGTTGAIYRQFSVTIVSAMVLSVLVALI
LTPALCATMLKPIKPGHHHAKRGFFGWFNRMFDRNAHRYERGVAQVLHHSVRYMLLYLLLLGGLALLFIKLPTSFLPLEDR
GVFMAQVQLPVGSTQQQTLRVVEKVENYFLTAEKNNVLSVFSTVGSGPGGNGQNVARLFIRLSDWEQRKDSNNSSFAII
ERATKVFNKIAEAKVSVSSPPAISGLGGSSGFDMELQDHGGLGHDKLMAARDQLLQMASQDPALTRVRHNGLDDSPQL
QIDIDQRKAQALGVSLDDINNTLKTAWGSTYVNDFVDRGRVKKVYVQSEATARMLPEDVNKWYVRNKNGGMVPFSAF
STTRWEYGSPRLERYNGYSALEIVGEAAPGVSTGTAMDVMEGLVKQLPNGFGLEWTGMSYQERLSGSQAPALYAISLLV
VFLCLAALYESWSIPFSVMLVVPLGVIGAVAATWMRGLENDVYFQVGLLTIIGLSAKNAILIVEFASELNNKGKDLVEATLEA
SRLRLRPILMTSLAFIFGVLPMAISQGAGSGSQHAVGTGVMGGMISATVLAIFFVPLFFVLVRRRFPGRPAHTKNSDTQ

>MexDYB_Y_enterocolitica
MFARFFIHRPVFAWVIAIVIMLGGAVAMQTLPIAQYPDVAPPSVSIKATYTGASAETLENSVTQVIEQELTGLDGLLYFSSSS
SSDGKANIVATFKQGTNADTAQVQVQNKVQQALTRLPTEVQSQGVTVTKSQTNFLLIMALYDEKDKHTGTDIADYMVS
NLQDPLARLEGVGSVQVFGSQYAMRIWLNPTKLAAYSLMPSDIQTAIEAQNTQVSAGKIGALPSGKEQQLTATVMAQS
RLKTPEQFNNIIVKSDSTGAVVRLSDVARVELGNEDYSVTTRLNGHPAAGIAVMLAPGANALATAERVKAKAAEFELSLPD
GYKIAYPKDSTDFIKVSVEEVVKTLIEAILLVVIVMYVFLQNIRATLIPAIAVPVVLLGTFGVLAIFGYSINTLTLFGMVLSIGLLV
DDAIVVVENVERVMREDNLPPREATEKSMGEISSALVGIALVLSAVFLPMAFFGGATGVIYRQFSITIVSAMALSVLVALTLT
PALCAAFLKPNHKPPSNTGFFGWFNRHYDRMQTRYESMVGNVIHRSLRYLLIYAVLIVVMGILFIRLPTGFLPTEDQGDV
MVQYTLPAGATSGRTMEVSKAVENYFLTQEKDNTKAVFTISGFGFSGSGQNAGMAFIALKNWSERPGSENTATAIADRA
MKALSIIRDAQIFSMTPPAVDGLGQSNGFTFELQAAGDTSREQLLTLRDQLIGNASKDPILTSVRANTLQQMPQLQVDID
NDKAAALGLSISDVNATLSAAWGGTYINDFIDRGRVKKVYMQGDVDSRSKPEDINQWFVRGSSDAMTSFSAFATTRWIY
GPETLSRYNGQASYEIQGQAASGSSSGTAMDRMEKLAAELPGTSYSWSGLSYQERLASGQALSLYAISILVVFLCLAALYE
SWSVPFSVMMVIPLGIIGAVAAATLRGLENDIYFQVALLTTLGLASKNAILIVEFAEAAYLRGEPLVAAALQGAATRLRPILM
TSLAFVAGVMPLAISTGAGANSRISIGSGIIGGTLTATALAVFFVPLFFVLIRRVFSGKSKKTTEHKGV

>MexFQ_Y_enterocolitica
MLHFFIRRPKFAIVIALVITIVGWVSLHVIPVEQYPDITPPVVSVSAVYPGASARDVAQAIASPLEAQVNGVSNMLYMESTS
GNNGSYQLSITFASGTDPDMAAVEVQNRISQVSAQLPAEVNENGISVRKRASNLLLGVSVFSPKQTHDALFVSNYTSIQL
RDAIARINGVGDVQVFGARDYSMRVWLNPQRMESLNVSVQDIIAALQQQNVQAAAGQIGSSPSTPDQQQTLTISGQG
RLSDPQEFANVIIRSNPQGGMIRLGDVARVALGAQNYQVSAAQNQTESAFLVVYPVPGANALNVANGVRDEMARLSA
SFPTDLTYEINYDSTLPVTATLHEIALSLTLTLIVVLAVVYLFLQNMRATFIVALTIPVSLLGTFAVLYVFGYSANTLSLFAIILALT
IVVDDAIVVVENVERLLSNDPDLTPAQATKQAMSQIAGPIIATTLVLMAVFVPIAILPGIVGELYRQFAVTLSAAVILSSINAL
TLSPALCAVLLKCRTLSTSGVFGGINRGLDSARDGYVSLTGRISSRAVFSIAALLLVGLATWWGYTRLPTSFLPEEDQGYFFV
SLQLPDGASLNRTQTVMDQMYQQVTANDAVEDVIKITGFSLLSGNNSPNAGFAIVMLKPWGQRPHIDQVLANIQANL

AAIPSAMIMAVNPPAIAGLGSASGFDLRIQALLGQTPQELAQVSQGVIFAANQDPKLSRVFTTFSASVPEINLSIDRDRAAL
LQVPVSRIFQTLQTSLGGMNAGDFTLNNRMFRVQLQNDMDFRQRSAQINSLNVRSDNGVLVSLANLVTLTPSVGAPFI
SNFNQFPSVAISGSAADGASSGQAMAAMEVLLAQNLPQGYSYSWSGMSWQEQQTGGQVVFIYLAALVFAYLFLVAQY
ESWSIPLVVILSVVFAVGGAVAGLSAMGFANDVYAQIGLVLLIGLAAKNAILIVEFSKARREEGATIAQAAQDGAKQRFRA
VMMTAISFILGVMPLVFASGAGAMSRQIIGITVFGGMLMATAVGILFIPALYLHIQRLREWTKTRKPPADKDPQH

>AcrB_A_pleuropneumoniae
MKFTDIFIKRPVLAVCISLLITILGLQSINKLQVREYPEMTISIVTVKVNYSGADASLMQALVTSQIEEAVAQADNIDYVTSSS
GPSSSTTTIKMKLNTNPQMALADISAKVNAIRANLPSGIDDPSISVSSGSNDALMYIRFVSDELSTPQITDYINRVVKPQFFT
VNGVSSVDIFGAANFGLRIWLDPDKMAGNNLSGAAVLSALSANNLNTAAGNANGYYTVYKNKVESSTPSVEELENVTIS
TTSDGRIVKLKDIAKVELDKYQDASRAIADGKEAVVLAVSAATTANSLTVAKDIYPVFDLITKNLPSSVNGQIMYDKTVAIN
SSINEVIKTIGEATVIVLAVIILFLGSLRAMIVPIITIPISLIGVLFFLQMFGFSINLLTLLALVLAIGLVVDDAIVVLENVERHVKEGK
TPFDAAIIGTREIALPVISMTITLAAVYSPMALMEGVTGSLFKEFALTLAGAVFISGIAALTLSPMMSSRVLKEHNEENESRFS
KCINTMLDKMTACYTNMLRGVMSVRWLVVIFAMSIFAVLPTLLTSLSSEVAPTEDRGFVVGIGNGPSNTNLDYTQAATEK
FNEDVGKIPEVASMMTVSGFNGANSALSIISLKDWSERERERKPISADVSAIAKNVVGMDINAISFPEISTGENGLPFSLVIT
TADNYVKLADVANDFLKKAQASGQFFFSSLDLKFDTVTMKWKFDREKMGTYGVTMQQVSGTLGAYLSGAIITRVDIDSR
AYPIVSQAVRQDRLNPQDLVNYYVTTASGESVPLSSFVSLELSTEPAALPRMNQLNSATISGVVSSSIGDAVNWAKAELD
RSLPQGYQYDFKSEARQYVQEGNAMAMTFALAVVIIYLVLAIQFESWRDPMVILVSVPLALSGALVVLNVLGIAGIAGATL
NIYSQVGLVTLVGLITKHGILMCEVAKEEQLHNGLSRFDAIVHSATIRLRPIMMTTAAMVAGLIPLLFAMGAGAIARFSMG
MVIVAGLSIGTLFTLFVLPVIYTFLGQEHKPLREFDEEKYAKASVEKIEHTGM

>AcrB_S_proteamaculans
MAKFFIDRPIFAWVIAIIVMLAGVLSIMKLPIAQYPTIAPPAVSISANYPGADAKTVQDTVTQIIEQNMNGIDNLMYMSSTS
DSSGSVTITLTFNSGTDPDIAQVQVQNKLSLATPLLPQEVQQQGLKVEKSSSSFLMVAGFISDDPSMTQDDIADYVASNI
KDPISRSSGVGEVQLFGAQYAMRVWLDPNKLNNFQLTTTDVTSAITEQNNQIAAGQLGGMPPVPGQQLNASIIAQTRL
TSPEEFGKILLKVNADGSQVRLSDVAHIERGAESYAVTARYNGKPAAGLGIKLATGANALNTAQGVKDALTKMQPFFPQG
MKVVYPYDTTPFVKISINEVVKTLIEAIILVFLVMYLFLQNFRATLIPTIAVPVVLLGTFAILAAFGFSINTLTMFGMVLAIGLLV
DDAIVVVENVERVMSEEGLPPKEATKKSMEQIQGALVGIAMVLSAVFVPMAFFGGSTGAIYRQFSITIVSAMALSVLVALIL
TPALCATLLKPVAKGDHGVKTGFFGWFNRMFEKSTHHYTDSVANILRSTGRYLVIYLLIVVGMALLFLRLPSSFLPDEDQGI
LLTMVQLPAGATEARTSKVLEEVTDYFMTKEKDNVVSVFTVAGFGFNGNGQNNGLSFVSLKDWSERKGEENKVPAIAGR
AMGAFSQIKDGLVFPFNLPAIIELGTATGFDFQLIDQGGLGHEKLTEARNQLLGMAAKHPDMLVGMRPNGLEDTPQFKLI
IDQEKAKALGVSITTINSTLSTALGGSYVNDFIDRGRVKKVYVQADAPFRMLPEDINKWFVRGTSGQMVPFSAFSSAKWE
YGSPRLERYNGLPSMEILGQATPGKSTGEAMNLMEELASKLPSGIGYDWTGMSYQERLSGNQAPALYAISILVVFLCLAAL
YESWSIPFSVMLVLPLGVIGALLAATMRGLNNDVYFQVGLLTTIGLSAKNAILIVEFAKDLMDKEGKGLIEATLEAVRMRLR
PILMTSLAFILGVLPLVISSGAGSGAQNAVGTGVMGGMITATVLAIFFVPVFFVVVRRRFSKKSEDVEHSHPVDHH

>AcrF_S_proteamaculans
MSNFFINRPIFAWVIAIIVMMAGALSIMKLPIAQYPTIAPPAVSISATYPGADAKTVQDTVTQVIEQNMNGLDGLMYMSS
NSDAAGNATVTLTFATGTDPDIAQVQVQNKLQLAMPLLPQTVQDQGINVQKASSSFLMVAAFTSDSSAMTQADLADY
AVANIKDPISRTSGVGQVQLFGAQYAMRIWLDPNKLDNYQLTSEDVVRAIKAQNNQIAGGQLGALPAVPGQQLNASII
AQTRLSSPQQFGNILLRVNQDGSQVHLRDVARIALGSENYTTSAEYNGKPAAGLGVKLATGANALDTANAVKAEIGRLE
PYFPAGMHVVYPYDTTPFVKISIGEVVKTLIEAIILVFLVMYLFLQNLRATLIPTIAVPVVLLGTFAILAAFGFSINTLTMFGMVL
AIGLLVDDAIVVVENVERVMTEEGLPPKEATRKSMAQIQGALVGIAMVLSAVFIPMAFFGGSTGAIYRQFSVTIVSAMVLS
VLVAMILTPALCVTLLKPGVHAPKTGFFAWFNRLFERSTQHYQGSIGNILNHTGRMLLIYLLIVIGMALLFIRLPTAFLPDED
QGVFMNMIQLPAGATQQRTEAVLGQVTHYYLNKEKANVNSVFTVAGFGFNGNGQNNGLAFVSLKDWSQRPGEKNK
VEAIIKRANMAFAHTIKQGIAFAFNLPAITDLGTATGFDFELIDNGGLGHAALTKARNQLLFMAAKYPNVATKIRPNGLEDT
PQFKVDVDQAKAEALGVSVDDINQTLSTAWGGTYVNDFIDRGRVKKVYVQADAPYRMLPDDLNQWYVKGNRGQMV

PFSAFASAHWEYGSPRLERYNGLPSMEILGEPAPGRSSGQAMMLMEKLAAKLPKGIGYDWTGMSYQERLSGNQAPALY
AISLVVVFLCLAALYESWSIPFAVMLVVPLGVVGALLGATLRGLNNDVYFQVGLLTTIGLSAKNAILIVEFAKDLMEKEGKPLI
AATLEATRMRLRPILMTSLAFILGVLPLVLSSGAGSGAQNAVGTGVMFGMISATLLAIFLVPVFFVVVQRRFGRK

>MexDYB_S_proteamaculans
MAQFFIRRPVFAWVIAIIIMLCGLMSINSLPISQYPDVSPPQISISATYPGADAETLENSVTQVIEQQLTGLDGLLYFSATSSS
DGSVSINVTFDQGTDPDIAQVQVQNKVQQAESRLPAEVTAQGVTVKKSQSSFLLIVGLYDETDKASMADISDYLVSNIQ
DPLSRIDGVGDVQVFGSEYAMRIWLNPTKMASFSLMPSDVETAIEAQNTQVSAGKVGDLPSGADQQLTATVKAQSRLQ
TPEQFRNIILKSDSSGALVRLGDVARVELGNEDYSSSARLNGHPASGIAVKLAAGANALNTAKLVKAKVAEYQTSMPHGY
KVAYPKDSTDFINISIEEVVKTLIEAVVLVVIVMFVFLQNLRTTLIPTITVPVVLLGTFGVLAAFGYSINTLTLFGMVLAIGLLVD
DAIVVVENVERVMREEKLPPREATEKSMKEITGALVGIAMVLSAVFLPMAFFGGSTGVIYRQFSVTIVASMVLSVILALTLAP
ALCATLLKPAHEELTNKGLLGKFNRGYNRLQEKYADKVGRVIHSPMRYLLLYGLLLIAAAVMYMRLPTGFLPNEDQGVVM
VQYTLPAGATNARTSAVSDEVKDYFLNDEKANVSTIFTINGFGFSGSGQNAGMAFVSLKDWSLRSGSANTADAIAKRAM
AHFAKIRDAQVFSMSPPAIDGFGQSDGFTFELQAKGATTRAELQVMRDQIIAAAAKNPSLSAVRANTLPDTPQLQVEIDN
DKLQALGLSNSDVSSTLTNAFGSTYINDFIDRNRVKKVYMQGDVEYRSKPEDLEKWFVRGTSSSGTTSMTPFSAFASSHW
IYGPENLSRYNGLSSFEITGEGAPGTSSGTAMDEMAKLAAKFSSASSFSWSGLSYQERLTSGQAGSLYAISLVVVFLCLAAL
YESWSIPFAVMMVVPMGVVGSLLAITLRGLENDIYFQVALLTIIGLSAKNAILIVEFAEEKYLRGETLIAAAVEASRMRLRPIL
MTSLAFSAGVLPLAVSSGAGANSRIAIGTGIIGGTITATILAIFFVPLFYVLVRRMFSRKTTVAKPLENTAPQAGE

>MexFQ_S_proteamaculans
MDFSRFFIDRPIFAAVLSILIFVGGVIAIPLLPISEYPDVVPPSVQVRAQYPGANPKEIAETVATPLEEAINGVENMMYMKSVA
GSDGVLVTTVTFRPGTDPDQAQVQVQNRVAQAEARLPEDVRRQGVTTQKQSPALTLVVHLVSPSGKYDSLYLRNYATL
KVKDELARLPGIGQVQIFGAGEYAMRIWLDPNKVASRGLTASDVVSAMQEQNVQVSAGQLGAEPMPTRSDYLLSINAQ
GRLQTEEEFGNIILKSGDNGEVVRLRDVARIEMGSGSYALRAQLNNKDAVGIGIFQAPGANAIELSNAVRAKMAELSTRFP
DGMSWQAPYDPTVFVRDSISSVVDTLLEAVLLVVLVVILFLQTWRASIIPLLAVPISIVGTFAALYLLGFSLNTLSLFGLVLAIGI
VVDDAIVVVENVERNIEEGLSPLAAAHQAMREVSGPIIAIAVVLCAVFVPMAFLSGVTGQFYKQFAVTIAISTVISAINSLTLS
PALAALLLKPHGAPKDMPSRLIDRLFGWLFRPFNRFFASSSQRYQRGVSRALGKRGAVFVVYVLLLCAAGVMFKTVPGGFI
PTQDKLYLIGGVKMPEGASLERTDAVIRKMSAIGLSVDGVTDAVSFPGLNALQFTNTPNTGTVFFALESLSTRTRSAAQIN
AEINAKISQIQEGFAFSIMPPPILGIGQGSGYSLYIQDRAGLGYGALQTAINTMSGAIMQTPGMGFPISSYQANVPQLDAKI
DRDKAKAQGVPLNALFSTLQTYLGSSYINDFNRYGRTWKVMAQADGQFRDSVEDIANLRTRNDKGEMVPIGSMVSISTT
YGPDPVIRYNGFPAADLIGDADPRVLSSSQAMSELTVMASKLLPNGMNIQWTDLSYQQSTQGNAAIIVFPVAVLLAFLAL
AALYESWTLPLAVILIVPMTMLSALFGVWLTGGDNNVFVQVGLVVLMGLACKNAILIVEFARELELQGKSIMEAALEACRL
RLRPIVMTSIAFIAGTIPLILGHGAGAEVRGVTGITVFSGMLGVTLFGLFLTPVFYVTLRRMVTRKTRQPVAE

>AcrB_P_ingrahamii
MKFTDIFIRRPVLAISISLLLVLLGLQALNKMQVREYPTMTNTVVNVSTSYYGANADLIQGFITQPIEQAIAQANNIDFITSQ
SYMGNSKISVFMNLNTDPSGAVADILAKVNSVRSQLPSEAEDPSIDMSTGSSTSVIYISFSSEQLNSSEITDYLERIIKPQLFSI
NGVAKVNLYGGTKFALRIWLDPERMAGFGLTTADVVQILQANNIQSAAGQTNSYYTLLNSNVGTQVETGAELENLVIAT
SGDGKVIRLQDIARVSLEKSHDVYRAMANGEDAVVVAIDAAPTANPLDITAGVRDMLPALAKNNPSSINMKVLYDSTIAI
EESISEVIKTIAEAGFIVLLVMILFLGSWRAVIIPIITIPLSLIGVVVVMQLFGFTLNLMTLLAMVLAIGLVVDDAIVVVENIDRHIK
AGEDPFRAAILGTREIAVPVISMTITLAAVYAPIALMGGITGSLFTEFALTLAGAVFVSGIVALTLSPMMCSKLLKNNQKPNR
FERNVESVLSSITRGYGRLLDKLMTRRPAVILYACLVFISLPFLFNSIPSELAPKEDKGALMVMAKAPANANLDYIENSMRIIT
GELAKQPEVTSSLAMSGIPNSNQAFGIAIMKPWSQRDKSPKELSASLMPKFNSVAAVATTAFQMPELPGSSGGLPVQFVI
TTGNQFESLVSIASDVLAATQASSNFVYSTLDLAFDSATMNIQIDRDKAGAYGVTMKDIGFTLGTLLSDGYLNRIDLNGRS
YEVIAQVERKYRLSPQKLGNFYVRAKNGDMIPLSSLITIDVTAEPRSLAHFNQLNSATVGAVLSPGVAMGDAIDFFEGIAET
KLPQGYSHDYLGEARQFVTEGSALYMTFVLAILIIFLVLASQFESIRDPLVILLTVPLAISGALVALAWGAATMNIYTQVGLITL

VGLITKHGILIAEVAKEEQLHHGLNRIDAVKKAATVRLRPILMTTAAMVAGLIPLLFATGAGAVSRFGIGIVIVAGLSIGTLFTL
FILPVIYSFIASKHQPRQEFDETLEHKVKDHEH

>AcrB_P_multocida
MRFTDIFIRRPVLAVSISLLIIILGLQAIAKLAVREYPKMTTTVINVSTVYPGADASLIQAFITSKLEEAVAQADNIDYLSSKSSP
NNSLITIKMKLNTEPNGALADVLAKVNSVRSQLPRGIDDPVITSSTGGTGMMYIQFSSTKLDASQVTDYIERVIKPQFFTVE
GVAEVQIFGASDYALRIWLDPAKMAAQNLSAMAVRNALSSNNVQTAAGNDNGFYVTYKNKVETTTKSVEELGNLIISSQ
GDKLVRLRDIADVELNKSSDSSRAVANGFESVVLAINPTSSANPLVVAENVRPLYEQIKQNLPDSMQAEILYDTTIAINSSID
EVIKTILEATIIVLVVITLFIGSLRAILIPVITIPISLIGVIMLLQAFDFSINLMTLLALILAIGLVVDDAIVVLENVDRHIKLGETPFRA
AIIGTREIAVPVISMTIALMAVYSPMALMDGITGSLFKEFALTLAGAVFISGIVALTLSPMMSSKLLKPHTDPSKLEQCIEKML
TTINQGYNYALNLVMASRRCMLAFAAVIFATLPFLFNGLASELTPAEDKGAFMVIGRAPSNVNVDYVQEAMHEYEQILKD
TPEVDFSMVISGAMGSNQSFSVVTLKDWKERTRSQAEILAELNKKSAHIPEVALTGFNFPEINTGEQGPPIGFVISTASGYE
DLANVAENFLQEMKKSGHFVYTALDLKFDTAQMRIKVDKEKAGTYGVTMQQISATLGSFLSAATIERVDIDGRAYRIIMQ
VKRGERLSPESLNNYYVTASNGKSVPLSSFLTMTLESQPTSLPRFSQLNSAVISAVPLPTKSTGEAIAWLKMAAKEYLPQGY
NYDFTGEARQLVQEGNALAVTFVLAVVIIFLVLAIQFESIRDPLVIMISVPLAVSGALLMLNFLGFFRIPGTSLNIYSQVGLITL
VGLITKHGILMCEVAKEEQLQHGKNRIEAITEAAKVRLRPILMTTAAMIAGLIPLLYASGAGAVSRFSIGIVIVSGLAIGTLFTLF
VLPVIYSYIATEHKPLPEFDESIKPLTDNSAH

>AcrB_M_succiniciproducens
MKFTDIFIKRPVMAIAISMLIVILGLQAISKLAVREYPKMTTTVITVSTTYAGADAGLIQAFVTSKLEEAIAQADNIDYLSSTSA
PSSSTITVKMKLNTDPASALADVLSKVQSVRSELPSGIEDPTLTSSTGGSGIMYISFRSDKLHPSQVTDYIERVVKPQLFTVEG
VAKVQVYGAAEYAMRIWLDPQKLAGQNLSATQVTTALSNNNVQTAAGSDKGYFNIYRNKVETTTNTVEDLGNLIVYSD
GDKLVRLRDVADVELNKESDDTRAAANGSDAVVLSIEPTSSANPLTVADNIKPLYETIKKNLPDSIESNILYDRTVAINSSIN
DVIHTIIEAVVIVLVVIMMFIGSLRAIFIPIVTIPISLIGVIFMLQMFDFSINLMTLLALILAIGLVVDDAIVVLENVDRHIKEGETPF
RAAIIGTREIAVPVISMTIALVAVYSPMALMGGITGTLFKEFALTLAGAVFISGIIALTLSPMMSAKILKHESSKFEEKVNRTLSK
LTTGYTYILGLVMQARKAILLFAVIIFATLPILFSSLSSELTPAEDKGGFLGMVTAPSNVNVDYVQQATKPYEEILNNTPEKQY
SQVIAGAPNTNQALVITTLKDWAERSRSQAEVMAELTKKAAAIPEVSISAFAFPEIETGEQGPPVVFVLSSPGSTKELAQTA
ETFLDKIRKSGKFVYSNLTLKYDVAQMRIQVDKEKAGTYGITMQQIASTLGSYLSEATITRVDIDGRAYKVISQVKRENRLSP
ESLKNYYISASNGQSVPLSSLLTVELEPQPYSLPRFSQLNSAEIQLVPSPTTTTGDAIAWLKDAAQDLPQGYSYDWKGEAR
QLVQEGNSLATTFILAVLIIFLVLAIQFESIRDPFVILVSVPLAISGALLTLNLLSFLGVTGVTLNIYSEVGLITLVGLITKHGILMCE
VAKEEQLNHGKTKMEAIMTAAQLRLRPILMTTAAMIAGLIPLLYASGAGAVMRFSMGVVVVAGLAIGTLFTLFVLPVIYTYI
GSNHKPLPEFDENAPRIGSSH

>AcrB_P_profundum
MRFTDVFIKRPVLAVSISFLIALLGLQAIFKMQVREYPDMTNTVVTVTTSYYGASANLIQGFITQPLEQAIAQADNIDYMTS
SSVLGSSTITVTMKLNTDPNGALADILAKTNSVRSQLPKESEDPTVTMSTGSTTAVMYIGFTSDELVSSQITDYLERVVTPQ
LFTVNGVSKVDLYGGLKYALRVWLDPPKMAAYNLTATDVMSVLNANNYQSATGQAVGEFVLYNGTADTQVSNTDELA
KLVVSTKNGQVIRLGDIAKVSLEKSHDIYRASANGEEAVVAAINAAPSANPINIAADVLTLLPELKRNMPSNIKMNVMYDS
TIAINESIQEVIKTIVEAALIVLVVITLFLGSFRAVLIPIITIPLSLIGVALVMQMFGFSWNLMTLLAMVLAIGLVVDDAIVVLENV
DRHIKLGEPPFRAAIIGTREIAVPVIAMTLTLGAVYAPIAMMGGITGSLFKEFALTLAGAVFVSGIIALTLSPMMCSKMLKANE
KPSKFESTVHSFLDRMTNRYEKMLGGVMTMRPVVIVFAAIVLVSLPLLFKFIPSELAPSEDKGVLMMMGTAPSNANLDYIE
NTMQEVNKMLSDQPEVAYAQVFSGVPSSNQAFGIASMVPWSEREASQAEVVKRVTDLVKDVPEMAVTAFQMPELPG
AGSGLPIQFVITTPNQFESLFQVTTDILTAVKNNPMFVYSDLDLNYDSATMKISIDKDKAGAYGVTMQDIGITLSTMMAD
GYVNRIDLNGRSYEVIPQVERKYRLNPESINGYYVRTADGKAIPLGSLITIDVVAEPRALPHFNQLNSATIGAVPAPGTAMG
DAINWFQTEAVDKLPSGYQYDFMGEARQYVTEGSALYMTFALALAIIYLVLAIQFESLRDPLVILVSVPLAICGALIALSWG
AASMNIYSQVGLITLIGLITKHGILICEVAKEEQLLHQKNRMEAVTEAAKVRLRPILMTTAAMIAGLIPLLYASGAGAAQRFSI
GIVIVSGLAIGTIFTLFVLPVIYTYLASVHKPLPIFMEDKDLEKLKRADEARNALKARDC

>MexFQ_P_profundum
MLSRFFIQRPKFALVISIILTLAGAIAALNLPVAEYPKISPPSVGVTAYYSGASAEVVEEAIADPIEASVNGVENMIYMSSKSAN
DGSYSLNVTFDIGTDPDMAQVNVQNRISQIESKLPAEVRMVGVTVKKRSPDLLMVLNFYSPDGKYDDKFLNNYINLNVK
DQLARVKGISEVNVLGGGEYAMRVWLNPEKMANLGLTTADVNAALTEQNVQVAAGQVGAPPYNNAQEVQFNLVTK
GRLETVEEFEDVVLRANNDGSIVFLKDVARVELGKKFYTVNGMFRDQPAAIVTLNLQSDANALESGDAVMSLLDGMNK
NFPEGMAYETSYDTTLFVAESIKGVVVTLIQAIILVIAVTYMFLGSMRSTLIPVVAIPVSLIGTFAIMLAAGFTINTVTLFGLILAI
GIVVDDAILVIENVDTNMARDPSLSPRQATLIAMKEVTGPIITSTMVLLAVFIPVAMLPGITGIMYRQFALTICISVVISSINAL
TLSPALCSLVLKQGDGNKSRWFQTFNKGLDKVTLKYGSIAGYLVRKSVLLVTFFILAVGAVTYLSKTTSTAFVPTEDKGILLV
NVQLPDSASLSRTEEATIKLQDILAEEPGIEGITVANGYAFLTGAAASNGASLFIKLKEWGVRNEMEGEHSSFAISNRINARS
ATELPEAIVFAMGAPAVPGMGAASGFEFVLEDTLGRSRTDLANVMGEMIQKANQQPEIQFTFSTFRANVPHFYVDIDRV
KAKQLGIPLTDIFQTLQGNLGSMYVNDFTLYGKNFRVTVQADSEYRDNLDTLERFHVRSASGDMIPLSTLTSIEQVFEPDV
AWRYNMYRSAVIQGSPAPGYSSGDAIAAMERVAADVLPQGYQYEWTGMAYQEVKAGNQAIYAFALALIFIYLFLVAQY
ESWSIPLAIILVVPVATLGSFLALAMTGTPLNLYAQIGLVLLIALAAKNAILIVEFAKIEREENELDIEKAAVRGGKLRFRAVNM
TSWSFILGILPLIFASGAGHISQNSLGISLTGGLLCVLLAGTILIPGFFSLVQKRREKFHGGSTKLIVLEDEK

>MexFQ2_P_profundum
MKFSHFFITRPIFASVLSILILIAGGLSLPNLPISEYPEVVPPTVVVRTAYPGANPKVIAETVSAPLEEEINGVEDMLYMFSQGTS
DGRMTLTITFALGTDLDKAQVQVQNRVSSALPRLPQEVQRLGVTTEKASPDLTMVVHLFSSDGQRTSQYLANYADIYVK
NQLLRLGGVGDVQLFGGDKYSIRVWLNPDEIASRNLSTQDVLNAIRQQNTQVSAGSLGAQPASSDNEFEYLLNVKGRL
GSLEEFEQIIVRVTPEGQITRLKDLARVELGQESYALRATLNGKEAVAIPVFQRPGANALELSSQVRDLMAGLSNNFPDGVE
YKIAYDPTVFVDSSIDAVIHTLVEAIGLVVIVVILFLQTWRASLIPLIAVPVSLIGTFAVMQLLGVSINTLSLFGMVLAIGIVVDD
AIVVVENVERNIANGLSPVDATRQAMNEVTGPIIAIALVLCAVFIPTAFISGLSGQFYKQFALTITISTIISAFNSLTLSPALSALL
LKGHDEKPDLFTRLLNRLFGRWLFTPFNKVFDKGASGYEKVVKKLIRFSVIVGVIYLGLLVATGKIYSSVPGGFIPAQDKQYL
VAIAQLPDASSLDRTADVIKEMENIALETEGVQNTIAFPGLSVNGFVNASNSGIVFINLDDFENRLSPELSASQVANTLNGK
FSVIGEGFVAVFPPPPVQGLGTTGGFKLYVEDRENAGFERLFSNLQTVVMAANQRPELAGVYSSFRIQVPQYDIDIDREQV
LLKQVPINSVFDTLQTFLGSTYVNDTNMFGKTYQVNAQAAPEYRVNSEQILSLKVKNIHGDMVPLSTFVSIEPTTGPDRV
MRYNSYVSADVNGAAAPGYSSDQAQAAMIEVLKTHLQDGMHYEWTDVVYQQIIAGNTMIYIFPLVIILVFLVLAAQYES
LKLPMAIILIVPMTIFSAVGGVWLTNGDNNIFTKIALIVLIALATKNAILMVEFAREKTNEGISHLDAILIACRLRLRPILMTSIAF
TAGVVPLVVATGAGSEMRQAMGVAVMYGMVGVTFFGLLLTPVFYMAMTYKKKKS

>MexFQ_L_pneumophila (“MexQ”)
MKIALFFIDRPVFATVISIIIVMVGVLSYFNLPVEQYPQVVPPTIQVYASYPGANAKTVAETVATPIEQEVNGVENMLYMDS
QSTDDGQMRLTITFKLGTDLDQAQVLVQNRVAVVEPKLPEEVRRLGITTNKNSPDLMLVVNMYSPNGQYDQTYIGNYAI
LHIRDKIRRIEGVGNVLVFGASEYAMRLWLNPDLMNTFNLTANDVLAAVRAQNVQVASGKLNLQPQKKQYGFEYHIETK
GRLVKKEEFENIIVKSGESGRIVRLKDIGRVELGTQNYLTKGYLDKYPAVALPIYQRPGTNALATAQEIIKTMKTIAKDFPPGIT
YDMAYNPTLFVQQSIDAVFHTIYEAIALVVLVILIFLQTWRASAIPVVAIPVSLIGTFAVMQAIGFSLNYLTLFGLVLAIGIVVD
DAIVVVENMERNIQAGMNPRDAARKTMTEVGSALVAMGLVLIAVFLPTVFLEGISGRFYQQFGTTLAVATAISVFVSLTLT
PTMAVLLLRAHHGTIKQEGLAWWKKPIYGFLHGFNRLMEGFSRQYGKLVATLTRKTALMIIAYGIFISITLLLFVYVPRGFIPR
QDQGYFIVAVQLPPGASLSRTDAVINKAVNKILAIPGIAHTVSFTGFSGATFTNSSNAGAIFTPLLSFQERQRMGINYNDIL
KRLRQELSTIKEALIVVIPPPPVRGIGNAGGFKMMLQDRGGRGLDVLMEAAATMVNAANQEKATTSVFTFFENSTPRLHL
KLDREKVERLNVPYANVVEALEVYLGSVFINEFNYLGRTFRVIAQADSEYRHTEDDILRIKVKSNSGDMVPIGSVAQIENTV
APSRMPRFNLYPAIDLQGDVAPGYSSDEALVTMEKLAQNNLPDGIGYEWTEIAYQQKMVGNTALMAFTLGVIFIFLVLAA
QYESWSLPLAVILIVPMCLFSSMLGVKILGMENNIMTQIGFLVLIGLASKNAILIVEFARQLENRGYNLWKAAIQAAKLRLRP
ILMTSFAFILGVFPLIISTGAGAEMRRALGVAVFSGMLGVTFFGLVFTPLFYVLISRLSRYKRKLKDLN

>MexFQ2_L_pneumophila (“MexF”)

MISKFFIERPVLANVIALFIVFIGIIAIAVLPVSQYPAIVPPTIQVTTTYPGADAKTLINTVALPIEQQVNGVEDMLYMQSTSTS
GGTYTLIVTFAIGTDLNYAQVLVQNRVQAAMAQLPESVQKQGVIVQQKSTAILQFITLTSKNNEYDGVFLDSYATINMQD
ELSRLPGVGNVVVFGSGSYAMRVWLDPKKMLAFSLNPSDVLNAISYQNKEVSAGQLGAPPTVGKQAYQFTVNVPGQLS
DPKEFENIIIKTIDTPPDQNSSASSSAQVVRIRDVGRVELGSESYNQLANLNGKPTAAIGIFQLPGANALDVAQEVRKAVAK
MAKQFPPGLEYSIPFDTTVFVKASIEEVYKTLFEAGILVLIVIVVFLQNFRASLVPATTVPVTIIGTFFAMLLLGYSINLLTLFALV
LAIGIVVDDAIVIVEGVTQHIEKGLSPKESAILAMKELFGPIIGITLVLMAVFVPAGFMPGLTGSMYAQFALVIAATAFISAINA
MTLKPTQCALWLKAPDTSKPKNIFFQAFDRIYNPIESAYVKFIDRLVHRSGKVCLIGIILVACAIYGLTRIPTGFIPMEDQGYL
MLSIQLPDGASLGRTDEVVSRLAKKASEVGGVDNVIAIDGISLLDNNSLLSNAGVIYVIFKDWSVRGKSENLRALYTKFNAI
AKETLDAKVLVVVPPPIQGLGMSGGFQMQVELQDGTFDYRKLQQATDQMINTGRQYPQLQNLMTTFRASVPQVAAPI
NRTKAESLGVRVADAFDTLQTYLGSSYVNLFTKFGQVFPVYVQADASSRVSSEDLRNYYVRNQSGSMVPLGTLTDVGPA
VGPSIISLYNLYPSSNINGVAARGYSSGQGIQVMEKLAKEQLPPGISYEWTSTAYQEKVAGNLSYFIFALSLVLVYLILSGQYE
NWLIPSAIILSVPLTLVGTVLALGSLGMDNNMYTQIGLLLLIALATKNAILIVEVAREQREIHNKSVLEAAVIGAKTRFRPILMT
SFAFIMGVMPLVFATGAGANSRRSIGIAVSSGMLASTCLAVVFVPVFYVLLQTWQDKRKAKH

>MexIW_L_pneumophila (“MexI”)
MKFTDLFIKRPVLSIVVSMLIFLFGINSIYKMQIRQYPKMENTVITIMTGYPGADAELIAGFITTPLESAVASAEGIDYMTSSSQ
QGLSTITLNIKLNFDPQIAFTDVMSKVQQTLNQLPKESQQPVILKKSDSSTALMYISLDSKDMTPQQITDYATRVVQPQLQ
TVDGVAKADILGGQTYSMRIFMNPIKMAALNVTPADVSAVLASNNFLTAAGSTKSEYVAINVTAKTDLNNVEEFERLIVKS
DKGSIIRLRDIAKVELGSQNYDSSVTFNSKKAVFISITPTPTANPLTVINDARKLFPSIVREFPPSLTGTIVYDATAFIRASIHEV
METIIEAAVIVIIVIFLFLGSVRSVLIPVVTIPLSLVGVCTLMLILGYSINLLTLLAFVLAIGLVVDDAIVVVENVHRHLEEGKTPFE
SAIIGAREIATPVIAMTITLAAVYAPIGFMSGLTGALFKEFAFTLASAVIISGVIALTLSPMMCSKILSEEATSGKFAIFLDEFFNK
LKRVYQNALHSLLDTREMMLLLAGVVILMLPYLYSNTAAETAPDEDQGFFFVMATAPQYATLDYIEAYTKPFDAIYKSFPET
ENYFTVNMSQPISGMVLKPWDQRKKSQFALKQPLQNKLSKIAGLNAFAIVPPPLPGGGGGPAVQFVVKTTNDFQSLMD
VSNKLTEKARNSGLFIYIDNSLKFNQPQVEFSINRAKASEMGLDMRALGSSLTSALSGNYINYFNLEGRSYQVIPQLARKFR
LTIEQLGQIYVKTLNGTMVPLSTVITPTEKTQPNAATHFQQLNSATIQAVMMPGKTLGQGLEFLQKAAQEVLPKGFTYDY
GGESRQFMQEGSALIFAFLFAIVVIFLVLAAQYESFRDPLIVLISVPMSICGALIPLNLGVATINIYTQVGLITLIGLISKHGILIVD
FANHLQREKNLDKRAAVEESAGIRLRPILMTTAAMVFGVIPLLIASGAGAASRFDIGLVISTGLLIGTCFTLFVVPTLYTYLAE
DHRHKPNEPAEIQ

>MexFQ_N_oceani
MNFARFFTDHPVFASVLSILIIIIGSLAILTLPIERHPQIAPPTVVVSTTYPGASGETVAKTVATPLEQEINGVEDMLYMSSQAT
NDGALRLTITFELGTDLDKAQVLVQNRVALAEPRLPEEVRRMGISVRKSSPDLSLVVNIISPDQRYDQIYLSNYALIHVREAL
ARLPGVGDIALFGGRDYSMRVWLDPESLASRELTAMDVVRAIREQNVQVAAGTLGEPPIPGSTDLQLTVNTMGRLTEAE
QFGKIIVKTGTEGQVTYVRDIARIELGAASYASNLYLGSQPTVGIIIFQRPGSNALETKQAVMETMERLAKDFPPGIAYRVVY
DTTTFIEESVKSVVKVLFEAVLIVLVVVLVFLQNWRATLIPMLAVPVSLIGTFAVLAALGFSLNTLTLAAMVLAIGIVVDDAIV
VVENVERNLEAGLPRREATLKAMGEVSGAIIAMALVLGAVFIPTAFISGVTGQFYQQFALTIAAATIISAINSLTLSPALSAIFL
KSPDEKKDFLQRWLDRLFGWFFKGFNWVFGWFWTKYTLLIRRLVRMSVLMLGIYGGLLGLTYLGFTQVPTGFIPQEDQGY
LIVFAQLPDAASLERTDAVLRRANEIILDTKGILSTNAVAGWSVIMRGNQPNVGTIFVILDPFSERTEENLGANAIAETLQQR
LGEIQEAFIGVFPPPSVRGIGNLGGFKMEIQDQGGRGLHALQDAVEQVIITGNQTPELSGLFTTFRANVPQIYLDIDRTQAR
SMGVPLSNVFDTLQVYLGSLYVNDFNLFGRTFRVVAQADGAFRDQPEDIYLLKTRNVSGEMVPLGALVDIQETTGPDKV
NRYNLFPAAEVNGDAMPGISSGQAISLMEAIAERELPRGFGFEWTELTYQQILAGNTAVYIFPLCVLFVFLVLAAQYESWSL
PLAVILIVPMCLLSAIMGIWFMGMDNNIFTQIGFIVLIGLASKNAILIVEFAKQLQDKQGLNRYKAAVESSRLRLRPILMTSFA
FVFGVLPLVIAEGAGAESRELLGVAVLSGMLGVTFFGLVFTPIFYVVVRTFVERRQVRVRAEMPKPVHKEGDIEPLTPGNSD

>MexFQ2_N_oceani
MFSAFFIDRPKFALVISLVMLLVGGIALSLIPVAEFPEITPPQVTVSASYPGANAEGVEQSVAVPIEEQVNGVEGMSYMSSTS
SSDGSYQLNITFEVGTDADIAAVNVQNRVAVANTQLPQEVIRQGLTTRKQSPNMLLIVNLISPKGSRDSLFLSNYASIYLQ

NPLARISGVGDVSQFGVQDYGMRVWLDPDRLTALGLTASDVVKAIEEQNIQASAGSLGAPPFTKAPQFQYTLQAKGRLA
TVEEFEDIIVRASADGSLVQLKDIARLELGSQSYNAISKLNSQPAASIVVYQSPGANALQVANQIYAELERLAQSFPPDMEY
HILYDTTQAVRASVQEVIKTLLITFVLVVAVTFLFLADWRSTLIPVCAIPVSLVGAFAAMYVLGFSANIITLFAMILAIGIVVDD
SIVVVENVQRIMRENALEAREATRRAMAEITGPVIATTLVLLAVFVPVSFMPGITGQLYQQFAVTISVAVAISSLNALTLSPA
LCRLLLKPDSGQTHGLLRAFAWLVNRLRNGYVRVCSLMLRHLRISLLLVLTFVIVTVFLFQRAPSGFLPTEDKGVLMVNVQL
PDGASLARTEAVIDEITQMTRDTAGITDVISVTGFSILGGAASNAGLVIPILAPWDERSSPQLQWEPILGHLNASLASIAAAE
AFAFPPPPITGLGTGGGIEAQLQDLGGGSPQELAAAMGSLVFAANQHPALMGVFSTYSASAPQLYLDIDRERAKVLGVSI
ADVFTTLSANLSSLYVNDFNLFGKSYRVMVQAEAEYRNAIKDIGNLYVRSGPGEMVPLRALIDVEPVLGPLSITRYNQFKS
AAITGNPAPGYSSGEAIAALEQVAAEALPEGYRIEWTGTSKEELAAGALVIYIFALAIVFAYLFLVAQYESWTLPLGVISSVVF
ALFGALLPVVMLPFLDNNLYAQIGIVMLIGLASKSAILIVEFAKERREQGLSTHEAALTAARLRFRAVLMTALSFILGVSPLLF
ASGAGAASRTSVGFVVFGGMLAATVIGIFFIPVLHVLYQDIRLSVPRVWQRPTLRPAKLVPKETEQSDDEQG

>MexIW_N_oceani
MKFTDLFVRRPVLASVVSLLILLIGMRSLTLLEVRQYPETENTVVTVSTAYPGASSELINGFITTPLQQAIAEAEGIDYLVATST
QGHSIIEAHMVLNYDSNAAVAEIQAKVASQRNVLPEEAENPVIDSTTGDSTALMYMAFYSEAMSPAQITDYLLRVVRPKL
QAVPGVSKAQLIGNKTFAMRIWLDPRRMAALGVTANDVREVLLENNYLASVGHTKGAYVAVDLSATTDISREEDFLNLV
VREADGALVRLRDVAEPQLGAEDYDSSNWYNGKPAIFIGIEQAPGANPLDIAGHLHDLMPEIRRQLPSGVDGYIVYDASA
YIEDAIREVFRTLAEAVLVVLVVIFLFLGSLRAALVPAIAVPLSLIGGAFLMLVLGFSLNLLTLLAMVLAIGLVVDDAIIVVENIH
RHLEHGESRFQAAIHGARELGLPIIAMTTTLVAVYAPIGFMGGLVGTLFTEFAFTLASAVLVSGVVALTLSPMLSSKMLRPVS
EAGRFEQWVERFFTRLAGYYLRLLRYALESLPVVIVFAGAILCSIYFMYVTSQNELAPTEDQSILFFQATAPQTATIDYDEAYS
RQIIDIFESFPEYHESFLLLGQGGDPSTVFGGFKMPVPSQRERSQMEIQPEMQQKLQGIAGFQIAVFPRPSLPGSGGGLPL
QFVITSAADFSRLDQIAEELIRQSMASGKFAFLQKSVKFSRPKTTLKINRDLAGDLGIRMKDIGQNLGIMLGGGYINWFNLE
GRSYKVIPQVDRRYRLDQEMLENYYIRTGAGELIPLATLISFEETVEPSKRVQFQQLNSLTVQGVMAPGVAVGEALAYLED
KAREIFPSAFSWDYAGESRQYTQQGSALMVTFFFSLLVIYLVLAAQFESWRDPVIILMSVPMSIAGALVFLTLGFATVNIYT
QVGLITLIGLIAKNGILIVEFANQLQLQEGLDKQAAVEKASSIRLRPILMTTVSMIVAMVPLLMASGPGAVSRFDIGLVVASG
LGIGTLFTLFVVPAVYLLVAGDHREEKEAVQEPMEQESGFKA

>MexDYB_H_chejuensis
MRDSIQGDALSQRRLAAFFVNRPVFSWVAAIVVMLAGALGLMSMPVSQYPDIAPPTIMISASYPGASAEVVENSVTQILE
QELKGLDNLMYFSSRSSSSGASEVMLTFQHGTDADIAQVQVQNKAAQAAGRLPAPVQQQGLTVNKSQNNFLMILGFY
DASDRLSDTDISDWLAGSLQDPLSRVDGVGAVQNFGSPYAMRIWLDPHKLNSFRLMPKDVTDAIAAQNTEVSVGELGA
LPARPGQQLNVTVTSMSRLTSPQQFRDIIIKTDADGAVVRLGDVARVELGAEDYGGTTKLNGHPASGLAVMLAPGANAL
QVAEAVKQKVAQLRGVFPPGVKVAFPEDTTRFVKLSIHSVLQTLLEAVALVVLVMFAFLQNWRATLIPAITVPVVLLGTFG
VLAAAGYSVNTLSLFAMVLAIGLLVDDAIVVVENVERIMREEGLDARAATLKSMDRITGVLIGVAAALAAVFLPMAFFSGS
VGVIYRQFSVTLVAAMALSAVAAICLTPALCVKFLRHGEASAGGFNAFNRGFARFQQRYVGGLRFMLSRPVRFSAAYILA
VGLMALVYWRLPTAFIPDEDQGTVLVQFNLPAGATYDRAAKVAQEIERYFLEQEKANVETVYILSGFSFDGAGQNAGMG
FVALKDWSQREGPENSAQAIADRATEAFSRLRDAQAYSMVLPPIEGLGRSNGFELWLQDLGGLGTEGLAAAAAELEQRL
QGGEAFSYVRAGADRNVQQLRVDIDREKASALGLALEDVNETLSVAWGGSYVNDFVHQGRVKKVIVQADAPYRAAPE
DLRHWFVRGAGDAMTPFTAFSATQWRNGPTELSRYNGLQAVPLFGAAAQGVSSGEAMAQVEKQAASLPGTNFEWSG
LSYQDKLSSGQAPLLYLASILFVFLCLAALYESWTIPCAVAPAIPLGVLGALLAVYWRGLPNDIYFQVGLLTTIGLSAKNAILIV
EFAEAAVRQGMAPMTAVVEGARLRLRPILMTSLAFGAGVAPLALSTGPGSASQNAIGTSVLGGVLAATALAIFFVPLFYL
WVKGVFFTGRRRAFATVKKSGAVA

>MexFQ_H_chejuensis
MNLSQYFIHRPILAGVLSIMIFIAGALAVFELPITEYPEVVPPTVVVNATYPGANPKVIAETVASPLEQEINGVENMLYMSSQ
ASSDGRMALTVTFALGTDLDEAQVDVQNRVSRALPRLPNEVQRLGVTTEKSSPDLTMVVHMVSPDNRYDTLYLANYAT
LNVKDALSRIEGVGAVRVFGAGDYSLRIWLDPEKVAALNLTTSDVLHAIREQNRAVAAGTLAAPPSPSASDFQLLINVKGR

LESTEEFENIIVRVGPNGEMTRLKDVGRVELGSSTYALRSLLNNKPAAALPIFQRPGSNAIEISDNVRATMAELKKTFPEGVD
YSIVYDPTVFVKGSIKAVVQTLLEAILLVVIVVVLFLQTWRASIIPLVAVPVSLVGTFAVMYLFGFSLNALSLFGLVLAIGIVVD
DAIVVVENVERNIELGMTPVEATKQAMKEVTGPIIATSLVLSAVFIPTAFISGLTGQFYKQFALTIAISTIISTINSLTLSPALSAV
LLKPHSAKKDLLSRIIDGLFGRWLFAPFNKFFAKASNAYTWGVKRIIRGGLVILVVYGGLIGLTYHQFQATPVGFVPQQDKQ
YLVSFAQLPDAASLDRTEDVIREMSAIALKHPGVESAVAFPGLSVNGFVNAPNSGIVFVTLKPFEERKDPSLSAGAIAGQLN
GEYSAIKEAFVAIFPPPPVMGLGTIGGFRLQVEDRASMGYNELYAQTQNIVQKAWATPGLTGLFSSFQVNVPQIDVDVDR
EKAKSHNVSLNDVFETMQVYMGSLYVNDYNQFGRTYQVNVQAEDDFRQEADQISRLKVRNGQGDMIPIGSFVNVTHT
AGPDRVMHYNGYPTAEINGAPAPGYSSGQAQAAIEKLLNDELPNGMTFEWTELTYQQILAGNTAVFVFPLVVLLVFLVL
AAQYESWSLPLAIILIVPMTLLSAMTGVILSGGDNNIFTQIGLIVLVGLATKNAILIVEFAKERQDAGLSLMDAILEACRLRLRP
ILMTSLAFIMGVAPLVYSSGAGAEMRHAMGVAVFSGMIGVTIFGLLLTPLFYHLIRRWVKKRDPQDEAQGEAAHA

>MexIW_H_chejuensis
MKAFTDIFVRRPVLALVVNIVIIIAGVQAWNSLSVRQYPQSENASVTVATTYVGASADVVRGFVTTAIERAIASADGVDYIE
SKSMLGLSLVTARLKLNYDPTKALADITAKVNQVRNELPAGSELPAISVQSADSEFAAAYLSFWSESLSQSEITDYLVRVVQ
PRLAALSGVQRADIFGARTFAMRIWLKPDHMAALNISPTQVRDALAVNNYLAPVGSTKGSFVQTYLTANTDLHTVEQFQ
ELVIRQENDTIIRLQDIADVELGAEEYDTEVSFNGQTAVFMGIFPLPSANTIEVVERVRKDLEAIKEDLPPGLQGGIGYDASEY
INNAIHEVVGTLTDTLLIVVVVIFLFLGSFRSVLVPIVAIPVSLIGAIFLMQMFGFTLNLLTLLAIVLSVGLVVDDAIVVVENVER
HLREGRSPVEAARIGARELIGPVIAMTITLAAVYTPIGLQGGLTGALFREFALTLAGAVTISGIVALTLSPMMSAKLLRSAESEE
KGFTGWVNHRFDRLRDGYGKVLEKVLRARPAVYTIWAGLSLMVIPMYMFSPKELAPLEDQGFMFGIINNAANASADQK
AHFGHAAEQIFLNAPERDLTFQILLSPSDPFAAALGVGGFSGMVVKPWNQRDRSIAEIVMDMQNQLNAIPGLQVFAAQ
PPALPGGSNFPVEFVIASTAEPEQMLQFAQQLQMKAMESGLFYFPPDIDLKYDQPQSEVVIDHRKVAALGLNNAQIGAD
LSAALGGNYVNRLNMDGRAYKVIPQVSRVERLNPDQLKDIYVSGPEGQLMPLSAVATITDSTVPRSLNRFQQLNAVKLS
GMTAQLDEGLKALEKAAEEILPPGYTINYTGESRQLRTEGGKFLPALGLAILMIFLVLSVQFNSFRDPFVILLGSAPLAMFGAL
LFTTLKMPDPNMPYWTNGWTTTMNIYAQVGLVTLVGLISKNGILIVEFANKLQMQGMDKLNAVHQAAMTRLRPVLMT
SVATVAGHFPLTLVEGPGAAARNSIGLVLVGGMAIGTLFTLFVTPALYVLLARQHKDDPVAEEIESGHAQPAPG

>MexIW2_H_chejuensis
MRFTDIFIRRPVLASVVSLIILLLGLNALKTLQVRQYPQLENAVINISTAYPGASSELVSGFITSPIQQAVASAEGIDYLTSSSN
QGSSLVEVHLKLGFDSSTAMTEILSKVAEARRQLPSDAEEPVIQKSTTGSTALMYVSFFSRDMSEAQITDYLKRVVQPQLET
ADGVAKAEILGGKTFAMRVWLNPQKLAAFDLTPQDVARAVGSNSFLSAAGATKGQYITANITADTDLHSVQEFQQLVV
KRDDATLVRLQDVATIELDAESDDSAVYFNGNRAVFMAITSLPAANPLEVIDNIYDLMPALEKQLPSALNVKVAYDATKFI
RDSINEVIKTVGEATVIVIFVIFLFLGSFRAVMIPVVTIPLSLIGVCFFMLALGYSINLLTLLAMVLAIGLVVDDAIVVVENIHRHI
DEGLSPFKAAIIGAREIATPVISMTLTLAAVYAPIGFLGGLTGGLFKEFAFALAGAVIISGVIALTLSPMMCSKLMTETNSKMAI
WLDEVFEKLTGAYQRRLHNALNYRPVTLVMVLTMLVSCFFLYQLSNKELARDEDQGIIFSMSTAPVSATHDYVAAYTDEV
IKTIETFDERNDYFIVQGADGAANSMFSGMILKTWGERERSQMELVPIMQQRMGDIAGYQSVSFNLPTLPGSSGLPVQF
VITTAQDYKVLYELGQELLDEARKSGKFLFVNSDLKFDKPTAHISINRSKAAELGISMQDIGAALATMLSGGYTTRFSVEGRS
YKVIPQVLREYRATAEQLDDYYVRTASGELIPLSTIAEVSKGVEPNKRGQFQQLNAMVVEGVMTPGISLGEALSFLEQKAQ
TIFPQGYGYEYAGQSRQYKQEGSALIFTFFFALLMIYLVLAAQFESFRDPFIILVSVPMSLLGALIPIAFGVTSVNIYTQIGLVTLI
GLITKHGILIVEFANQLQVEQGLSKREAIEQAAGIRLRAILMTTAAMVVGEAPLLFASGAGAVSRFDIGLVIASGLSIGTLFTLF
VVPTMYMILGADHSQIEKQEDVDGELSHA

>MexFQ_A_ehrlichii
MLRFFIDRPIFASVISIIILVAGLASLRALPIEQYPDVVPPEIVVQAAYPGASSEVLAEAVAAPLEQEINGVDDMIYMESTSTDA
GTVQISVSFEMGTDPDQAEINVNNRVQAALPRLPQAVRDQGVRVEARSTNILLVATLSSPDGRHGTLELSNYALLNIIDEL
ERLPGVGEASLFGQQDYAMRIWLRPDKLAQYDLTPAEVAAAIREQNAQFAAGQMGAEPAPDGQAFTLTVTTRGQLEG
AEEFEAIILRSDESGATLRLGDVARAELGAQSYAFSATYNGEPTVPIGVYQAPGANALETAEQVRAALADSAERFPEGVEYT
IPYDTTEFVEVSIREVYTTLLIAVGLVVLVTFVFLQHLRATLIPITAIPVSLIGTFAGMQAMGFSVNLLTLFGLVLAIGIVVDNAII

VMENVERLMREKGLKAREASVETMKQVSGAVVSSTLVLVAVFAPVAFLGGLTGELYRQFAVTIAFSVVISGVVALTLTPAM
CALLLDKQPKTPWLPFRLFNAGFEHLTRAFVAAVGFLVRNRTVGVGLFALAVGGAVFLVERMPDGLVPQEDQGFALVVA
QLPPTSALNRTEAVRDALAAQLTQLEEIQEFTAFAGFDIIAGSLRTNAAVGFVNFTDWADRPRPDQHAAAMSERISGMG
FGLQEANVFAFIPPPIQGLSLTGGVEGFLQVREDMSARQVEALANRVVQRANERPELVNARSTLDTGIPRYRAELDREKAK
AAGVRIDEVFNTMRATFGALYVNDFTFAGRLWQVNLQSEQDFRSHPEDLRHVFVRSESGDLVPLSALVRLTRESGADIIN
RFNIYQAAKLMADPAPGYTSGDAKAALEAVVAEVRDEEGADALLGWIGEAYQLEVAAGAGAAAFAMGLLMVFLILAAQ
YERWTLPLAVATAVPFAVLGAALFALLRGFPNDIYFQVGLLVLIGLAAKNAILIVEFAAQNRATGMTSTEAAMAAARQRFR
AIMMTALTFIIGTLPLVFATGAGAASRQEIGTVVVGGMLAASTLALLFVPLFYKLLEDVATWRNERRARREQEKAAQADDK
EAGNHA

>MexFQ_C_psychrerythraea
MFSRYFIDRPIFAFVISIFIVLAGLAAMRSLSIAQYPEMSPPVVQVTAVYPGASAEVLEQTVATPLENAITGVEGMMYMSST
STSAGATTITVTFEIGTDPDQASVNVNNRVKQVEARLPEETRRQGVVVAKGSSSFLHVHAFYSPDDSREALWTSNYVTLN
IIDKIKRIPGTTSVQIFGAKDYAMRIWLRPDVMSQLGVTVEEISSAVREQNSQYAAGRIGATPTSVSPQSLVYSVKVQGRLS
TAKEFEDIIIRANNDGSSLRLKDIARVELGSKDYDFDGRINGKKAVLLGIFLQPGANALDVADEVNATIAELETRFPTGLEHLL
PYDTTRFVKVSIREVLKTLGEAMLLVFLVVFIFLQNWRATLIPIMAVPVSLLGTFAGLYMLGYSINTLTLFGMVLSIGIVVDDAI
VVLENVERIMHEEDVSVRDAAIKAMKEVSGPVVAIVLVLCSVFVPIAFLGGLTGELFRQFAITISISVSLSGLVALTMTPALCV
LVLKHEDKKTNFFFNGFNRFFNKVTGHYVTGVSFFLRRGLLALMLVLGMVVITANMWLKTPSSLVPDEDQGFYISAVFLPD
GASLQRTKKVVDEVIAAIESNPANENVISFTGFDFLGGGYKNNAATIFVTQKHWDDREISTQQLVGELFMKTANINEALVL
AFNPPAIPGLGNTGGFEFYLQNRGDGGVAKLKQSMQAMMGAAQQSPILAGVQTLWRSDSPQLTVEIDREQARAMDIP
INNAFNALAGTMGTYYLNDFNKFGRTWQVLMSAEPEFRMSPKDISRIYVKSNGGEMVPISAFAEINYSSGPDSLDRYNNL
SAVKLLGSAAPGYSSGQAIAEIERIANEVLPSDMSYEWTGTAYQEKRSSGSTAIALGMAVIMVFLILAALYERWSLPLSVLLA
LPFGIFGALVSVWIAGMTSDVYFQIGLVTLLGLASKNAILIVEYALMKKQEGWTTGAAALEAARLRFRPIIMTSLAFILGVVPL
ALSSGAGAGARHSVGTGVMGGMIAATFLAIFFVPLFFYWLTERKISENRKQQAITDEIAQHHEHELNSK

>MexFQ2_C_psychrerythraea
MISKTFINRPKFAFVISIVITLAGLIAMSVLPVNMYPEITPPQVQISAVYPGASAQIVEESVIRPIEEQINGVEDMMYIESSASN
NGSAVITVFFKVGTDGDIAQVNVQNRVALATSSLPSEVTRQGVDVKKKSSSMLLGINLFSEQEGINQLFLSNYATNYLTEP
LGRIKGVASAEVMGEMTYSMRAWLNPERMSSLSITVTEVQQALQEQNMIVAAGKLGASPTLPNQQFEYSIQAKGRLQT
PEEFGQTIIRAQRNGNFIRLNDIARLELGAEDYSTQARLNGKETAFLVIYQLPDANATEVASLVKAEMAELSQRFPDGLKYV
IPYDTTKFIDRSIEEVVVTLFQAIVLVILVVFLFLQNWRATIIPTLAIPVSLIGTFAFMVLMGYSINTVTLFGLVLAIGIVVDDAIIVI
ENVERILKEEQLPIKEAVTKAMDQVSGPIIATTLVLLAVFVPVAFMPGITGELYKQFSVTISFAVLISSLNALTLSPALCTVLLSA
EKMKPITWLLPFERLITKATGGYTKTVGFMLKRLTRVGLFVGLIFIGAGWLTTAVPTAFLPAEDQGYLFVDIQLPDAASSNRT
QAVLDKITPTILNDAAVEDIITVSGFSLLGGAGSNNGLAIILLKDWEERTTPELGLKQIIPRLMGQLWTMPDAQIMVFNPPPI
PGLGNSSGFDFKLQDSEGRDPAELAQVMNGLIYEANQRPELSRVFSTYRANVPQYFLEVDRNKAKAQGVALSDIFATLQ
AQLGSLYINDFSQFGRTYRVTIQAESQYRAEPSDLRYYFVRNKDDEMVPLTTLASVKPILGPTTISHFNLYRSASITGNASPG
YSSGQSIDVMEELASTLPQGYIYEWSGQSKQELEAGNLAPILFGLAIIFVYLFLVAQYESWTIPFSVIAAVPLALFGAMLALYT
VGMANNIYAQVGLVLLIGLSTKTAILIVEFAMEQRAAGETIMNAALNAAKMRFRAVLMTAFSFILGVLPLVFATGAGAGSR
VSLGITVLAGMIAATLFGTLLVPYFYLLIQRMREKIKGKPDVT

>AcrB_C_psychrerythraea
MQESKPSVMDIFVRRPVVAIVLSIIICIAGIWSASKIPVLQFPKIDSASLVIETYYIGASADVVKGFITEPIERAAATVPGVEYVD
STSTSGSSKVTAWLKLNENSTLALAELTARLGQIRFELPQGAQDPVVSVSRADRPFAVFYLNVQANGNDLSRITDYLTRQ
VNPVLNAIEGVQRVGIEGARTPAMRVWLNAQALQIFNLSASDVYQALATNNTIATMGYVENSRQKIDIVANTQLSSVEE
FQQLVISEVDGATIHLKDVAKIELAAEDTSVTARLNDKDAVYISIWPLPGANEIAIGDRLYEKVDELNKTLPHGISIDYAYDG
TLYMRDALKEIFTTLAETVILVGLVVVLLMGSFRSAIVPLITIPISILGAIAAMGIMGFSLNLLTVLAIVLSVGLVVDDAIVVVEN
VARLMRQGLSKFDAALISSRQLLVPIISMTLTLAAVYAPIGFLSGLTGVLFKEFAFTLAIAVIISGIVAITLSPIMSAYVSPKGGEE

SWLTTKVNHLFERLQRRYGQLLTHIFKWQGQVFLIALVVSLLTVPFYLYSKKELAPIEDQATVMFVIQSPPDASMAYNVAQ
MNPVVESLQEIEGGKKIWQIIFGGGGFGGLELVNADKRDYSAQSLVPQIYGKLSQIPGLNMFPILPSSLPSSGQFEIEMIVKA
TAPYSEMKQYADQLVGAAFSSGKFLFADTDLKIDLPQVELQLNREKIADLGMNVGEVSEQLGILLSSNFVNRFDANGKAY
RVIPIVNDEVRSKTEAILSLDIKLPTGELLPVSAVADLKWKTVPRQLGTFAQQASFRIYGGVAPGSNKEAALSAIEDAAKEILP
LGYSIDYAGESRQLRKEGNTLVMVLGISLLIVFLVLSIQFNSFRDPLVVLLGCVPLALSGALLIPYIELTTINIYSQIGLITLIGLIAK
NGILIVEFANHAQELGANKLAAVTEAATTRLRPILMTTAATILGHFPLVLVTGAGAEARNSIGIILVAGMFIGTIFTLFILPSFYL
LLAEKRDKLMLDEIQVSDEALQKVHS

>MexDYB_K_koreensis
MAQFFINRPIFAWVISLLILLGGTLAVQNLPIMAYPDISPPQVTINATYPGASAKVIEDTVTSVIEEEMNGIEGLRYIKSESSRT
GTSTIILTFATGTDTDIAGVEVQNRLKRVEARLPSSVRTQGVNIDKTRPDFLMVVSLYSPNGSHDATDLGDYIDRAILGEMR
RIDGIGSAQLFSATYAMRIWVDPKKMASFAITADEIAQAIRNQNAQLATGELGSLPAPDKQQINATILVPSRLNTPEQFGD
IILRSSSEGAIVRLKDVAMVERGANSYTAQAFLNGQESAAFALKLSNTGNALEAAEAVKAKMAELEQYFPDDMSWVVPY
DTSIFIDESIGQVLQTLIEAVFLVTLVMFLFLQSWRTTIIPLIVVPVSLIGTGMGLYLLGYSINMLTMFAMVLAIGIVVDDAIIVV
ENIKRIIDQEKLGPYQATKKAMKQISGAIIGTTAVLIAVFIPMAFFSGSVGRIYQQFSLTIVLSVSISAFLSLSLSPAIAQGFLKTE
TKEKKEWAFFRWFNQGFNWFTETYMSQEEKLFNKRGQLITMAIFVVICGLVGWRFASMPTGFVPSEDQGFIVSSSLMPS
GSTRSRTLELTQKTDAWFLEQPEVERIITVAGFSFFGTGQNTSITFVNLKSWAEREGMRNADQFAAAATAEFMKYSEGSTF
AFNMPPIPGLGNSSGFDFQLLDKSGVGTDQLMGAAFQLVGMASQSGKFAEIRPDTLPPAPLLTIEVDRIKARSLGVDIGEL
NSTLQIALGSAYINDYVEGQKVRQVWLQSDAETRSTPDEILTMQVRNDQGDLVNLSEVAKAQWTEAPAKLTRYNGSPS
LPITGSGSPGVSSGEVLALMEEFAQTLPKGLGYEWSGQSLEEKVAGNQTTLLFSLSFLVIFLVLAALYESWSVPLSVILVAPLG
ILGSIIAAFIGGLPNDIYFKVGLITIIGLSAKNAILIVEFAREAEGEGKSPLEAVTEACRLRLRPILMTSLAFVMGVLPLVLATGAG
SASRQAIGTSVVGGMISAAILAIIFVPVFYLLVRKIFPRKLKHYEMAARGMEINDD

>MexDYB_D_nodosus
MAKFFINRPIFAWVVAIFIMIAGLFSATRLPIEQYPSISAPTINISFAYLGASAQTIQDSVISIIEEQMNAIEGIDYMESKAYSNG
HGAITITFHSGIDADLAQVNVQNKLSQVESRLPQSVRNMGIMISQASDNFLLLTALYAEDNARQDIGEISDYAVQVIKPEL
QRVSGVGEVQIFGSERAMRIWVDPQKLKSYNLSFADVQQAIASQNIQITAGTLGDTPNVLGQQYSASIHIQGLLGSVEEF
EKIVIKYDFNGATVKLKDVAVVELGKQTYAFNTRLNGKPAIGIGVRLSSTGNAVAAAKEIRTKMESLSHYFPEGVSWDIPYD
TSTFIKISLGKVLHTLAEAILLVFIVMYLFLQNIRYTIIPTIVVPISLLGALALMYPMGLSINVLTMFAMVLVIGIVVDDAIVVVEN
VERLIVTEQLTPYQAAQKSMQQITNAVIGITLVLISVFIPMAFLSGATGAIYRQFSLVMAASIGFSAFLALSLTPALCATILKPA
RHHANKTGFFAGFNRIIKTITRLYQETVGRLIRLSYAMMAVFFGLVLLSVFIFTRIPTSFLPVEDQGTVITTYQLPAGATLERTK
HMIEKAEDIVLNNKDLVKSMTTVIGFSFSGQGQNMAFSFISLNDWSQRRGKGQDSISFTRRLNGMLQSINEAFIFALTPPA
IPSLGVSSGFSLYLQDRNNQGHEALLAARNQLIGMARQSKILTSVRPSGLEDATQLQIDIDRNAAFAQKVPLTSIAHTLGN
ALGSNYLNDFPNKGRMQRVYVMAQANARMQPEDVLNLTVANLRGELVPISSLANVKWIKGSEQVTRYNGYTAMAVE
GAAVPGHSTGEAMAEISRLAAQLPDGFAVEWTGQSLEEIRSGHSEYYIYAFSALAVFLCLAALYESWSIPFAVLLVVPLGIFG
VTMGNFFRGYENDIYFKIGMITVMGLSAKNAILIIEFAKELQDDGMSKVRAAMSAAALRFRPIIMTSLAFIAGVVPLYFASG
ASSSSQRAIGTSVLWGMLIGTILSIFFVPIYYVVIRKIFSGKKRNTDAESSLTHQQG

>MexIW_T_sulfidiphilus
MRFTDVFIKKPVLATVVSLFILLLGIRAGFDLNVRQFPEIKNAVVTVTTAYIGADADLIQGFITTPIEREIAAAEGIDYIVSTSVA
GVSLVQANLRLDYDPNEALTQIAAQVNKVRSDLPLEAEEPVIELSVGQTIAAMYMSFYSELLSNNQITDYLVRVVQPQLST
IPGVQRAQILGDRTFAMRIWLDPERMAALNVTGADVAAALRANNVLSAVGRTKGEMVSIDLKASTDLSSAEEFEQLVVR
AEEGAIVRIVDIAEVELGSENYDVSVSFNGQSATFIGIEVAPDANALDVIAEVRRVWAEQIEPQFPQGLSGDIVYDSTLYIQS
AIDEVLLTIFLALVIVVGVIYLFLGSVRSVIIPALAVPLSLVGTLFLMLLMGFSINLLTLLAMVLAIGIVVDDAIIMVENIHRHIEEG
MAPLEAAMRGARELAWPIVAMSTTLVAVFLPIGFVGGITGTLFIEFAFTLAASVIISGIVALTLSPMLSSRLLKAHGEGSKGRL
EAWLDARFDGLRRRYQRRLHGALDDKHVVLSFGLIVLVSCYFLFITSPSELEPAEDQGFVFSIAEADAPTTLDHLERNTAVM
NRYLDEIPEMRNIFIVNGFGGAGVGATNTAIAGFVLAPWDQRERSTQQVLEQDLQPRIEQIPGLNIFALVPPSLPGGGGG

PPVEFVIRSTEPAENLQAVAQEVVGRAMQSGRFIFIDGDLKIDKPRTEIHIDRDRAALMGVDMQSLSADLSALFSGGPAGR
FSLDNRSYRVIPQLERVQRLNPEQIEDLYTRNREGELVPLATLITLEDTVQPQQLKRFQQLNSVTLSGVPRPGVTLGEALDL
LDGIAAEVLPPGYSVDYAGQSRQFKSEGAQLILTFFFALVVIYLVLAAQFESFRDSLIMMMTVPMSIAGALIFVSLGFATLNI
YTQVGLVTLIGLIAKHGILIVEFANKLREEGLSKREAVEEAAAIRLRPILMTTAATVLAMIPLLIAAGPGAGARFAMGLVIASG
MTIGTLFTLFVVPAMYVYLSRDRAVQTAADEAVRV

>MexIW_S_degradans
MKSITDIFVRKPVLAIVVNIIIIIAGLQAVSSLTVRQYPRNDNAVITINTAYIGADAELVRGFITSPIERAVSGADGIDYVSSSSS
QGVSSVSVRLELNYDPIKALAEITSKVNQVRGDLPPEAEVPVINVESADSQFAAAYLSFSSDILADNQITDYLVRVVQPRLS
ALEGIQRADVLGARTFAMRIWLQPDRMAAHNISPTQVRQALQANNYLAALGSTKGQYIQVNLSANTDLNSVDEFKELV
LRSENGAIIRLKDVAEVVLGSESYDMEVRYSGKTATFMGIFPQPNANSLDVMALVREEMKNMQAALPKGLDAVIAYDAT
AYIESSISEVISTLIETLLIVVVVIFLFLGSIRSVIIPVVAIPLSLIGAVFVMQIFGFSLNLLTLLAIVLSVGIVVDDAIVMVENVERHIS
EGQTPFKAAILGARELVGPVIAMTTTLVAVYLPIGLQGGLTGSLFKEFAFTLAGAVTISGIVAITLSPMMASKMLKEGMEDEG
FASTVARIFDRVKNRYTRMLDHTLEHRFPVYMVWVATAILGVVFYMQSPKELAPTEDQGVIFGIVEGSASSTMDQASIFA
AAANDAFSSFEETDFTFQLTFPNSGFGGMVLKPWDERERTVFDVLPEAGGKLSQIPGINLFPITPPALPGGGNMPVEFVIA
STAEPEQILKFAQELQQKATMSGQFAFPPMIDTQIDLPKAELIIDRDKVADMGLNMQQVGADVGTMLGGGYVNRFNIA
GRSYRVIPQVERIERLNPEQLGNIYVTGPEGKLVPLSTLARVERKTVARSLNRFQQLNAVKLSGVAIGPLDNALKVLEEEAA
KILPQGYMIDYTGESRQLRREGNKLLPAMMMASLLIFLVLAAQFNSFRDPLIVLGGSVPLAWFGALIFTFLKMPNPNMHF
WTDGWTTTLNIYSQIGLVTLIGLVAKNGILVVEFANKMQEQGLTKLAAIKESAGTRLRPVLMTTVATVAGHFPLVLVTGAG
AEARNSIGLILVGGMAIGTFFTLFVIPSIYMLIAKDHRGEKFHVSEFELEDELIEEESKTTA

>AcrB_D_dadantii
MAKFFIDRPIFAWVIAIMVMLTGTLAILKLPIAQYPTIAPPAVQITANYPGADAKTLQDTVTQVIEQNMNGIDKLLYMSSNS
DSSGTVQITLTFDADANPDIAQVQVQNKLQLAMPLLPQEVQQQGVSVQKSSSSFLMVLGFISDDNNMTQQDIADFVG
ASIKDPISRVRGVGDTQLFGAQYAMRIWLDPDKLNNYQLTTSDVVSAIQVQNNQIAAGQLGGTPPVPGQKLNASIIAQT
RLNSPEQFGNILLKVNQDGSQVRLNNVARIELGGESYSVIARYNGRPAAGLGIKLATGANALDTASAVKAEITKLQSTFPA
GLKVVYPYDTTPFVKISIYEVVKTLLEAIVLVFVVMYLFLQNFRATLIPTIAVPVVLLGTFSILSAFGYSINTLTMFAMVLAIGLLV
DDAIVVVENVERVMAEEGLSPKEATRKSMGQIQGALVGIALVLSAVFIPMAFSGGSTGAIYRQFSVTIVSSMVLSVLVALIL
TPALCATILKPITNHGEKKGFFGWFNRLFDKSTHHYTNSVANILRSTGRYVVVYLIVVAGLGLMFTRLPSSFLPQEDQGVLL
TMVQLPVGATQESTQKVLDQVNDYYLQHEKENVKSVFTVNGFGFAGRGQNMGIAFASLKDWDERSGAANKVDAIIGR
AFGAFSQIKEALVIPFNIPAIVELGTASGFDFELIDQNNLGHDKLMAARNQLLGMIAQHPDTLVRVRPNGMEDMPQYRLE
IDQEKAESLGVSIATINATLSTALGGTYVNDFIDRGRVKKVYVQAEAKFRMLPNDIQKWYVRGTSGQMVPFSAFASERWE
YGSPRLERYNGLPAVELVGEAAPGKSTGEAMALMEELAAQLPPGIGFDWTGMSYQERLAGNQAPALIIISAIVVFLCLAAL
YESWSIPFSVMLVVPLGVFGAVMAAGLRGLENDVYFKVGLLTTIGLSAKNAILIVEFAKDLMEKEGKGLVEATLDAVRMRL
RPILMTSLAFILGVVPLVISTGAGSGAQNAVGTGVMGGMITATVLAIYFVPVFFVVVRRRFGKKKEADDSHNEHTQTSH

>MexDYB_D_dadantii
MFSRFFIHRPVFAWVIAIVIMLAGALSIESLPVAQYPNVAPPSVAIKASYPGASADTLENSVTQVIEQQLTGLDGLLYFSSSS
DSSGDVRITATFTQGTNPDTAQVQVQNKVQQATSRLPTEVQQQGITVTKSQQDFLLIMALYDTTDKSTAADIADYMVS
NLQDPLSRVEGVGSVQVFGSQYAMRIWMDPTKLAAYSLMPSDISSAVETQNTQVSAGKIGAQPASSDQQLTATVTAQS
RLKTPAQFRDIIVKSESSGALVRLGDVARVELGNEDYTTTTRLNGHPAAGIAVMLSSGANALATAERVKAKVAEFTPQMP
QGYEVSYPKDSTDFIKVSVEEVVQTLFEAIALVVLVMFLFLQNLRATLIPAVAVPVVLLGTFGVLAVFGYSINTLTLFGMVLAI
GLLVDDAIVVVENVERLMRDQGLSPVAATEQSMREISSALVGVALVLSAVFLPMAFFGGSTGVIYRQFSITIVSSMLLSVVV
ALTLTPALCAALLKPAAHTPSNGNGFFARFNRGYENLQQRYTGRVAHVITGPWRYLALYGLVIVVMGVLFLRLPGGFLPN
EDQGDVMVQFTLPAGATETRTSKVGERIEHYFLSEEKDNVEAIFVISGFNFSGSGQNAGMAFVALKNWSQRPGSANSST
AIAHRAMQALSAIRDAQVFTMSPPAVQGLGQSDGFTFELQATGNTDRSRLLALRDQLLAEAAKNPRLTAVRPNDLVQM
PQLQVDLDYAKVRALGLSISDVTSTLSSAWGGTYVNDFIDRGRVKKVYIQGDAGSRGQPSDLDKWFVRGTDSSGNSVM

TPFSAFAATRWSYGPESLSRYNGLASYEIQGAGAPGVSSGKAMDEMEALARALPAGTTFAWSELSYQERLASGQAISLYA
MSILVVFLCLAALYESWSIPFSVMMVIPLGVVGAVLAATLRGLENDVYFQVALLTTIGLSAKNAILIVEFAEAAYRRGATLYE
AALEGARTRLRPVLMTSLAFIAGVFPLAVATGAGANSRISIGSGIVGGTLTATVLAIFLVPMFFVLVRRVFPGRHAPTSPSTS
MPTPTQGEN

>MexDYB2_D_dadantii
MPQFFIHRPVFAWVIALFIVLLGTIAIPQLPIARYPSVAPPTVSITASYPGATPQTMNDAVVGLIERELSSVKNLLYFESSVDTS
GSAQISITFKPGTDPELAQVDVQNRIKSVEPRLPQTVRQNGLQVESASSGFLLLVSLTSDSGRFDQVALSDYLARNVIEELR
RVEGVGRVQLFGAEQAMRIWVDPNKLLSYGLTMNDLSTAITQQNAQIAPGRVGDAPTLPGQRVTIPLTVQGQLETPEQ
FAAIVLRAREDGSRVVLADVARVELGAQSYGFSVRENGKDATAAAVQLAPGANAVRTAAAIAQRMAELKPSMPAGMG
YSLPFNTAPFVKISIEQVIRTLAEAMVLVFLVMYLFLQNVRYTLIPAIVAPIALLGTFTVMLAAGFSINVLTMFGMVLAIGIIVD
DAIVVVENVERLMAQERLSPKAATTQAMKEITGAIVGITLVLSAVFIPMAFASGSVGVIYRQFTLSMAVSILFSAFLALTLTP
ALCATLLTPVADGHHEKRGFFGGFNRRFEQLTQHYAAGTRALLRRSGSMLLLFAALTGALAWIFRELPSDFLPQEDQGYF
MTSIQLPSDATEARTRDVVKTYEQHIASRPGIASNMSILGFGFSGSGPNAAMAFTTLKDWSARDGATAQGEVALAQQA
MSSIREGTAMTLMPPAIDELGTSSGFTLRLQDRSGQGYAALKAAETKLLTLAAQSHIVAGVYPDGLPPGSTVRLNIDRQKA
EALGVSFSTLSETLSAAIGSLYVNDFPNAGRMQQVIIQADAPARMQLDDVLRLYVRNTAGGMVSLKEVVTPVWSDTPLQ
LTRYQGFLAARISGSAAPGVSSGAAMAEMERLAAQLPPGFTVEWTGQSLQERQSAAQAPMLLLLSMVVVFLVLAALYES
WSIPLSVMLVVPLGLLGAVLAVILRDMPNDVFFKVGLITVIGLSAKNAILIVEFARQLRDQGHELVDAAVTAARLRLRPILMT
SLAFALGVVPLMVASGASAETQHAIGTGVFGGMVSATVLAVFFVPVFFVAVMSVQERYDRWREKRRTTHVNAPH

>AcrB_S_glossinidius
MSKYFIDRPIFAWVIAIIIMLAGALSILKLPIEQYPAIAPPTIRITATYPGADASTLQDSVTQIVEQNMNGIDHLMYMASDSDS
SGTATITLTFESGADPDIAQVQVQNKLQLAMPLLPQEVQQQGVQVQKSSASFLMVAGFISDDKNMTQEDIADYVGSSL
KDPISRTNGVGEVQLFGAQYAMRIWLDPNKLNNFQLTPVDVISALEVQNNQIAAGQLGGSPPVKGQQLNTSIIVQTRLK
TTDEFGKIQLKVNPDGSQVRLKDVATIALGGESYDIIARINGQPATGLGIKLATGANALDTANAVKAELGRLQANFPAGLK
VVYPYDTTPFVKISITEVVKTLVEAIILVFLVMYLFLQNFRATLIPTIAVPVVLLGTFAILGAFGYTINTLTMFACVLAIGLLVDDA
IVVVENVERVMAEEGLSPIEATRKSMGQIQGALVGIALVLSAVFVPMAFFGGSTGAIYRQFSITIVSAMILSVIVALVLTPALC
ATLLKPIKKGDHGKTTGFFGWFNKKFDQSTHHYTDSIGHILNSTGRYLLIYLLIVVGMALLFIRLPTSFLPDEDQGVFLTMTLL
PAGATQERTQHVLDEVTQYYLNNEKDNVRSVFTVNGFGFAGRGQNSGIVFVSLKDWGERSGAENKVPAITARANQAFS
KIKDGIVLAFNLPAIVELGTATGFDFELIDENNLGHEQLTAARNQLLGMVAQHPDVLQGVRPNGLEDTPEYKLDVDHEKA
EVLGVSLSDINTTIGAALGGSYVNDFIDRGRVKKVYVQGDAPYRMLPQDINNWYVRGSDNQMVPLSAVTTARWQYGS
PRLERYNGLPSMEIQGEAAPGKSTGEAMALMEQLAGKLPAGIGYDWTGMSYQERLSGNQAPALYAISLIVVFLCLAALYE
SWSIPFSVMLVVPLGVIGALLAATLRGLNNDVYFQVGLLTTIGLSAKNAILIVEFAKDLMEKEGKGLIEATLEAVRMRLRPIL
MTSLAFILGVLPLAISTGTGSGSQNAVGTGVMGGMVTATLLAIFFVPVFFVVVRRRFGKKGHQDELEQGHPVENPPPRP

>AcrB_Enterobacter
MPNFFIDRPIFAWVIAIIIMLAGGLAILKLPVAQYPTIAPPAVSITAVYPGADAKTVQDTVTQVIEQNMNGIDKLMYMSSNS
DSTGTVQITLTFESGTDADIAQVQVQNKLQLAMPLLPQEVQQQGVSVEKSSSSFLMVVGVINTNGTMTQEDISDYVGA
NMKDAISRTSGVGDVQLFGSQYAMRIWMDPNKLNNFQLTPVDVIAAIKAQNAQVAAGQLGGTPPVKGQQLNASIIAQ
TRLTSADEFSKILLKVNQDGSQVRLRDVAKVELGGENYDVIAKFNGQPASGLGIKLATGANALDTATAIRAELKKMEPFFPS
GLKIVYPYDTTPFVKISIHEVVKTLVEAIILVFLVMYLFLQNFRATLIPTIAVPVVLLGTFAILSIFGFSINTLTMFGMVLAIGLLVD
DAIVVVENVERVMAEEGLPPKEATRKSMGQIQGALVGIAMVLSAVFIPMAFFGGSTGAIYRQFSITIVSAMALSVLVALILT
PALCATMLKPIAKGDHGENKKGFFGWFNRMFDKSTNHYTDSIGGILRSTGRYLLLYIIIVVGMAFLFVRLPSSFLPDEDQGV
FLTMAQLPAGASQERTQKVLDEVTDYYLTKEKANVESVFAVNGFGFAGRGQNTGIAFVSLKDWSERSGDENKVEAITGR
AMGTFSQIKDAMVFAFNLPAIVELGTATGFDFQLIDQGGLGHEKLTQARNQLFGEIAKHPDLLVGVRPNGMEDTPQYKI
DIDQEKAQALGVSISDINTTLGAAWGGSYVNDFIDRGRVKKVYIMSEAQYRMLPNDINNWFVRGSDGQMVPFSAFSTS
HWEYGSPRLERYNGLPSMEILGQAAPGKSTGEAMNLMEELASKLPSGIGYDWTGMSYQERLSGNQAPALYAISLIVVFLC

LAALYESWSIPFSVMLVVPLGVIGALLAATFRGLTNDVYFQVGLLTTIGLSAKNAILIVEFAKDLMDKEGKGLIEATLEAVRM
RLRPILMTSLAFILGVMPLVISTGAGSGAQNAVGTGVMGGMITATVLAIFFVPVFFVVVRRRFSRKNEDVEHSHSVDHH

>AcrF_Enterobacter
MANFFIQRPIFAWVLAIILMIAGGLAILQLPVAQYPTIAPPAVAVTATYPGADAQTVQDTVTQVIEQNMNGIDNLMYMSS
TSDSAGNVTITLTFQSGTDPDIAQVQVQNKLQLAMPLLPQEVQQQGIGVEKSSSSFLLVAGFVSDNKTLTQDDISDYVAS
NVKDAISRTSGVGDVQLFGAQYAMRIWLDSNALNKFQLTPLDVINQLKIQNNQIAAGQLGGTPSIPGQQLNASIIAQTR
LKTPEEFGNVTLKVNQDGSMVHLKDVARIELGGENYNMVTKINGQAATGLGIKLATGANALDTAAAIKTKLAELQSFFPQ
GMRVVYPYDTTPFVKISIHEVIKTLFEAIVLVFLVMYLFLQNLRATLIPTIAVPVVLLGAFAILSAFGFSINTLTMFGMVLAIGLL
VDDAIVVVENVERVMVEDKLPPKEATQKSMEQIQGALVGIAMVLSAVFIPMAFFGGSTGAIYRQFSLTIVSAMALSVLVAL
ILTPALCATLLKPASEEHHAKGGFFGWFNTLFDKSVDHYSNSVSGILRKTGRYLVVYVLIVGGMAVLFLRLPTSFLPEEDQG
VFMTMVQLPAGATQTRTQQILDQVQQYYLTQEKANVESVFTVNGFSFSGQGQNSGIAFVSLKPWEDRPGEKNGVEAIV
GRATKAFSQIKDGLVFPFNLPAIIELGTATGFDFELIDQANLGHTSLTQARNQLLGMVKEHSDLLVRVRPNGLEDTPQFKL
DIDQEKAQALGVSVSDINQTISTALGGTYVNDFIDHGRVKKVYAQAAAQFRMLPGDINNLYVRSANGEMVPFSAFSSSR
WVSGSPRLERYNGMPSMEILGESAPGKSTGEAMVMMERLANKLPSGIGYDWTGMSYQERLSGNQAPALYAISLIIVFLCL
AALYESWSIPFSVMLVVPLGVIGALLGASLRGLNNDVYFQVGLLTTIGLSAKNAILIVEFAKDLMEKEGKGVVEATLEASRM
RLRPILMTSLAFILGVMPLVISNGAGSGAQNAVGTGVMGGMLSATLLAIFFVPVFFVVVRRRFTRQHH
>AcrD_Enterobacter
MANFFIDRPIFAWVLAIILCLTGTLAILSLPVEQYPDLAPPNVRITANYPGASAQTLENTVTQVIEQNMTGLDNLMYMSSQ
SSATGQATVTLSFTAGSDPDEAVQQVQNQLQSAMRKLPQAVQNQGVTVRKTGDSNILTIAFVSTDGSMDKQDISDYV
ASNIQDPLSRINGVGDIDAYGSQYSMRIWLDPAKLNSVQMTATDVTNAIKSQNAQIAVGQLGGTPSVDNQALNATINS
QSLLQTPQQFRDITLRVNPDGSEVRLGDVAKVEMGAEKYDNLSRFNGNQASGLGVKLASGANEMATAKLVIDRLDELA
PYFPHGLEYKVAFETTSFVKASIEDVVKTLLEAIALVFLVMYLFLQNFRATLIPTIAVPVVLLGTFAVLYAFGYSINTLTMFAMV
LAIGLLVDDAIVVVENVERIMTEEGLSPRDATRKSMGQIQGALVGIAMVLSAVFVPMAFFGGTTGAIYRQFSITIVSAMVLS
VLVAMILTPALCSTLLKPLHKGEQHGQKGFFGWFNRMFNRNAARYEAGVANILHRSLRWIVIYVLLLGGMVFLFLRLPTSF
LPLEDRGMFITSVQLPSGATQQQTLKVVQKVEQYFFTHEKDNIVSVFSTVGSGPGGNGQNVARMFVRLKGWDERDSKT
GSSFAIIERATKAFTRIKEARVFASSPAAISGLGSSAGFDMELQDHAGAGHDALMAARDRLLDLAGKDPSLTRVRHNGLD
DSPQLQIDIDQRKAQALGVSINDINDTLQTAWGSSYVNDFMDRGRVKKVYVQAAAPYRMLPDDINLWYVRNNAGGM
VPFSAFATSHWESGSPRLERYNGYSALEIVGEAAPGVSTGTAMDTMEKLVQQLPAGFGLEWTAMSYQERLSGAQAPAL
YALSLLVVFLCLAALYESWSVPFSVMLVVPLGVIGALLATWMRGLENDVYFQVGLLTVIGLSAKNAILIVEFANEMNSKGH
DLLSSTLHACRQRLRPILMTSLAFVFGVLPMATSSGAGSSSQHAVGTGVMGGMISATVLAIYFVPLFFVLVRRRFPLKERPE

>MexDYB_Enterobacter
MPHFFIERPVFAWVIALFIVLTGLLSIPRLPVAQYPAVAPPGIIISVSYPGASPDVMNTSVVSLIEREISGVDNLLYFESSSDTT
GMASITVTFKPGTDIKLAQMDLQNQIKIVEPRLPQAVRQNGLTVEAANSGFLMMVGLKSQTGEYQEADLSDYFARNLTD
ELRRVPGVGKVQLFGGEKALRIWLDPMKLHSHGLSVTDVLTAVSQQNVIVSPGRTGDEPAIPGQGVTYPITVRGQLSSVE
AFSQITIKSLTSGARLKLSDIARVESGLQSYAFGIRENGMPATAAAIQLSPGANAISTASGIRSRLAELSSVLPEGMEFTVPFD
TAPFVKLSIMKVVETFAEAMVLVFLVMLLFLHKIRCTLIPAIVAPVALLGTFTVMLLSGYSINILTMFGMVLAIGIIVDDAIVVV
ENVERLMEEKNLSPKEATREAMHEITPAIIGITLVLTAVFIPMGFASGSVGIIYRQFCISVAVSILLSAFLALTLTPALCATLLKP
HHVGQSKGKSFSAWFNTRFHTLTSFYASGLGFVLKRTGRMMMLYAALCVALYAGLSSLASSFLPDEDQGYFMSSIQLPS
DATMQRTLKVVKQFEDEIAQQQAIESNIMILGFGFSGSGPNSAMAFTTLKNWKKREGRSAQDEADHIQSKMESVPDAV
TMSLLPPAISDMGTSSGFTYYLQDRGGKGHQALKNAADELVQRANKSGQLSDVYIDGLPEGTSLTLNVDREKAEAMGVS
FDEINQTISVAMGSNYVNDYTNKGRVQQVIIQADAPYRMQPEHVLALSVKNRLGEMLPLSSFVTLSWNVAPQQLMRY
QGYPSIRITGSASAGESTGTAMNAMEMLAKDLPAGFAGEWAGSSLQEKESASQLPGLIVLSMLVVFMVLAALYESWSVP
FAVMLVVPLGLLGAVLAVTVANMTNDVFFKVGLITLIGLSAKNAILIIEFARQLIHQGHNVIEATLMASKQRLRPILMTSLAF
TLGVVPLMLAKGASDSTQHAIGTGVFGGMISGTLLAIFFVPVFFVVITRFIETRRKNRESKVQL

>MexFQ_Enterobacter
MDFSRFFIDRPIFAAVLSILIFITGLIAIPLLPVSEYPDVVPPSVQVRAEYPGANPKVIAETVATPLEEAINGVENMMYMKSVA
GSDGVLVTTVTFRPGTDPDQAQVQVQNRVSQAEARLPEDVRRLGITTQKQSPTLTLVVHLFSPGGKYDSLYMRNYATLK
VKDELARLPGVGQIQIFGSGEYAMRIWLDPNKVAARGLTASDVVTAMQEQNVQVSAGQLGAEPLPKESDFLISINAQGR
LHTEEEFGNIILKTAQDGSLVRLRDVARIEMGSGSYALRSQLNNKDAVGIGIFQSPGANAIDLSNAVRAKMDELSTRFPED
MKWAAPYDPTVFVRDSIRAVVQTLLEAVALVVLVVILFLQTWRASIIPLIAVPVSIVGTFGILYLLGFSLNTLSLFGLVLAIGIVV
DDAIVVVENVERNIEEGLAPLAAAHQAMREVSGPIIAIALVLCAVFVPMAFLSGVTGQFYKQFAVTIAISTVISAINSLTLSPA
LAALLLKPHGAPKDYPTRLIDRLFGWIFRPFNRFFHRSSNGYQGLVGKTLGRRGAVFAVYLLLLCAAGVMFKAVPGGFIPT
QDKLYLIGGVKMPEGSSLARTDAVIRKMSEIGMNTEGVDYAVAFPGLNALQFTNTPNTGTVFFGLKPFDQRKHTAAEINA
EINAKIAQIQQGFGFSILPPPILGLGQGSGYSLYIQDRAGLGYGALQNAVNTMSGSIMQTPGMHFPISTYQANVPQLDVQ
VDRDKAKAQGVSLTDLFGTLQTYLGSSYVNDFNQFGRTWRVMAQADGPYRDSVEDIANLRTRNNQGEMVPIGSMVNI
STTYGPDPVIRYNGYPAADLIGDADPRVLSSTQAMTQLEGMSKQILPNGMNIEWTDLSFQQATQGNTALIVFPVAVLLA
FLVLAALYESWTLPLAVILIVPMTMLSALFGVWLTGGDNNVFVQVGLVVLMGLACKNAILIVEFARELEIQGKGIMEAALE
ACRLRLRPIVMTSIAFIAGTIPLILGHGAGAEVRGVTGITVFSGMLGVTLFGLFLTPVFYVTLRKFVTRGKAERDVLPA

>MexFQ2_Enterobacter
MLTFFIKRPRFAMVIALVITLLGAIALKLIPVEQYPQITPPVVNVSASWPGASAADVAEAIATPLETQINGVDHMLYMESTSS
DEGAYSLNITFAAGTDADLAAIDVQNRVAQAVAQLPADAQQNGVQVRKRATNLMMGVSLYSPEQTHSPLFVSNYAST
QIREALARLPGVGQVQIFGARDFSMRIWLRPDRMNALNVTTDDISQALREQNVQGAAGQVGTPPVFNGQQQTLTING
LGRLNEADAFGDIIIRAGEMGQLVRLKDVANIELGSRSYSSGAQLNGHDSAYLGIYPTPSANALRVAEAVRGELERLSARFP
ADLTYEVKFDTTEFVAATIKEIGVSLALTLLAVVVIVSLFLQSWRATLIVAIAIPVSLIGTFAVLYALGYSANTLSLFAIILALTMV
VDDAIVVVENVETLMAEGQSRTAATALALRQIAGPVIATTLVLLAVFIPVALLPGIVGELYRQFAVTLSTAVTLSSLVALTLTP
ALCAMLLRPRPARPAAIFRGFNRGLDATRDAYARLVNVLNHRPLLAFIATAGAAAVVVFSFTAMPKAFLPQEDQGYFFAS
VQLPEAASLERTEAVMSTARQLIAANPAVADVIQVSGFNLLNGTSASNGGFISVMLKDWSERPPLDEVMGTLQRQLLAL
PEATIMTFAPPTLPGLGNASGFDLRIQAQAGQSPADLERVTREVLAKANQHPQLSRVFTTWSSNVPQLTLSVDRERAARL
DVPVSRIFSSLQTAFGGTRAGDFSINNRVYHVVMQNEMQWRERAEQISELYVRSNNGERVRLSNLVTITPTVGAPFLQQ
YNQFPSVSVSGSAAPGVSSSTAMAAMGQLLVDNLPAGYDYAWSGMSYQEQQTGNQAVWIVLAAVVMAWLFLVAQ
YESWTLPASVMLSVLFAIGGALTWLWMAGYANDVYVQIGLVLLIALAAKNAILIVEFARARRNEGMAIVDAAREGASRRF
RAVMMTAVSFIIGVLPMMLATGAGAQSRRIIGTTVFSGMLVATVVGIVFIPALFVLFQRLREWGHRLTGE

>MexB_A_borkumensis
MARFFIDRPIFAWVIAIIMMMAGALAIYTLPIEQYPTVAPPQVSIAGNYPGASAKTVEDSVTQVIEQQMNGIDNLLYMSSS
SDSFGNAAVNLTFAPGTDPDIAQVQVQNKLQLATPLLPQEVQQQGMQVTKSSDSFLMVAGFTSEDGSLSRADLADYV
ASNVQDPVSRVPGVGQIQLFGSPYAMRVWLDPNKLNKFDLTPQDVTQTIEVQNNQVASGQLGGAPAVEGQQLNATII
AQTRLEDVDQFENILLKVNPDGSRVFLKDVARVELAAQNYDVQGRYNGQPAAGLAISLATGANALDTAEALRARLAELQ
PYFPDKMEMVFPYDTTPFVSISIEEVVHTLFEAIILVFLVMYLFLQNFRATLIPTLAVPVVLLGTFAVLAAFGFSINTLTMFGMV
LAIGLLVDDAIVVVENVERVMHEEGLPPKEATRKSMGQITGALVGIALVLSAVFVPMAFFPGSTGAIYRQFSITIVSAMVLS
VLVALILTPALCATMLKAKDSEHQTDHGFFGWFNRTFDKSSRRYQGSVEKILGRRGRYLFIYVVIVGVLGFSFMRLPSSFLPE
EDQGILFTLVTLPAGSTQEQTVKVLEKMEDYYLNEEASAVDGLFTVAGFSFTGRGQNAGMAFVNLKDWSERDLSVDGA
DNVVARAMGYFSTIREAMMFALNPPSIPELGNASGFDFQLLDQSGQGHEALIQARNQMLGMAAQDPRLAGVRPNGLE
DSPQYQIDIDQQKAKALGLSISDINSTLQIAWGSSYVNNFVDRGRVKRVYVQADAPYRMLPENVNDWFVRNNQGKMV
PFSTFATGHWTYGSPKLERYNGVSSVNIQGNAAPGYSTGDAMDAMEELSAKLPAGFGFEWTGMSYQERQSGDQAPAL
YVISLLVVFLCLAALYESWSIPFAVMLVVPLGILGAVLAATFRDLNNDVFFQVGLLTTIGLSAKNAILIAEFALELEQKGEHLLK
ATLEAVRMRLRPILMTSLAFMLGVTPLMISTGAGAGARNAIGTGVFGGMLTATVLAIFFIPLFYVAVRKLSGVPLDGKKKGK
E

>AcrB_P_carotovorum

MAKFFIDRPIFAWVLAIMVMLTGLLAIVKLPIAQYPTIAPPAIDVTATYPGADASTLQDSVTQVIEQNMNGIDNLMYMSSS
SDSSGTVQITLTFEAGTDPDIAQVQVQNKLQLAMPLLPQEVQQQGVNVQKSSSSFLMVAAFISEDGKMSQEDIADYVA
ANVKDPISRTSGVGDAQLFGSQYAMRIWLDPNKLNNYQLTAGDVTAAIRVQNNQIAAGQLGGAPPVPGQQLNASIIA
QTRLNSAEEFSKILLKVNADGSQVRLKDVARVELGAEGYDVIARFNGKPAAGIGIKLATGANALDTATAVKEALTKAEEFFP
AGLKVVYPYDTTPFVKISINEVVKTLVEAIVLVFLVMYLFLQNFRATLIPTIAVPVVLLGTFAILSAFGYSINTLTMFAMVLAIGL
LVDDAIVVVENVERVMAEEGLPPKEATKRSMEQIQGALVGIALVLSAVFVPMAFTGGSTGAIYRQFSITIVSAMVLSVMVA
LILTPALCATLLKPIAKGDHSEKTGFFGWFNRLFEKSTHHYTDSVANILRSTGRYLVIYLLIVVGLALLFLRLPTSFLPDEDQGV
LLNIVQLPSGATQENTQKIMDRMTQYYLENEKGNVKSVFTVTGFGFSGRGQNAGLAFASLNDWSERSGAENKVMAIAG
RANAAFSQYKEAMVFAVNLPAIIELGTATGFDFQLIDQANLGHAKLTEARNQLLGMAAQRPDTLEQVRPNGMEDTPQF
RLDIDQEKAQALGVSLSDISSTLATTLGGSYVNDFIDRGRVKKVYVQADAPFRMLPDDIKNWYIRGSNGQMVPFSAFTQS
HWEYGSPRLERYNGQPSMQIQGQAAPGKSTGEAMALMESFVAQLPQGIGYEWTGMSYQERLSGNQAPAIYAISLIVVF
LCLAALYESWSIPFSVMLVVPLGIIGALIAANMTGLENDVYFKVGLLTTIGLSAKNAILIVEFAKDLMEKEGKGLIEATLDAVR
MRLRPILMTSLAFILGVMPLVISSGAGSGAQNAVGTGVMGGMITATVLAIFFVPVFFVVVRRRFGKKVDDTTAH

>AcrD_P_carotovorum
MANFFVDRPIFAWVLAILLSLCGMLAIKSLPLEQYPDLAPPSVRITASYPGASAQTLENTVTQVIEQSMTGLDNLMYMSSD
SSNTGQARVMLTFEAGTNPDEARQQVQNQLQSAIRKLPQEVQQQGVTVSKTGDTNILMVAFVSTDGSMDKQDIADY
VATNIQEPISRISGVGEVSAYGSQYAMRIWLDPAKLMDYALTTSDVVRAIESQNSQVSVGQVGGVPSVDNQALNATINA
QSLLQTPQQFRDITLRVNQDGSAVTLANVAEVELGAERYDFLSRFNGQSASGLGVTLASGANELETDKRVRERIDELSRYF
PHGMEAKIAFETSPFVKASITDVVKTLFEAVLLVFLVMYLFLQNFRATLIPTIAVPVVLLGTFAVLYACGFSLNTLTMFAMVL
AIGLLVDDAIVVVENVERVMSEEGLSPREATRKSMGQVQGALVGIALVLSAVFVPMAFFGGTTGAIYRQFSITIVTSMILSV
LVAMILTPALCATLLKPLAKGQHHGRKGFFGWFNRSFTRTSLKYERGVGKILMSSGRWLLLYMGIIGVMAFLFFRLPTSFLP
QEDRGVFTTQVQLPPGSTQQQTLQVVNKIEQYYLTQEKDTVTSVFSTIGSGPGGNGQNVARLFIRLKDWNERTTPESSSF
AVIERATKAFRHIQEARVFASSPPSINGLGSAAGFAMRLQDRGGLGHDALMAARDQLLNMADSNPELTRVRHNGLDDS
SQLRIHIDQRKAQALGVSVDDINSTLKTGWGSTYVNDFLDRGRVKKVYVQAAAKFRMLPDDISKWYVRNNRGGMVPFS
AFAQTVWETGSPRLERYNGYSSLEIVGEAMPGVSTGTAMTVMESLVAKLPEGFGLEWTGMSLQERLSGAQAPALYAISLL
VVFLCLAALYESWTVPFSVMLVVPMGVLGALVATWARGLENDVYFQVGLLTVVGLSAKNAILIVEFANEMNQKGKDLVE
ATLEASRQRLRPILMTSLAFIFGVLPMATSSGAGSASQHAVGTGVIGGMLAATFLAIFFVPLFFVVVRRRFPLKEKTLE

>AcrB_E_tasmaniensis
MAKFFIDRPIFAWVIAIIIMLAGALSILKLPIEQYPNVAPPAIEITAAYPGADAKTLQDSVTQVIEQNMNGIDGLMYMSSSSD
SSGALQLTLTFESGTNADIAQVQVQNKLQLATPLLPQEVQQQGIKVQKSSSSFLMVAGFINDNDTMTQNDIADYVGSNI
KDPISRVTGVGDTQLFGAQYAMRIWMDPHKLNNYQLTPVDVISAINTQNAQVAAGQLGGSPPVKGQQLNASIIAQTRL
TSPEEFGKILLKVNTDGSRVRLSDVAHIELGGENYEIIARFNGKPASGLGIKLATGANALDTASAVKAELSKLEPFFPSGLKVV
YPYDTTPFVKISINEVVKTLVEAIVLVFLVMYLFLQNFRATLIPTIAVPVVLLGTFAIINTFGYSINTLTMFGMVLAIGLLVDDAI
VVVENVERVMAEEGLPPKEATRKSMDQIQGALVGIALVLSAVFIPMAFFGGSTGVIYRQFSITIVSAMVLSVIVALVLTPALC
ATMLKPIAKGEHGKTTGFFGWFNRMFDKSTHHYTDSVGHIVRSTGRYLLIYLLIVVGMAFLFLRLPSSFLPEEDQGVFLTMA
QLPAGATQERTEKVLDQVTDYYLNNEKANVNSVFTVNGFGFAGRGQNTGIAFISLKPWDERPGAENKVPAIAGRAMTA
LSAIKDAMVIPFNLPAIVELGTATGFDFQLVDQGGLGHEKLTAARNQLLGMIAQHPDTLVGVRPNGLEDTPQFKLSIDQE
KAQALGVSLSNINTTLAAAWGGSYVNDFIDRGRVKKVYVMAAAPYRMLPDDIGNWYVRATNGEMVSFNAFSSAQWV
YGSPRLERYNGLPSMELLGQPAAGKSSGEAMNLMQELAGKLPTGIGYDWTGMSYQERLSGNQAPALYAISLIVVFLCLA
ALYESWSIPFSVMLVVPLGVIGALIFTTLRGLSNDVYFVVGLLTTIGLSAKNAILIVEFAKDLMDKEGKGLIEATLEAVRMRLR
PILMTSLAFILGVLPLAISSGAGSGAQNAVGTGVMGGMVTATVLAIFFVPVFFVVVRRRFGKNKEDGERSHAVDNH

>AcrD_E_tasmaniensis
MANFFIDRPIFAWVLAILLCLTGGLAIVSLPIEQYPDLAPPNVRITANYPGASAETLENTVTQVIEQNMTGIDNLMYMSSQS
SNTGQATVTLTFEAGTNPDEARQQVQNQLQTAMRKLPQDVQSQGVTVNKTGDTNILMVAFVSTDGSMDRQDISDYV

ASHLQDPLSRINGVGQVDAYGSQYAMRIWLDPDKLIDYALTTTEVVDAIKSQNSQIAVGQLGGLPSVPSQALNATINSQ
SLLQTPEQFRAITLRVNTDGSVVTLGQVARIELGAEKYDYLSRYNGMDASGLGIKLASGANELQTDTLVRARLDELSRFFPH
GLKAEIAFETSPFVKASIEDVVKTLFEAVLLVFLVMYLFLQNFRATLIPTIAVPVVLFGTFGVLYAFGYSINTLTMFAMVLAIGLL
VDDAIVVVENVERIMTEEGLSPRDATRKSMGQIQGALVGIALVLSAVFVPMAFFGGTTGAIYRQFSVTIVSAMVLSVLVAM
ILTPALCSTLLKPLAQGSHHGRRGFFGWFNRMFNHNAERYKDGVGKMLAKGGRWLLVYLALIGVMALLFIRLPGSFLPLE
DRGIFTTQVQLPPGSTLQQTMKVVEKVEHYYLTHEQNNVLSVFATVGAGPGGNGQNVARLFVRLKDWQDRHGSDNSS
FAIIERATRAFRSINEAKVVASSPAAITGLGNSAGFDMELQDNAGLGHRALMSARNTLLDMAQKNPSLTRVRHNGLDDS
SQLRIDIDQRKAQALGISLDDINATLQTGWGSSYVNDFLDRGRVKKVYVQADAPFRMLPDDISKWYVRNDSGGMVPFT
AFARSHWESGSPRLERYNGYSALEIVGESATGVSNGAAMDVMEKLVAQLPVGISLQWTGASLQERMSGAQAPALYALS
LLVVFLCLAALYESWSIPFSVMLVVPLGVLGALIATWLRGLENDVYFQVGLLTVIGLSAKNAILIVEFANEINARGQELVAAT
LEASRQRLRPILMTSLAFIFGVLPMAISQGAGSSSQHAVGTGVLGGMISATVLAVFFVPLFFVMVRRRFPLKDKIR

>MexB_P_fluorescens
MSKFFIDRPIFAWVIALVIMLVGALSILKLPINQYPSIAPPAIAIQVTYPGASAQTVQDTVVQVIEQQLNGIDNLRYVSSESN
SDGSMTITATFEQGTNSDTAQVQVQNKLNLATPLLPQEVQQQGIRVTKSVRNFLMVIGVVSRDGSMTREDLSNYIVSN
MQDPISRTAGVGDFQVFGAQYAMRIWLDPAKLNNFNLTPVDVSTAITAQNVQIASGQLGGLPAMPGQQLNATIIGKTR
LQTAEQFKAILLKVNPDGSQVRVGDVADVALGGENYSINAQFNGAPASGLAVRLATGANALDTAKALRKTVDDLKPFFP
QGLEVVFPYDTTPVVSESIKGVVETLVEAIVLVFLVMFLFLQNFRATIITTMTVPVVLLGTFGILAAFGFSINTLTMFGMVLAIG
LLVDDAIVVVENVERVMSEEGLSPKEATKKSMGQIQGALVGIALVLSAVLLPMAFFGGSTGVIYKQFSITIVSAMALSVLVA
LIFTPALCATMLKPIPKGEHGIPKRGFFGWFNRTFDRGVRSYERGVGNMLKHKAPYLLAYIIIVVGMVWLFTRIPTAFLPEED
QGVLFAQVQTPAGSSAERTQVVVDKMREFLLRPSKDGGEGDGVASVFTVTGFNFAGRGQSSGLAFIMLKPWEERNADN
TVFKIAGRAQQHFFSFRDAMVFAFAPPAVMELGNATGFDVFLQDRAGIGHDKLMEARNQFLGMAAQSKILAQVRPNG
LNDEPQYQLEIDDEKASALGITLSEINNTLSIALGSSYVNDFIDRGRVKRVYVQGQPNARMSPEDLQKWYVRNSAGTMVP
FTAFAKGEWVYGSPKLARYNGVEAMEILGTPAPGYSTGEAMAEVEAIAKKLPAGVGISWTGLSYEERLSGSQAPALYALSL
LMVFLCLAALYESWSIPIAVMLVVPLGIIGALMATSLRGLSNDVYFQVGLLTTIGLAAKNAILIVEFAKELHEQGRTLVEAAVE
ACRMRLRPIIMTSLAFVLGVVPLAISTGAGSGSQHAIGTGVIGGMLTATILAIFWVPLFFVTVSSIGRRKNTDEHDTPETSKE
AGQ

>AcrDMexB_P_fluorescens
MARFFIDRPIFAWVIAIVIMLAGALSISQLPLEQYPDIAPPTVRISATYTGASAKTVEDSVTQVIEQQMKGLDNLTYMSASSS
SDGSASISLTFTAGTNPDVAQMQVQNKLQQAESRLPQPVQSEGLTVTKGGSDFLMIAALASDNPSVTGTQIGDYISSTLL
DSISRIDGVGDVQTLGSGYAMRIWLDPTLLEKYALMPSDVSTALEAQNTEVSAGQLGAMPAVKGQQLNATISARSKLQT
VEEFRNVVVKSTSDGAVVLLGDVARVELGSESYDVSSALNGKPAAAMGVQLAAGANALNVGEAVKAKLKELEPFYPTEM
QLKNVIAYDTTPFVSLSIEEVVKSLGEAIVLVVLIMFLFLQNLRATLIPAITVPVVLLGTFGVLALFGYSINTLTMFAMVLAIGLL
VDDAIVVVENVERVMGEQGLSALDATRRSMDEITSALVGIALVLSAVFIPMAFFGGSTGVIYRQFSVTIVSAMVLSVLVAM
TLTPALCATLLKPSDGQGHGAQRGFFGWFNRTFERAAEAYKRQVGGILQRGRVGWLVYGLVLLVMAAGYFSLPTSFLPD
EDQGILMAQVQLPVGATDSRTQAVVKQFESYLLEQPEVEALISISGLGMNGNSQNSARAFIRLKDWSERTGAGQDAASF
AQRATMALSSIGDANVFVMQPPAVRGLGQTSGFDLQLKDLAGLGHDTLVAAREKLIELANQDPRLLGVRSNGLDDAPQ
LKVSIDDRKAGALSLSTSDINTTLSTALGGTYVNDFLNQGRVKKVYVQGEAAARMQAADLEHWFVRNSNDEMVPFSSFA
SSSWSYGSPLLERYNGNASLEVVGDPAPGVSSGDAMDAVEALVKQLPEGIGYEWTGQSYQLRLSGSQAPLLYGISVLFVF
LCLAALYESWSVPFSVMLVVPLGVVGAVLATRVSGLSNDVYFQVGLLTTVGLAAKNAILIVEFAKHLQEQGNSLREATLIA
VRQRLRPILMTSLAFMFGVLPLALSSGAGSAGRQAIGTGVLGGMLSATVLGIFFVPLFFVQIRRRFGRVSTASTSVPSAQSG
DA

>MexDY_P_fluorescens
MPQFFIDRPVFAWVVALFILLAGILAIPQLPVAQYPVVAPPQIEIYTVYPGASAQTVDESVVSLIEEELNGTDNLLYFESQSSL
GSATITATFQPGTNPELAQVDVQNRLKAVESRLPQAVTQQGLQVDKVSAGFLLLITLTSSDGKLDDVALSDYLARNVMN

EIKRLDGVGKAQLYGAERAMRIWIDPQKLVGFNLTPADVNAAIVAQNAQVSAGSLGDLPSRSTQEITATIVVKGQLNTPQ
EFADIVLKANPDGSTVRIADVARVEIGSQEYQFSTRLNGKPSTAVAVQLSPGANALNTATLVRNKMDELSRYFPAGVEYKI
PYDTSPFVKVSITKVIYTLGEAMLLVFAVMFLFLQNIRYTLIPTLVVPVALMGTFATMLALGFSINVLTMFGMVLAIGILVDD
AIVVVENVERIMAQEGLSPKEATRKAMGQITGAIIGITLVLVAVFIPMAFMQGSVGVIYQQFSLSMATSILFSAFLALTLTPA
LCATLLKPVAKGDHHAKTGFFGLFNRGFDRFGERYQGWVAYALKRSGRYLLIYGVLLVGMGLLFSRLPSSFLPVEDQGYTI
TDIQLPPGASKNRTVQVVEQIEAHNAGEPGVGDSTIILGFSFSGSGQNAALAFTTLKDWSQRGADDSAASIADRANLALG
QIKDAMAFSVLPPPVDGLGTSSGFEFRLQDRGGLGHATLMQARSELLAAADKSPMLMNVRESALAEAPQVQLEVDRKQ
ANALGISFADVGSVLSTAVGSAYINDFPNQGRMQRVVVQAEGDQRSQVEDLLKIHVRNNSGKMVPLSAFVQARWSQG
PAQLTRYNGYPAVSISGEPAPGYSTGQAMAEIERLVALGPTGLGQEWTGLSLQERLSGSQAPILLGLSLLVVFLCLAALYES
WSIPTSVLLVVPLGVLGAVLAVSLRGMPNDVFFKIGLITIIGLSAKNAILIIEFAKSLYDEGHDLIDATLQAARLRLRPIIMTSLA
FILGVVPLAIATGASSASQQAIGTGVIGGMISATLAVVFVPVFFVVVMKRVRRVDPKG

>MexFQ_P_fluorescens
MKFSQFFISRPIFAAVLSLLILIAGAISLFQLPISEYPEVVPPTVVVRANFPGANPKVIGETVAAPLEQAITGVENMLYMSSQST
ADGKITLTITFALGTDLDNAQVQVQNRVTRTEPKLPEEVTRIGITVDKASPDLTMVVHLTSPDKRYDMLYLSNYALLNIKDE
LARLGGVGDVQLFGMGDYSLRVWLDPNKTASRNLTATDVVTAIREQNRQVAAGALGAPPAPNAQAFQLSINTQGRLV
NEEEFENIIIRSGANGEITRLKDIARVELGSSQYALRSLLDNQPAVAIPIFQRPGSNAIQISNDVREKMDELKKGFPAGMDYSI
VYDPTIFVRGSIEAVVHTLFEALILVVLVVILFLQTWRASIIPLVAVPVSLIGTFAVMHLFGFSLNALSLFGLVLAIGIVVDDAIV
VVENVERNIELGLTPVEATKRAMGEVTGPIIATALVLCAVFVPAAFISGLTGQFYKQFALTIAISTVISAINSLTLSPALAAVLLK
GHDAPKDRFSKFLDKVFGGWLFRPFNRFFERASHGYVGTVARVIRSSGIALVLYAGLMVLTFFGFSNTPTGFVPGQDKQYL
VAFAQLPDAASLDRTEDVIKRMSDLALKQPGVESAVAFPGLSINGFTNSPNAGIVFVTLKPFDERKDPSMSAGAIAGALN
GQYAGIQEAYMAIFPPPPVQGLGTIGGFRLQIEDRGNLGYDELYKETMNIITKSHNVPELAGLFTSYTVNVPQVDAAIDRE
KAKTHGVAVSDIFDTLQIYLGSLYANDFNRFGRTYQVNVQAEQQFRLESDQIGQLKVRNNRGEMIPLATFIKVSDTSGPD
RVMHYNGFITAEINGAAAPGYSSGQAEKAIEKLLKDELPNGMTYEWTDLTYQQILSGNTALFVFPLCVLLAFLVLAAQYES
WSLPLAVILIVPMTLLSAITGVILSGGDNNIFTQIGLIVLVGLACKNAILIVEFAKDKQLEGMNPLAAVLEACRLRLRPILMTSF
AFIMGVVPLVFSSGAGAEMRHAMGVAVFSGMLGVTFFGLLLTPVFYVLIRNFVERSEARKAARALKLEAQQ

>MexIW_P_fluorescens
MAFTDPFIRRPVLATVVSLLIVLLGFQAWSKLPLRQYPQMENALITVTTAYPGANAETIQGYITQPMQQSLASAEGIDYMT
SVSRQNFSVISVYARIGSNSDRLFTELLAKANEVKNQLPQDAEDPVLSREAADASALMYISFFSKELNNPQITDYLSRVIQP
KLATLPGMAEAEILGNQVFAMRLWLDPVKLAGFGLTAADVTNAVRQHNFLSAAGEVKGEYVVTSINANTELKSAEAFGAI
PLKTDGDSRVLLRDVARVEMGAENYDTISSFGGTPSVYIGIKATPGANPLDVIKEVRKIMPDMEAQLPTNLKAEIAYDATLF
IQASIDEVVKTLFEAVLIVIVVVFLFLGALRSVVIPVVTIPLSMIGVMFFMQLMGYSMNLLTLLAMVLAIGLVVDDAIVVVENI
HRHIEEGKTPFDAAIEGAREIAMPVVSMTITLAAVYAPIGLLEGLTGALFKEFALTLAGAVVISGIVALTLSPMMCALLLRHDE
NPSGLAHRLDMVFERLKSRYQRMLHGTLNTRPVVLVFAVIVLLLIPVLIMFTKSELAPDEDQGIIFMMATAPQPTNLDYLN
TYTDHFITIFKEFPEYYSSFQINGYNGVQSGIGGFLLKPWNERSRTQMEILPEVQAKLESVPGLQIFGFNLPSLPGTGEGLPF
AFVINSPKDYATLLEVADRVKKRALESGKFAFMDIDLAFDKPEVVVDIDRAKAAQMGVSMQDLGGTLATLLGEAEINRFTL
EGRSYKVIAQVERPFRDNPDWLNNYYVKNAKGESLPLSTLIKVSDRARPRQLNQFQQLNAVTISGFPIVSMGEAIETVRQI
AREEAPVGFAFDYAGASRQFVQEGSALWVTFALALAIIFLVLAAQFESFRDPLVILVTVPLSICGALIPLFLGWSSMNIYTQV
GLVTLIGLISKHGILIVEFANQLRREQNLTPREAVEQAASIRLRPVLMTTAAMVFGMVPLIFATGAGAVSRFDIGMVIATGM
SIGTLFTLFVLPCVYTLLAKPDKS

>MexB_P_putida
MSKFFIDRPIFAWVIALVIMLVGALSILKLPINQYPSIAPPAIAIAVTYPGASAQTVQDTVVQVIEQQLNGIDNLRYVSSESNS
DGSMTITATFEQGTNPDTAQVQVQNKLNLATPLLPQEVQQQGIRVTKAVKNFLLVIGLVSEDGSMTKDDLANYIVSNM
QDPISRTAGVGDFQVFGAQYAMRIWLDPAKLNKFQLTPVDVKTAVAAQNVQVSSGQLGGLPALPGTQLNATIIGKTRL
QTAEQFESILLKVNKDGSQVRLGDVAQVGLGGENYAVSAQFNGKPASGLAVKLATGANALDTAKALRETIKGLEPFFPPG

VKAVFPYDTTPVVTESISGVIHTLIEAVVLVFLVMYLFLQNFRATIITTMTVPVVLLGTFGILAAAGFSINTLTMFAMVLAIGLL
VDDAIVVVENVERVMSEEGLPPKEATKRSMEQIQGALVGIALVLSAVLLPMAFFGGSTGVIYRQFSITIVSAMGLSVLVALIF
TPALCATMLKPLKKGEHHTAKGGFFGWFNRNFDRSVNGYERSVGAILRNKVPFLLAYALIVVGMIWLFARIPTAFLPEEDQ
GVLFAQVQTPAGSSAERTQVVVDQMREYLLKDEADTVSSVFTVNGFNFAGRGQSSGMAFIMLKPWDERSKENSVFALA
QRAQQHFFTFRDAMVFAFAPPAVLELGNATGFDVFLQDRGGVGHEKLMEARNQFLAKAAQSKILSAVRPNGLNDEPQ
YQLTIDDERASALGVTIADINNTLSIALGASYVNDFIDRGRVKKVYIQGEPSARMSPEDLQKWYVRNGAGEMVPFSSFAKG
EWTYGSPKLSRYNGVEAMEILGAPAPGYSTGEAMAEVERIAGELPSGIGFSWTGMSYEEKLSGSQMPALFALSVLFVFLCL
AALYESWSIPIAVVLVVPLGIIGALIATSLRGLSNDVYFLVGLLTTIGLAAKNAILIVEFAKELHEQGRSLYDAAIEACRMRLRPII
MTSLAFILGVVPLTIASGAGAGSQHAIGTGVIGGMISATVLAIFWVPLFFVAVSSLFGSKEPEKDVTPENPRYEAGQ

>MexDY_P_putida
MPQFFIDRPVFAWVVALFILLAGALAIPQLPVAQYPNVAPPQVEIYAVYPGASAATMDESVVSLIEQELNGADNLLYFESQ
SSLGSATITATFAPGTHPDLAQVDVQNRLKVVESRLPRPVTQQGLQVEKVSTGFLLLATLTSEDGKLDETALSDILARNVM
DEIRRLKGVGKAQLYGSERAMRIWIDPRKLIGFNLTPNDVAEAIAAQNAQVAPGSIGDLPSRSTQEITANVVVKGQLSSPD
EFAAIVLRANPDGSTVTIGDVARVEIGAQEYQYGTRLNGKPATAFSVQLSPGANAMETATLVRAKMQDLARYFPEGVKY
DIPYDTSPFVKVSIEQVINTLFEAMLLVFAVMFLFLQNLRYTLIPTLVVPVALMGTFAVMLAMGFSVNVLTLFGMVLAIGILV
DDAIVVVENVERIMAEEGLPPKQATRKAMGQISGAIVGITLVLVAVFLPMAFMQGSVGVIYQQFSLSMAVSILFSAFLALSL
TPALCATLLKPVAKGEHHERKGFFGWFNRRFESMSNGYQRWVVQALKRSGRYLLVYAVLLAVLGYGFSQLPTAFLPTED
QGYTITDIQLPPGASRMRTEQVAAQIEAHNAEEPGVGNTTLILGFSFSGSGQNAALAFTTLKDWSERGADDSAQSIADRA
TMAFTQLKDAIAYSVLPPPIDGLGESTGFEFRLQDRGGMGHAELMAARDQLLESASKSKVLTNVREASLAESPQVQLEIDR
RQANALGVSFADIGTVLDVAVGSSYVNDFPNQGRMQRVVVQAEGDQRSQVEDLLNIHVRNDSGKMVPLGAFVQAR
WVSGPVQLTRYNGYPAVSISGEPAAGYSSGEAMAEVERLVAQLPAGTGLEWTGLSLQERLSGSQAPLLMALSLLVVFLCL
AALYESWSIPTAVLLVVPLGVLGAVLAVTLRGMPNDVFFKVGLITLIGLSAKNAILIIEFAKHLVDQGVDAADAAVQAARLR
LRPIVMTSLAFILGVVPLAIASGASSASQQAIGTGVIGGMLSATLAVVFVPVFFVVVMRLSGRRQAHDSDGQPVPRES

>MexDY2_P_putida
MSRFFIHRPNFAWVVALFISLAGLLVIPSLPVAQYPNVAPPQISITASYPGASAKVMVESVTSIIEQSLNGAKGLLYYESTNN
SNGVAEVMVTFEPGTDPDMAQVDVQNRLKQAEARMPQAVLTQGLKVEQASSGFLLIYALTSTAGNRGDTTALADYAA
RNINNELLRVPGVGKLQFFASEAAMRVWVDPQKLVGYGLSIDDINSAIRGQNVQVPAGSFGSTPGASEQELTATLAVQG
TLDTPEAFAGIVLRANPDGSSVRLGDVARMAIGSENYNLSARLNGHPAVAGAVQLAPGANAIQTATLVKERLAELSQFFP
EGVEYSVPYDTSRFVDVAIEKVIHTLIEAMVLVFLVMFLFLQNVRYTLVPSIVVPVCLLGTLMIMKLLGFSVNMMTMFGMV
LAIGILVDDAIVVVENVERLMAEEGLSPVEATIKAMGQVSGAIIGITLVLAAVFLPLAFMSGSVGVIYQQFSVSLAVSILFSGF
LALTFTPALCATLLKPVPHGHHEKAGFFGAFNRGFARVTERYSLLNSELVARAGRWMLAYVGILVVLGYSYLRLPEAFVPAE
DLGYSVVDVQLPPGASRVRTDHTAEALEKFLMSRDAVANSFIVSGFSFSGQGDNAALAFPTFKDWSQRDKAQSAEAETA
AINAQFAANGDGAITAVMPPPIDGLGNSGGFALRLMDRGGLGREALLAARDQLLARANGNPVILYAMMEGLAEAPQLR
LHIDREKARALGVSFEAINSTLATAFGSAVINDFTNAGRQQRVVVQAEQGERMTPESVLRLYAPNANGEQVPFSAFVTTQ
WEEGPVQLVRYNGYPSIRIAGDASPGHSTGQAMAEMERLVSELPPGIGYAWTGLSYQEKVSSGQAASLFALAILVVFLLLV
ALYESWAIPLTVMLIVPIGALGAVWAVTLTGMPNDVYFKVGLITIIGLAAKNAILIVEFAKELWEKGYSLCDAAIEAARLRFRP
IVMTSMAFILGVVPLAIASGAGAASQRAIGTGVIGGMLSATLLGVVFVPVCFVWVLTLLKRKPSPVQQAVEEAE

>MexF_P_putida
MNFSKFFITRPIFAAVLSLVLLIAGSISLFQLPISEYPEVVPPTVVVRANFPGANPKVIGETVAAPLEQAITGVENMLYMSSQS
TADGKLTLTITFALGTDLDNAQVQVQNRVTRTQPKLPEEVTRIGITVDKASPDLTMVVHLTSPDNRYDMLYLSNYAILNIK
DELARLGGVGDVQLFGMGDYSLRVWLDPNKTASRNLTASDVVAAIREQNRQVAAGQLGAPPAPGSTSFQLSINTQGRL
VNEEEFENIIIRAGADGEITRLKDIARVELGSSQYALRSLLNNQPAVAIPIFQRPGSNAIEISDEVRAKMAELKKDFPEGMDYS
IVYDPTIFVRGSIEAVVHTLFEALVLVVLVVILFLQTWRASIIPLLAVPVSLIGTFAVMHLFGFSLNALSLFGLVLAIGIVVDDAIV
VVENVERNIGLGLKPLEATQKAMSEVTGPIIATALVLCAVFVPAAFISGLTGQFYKQFALTIAISTVISAFNSLTLSPALAAVLL

KDHHAPKDRFSRFLDKLLGSWLFSPFNRFFDRASHSYVGGVRRVIRSSGIALFVYAGLMGLTYLGFSSTPTGFVPAQDKQY
LVAFAQLPDAASLDRTEAVIKRMSEIALKQPGVADSVAFPGLSINGFTNSPNSGIVFTPLKPFDERKDPSQSAAAIAAALNA
QFADIQDAYIAIFPPPPVQGLGTIGGFRLQIEDRGNLGYEALYKETQNIIAKSHNVPELAGLFTSYQVNVPQVDAAIDREKA
KTHGVAITDIFDTLQVYLGSLYTNDFNRFGRTYQVNVQAEQQFRLDAEQIGQLKVRNNLGEMIPLATFLKVSDTSGPDRV
MHYNGFITAEINGAAAPGYSSGQAEAAIEKLLKEELPNGMTFEWTDLTYQQILSGNTALLVFPLCVLLAFLVLAAQYESWS
LPLAVILIVPMTLLSAITGVIVSGGDNNIFTQIGLIVLVGLACKNAILIVEFAKDEQAKGLDPLAAVLEACRLRLRPILMTSIAFI
MGVVPLVFSSGAGSEMRHAMGVAVFSGMIGVTVFGLFLTPVFFFLIRRFVERRQARKAEHAQVLENHA

>MexW_P_putida
MAFTDTFIQRPVLASVVSLLILLLGFQAWNKLQIRQYPQMENALITVTTAYPGANAETIQGYITQPLQQSLASAEGIDYMT
SVSRQNFSIISIYARIGADSDRLFTELLAKANEVRNKLPQDSEDPVLSKEAADASALMYVSFYSKEMSNPQITDYLSRVIQPK
LATLPGMAEAEILGNQVFAMRLWLDPVKLAGFGLSATDVTNAVRRYNFLSAAGEVKGEYVVTSVNASTELKSAEAFAALP
LKTSGDSRVLLGDVARVEMGAENYDTVSSFDGTPSVYIGIKATPAANPLDVIKEVRRIMPELESQLPSALNVSIAYDATRFIQ
ASIDEVIKTLGEAVLIVIVVVFLFLGALRSVLIPVVTIPLSMIGVLFFMQMMGYSLNLLTLLAMVLAIGLVVDDAIVVVENIHR
HMEEGKSPFDAALEGAREIAMPVVSMTITLAAVYAPIGFLTGLTGALFKEFALTLAGAVIISGIVALTLSPMMCALLLRREQN
PSGLAHRLDQLFERLKGRYQRLLHATLDSRPVVLVFAVIILCLIPVLLKFTQNELAPNEDQGVIFMMSSSPQPANLDYLNAY
TDQFTPLFKAFPEYYSSFQINGFNGVQTGIGGFLLKPWNERERTQMELLPLVQAKLEEISGLQIFGFNLPSLPGTGEGLPFQF
VINTAGDYPALLDVAQRIKTRAQESGKFAFLDIDLAFDKPEVVVDIDRAKAAQMGVSMDTLGGTLATLLGEAEINRFTLEG
RSYKVIAQVERPYRDNPGWLNNYYVKNDQGQLLPLSTLITLTDRARPRQLNQFQQLNAAIIQGVPMVSLGEALKTVQDI
AREEAPEGFAFDYAGAARQYVQEGSALWVTFGLALAIIFLVLAAQFESFRDPLVILVTVPLSICGALLPLFLGVSSMNIYTQV
GLVTLIGLISKHGILIVEFANQLRDERGLSVREAIEEAAAIRLRPVLMTTAAMVFGMVPLILASGAGAVSRFDIGMVIATGMSI
GTLFTLFVLPCIYTLLAHKASAKAPAIA

>MexI_P_putida
MRFTDVFVRRPVLALVVSSLIILMGLFAMGKLPIRQYPLLESSTITISTEYPGASAELMQGFVTQPITQAVSSVEGIDYLSSSS
QQGRSLITLRMVLNRDSTQALAETMAKVNQVRYRLPEKAYDPVVELSAGDSTAVAYVGFASDSLSIPELSDYLSRVVEPQ
FSGIDGVAKVQSFGGQRLAMRLWLDSEQMAGRGVTAADVAQAVRANNYQATPGQVRGQYVLADIQVDTDLTRVEDF
RELIIRNDGTDLVRLRDIGTVELSAAATQTSATMDGKPAVHLGLFPTPSGNPLVIVEGIRQLLPQIQQTLPPGVYVALAYET
ARFIDASIHEVLRTLVEAMLIVVLVIWLCLGSLRSVLIAVVAIPLSMLGAAGLMLMFGFSLNLLTLLAMVLAIGLVVDDAIVVV
ENVHRHIEEGKSPIAAALAGAREIAGPVIAMTLTLAAVYAPIGLMGGLTGTLFREFALTLAGAVIVSGIVALTLSPVMSSLLLQ
PGQQHGAMAAIADRLFGTLSGVYGRVLAYTLAHRWISGGVALLVCLSLPWLYLLPQRELAPPEDQAAVLTAIKSPQHASL
EYAERFALKLDQVMKSIAETTDTWIINGTDGPAASFGGINLSAWQARERSAAQVQAQLQQAVADIEGSSIFAFQVASLPG
SSGGLPVQMVLRSAQDYPELFQTMEVLKQRARDSGLFAVVDSDLDYNNPVVKVRVDRAKAASLGISMQAIGESLGVLV
GEQYLNRFALFGRSYDVIPQSIQDQRLTPAALSRQYVRAEDGSLVPLATLVRLDIEVAPNRLLQFDQQNASTLQAIPAPGV
SMGNAVAFLEQLTAELPPGFSHDWQSESRQYVQEGFALMWAFLAALVVIYLVLAAQYESLVDPLIILVTVPLSICGALLPL
ALGWATLNIYTQIGLVTLIGLISKHGILMVAFANEIQVRDNLDRAAAIVRAAQIRLRPVLMTTAAMTFGVLPLLFASGAGAN
SRFGLGVVIVCGMLVGTLFTLFVLPTIYAWLARDHRVSTARARQLVEAERQLGAL

>AcrF_T_auensis
MSRFFIDRPIFAWVIAIVIMLAGALAIKSLPIEQYPTIAPPSVRISTSYPGASAQTVEDSVTQVLEQNMTGIDNLLYMSSQSD
SSGNVTVSLSFLAGTNADTAQVQVQNKVQQALSSLPQTVQDQGVRVEKSTESFLMVAGLISENGEMDQSDISDYMVT
NIKEPLSRIKGVGTVQIFGSQYAMRVWLDPNKLNSFNLTPGDVTSAITSQNTQISVGQLGATPAVAGQQFNATITGQGRL
TSMDEFKNILLKVNTNGSQVRLRDVARVELGAESYNAVSRYNGKPASGIAIRLASNANALDTAELVKAKMADLSKYFPTG
LKVVYPFDTTTFVKISISEVAQTLVEAVILVFFIMYLFLQNFRATLIPTIAVPVVLLGTFGIMSVFGYTINTLTMFGLVLAIGLLVD
DAIVVVENVERVMSEEGLSPLEATRKSMDQITGALIGIALVLSAVFVPMAFFSGSTGAIYRQFSLTIVSAMALSVVVALILTP
ALCATMLKPVPKGDHGIQTGFFGWFNHMFERNSRRYRNTVGRILQRSGRMLVIYVVLLLGLVWLFTRMPTSFLPEEDQGI
ALTMVQLPTGATQERTLKVLDKVSDHFTTAEKKNVESVFTVAGFSFAGSGQNMGLAFVKLKDWSERKASDDAASAIIGR

AMGAFSQIKDASIFAFNMSSIPALGNSSGFDLYLQDRGGLGHEKLIEARNQLLGMAAKEPSLTRVRPNGMEDTPQYNINI
DYEKAMALGLTVSDINSTLSTAWGSNYVNDFIDRGRVKKVYVQADAPFRMQPEHLQLWYVRNAQEQMVPFSAFAEGN
WSYGSPRLERYNGSSAIDIVGEAAEGYTTGEAMATMTRLISQLPEGIGFEWTGLSYQEQESGSQAPALYAISLLVVFLCLAA
LYESWTIPFSVMLIVPLGVVGALAAATLRGLSNDIFFQVGLLTTIGLAAKNAILIVEFAKELFDKGMGIKQAVIEASRMRLRPIL
MTSLAFILGVFPLVISSGAGASGRHAIGTGVTGGMIAATVLAIFYVPSFFVLVMKYFSKHRHSLRAPTSAEKHHD

>AcrB_T_auensis
MRFTDLFIRRPVLAVTISLMMVLFGLEALRGMAIREYPKVTNTVITVTTNYYGASADVIQGFISQPVEQAIAQAENIDYMTS
RSQLGRSTITVNMRLNTDPNAALADVLAKVNSVRSQLPREADDPSITSSTGSNTSIVYIAFSSAELNSSQITDYLERVIKPQL
FTVPGVAKVNLYGGTQFAMRVWLDPLKMGAYNLSSTQVLSVLQSNNYQSAPGQATGYFVLYNAEADTQVNSTEQLEE
LVVASHDGAVIRLRDIAKVSLEKNHDQYRALANGKEAVIAAVDSAPSANPLEIAANVRKLLPDFERNLPNTMNMKLLYDS
TQAINESIHEVLKTIVEAAAIVLVVIALFLGSMRAVVIPIVTIPLSLIGVVMLMQMFGFSINLITLLAMVLAIGLVVDDAIVVVE
NVDRHIKAGEPPFRAAIIGTREIASPVITMTITLAAVYTPIALMGGVTGSIFKEFALTLAGAVFISGVIALTLSPMMCSVMLKP
HINPNRFERGVENTLNSITSGYSRMLDKVMTRRPVIIIFGIIVLAVMPLLFKLIPAELAPSEDRGAYLMMAKAPNTANLDYIQ
NNMAAAGETLMKDPAMSTNLALAGIPNNNQGLAIAILKPWSEREKAPVIVQRTNAALQDMPGVAISSFQFPELPGAGG
GLPLQLVITTPNEFRSLFEVASDALAKIKTNRQFVYTDLDLAFDSATMHIAINRDKAGAYGVSMLAIAETLSTLMGDGNVN
RVSLNGRSYEVIPQVERQFRLNPESIKHYYVTAANGQSIPLSNLVTVDLKSEIRALPHFNQLNSATIGIVPIMPMGDAVTWL
EENVVSELPLGYQHDYMGEARQYVQEGSALMYTFLLALCVIFLVLAAQFESWRDPLVIMMSVPLAISGALVALAWGLST
MNIYTQVGIITLVGLISKHGILICEVAREQQLLKGLSKMDAVKIAARVRLRPILMTTAAMVTGLLPLLGAMGAGALSRFSIGI
VIVAGLTIGTFFTLFVLPVIYTYVGKTHKPLQEFDESVAPLALHDDE

>AcrBF_C_japonicus
MARFFIDRPIFAWVIALVIMLAGVLSISALPIAQYPSIAPPAISISATYPGASARTLEDSVTQVIEQKMKGLDGLMYMSSTSES
SGTVTLMLTFTADTNPDIAQVQVQNKLALATPLLPQEVQRQGVTVAKSARNFLMVLGFVSADGSMSNIDIGDYVAANV
QDIISRVDGVGEVQLFGTQYAMRIWLDPARLQNYKLTPADISNAITAQNAQVSAGQLGGMPAIEGQQLNATVTAQSRL
QTVEQFRHIILRTNPDGSNVRLGDVAKIELGGDTYDVVARFNGKPAAGLGVKLAAGANALDTAEGVKAKIAELQPFFPEG
LQAVVPYDTTPFVKISIQKVVETLIEAVVLVFLVMYLFLQNFRATLIPTIAVPVVLLGTFGVLAAFGYSINTLTMFAMVLAIGLL
VDDAIVVVENVERVMTEEKLSPKEATRKSMREITGALIGIALVLSAVFVPMAFFGGSTGVIYRQFSITLVSAMALSVLVALSL
TPALCATLLKAGDHNHDAPKSLIGRFFAGFNRNFERVNKGTRSFVGRMIGQSKRYLVIYGLILLGLGFLFSRLPTAFLPDED
QGILFNQILLPAGATTERTLQVVEQVEKHFLEEQGDAVKSIFTVTGFSFAGRGQNAAIGFVNLRPWDERTESHLALNAVVG
KAMGSFSKIRDAMVFAFAPPAVIELGTANGFNLHLQDRAGLGHDQLLAARNQLLGMAAQNPVLSSVRPNGQEDKPEL
QLDIDLAKAGALGVSQADINSTLATAWGGSYVNDFIDKGRVKRVFVQGDASSRMNPEDLNQWYVRNSQGDMVPFSA
FASSHWGYGSPRLERYNGLASMEIQGAAAPGYSTGDAMLAMEELIAKLPPGIGFEWTGMSYQERMTGDQAPLLYALSLL
VVFLCLAALYESWFVPAAVMLVVPLGVLGAVIAATLVKLPNDIYFQVGLLTTMGLASKNAILIVEFAKGRMEQGMDLVAA
SIEAVRLRLRPIIMTSMAFVCGVLPLALATGAGSGAQNALGIGVVGGMLTSAGLAILFVPLFFVLIRRLFPVKKDIEATDEHD
TH

>MexIW_C_japonicus
MKFTDIFIQRPVLSIVVSLLILILGIQAASSLSVRQYPRSDIAVITIETVYVGANAELIRGFITAPIERAVASAEGIDYIASSSVMG
MSTIKVHLRLNYDPLKAMTEISAKVNQVRGNLPPEAEVPAIRVDSADSEFAAAYLSFSSEILEQNQITDFLTRLVQPRLTAV
AGVQKAEVLGGRTFAMRIWLKPERMAALNVTPVQVRAALAANNVQAAIGQTRGSYTQVNLRADTDLTSVDEFRQMIIR
NTADGIVRLSDIADVVLGAESYDVVVNYSGDTAVFMGVWVAPTANSLEVMKAVNEEMEIIKSELPTGLSGTVAYDSTEYI
DAAIHEVIKTLTETLLIIVLVIFLFLGFSRSVIIPVLAIPLSLIGALFIMQICGFSLNLLTLLSIVLCVGLVVDDAIVMVENIERHIQEG
LKPYRAAIVAARELAGPILAMTVTLASVYIPIGLQGGLTGTLFREFAITLAGAMTISAVVAITLSPMLGSRMLKPHKGLNAPLE
KPFEKFKTWYAEKLGVSLENRAGIYVFWVVVAFLCVPLYMMSPKELAPTEDQGVIFGIVDAQANSTVDQSDFYGRQVNQ
ALMSFPESDFTFQLTMPTGGFAGLVVKPWGDRERSVNEILPDVQAKLAEISGQKVLPVTPPALPGGGQFPFEFVLASTADI
KEIYEYALKIQEIMVKSGKFYFQVLDVKVDQPDYKLSIDRNKVADLGLDLQTVTNDVGTLMGGGYVNRFNMAGQSYKVIP

QVKRIGRLTPDDLANLYITGPDGGLIRLDQVASISHTTEPRTINRMQQLNAVKLSGVPGVPLGEALAFLEDEARKILPKSYSI
DYTGETRQLVREGNTFLPALTLAIIMIFLVLAAQYNSFRDPLVILAGSVPLAMFGALIFTFLKLPMPMPFFTNGFTSTLNIYSQ
VGLVTLMGLIARNGILVVEFANKLQEQGRAKLDAIREAAVLRLRPVMMTSIATIAGHTPLIFASGPGAEARNSIGIVLVLGM
TIGTFFTLFVLPSVYMLLAKDHQHDHEQLDELEEDISKA

>AcrBF_Shewanella
MARFFIDRPIFAWVIALIIMLAGILSIRSLPVSQYPSIAPPTVVISANYPGASAKIVEDSVTQVIEQRMTGIDHLRYITSTSDSFG
NASITLTFNAEADADIAQVQVQNKLQGAMTLLPQEVQSQGVNVNKSSSGFLLVLGFVSTDGSLDKSDIADYVGSNVQD
PMSRVSGVGEIQIFGAQYAMRIWLDPLKLTQYNLTSLDVVAAIRAQNAQVSAGQLGGTPSVAGQELNATVSAQSRLQT
PEEFRKIILKSDTSGANVFLGDVARVELGSESYSVVSLYNGKPATGLAIKLATGANALDTAEAVRAKIAEMKPFFPQGLEVVY
PYDTTPFVEKSIEGVVHTLLEAVVLVFLIMYLFLQNFRATLIPTIAVPVVLLGTFAILSATGFSINTLTMFAMVLAIGLLVDDAIV
VVENVERVMSEEGLSPLEATRKSMDQITGALVGIGLTLSAVFVPMAFMSGSTGVIYRQFSITIVSAMALSVLVALILTPALCA
TMLKPLKKGHGHIETGFFGWFNRGFDRLTNRYESSVAGIIKRGFRVMMIYVALVVAVGWIFMRMPTAFLPDEDQGILFTQ
AILPTNSTQESTLKVLEKVSDHFMAEEGVRSVFSVAGFSFAGQGQNMGIAFVGLKDWSERTAPGADVQSIAGRAMGVFS
QMKDAFVFAFVPPAVIELGTANGFDMFLQDKNGQGHEKLLAARNQLLGMAAQNPNLVGVRPNGQEDAPIYQLHIDH
AKLSALGIEIANVNSVLATAWGGSYVNDFIDRGRVKKVYVQGDAQYRMQPGDLDTWYVRNNKGDMVPFSAFAMGTW
EYGSPRLERFNGLPAMNIQGATVPGFSTGAAMDIMEDLVKQLPPGFGIEWNGLSYEERLSGNQAPALYALSILVVFLVLA
ALYESWSVPFAVVLVVPLGIIGALLAMNGRGLPNDVFFQVGLLTTVGLATKNAILIVEFAKEYYEKGAGLVEATLHAVRVRL
RPILMTSLAFGLGVVPLAISTGVGSGSQNAIGTGVLGGMMSSTFLGIFFVPLFFVIVERIFSKRERKAKDEKPTSAE

>MexBYD_Shewanella
MPQFFINRPVFAWVIALFIVLIGVIAIPKLPISRYPSVAPVSVSIYAAYPGATPQTLNDSVVSLVERELSGVKNLLYFESSVDSS
GSAQITATFKPGTDAEMAQVDVQNRIKAVEPRLPQVVRQIGLQVESAASSFLMMIGMTSPNGQFDEIALNDYLARNIVE
ELRRIDGVGRVQLFGAEQAMRVWVDPNKLTAYSLTMNDLAIAIEQQNAQIAPGRLGDEPVLQGQRLTVPLTVQGQLST
PEEFAAIVLRAGADGAKLVLADVAWVELGAQSYGFSNRENGVAATSAAIQLSPGANAVRTASAVRERLAELAPMMPAG
MNYSVPFDTAPFVKVSIEKVIYTLFEAMALVFMVMFLFLQNLRYTLIPAIVAPIALLGTFAVMLLAGFSINSLTMFGMVLAIGI
IVDDAIVVVENVERLMATQGLSPREATSQAMKEITGAVIGITLVLTAVFIPMAFGSGSVGVIYQQFTLSMAVSILFSAFLALT
LTPALCATLLRPVSADHHQKGGFFGTFNRGFEHLTAGYSERVARLVGRSGRMMAAFAVLCMVLILAAGQLPSSFLPEEDQ
GYFMTSIQLPTGATSERTLDVVKAYEAHVASRPTLDANMVVLGFSFAGSGPNAAMAFTMLKDWDQRNGATAAEEAAL
AQQAMADNVEGTVMSLMPPAIDELGTSSGFTLFLQDRANRGEAALIAAQTQLLALAAQSEVVSDVYPDGLPPGESISLNI
NRSKAEAMGLSFAAVSSTLSAAMGSLYVNDFPNNGRMQQVILQADATARMQLDDVLSLRVRNSSGGMVPLREIVTAE
WKESPQQMMRFQGFPAMRISGGAAAGVSSGAAMTEMERLVAQLPQGFAVAWTGASLQERQSAAQAPLLMLLSALV
VFLVLAALYESWSIPLSVMLVVPLGLLGAVAAVILRDMPNDVFFKVGMITIIGLSAKNAILIVEFARQLHAEGHTLIDAAVTA
ARLRLRPILMTSLAFTLGVVPLMLASGASAETQHAIGTGVFGGMLSGTLLAIFFVPAFFVFVIGTQERLSAWLGRRNHSKM
AKG

>MexQ_Shewanella
MNFPRFFINRPIFAIVLSVLMLIAGAISYFQLPLSEYPQVTPPTVQVTASYPGANPQVIADTVASPLEQAINGVEGMMYMQ
SQMSTDGKMVLTISFEQHINADIAQIQVQNRVSRALPRLPPEVQRIGVVTEKTAPDILMVVNMLSPGDRYDPLYVSNYA
MLNVRDELARLPGIANVLLGGEGEYAMRVWLDPEKVASRGMTASDVVAAIREQNVQVAAGSIGQQPNASSAFQVTVN
ALGRLTTEEQFGDIVIKAGTHGQVTRLRDVARVELGAENYTMRAQLDGGNTVGIQILMTPGSNALDVSSAVRATMDRLQ
ASFPEGIEYKIAYDPTVFVRASLQSVAVTLLEAILLVVIVVVLFLQSWRASIIPLIAVPVSLVGTFAVMHMFGFSLNTLSLFGLV
LSIGIVVDDAIVVVENVERHMALGESPKDAALKAMDEVTGPILAITSVLAAVFIPSAFLSGLQGEFYRQFALTIAFSTILSAINS
LTLSPALAAILLKPHHGAAQPDRVTRLIDGLFGGFFRRFNRFFDSASNAYVGGVRRAVRGSAVVLLLYVGFLGLTWLGFHK
VPAGFVPAQDKYYLVGIAQLPTGASLDRTDAVVKEMSKIALAEPGVESVMAFPGFSFNGPVNQPNTALVFAMLKPFNER
KDPSLSAFAIQGRLMGKFSQIPDGFVGIFPPPPVPGLGSMGGFKLQIEDRAGLGPDALAQAQGQIMGKAMQAPELANM
LASFQANAPQLQVDIDRVKAKSQGVSLTEVFDTLQINLGSLYVNDFNRFGRTYRVMTQADAKFRMQAEDIGMLKVRNA

SGDMIPLSAIATISRSSGPDRVMHYNGFPSADISGGPAPGYSSGQATAAIEKIVSESLPDGMTYEWTDLTFQEKRVGNTSIY
IFALAVLLAFLFLAAQYNSWSLPFAVLLIAPMALLSAIAGVWLTGGDNNIFTQIGFVVLVGLAAKNAILIVEFARAKESEGVD
PLAAVLEAARLRLRPILMTSLAFIAGVIPLVLASGAGAEMRHAMGIAVFAGMLGVTAFGLVLTPVFYVVVRKLALRREASQ
ARVGMSDKHNGQEEF

>MexF_Shewanella
MLSQFFIKRPIFAAVLSLLFLITGAIAVWQLPITEYPEVVPPTVVVTANYPGANPKVIAETVASPLEQEINGVEDMLYMSSQA
TSDGRMTLTITFAIGTDVDRAQTQVQSRVDRAMPRLPQEVQRLGIVTEKSSPDLTMVVHLLSPDNRYDMLYLSNYAALN
VKDELARIKGVGAVRLFGAGEYSLRIWLDPNKVSALGLSPADIIAAVREQNQQAAAGSLGAQPSGSADFQLLINVKGRLTE
LSEFEDIIIKVGQNAEVIRLKDVARVELGATSYALRSLLDNKDAVAIPVFQASGSNAIQISDDVRAEMARLAKSFPEGLQYEI
VYDPTVFVRGSIEAVVKTLLEAVLLVVLVVVLFLQTWRASIIPLVAVPVSLVGTFAFMHLLGFSLNALSLFGLVLAIGIVVDDA
IVVVENVERNIAAGLSPMAATQKAMKEVTGPIVATTLVLAAVFIPTAFMSGLTGQFYKQFALTITISTFISAINSLTLSPALSAL
LLKSHDAPKDGLTRLMDKLFGAWLFVPFNRLFNRASDGYGYLVRKVIRFGGIIGLVYLGMVALTGVQFANTPTGYVPGQD
KQYLVAFAQLPDAASLERTDSVIKKMSEIALNHPGVAHSIAFPGLSINGFTNSPNSGVVFVALDDFELRKSPELSANAIAGQ
LNQQFAGIQDAFIAIFPPPPVQGLGTIGGFRLQIQDRANLGYEALYQVTQQVMYKAWADPQLAGIFSSYQVNVPQLELDI
DRTKAKQQAVSLDQIFQTLQTYMGSTYVNDFNRFGRTYQVNMQADEAFRQSPQQISQLKVPNVNGDMIPLGSFINVS
QSAGPDRVMHYNGFTTAEINGGPAPGVSTGQAQAAIEKILAETLPIGMTYEWTELTYQQILAGNTGLLVFPLVILLVFMVL
AAQYESLSLPLAIILIIPMTLLSALSGVLIYGGDNNIFTQIGLIVLVGLATKNAILIVEFAKEKQDHGMAPMEAILEAARLRLRPIL
MTSIAFIMGVVPMVFSTGAGAEMRQAMGVAVFAGMIGVTVFGLILTPLFYYALAKRGSKNVVQLEGISTNQHHEKT

>AcrB_Shewanella
MRLPEVCIRHPIFASVLSIMAVLLGLIAFHKLDIQYFPEHTTHSASVNASIAGASADFMSSNVADKLVAAASGLDKVDTMST
DCSEGRCSLTIKFKDDTTDIEYTNLMNKLRSSVEGINDFPQSMIDKPTVTDDTSATDSASNIITFVNTGGMEKQAMYDYIS
QQLVPQLKQVQGVGAVWGPYGGSQKAVRVWLNPEQMKALNIKAADVVGTLGSYNASFTSGAIKGKSRDFSINPLNQV
ETLEDVKDLVIKVSDGKIIRVSDVAEVVMGEESLSPSLLSIGGHSAMSLQILPLSNANPVTVASNIKAEIARMQKHLPQGLE
MNLAYNQADFIEAAIDEGFAALIEAVILVSLIVVLFLGSLRAASIPIITIPVCVIGVFAVMSALGFSINVLTILAIILAIGLVVDDAI
VVVENCYRHIENGETPFNAAINGCQEIIFPIIAMTLTLAAVYLPIGLMSGLTADLFRQFSFTLAAAVIISGMVALTLSPMMSA
YLINTTEQQPKWFSRVEGALQQLNHLYINELSKWFTRKRQMLGMALVLMGLAGIAYWQLPKILLPVEDSGFIDVAANGP
TGVGRQYHLDHNSELNGVIDGHPAVGANLSYIEGEPVNHVLLKPWGERREGIDEVIADLIAKSKESVSAYNMSFSIRSADN
LNIATNVRLELTTLDRNKDKLSETAAKVQKLMEDYPGLTNVGNSVLRDQLRYDLSIDRNAIILSGVSYGDVTNALSTFLGSV
KAADLHATDGFTYPIQVQVNLDKLSDFRVMDKLYVTSESGQALPLSQFVSINQTTAESNIKTFMGLDSAELTADIMPGYST
DEIKAYLDEQLPTLLTDAQGFKYNGVIKELMDSQAGTQSLFLLALVFIYLILAAQFESFVDPLIILLTVPLCIVGALLTLTLFGQS
LNIYSQIGLLTLVGLVTKHGILLVEFANKQQLQGFSAIESALSSAKSRLRPILMTSLTMILSAIPLALASGPGSLGLANIGLVLV
GGLLAGTFFSLFVVPVAYVAMAELKARDVYPSYLKMQDSAADFND

>AcrBF_P_mirabilis
MPKFFIDRPIFAWVIAIITMLAGLLALIKLPVAQYPTIAPPAISISAVYPGADATTVQNTVTQVIEQNMNGIDNMVYMSSTS
DSAGMMNITLTFEAGTDPDIAQVQVQNKLQLAMPLLPQEVQQQGISVDKSSSSFLMVAGFISGDGSMSQDDIADYVG
ATIKDPLSRVTGVGETQLFGTQYAMRIWLDPDKLVKYNMTTLDVINAIKSQNNQVAAGQLGGTPPVPGQRLNVSIIAQT
RLNTPEQFADILMKVNQDGSQVKLKDLGIVEMGAENYSTIARFNGLPAAGIGIKLATGANALDTATAVRAALADMEPFFP
SGLEVVYPYDTTPFVKISIFEVVKTLVEAIMLVFVVMYLFLQNFRATLIPTIAVPVVLLGTFAILSAFGYSINTLTMFAMVLAIGL
LVDDAIVVVENVERVMQEEGLSPKEATRKSMGQIQGALVGIALVLSAVFIPMAFFGGSTGAIYRQFSVTIVSAMVLSVFVA
LILTPALCATLLKPIPKGSHGVQTGFFGWFNRVFEKSSHHYTDSVSRTLRGTGRYLLIYVLLVAAMAIMFVRLPASFLPEEDQ
GVLLTMVQLPAGSTQEQTQDVLAQVNDYFNTKEKDVVKSVFTVSGFGFGGQGQNMGLAFVVLNDWDERKSDQDKV
PAIVGRANQALSQIKEAFVYSFNIPAIVELGSAGGFEFELIDNANLGHEKLMEARNQLLGMAAQEPQMLTGVRPNGQED
TSQYRLYIDLEKAQAQGVAVSDIYSTLGTMFGGSYVNDFIDRGRVKKVYVQAESQFRMLPQDIANLYVRNNAGQMVPF
SSFIDTSKDPWLYGSPRLERYNGLPAVQIQGSATPGQSSGDAMLMMEQLASKLPSGIGYEWTGMSYQERLSGNQAPAL

YTISLIVVFLCLAALYESWSVPFSVMLVVPLGVIGALAFTTLRGLENDVYFKVGLLTTIGLSAKNAILIVEFAKDLMEKEGKGLV
EATLDSVRMRLRPILMTSLAFMLGVIPLVLSNGAGSGAQNSVGTGVFGGMIAATSLAIYFVPIFFVVIRRRFAKKNEDLEHP
NYPHQTK

>AcrBF2_P_mirabilis
MSRFFINRPILSWVMAIVIMIAGGLSLIKLPVSQYPEIAAPAIMITAVYPGASAETVQDTVVQVIEQELSGLDGFRYLSSNSN
TDGSFEVIATFEQGTDSDIALMQVQNKLQLATPKLPLEVQRQGMRVVKFQINFMQVVALVDESGKLDAFDLGNYLASH
MKDPISRIDGMGDILLLGSPRAMRIWLDPDKLTNYHLMPQDIAAAIQDENVQVSAGQLAGLPALKGVELNATITSASRM
VSVDEFNHILLKVNLDGSQVLLQDVAKVEVGADNFSISSLYNGKPSAGMAMRLAPGANLLDVSKKVKEVVEAQSQFFPQ
GVKALFPYDTTPSVKSSISSVVRTIVEASILVFLVMFLFLQNIRATLVPTLAIPVVLLGTFGVLAALGLTINVLTMYAMVLAIGLL
VDDAIVVVENVERIMHEQAISVKEATILSMTQIQGALVGIGLVLSAVFVPMAFFGGSAGIIYRQFAATIVVAMALSVLIALTF
TPALCASILKKPQPKVKKRRGFFWLFNRFFDRGTNYYEKGVRNILYRRGRYMFIFLIITVGTGWLFTQLPKAFLPNEDQGVV
MIEVRMPANSTAERMEQTMAEFRDYLLTEEKHLVKTVFTVNGFNFAGRGQNAGMAFVNLADWSERKGKGEDVFSLVA
RAQQHFNSIRDGMVIAFPPPAIMEMGNAMGFDFYLQDNAGLGHTELVKARDKFLELAAQSPVLTNVRPNGKNDEPQL
RVVIDKERARALQLRLSDINDTLSAAWGSMYVNDFIDEGRVKRVYLQGEASARMLPTDLDKWHVRNQRGEMIPFNAFA
TTQWYMGPPKLERFNGLSAIEILGEPAPGYSTGDAMLAIADIMKQLPSEISLSYSGLSYEEIQTQDQAPALYVMTILFVFLSL
AALYESWSVPFSVALVVPFGILGAVIATLLRGLSSDVYFQIGLMTTIGLSAKNAILIVEFAKHLYEKDGKPLIDAAAEAARMRL
RPIIMTSLAFTFGVLPMALAAGAGQGSQHSVATGVIGGMLASTFLAIFFVPLFYVFVVKLFSRKKAHVSIEEIDK

>MexIW_P_mirabilis
MKFTDIFVRRPVLALVVSTLIFLLGAFAFSKLPIRQYPMLQNSTITIATDYPGASSELMQGFVTQPITQAVSSVEGVDYISSSS
VQGKSFVTVRMELNRDPTQALTQVMAKVNQVRYKLPEQAYDPVIELSSGESTAVAYVGFSSEQISIPELTDYLSRVVEPMF
SSINGVAKVQVFGGQQLAMRVWLDTDKLAGRNLTASDVADAIRRNNYQAAPGKVEGEFVIANVYVNTDLTNVEEFKD
MVIHNDGHNLIRLRDIGTVELGAAATETSGIMNGKKAVFLGLFPTPTGNPLIIVDGIRENLTDIEKTLPPGVNVELAFETSRFI
KASIDQVVQTLIEAILIVIAVIYLCLGSLRSVIIPVLAIPLSMLGAAGLMLAFGFSINLLTLLAMVLAIGLVVDDAIVVVENVHRH
IEEGLSPVQAALVGAREVAGPVIAMTITLAAVYAPIGLMSGLTGALFKEFAITLAGSVIVSGIVALTLSPVMSSLMLKPKENEG
RMAKIAEYTFDKLAYYYSYLLNFSLTHRWLTVVFAFAVFVSLPFLYSQTKQELAPLEDQASVLTAVKAPQHANLAYAEHFN
QKLDDIYMSLPETDSTWIINGTDGPSASFGGINFDGWKGRDRNANQIQADLQNRVADVEGSSIFAFQLASLPGSVGGLP
VQMVLRSPLGYPVLFDTMEEIKQQARESGLFVVVDSDLDYNNPVVQITIDRAKANTLGIRMQDIGESLSLLVGEHYINRFG
MDGRSYDVIPQSIRQQRLTPAALAGHYIRTQDNLLIPLSTVVNITTQVEPNKLTQFNQQNAAIFQAIPAPGVTMGQAVAF
LENVANSLPAGFSHDWQSDARQFTQEGNTLVFAFIAALIIIYLVLAAQYESLVDPLIILITVPLSICGALVPLALGMVTLNIYT
QIGLVTLIGLISKHGILMVEFANELQVHKNLNRREAIIEAAKIRLRPVLMTTAAMVIGLIPLLFASGAGANSRYGLGLIIVSGML
VGTLFTLFVLPTMYSFLARNHQVSAQTERQKQLQQISEQ

>MtrD_N_gonorrhoeae
MAKFFIDRPIFAWVISIFIIAAGIFGIKSLPVSQYPSVAAPTITLHAIYPGASAQVMEGSVLSVIERNMNGVEGLDYMSTSADS
SGSGSVSLTFTPDTDENLAQVEVQNKLSEVLRTLPATVQQYGVTVSKARSNFLMIVMLSSDVQSTEEMNDYAQRNVVPE
LQRIEGVGQVRLFGAQRAMRIWVDPKKLQNYNLSFADVGSALSAQNIQISAGSIGSLPAVRGQTVTATVTAQGQLGTAE
EFGNVILRANTDGSNIYLKDVAKVGLGMEDYSSSTRLNGVNTTGMAVMLSNSGNAMATAKAVKERLAVLEKYFPQGMS
WKTPYDTSKFVEISIEKVIHTLIEAMVLVFVVMYLFLQNIRYTLIPTIVVPISLLGGFAFISYMGMSINVLTMFAMILVIGIVVDD
AIVVVENVERIMAGEGLPPKEATKKAMGQISGAVIGITAVLISVFVPLAMFSGAAGNIYKQFALTMASSIAFSAFLALTLTPA
LCATMLKTIPKGHHEEKKGFFGWFNKKFDSWTHGYEGRVAKVLRKTFRMMVVYIGLAVVGVFLFMRLPTSFLPTEDQGFV
MVSVQLPAGATKERTDATLAQVTQLAKSIPEIENIITVSGFSFSGSGQNMAMGFAIFKDWNERTASGSDAVAVAGKLTG
MMMGTLKDGFGIAVVPPPILELGNGSGLSINLQDRNNTGHTGIAGEGNELIQKMRASGLFDPSTVRAGGLEDSPQLKIDI
NRAAAAAQGISFADIRTALASALSSSYVSDFPNQGRLQRVMVQADEDARMQPADILNLTVPNKSGVAVPLSTIATVSWE
NGTEQSVRFNGYPSMKLSASPATGVSTGQAMEAVQKMVDELGGGYSFEWGGQSSEEAKGGSQTLILYGLAVAAVFLVL

AALYESWSIPLAVILVIPLGLIGAAAGVTGRNLFEGLLGSVPSFANDIYFQVGFVTVMGLSAKNAILIIEFAKDLQAQGKSAV
EAALEAARLRFRPIIMTSFAFILGVVPLYIAAGASSASQRAIGTTVFWGMLVGTLLSVFLVPLFYVVVRKFFKETAHEHEMAV
RHASKAGITGSDDKQY

>MexB_X_campestris
MARFFIDRPIFAWVLAIIVMLAGILSIATLPIAQYPSIAPPAVAITANYPGASAQTLEDTVTQVIEQKMKGLDNLSYMASTSE
SSGAVTITLTFENGTDPDTAQVQVQNKLSLATALLPQEVQQQGVTVTKSATNFLNVLAFTSEDGSMSDSDLSDYVAANV
QETISRVQGVGDTTLFGSQYSMRIWLDPDKLNNFNLTPVDVRAAIQAQNAQVSAGQLGALPAVANQQLNATITAQTRL
KTAEEFENILLRTRTDGSQVRLRDVARIELGSESYNTVGRYNGKPAAGLAIKLATGANALDTVRAIDKSLEEQEKFFPPGMK
VQKPYDTTPFVRISIEQVVHTLVEAVVLVFLVMYLFLQNFRATLIPTIAVPVVLLGTFGVLAVFGFTINTLTMFAMVLAIGLLV
DDAIVVVENVERVMAEEQLSPKEATRKSMDQITGALVGVALVLAAVFVPMAFFGGSTGVIYRQFSITIVSAMTLSVLVAM
VLTPALCATLLKPGHGMATTGFFGWFNRVFDRSNGRYQGVVRHMLGKGWRYMIAYVVIVALVVVGFMKTPVGFLPDE
DQGTLFVLVQLPPGATDARTGAVLKQVEHHFLVDQKDSVAGIFAVSGFSFAGTGQNVGFAFVKLRPWDERTGKGQSVT
DVAAKAGAFFATLRDAQVFAVAPPAVSELGNATGFDLMLQDRANLGHAALMQARNQLLAELSQDKRLVAVRPNGQE
DTPEFKLEIDPHKAEAMGVSIADINNTFSSAWGSTYVNDFIDKGRVKKVMLQADAPYRMNPQDIDRWFVRNSAGTMVP
FNAFATARWTQGSPRLERYNSLPSVEILGMALPGAASSGQAMQIVEAAAAKLPAGIGFEWTGLSRQEKASTGQTGLLYG
LSILIVFLCLAALYESWSIPFSVILVVPLGVFGTLVAAVLTWKMNDVYFQVGLLTTIGLASKNAILIVEFAKELHEGGKSLVAAA
LEAARMRLRPILMTSLAFILGVVPLVLTSGAGAGAQHALGTAVIGGMISGTVLAIFFVPLFFVLVRGLFERRAQPDASVPQH
AGH

>MexDY_X_campestris
MPKFFIEHPVFAWVVAILISLAGVISILNLGIESYPTIAPPQVTVTANFPGASADTAEKAVTQVIEQQLTGIDHLLYFNSSSAS
NGRVTITLTFETGTDADIAQVQVQNKVSLATPRLPSEVTQQGVVVAKANAGFLMVAALRSDNPSIDRDALNDIVGSRVL
EQISRVPGVGSTNQFGAEYAMNIWLNPEKLQGYNLSATQVLTAVRNQNVQFAAGSVGADPTPQGISFTATVSAEGRFS
SPQEFENIILRTDNNGATVRLKDVARVTVGPSNYGFDTQYNGKPTGAFGIQLLPGANALNVSEAVGAKLDELQPTFPQG
VTWFAPYESTTFVRISIEEVVHTLVEAIVLVFLVMLLFLQNFRATVIPTLVIPVALLGTFFGMYVIGFTINQLTLFAMVLAIGIVV
DDAIVVIENVERIMSEEHLEPKAATQKAMTQITGAVVAITVVLAAVFIPSAMQPGASGAIYKQFALTIAMAMAFSAFLALSF
TPALCAAFLKSTHSDKKNWIYRTFDKYYDKLAHRYVGVVGNTLKRSPAWMVVFVVLVVLCGFLFTRMPGSFLPEEDQGF
AVAIVQLPPGATKIRTNEAFAQMRAVLEKQPAVEGMLQIAGFSFLGSGENVGMGFIRLKPWEERDVTAEQLIQQLNGAF
YGIKGAQIFVVNLPTVQGLGQFGGFDMWLQDRSGAGQEALTQARNIVLGKAAQKQDTVVGVRPNGLENSPQLQLHV
DRVQAQSMGLEVSDIYSSIQLMLAPVYVNDYFSEGRIKRVNIRADDQFRTGPESLRNFFTPSSTATGADGQPGMIPLSNV
VKAEWTYASPALNRYNGYSAVNIVGNPAPGGSSGQAMQAMEEIVNNDLPPGFGFDWSGMSYQEIIAGNAATLLLALSV
VVVFLCLAALYESWSIPVAVLLVVPIGVLGAITFSMLRGLPNDLYFKIGMITVIGLAAKNAILIVEFAVEQRAAGKTLREATLEA
AHLRFRPILMTSFAFILGVLPLAISTGAGANSRHSIGTGVIGGMVFATVLGVIFIPLFFVVVRRMLGDKLDEPPKEYLAVQND
SGTHRPDR

>MexFQ_X_campestris
MDFSRFFIDRPIFAAVLSIIIFAAGLIAMPLLPISEYPEVVPPSVQVRAVYPGANPKVIAETVATPLEEAINGVENMMYMKSVA
GSDGVLVVTVTFKPGTDPDQAQVQVQNRVSQAQARLPEDVRRQGVTTQKQSPTLTMVVHLTSPKGKYDSLYLSNYAT
LKVKDELSRLPGVGQIQIFGAGDYAMRIWLDPDKVAARGLTASDVVAAIREQNVQVSAGQLGAEPMPNKSEFLLSINAQ
GRLTTEEEFGNIVIRSGTSGEIVRLSDVARLELGAGKYTLRSQLDSKSAVGMGVFQSPGANAIELSDAVRAKMAELEKQFPQ
DMAWSAAYDPTIFVRDSIKAVVSTLLEAVLLVVLVVILFLQTWRASIIPLLAVPVSVVGTFAALYVLGFSINTLTLFGLVLAIGI
VVDDAIVVVENVERNIEEGLSPLAAAHQAMREVSGPIIAIALVLCAVFVPMAFLSGVTGQFYKQFAVTIAISTVISAINSLTLS
PALAAMLLKAHDAPKDGPTRLIDRLFGWIFRPFNRFFNTSSHKYQGAVSRALGKRGMVFMVYLLLLVGTGVMFKLVPGG
FIPTQDKLYLIAGAKLPEGASLERTSEVISQISDIALQTEGVAHAVAFPGLNPLQFTNTPNTGTLFLTLKPFSERSRTAAQINA
EINARISQIEKGFAFAFMPPPILGLGQGSGYSLYIQDRAGLGYGQLQTAVTAMSGAISQTPGMQFPIGTYQANVPQLDAK
VDRDKAKAQGVPLTNVFDTLQTYLGSAYINDFNRFGRTYQVIAQADGQFRDSVEDIANLRTRNDRGQMVPIGSMVTLG

QTYGPDPVIRYNGYPAADLIGEADTRVLSSAQAMQTLAGMAPKVLPNGMNIEWTDLSYQQSIQGNSALIVFPMAVLLA
FLVLAALYESWTLPLAVILIVPMTLLSALFGVWLTGGDNNVFVQVGLVVLMGLACKNAILIVEFARELEMHGKGIVEAALEA
CRLRLRPIVMTSIAFIAGTVPLVFGHGAGAEVRSVTGITVFAGMLGVTLFGLFLTPVFYVALRKWVTRGEPAAPAAVAHDP
VKA

>MexB_T_turnerae
MARFFIDRPVFAWVIAIVIMLAGVLAIVQLPVAQYPEVAPPSVSIRASYPGASAKVVEDSVVQIIEQNLTGVDNMQYMSST
SDSSGTAEVTLTFVAGTDPDIAQVQVQNKLQAAMPLLPNEVQQQGIRVTKSTAGFLMVLGFVSEDGSLDKYDISDFIAA
NVREPLSRVNGVGTIQLFGSQYAMRIWLDAHKMAAYDFTTVDVVNAIRAQNAQIAAGELGGAPALPGQDINASIVVQT
RLNNAQQFSNILLRVNPDGSQITLGDVARVEMGAENYQFETEYNNQAATGLAVTLASGANALETADAVMARIEDLKPFF
PHGLTVVKAFDTTPFVKLSIEGVVHTLIEAIVLVFLVMYLFLQNFRATLIPTIAVPVVLMGTFATLLAFGFSVNTLTMFAMVLA
IGLLVDDAIVVVENVERVMHEDNLGPREATRKSMSQITGALIGIALVLSAVFIPMAFFGGSTGIIYRQFSITIVSAMLLSVVVA
LTLTPALCASMLRKSDVDHYARRGFFGWFNRKFDSSSDGYQRGVGYMLARKGRFMVVYLVIVGALALLFGRLPGSFLPDE
DQGIMFTQMSLPVGSTKERSMAVMDKVESYFLEQEKANVESVFSVIGFSFSGRGQNNGIAFVKMRDWSERTGAGQDV
QSIAGRAMGYFMQMREAMVFPFVPPAITQLGTSSGFNLQLKDLSAQGHDALMAARNQLLGMAAQDSRLARVRPNGQ
EDMPQFKLDVDTARAAALGVSVDQINAVFSTAWAASYVNDFIDNGRVKRVYVQGDTPYRMNPDDVGDWYVRNDKG
EMVSFSAFASGRWIYGSPRLERYNGISSVNIQGQGAPGVSSGEAMAIMEELAAKLPPGFGYEWTGMSLQERQSGDQAP
MLYALSILVVFLCLAALYESWSVPIAVMMVVPLGVIGALLFAMLRGMSNDVYFQVGLLTTIGLSAKNAILIVEFARAIYEEEK
KSLIEATMEAVRMRLRPIIMTSLAFGLGVLPLVIASGAGSGSQNAIGTGVFGGVLSATALAIFFVPVFFVVVFQLSHRFGNKT
AESSDKPAETQST

>MexDYB_T_turnerae
MIARIFVQRPVLAGVFAIALMLLGGIALRFLPVSQYPNISPPMVLVEAVYPGASAKVVENSVTQILEQSLKGLDGLLYFDSVS
TSDGVVELTLVFDLNTDVEVAQVRVQNKVNQISYRLPAQVQQRGITVNSLQNSFLMVAVFHDASGRLSDSDIANWISSS
MEDPLSRLPGVGAIRNFGAPYAMRIWLDPHQLAAHQLMPSDVEEAIYAQNTEVPVGELGARPHREGQQLNVTVTAMS
RLQSVDDFASIVLKTRAQGDVVRLGDVARIEIGRESYASTSRLNGKPASGLAMMMAPGANAMATASAVKNRIAQLRHTF
PAGVDVFYPEDASRFVKRSIRDVLRTLAEALVLVVLVMYLFLQNWRVTLIPTLTIPIVLLGTCCVIALLGYSLNTLNLFAMVLA
IGLLVDDAIVVVEHVERTMMQEKLDARSATLKSMQHMTRVLIGIALVLAAVFLPMAAFPGSVGVIYRQFSVTLVTAMALS
AVVALTLTPALCAALLKPADGHKRNPVTRAFNRLFSVMKLRYHHRVTALLKRPLRFSAIYLVLLGSTWWAYQSLPTAFIPED
DQGTVMVRYALPEGASYTRTRELVRTVENYFMQHEAKNIAGIYTVSGFSFSGSGQNAGIGFVPLKDWSQRQGQENSAQ
AIAARANRALADLVEGKVFAMVLPPIDGLGDTNGFEFWLQDVAGRGREALVADAWQLVNSLTNEPDIMFADADGGES
SPNLNIHIDQQKASAMGLNLHTINQTLSTAWGGRYVNDFVYDGQLRKVFIQADAPFRSAPENINDWQVRNNLGEMVP
FSAFTAQQWESGPGQISRFNGLPAVRITGAAAPGASSGRIMERIETLAATLPGTNYEWSGLSYQDKISSGSATLLYTVSILFV
FLCLAALYESWSIPLAILLVIPLGVLGAVVLVMLSGQSNDIFFQIGVLTSIGLSARNGILIVEFAEHAIQRGGSAFHATRKAASL
RLRPILMTSLSFGAGVVPLIVATGPAANAQHAIGLAVLGGVITATLLTIFYVPLAYLAICRIRGWLSSSTQPINPVASEGVPL

>MexFQ_T_turnerae
MNISRFFVDRPIFAGVISLLIFISGLLAVFQLPISEYPEVAPPSVVVNASFPGANPKVIAETVARPLEEQMSGVENMIYMFSQA
TTDGQMMLTVTFKIGTDPDLAQQMVQNRVSQALPRLPEVTRQVGVTVTKSSPNLTMVVHLTSPDQRYDDLFLRNYAVL
SVKDELAKLTGVGMVRLFGSGDYAMRIWLNPDKIAEYNLTSTDVVNAIREQNVQVAAGIIGGAPVSSAIEVQMPINAKGR
LETPEEFGSIIIKAGQHGEITRLRDVARIELGASDFGLNALLDNKPAVAIPIFQAPGANAIQISDQVRATMKRLSEDFPAGMS
YELVYDPTVFVRGSIEAVVHTLFEALLLVVIVVVVFLQTWRASIIPLLAVPVSIVGTFALMHVFGFSINTLSLFGLVLSIGIVVDD
AIVVVENVERNIEEGLHPIQATYKAMQEVSGPIIAISLTLVAVFVPIAFVSGLTGQFYKQFALTIAISTVISAVNSLTLSPALSAL
LLKDRDAPKDLLTRGMEKVFGPFFRGFNRVFGASSNGYGRSVQHLLGRKTIMMAIYAGFLAITCWEFISIPKGFVPPQDKQ
YLIAFAILPDGATLERTRQVIEDMGAIGLEEEGVANAVQFPGLSINGFVNSSSAGIVFFPLTDFDERTDSSQSAAAIAQRLQ
QKFGAIDEAFIAIFPPPPVRGLGTTGGFKLQIEDRAGLGYEALADVVAQASQKAMQRPELSRVYSNYKINVPQLYADLDRT

KAKQLGLDLREIFETMQIYLGSLYINDFNQFGRTYRVIAQADAQYRESIDDALNLKVRNRDGDMVPLGSVVHISDSYGPES
ATHYNGYLAADLNGDAAPGYSSGQAQDAISEILDEVLPPGISYEWTDLTYQQVLSGNTAIFIFPLCLILVFLVLAAQYESVV
LPLAVILVVPLSILAALAGVFISGGDNNVFTQVSLFVLAGLACKNAILIVEFARELEMEGMPTLKAAITASRLRLRPILMTSFAF
IMGVVPMVFSTGAGAEMRVVMGIAVFSGMLGVTAFGLFFTPIFYVLLRTLEKKFQKSPAEKPTTTQAEAI

>MexIW_T_turnerae
MKTVFTDIFVRRPVLALVVNLVIIIAGIQAISSLTVRQYPRNDNAVITVNTAYIGANAQLVRGFITTPLERAISGADGIDYINSS
STQGMSTINVQLELNYDPIKALSEITSKVNQVRGDLPPEAEVPIINVQSADSQFASAYLSFSSEILEPNQITDYLTRVVQPRL
AALEGVQRADVLGGRTFAMRIWLKPDRMAAYGITPVQIRQALTSNNYLAALGNTKGSFIQVNLSANTDLNTVEEFRDLV
LRSENGALVRLSDVADVELGSESYNEEVRYSGMTTTFMGIWPMPNANTLDVMKLVRKELDSLRDGMPKGMDAVIGYDA
TEYIESSIHEVIKTLSETLLIVIAIIFLFLGSVRSVVIPVMAIPVSLIGAIFLMQLFGFSLNLLTLLAIVLSVGLVVDDAIVIVENVERHI
SEGQSPMKAAMLGARELVGPVIAMTITLIAVYLPIGLQGGLTGSLFREFAFTLAGAVTISGIVALTLSPMMSSKLLKEGIEDEG
FAKRSASAFNAIRARYLRILDVSLRARGAIYVLWFLLSAGALAMFMSSPKELAPTEDQGIVFIMSEGASSSTIDQSKIYANAL
NEKLLSVPEGEFTFQITFPDGGMGGLLLKPWAERDRSVFQLLPEITADISQIGGINNFAIAPPALPGGGSFPVEFVIAATGETE
DILKYAEQLQQAAATSGMFMFPPQIDTKVDVPEAELVIDRDKVADLGLSLQQVGADVGVMLGGGYVNRFNIGGRSYRVI
PQVKRSERLNPAQLQDIFITGPEGKLIPLSTVARIEEKTVPRSLNRFQQLNAVKLSGVSTQSLDAALNFLETEAAKILPSDYM
VDYTGESRQLRREGNKFVPAFTMALLLIFLVLAAQFNSFRDPFVILAGSVPLAMFGASIFTFLKFPVPGMGFWTDGFTTTLN
IYSQVGLVTLIGLVAKNGILLVEFANKMQELGLTKMAAVREAASTRLRPILMTTVATVAGHFPLTLVTGAGAEARNSIGLVL
VGGMAIGTIFTLFVIPSIYVLLAKNLHGELEEVPEEPEEPMDPITV

>AcrBF_P_haloplanktis
MSRYFIDKPIFAWVLAIIVMLAGILAVKSLPIAQYPSIAPPAISITANYPGASAQTLEDSVTQVIEQKMKGLDGLLYMSSTSES
NGSATLTLTFNADTDPDIAQVQVQNKLATATPLLPEEVQRQGVVVAKAARNFLLVLAFVSKDGSMTNIDIGDYVASNVQ
DIVSRVDGVGEAQLFGSQYAMRIWLDPAKLQNFKLTPNDIGAAITAQNAQVSAGQLGGMPAIAGQQLNATITAQSRLQ
TPEQFENILVKTNSDGSVVRLKDVARVELGGENYGVVARYNGDPAAGIGIKLASGANALDTAEGVKKALEDLKPFFPEGLD
VAIPYDTTPFVSLSIEKVVHTLIEAVILVFVVMYLFLQNFRATLIPTIAVPVVLLGTFAILYTFGYSINTLTMFAMVLAIGLLVDD
AIVVVENVERLMTEEKLSALDATRKSMDEIKGALVGIAMVLSAVFIPMAFFSGSTGVIYRQFSITIVSAMGLSVLVALILTPAL
CATLLKPSHVHDKTSFIGRFFSGFNRGFDKTNRGAQGIVGRMISQSKRYLLIYGVLVGGMVYVFSSLPTAFLPDEDQGILFN
QVMLPAGSTIEQTLGVVEKVENHYLNDQAEAVGSIFTVTGFSFAGSGQNSAIGFVNLKHWDERQRDDLSVNAVAGKG
MGYFSTIKEAFVFAFPPPAIVELGTANGFNMFLQDRVGLGHDELLNARNMLLGMASKSPVLAGVRPNGQEDMPELQLDI
DLAKAEALGVTQTDINSTLSTAWGSRYVNDFIDRGRVKKVYMQGEADSRMVPEDLNKWYVRNNNGDMVPFAAFASS
HWSYGSPRLERYNGFSAMEIQGSAAPGYSTGQAMDEMERLVKQLPNGIASEWSGISYQERSSGGQAPLLYGLSLLFVFL
CLAALYESWSVPFAVMIIVPLGIFGAIMAALIGNLSNDIYLQVGLLTTIGLASKNAILIVEFAIHKMEEGLSLVDAAIAAVKLRL
RPVLMTSLAFICGVVPLAIASSAGSGAQNALGISIIGGTLAASTLVVLFVPLFFVLVRKTFSSKSAVATKESE

>MexFQ_P_haloplanktis
MKFSHFFIQRPIFAAMLSLIILIAGGISLFQLPVSEYPEVVPPTVVVTASYPGANPTVIAQTVATPLEQEINGTENMLYMFSQA
TSDGRMTLTVTFALGTDLDRAQVQVQNRVNSALPRLPQEVQRLGVVAEKSSPDLTMVVHLYSPENTHDTAYLSNYADL
NIKDEIARLSGVGDIRLFGGGKYSMRVWLNPDALASRELTASDVVSALRSQNQQVAAGSLGAQPISNDSQFQILLNVKG
RLNSIEEFEQVIIKVGEQGQLTRLSDIARVDLGQDTYALRAELDNQPALAMPIFQRPGSNAIELSDQVRETMARLAKDFPT
GVEYDIVYDPTVFVRGSIDAVITTLLEAIALVVIVVIVFLQTWRASIIPLIAVPVSLIGTFAVMQLLGVSINTLSLFGLVLAIGIVV
DDAIVVVENVERNIEKGLSPLEATRVAMSEVTGPIIAIALVLSAVFIPTAFITGLSGQFYKQFALTITISTIISAFNSLTLSPALAAL
LLKPHDAKPDAFTRLLNKLFGRWLFAPFNRVFNRGAKGYEKLVQKLIRMSVVVMVAYVALVGGTIKLFDSVPGGFIPQQD
KQYLVAIAQLPDAASLDRTEAVIKQMQQIALEVPGVAHTVAFPGLSVNGFTNSPNSGIVFTPLAPFSERTDPSLSAMAIAA
QLNQRFAAIDEAFVAVFPPPPIQGLGTTGGFKLQIEDRANKGFEALFNSLQAVIAKAQQDPALVGLYSSFRIQVPQMDIDI
DREQALIQGIPLDEVFNALQIYLGSVYVNDFNLFGRTYQVNAQADTDYRVDPEQILHLKVRNRAGNMVPLGSVLTVTPTI
GPDRVMHYNGYPSAELNGSPAPGYSSDQAQVAIEKVLADTLPSGIEYEWTEVTYQQVLAGNTMVYVFPLVVLLVFMVLA

AQYESLRLPLAIIMIVPMTIFSALIGVWFVGSDNNIFTQIALIVLVALASKNAILMVEFAKDQHETGLSHLDAMLAACRMRL
RPILMTSIAFTAGVVPLVLATGAGAEMRHAMGNAVFAGMIGVTVFGLLFTPVFYMLVANKKQKAQQVSHD

>AcrBF_H_defensa
MANFFIERPIFAWVIALIVMFLGALAITKLPVAQYPAVAPPTILIVANYPGADATTVQNTVTQVIEQNMNSIDNLLYMSSNS
DSSGNANITLTFNLGTDPDIAQVQVQNKLQRVMPALPQEVQKQGVIVEKSTGNFLMFLAFISENGSMKQEEIADYVASH
LKDPLSRINGVGDVQLFGAQHAMRIWLDPYKLNDFKLTPLDVMNAIQAENNQVAAGELGSAPTIAGQQLNASIVAQTR
LTSAKEFSQILLKANPDGSQVRLKDVANVELGAEGYKLNTLYNGKPATGVGIKLATGANALEASNALKEELIKLEPSFPAGF
KVVHPYDTTPFIKISIQEVIKTLLEAIVLVFLVMYLFLQNFRATLIPTIAVPVVLLGTFGVLSVLGYSINTLTMFGMVLAIGLLVD
DAIVVVENVERVMHEDGLSPKEASKKSMQQIQGALVGIALVLSSVFIPMAFLGGSTGEIYRQFSVTIVSAMVLSVLVALILT
PALCATILKPIDKDKKNKVSAFLGWFNHIFEKSCHHYTYSVKHILKGTGRYLIIYLFIVIGMCVLFLRLPTSFLPEEDQGVFVTM
VQLPVGASQERTDKVLSQVTDYFLTQEKDVIKSVLTLSGFGFNGQGQNMGLVFISLNDWSQRTEKKDTVSAVVARASRA
FSQIRDGLVFAFNLPPITELGVATGFDFELLDQRNLGHQKLTQARNQLLAMAAQHRDVLVNMRPSGMEDSPQFKIEVDK
EKARVLGLSLSDINTTLGSAFAGIYVNDFIDAGRVKKVFVQGAAPFRMLPEDINHWYVRNKQGQMVPFSSFSTTQWKYN
SPRLERYNGFPSMKIQGQAAPGKSTGEAMALMQELAKKLPNGIGYDWTGVSFQERMSANQSLFLYAISLIFVFLCLAALY
ESWSIPFSVIMVVPVGVLGSLLAAYLGGLDNDVYFQVSLLTTIGLSAKNAILIIEFSKNLIQKEGKGLIEATLSAVRMRLRPILM
TSLAFMLGVLPLAMSHGAGSGAQHALGVGVIGGMIASTVLAIYFVPVFFVVFLRRFGQKTHLGIQHSDKN

>MexB_S_maltophilia
MARFFIDRPIFAWVIAIIIMLAGGLALFKLPVSMYPNVAPPAVEISATYPGASAKVVEDSVTQIIEQNMKGLDGLIYFSSNSSS
NGQATITLTFESGTNPDIAQVQVQNKLQLAMPLLPQEVQRQGINVAKSSSGFLNVIAFVSENGSMDANDIADYVGSNV
VDRLSRVPGVGNIQVFGGKYAMRIWLDPNKLHTYGLSVAEVTAAIKAQNAQVAIGQLGGAPSIKGQQLNATINAQSRL
QTPEQFRNIIVRGAQDGAELRLGDVARVELGAESYDFVTRYNGQPASGLAVTLATGANALDTAAGVNAALEDMKSFFPA
GLKAEVPYDTTPFVRVSIKGVVQTLIEAIVLVFVVMYLFLQNFRATLIPTIAVPVVLLGTFGVLAMLGFSVNMLTMFAMVLAI
GLLVDDAIVVVENVERIMSEEGLSPLEATRKSMGQITGALVGIGLVLSAVFVPMAFMSGSTGVIYRQFSATIVSAMALSVLV
AIVLTPALCATMLKPLKKGEHHVAHRGLAGRFFNGFNRGFDRTSESYQRGVRGIIHRPWRFMGIVAALFVLMGVLFVRLP
SSFLPNEDQGVLMALVQAPVGATQERTLESIAALENHFLQNEKDAVDSVFSVQGFSFAGMGQNAGMAFVKLKDWSER
DANNGVMPITGRAMAALGQIKDAFIFAFPPPAIPELGTASGYTFFLKDNSGQGHEALVAARNQLLGLAAGSKKLANVRP
NGQEDTPQFRIDIDAAKATSLGLSIDQINGTLAAAWGSSYIDDFVDRGRVKRVFVQADQPFRMVPEDFDLWSVKNDKG
EMVPFSAFATKHWDYGSPRLERYNGVSAMEIQGEPAPGVASGDAMAEIEQLAKQLPAGFGIEWTAMSYQERQAGSQT
PLLYTLSLMIVFLCLAAMYESWSVPTAVLLAAPLGILGAVLANTFKGLERDIYFQVAMLTTVGLTSKNAILIVEFAKENLEKGA
GLIESIMHAVRDRLRPIVMTSLAFGMGVVPLAISTGAGSGAKQAIGTGVLGGMIVGTVLGVFFVPLFFVVVQRVFKRKSTT

>AcrDMexB_S_maltophilia
MVRFFIDRPIFAWVIAIAVSLLGLLAILILPVDRYPQIAPPTITIRATYTGASSQTVENAVTQVIEQSQQSLDHLMYMTSTSAS
DGSAQVNLVFATGTNPDTAQVQVQNQLQAAMATLPQAVQQNGLTITKSSGSIFEVLSFTSEDGSMDNFDVANFMEAR
IDDQISRVSGVGNIQPIGQEYAMRIWLDPEKMRQYALMPSDIETALQAQNTDVSAGELGGQPALKGQQLDATVTARSR
LHTPEQFAQVVLKADANGSVVRLGDVATIGLGPESYDSISTFNGKPSASLGIELNAGANAIAVSKAIDARLQQLQKYWPH
GYTAHVAFTTTPFVTISLKEVVITLIEAIILVVLVMYLFLQNWRATLIPTIAVPVVLLGTFGVLAAFGYSINTLTMFALVLAIGLL
VDDAIVVVENVERVMTFEGLAPKPATLKAMGQITGALVGIVLVLTAVFLPMAFFSGVTGVIYRQFSVTIAAAMILSVLVAM
TITPALCGSILHQIPKGGHPHGDHGGEPSLLGKFFIWFNHRFERTSNGLRHRVDRFLGRRTLGVLFYLVLSVATGLLLWHLP
GAFLPDEDQGMLNALVKLPAGSTLEQTRAVMDRLSAVAVKDDGVLSIQATAGFSVTGSGQNVGQAFIRLKDWDDRKD
DADTIAARLTRAMASVPDAQVFITSPPAILGLGDAGGFTLELQDEGGAGHAAAVAARNTLLKEAAKDPKLVNVRYASLED
APVYAVKVDDAKAQAMGVNPQDVNDTLNAALGGDFVNNFIYKGRIKKVFIQGTAEARMQPQDIERWSVRNQAGQM
VPLSSLISTHWTSAPAALQRYNGISAMEITGQPAPGVSSGEAMAEIARLADTLPEGFSHAWSDMAYQEQLSGNQAPMLY
AISLLFVFLCLAALYESWAVPFAVMLAVPVGVFGAVLMMNLRGLNNDVYFQVGLLTTIGLAAKNGILIVEFARILEQQGRD

TRDAILQAVYLRLRPIVMTSLAFLMGVLPLVFATGAGSAARRSLGTGVAGGTVASMVLGMFFVPLFYLLVRRLFPGRAPAD
ATVPETSP

>MexDY_S_maltophilia
MPKFFIEHPVFAWVVAILISLSGVIAILNLGVESYPNIAPPQVTVSATYPGASADTTEKSVTQVIEQQLTGIDHLLYFSSSSSS
NGRAQITLTFETGTDPDIAQVQVQNKVSLATPRLPSEVTQQGVVVAKANAGFLMVVALQSDTPTINRDALNDIVGSRVL
DQVSRIPGVGSTQQFGSEYAMNIWLNPEKMQGYGLSASQVLAAVRAQNVQFAAGALGSDPSPEGQHFTATVSAEGRF
SSPQEFENIILRANADGSRVLLKDIARVAFGANNYGFDTQYNGKPTGAFAIQLLPGANALNVAEAVRAKMDELQPSFPAG
VTWFSPYDSTTFVKISIQEVVKTLFEAVFLVFLVMLIFLQNFRATLIPTLVIPVALLGTFLGMWMIGFTINQLTLFAMVLAIGIV
VDDAIVVIENVERIMTEEGLAPKPATQKAMTQITGAVVAITVVLAAVFIPSALQGGAAGEIYKQFALTIAISMAFSAFLALGF
TPALCATFLKPTHNDNPNIVYRTFNKYYDKISHTYVGHITSAVRHAPRWMILFVVLTALCGFLFTRMPGSFLPEEDQGYAL
AIVQLPPGSTKGQTNEVFAQMRGILEKQDGYEGMLQVAGFSFVGSGENVGMGFIRLKQWEERKFTAPEFIQNMNGAFY
GIKEAQIFVVNLPTVQGLGQFGGFDMWLQDRSGAGYEQLTQARNILLGQAAQKPDHLVGVRPNGLENAPQLQLHVDR
VQAQSMGMSVSDVYSTIQLMLAPVYVNDFFYEGRIKRVTMQADGPYRTGQESLKSFYSPSSLTKNADGTNSMIPLNTVV
KSEWVSAPPSLSRYNGYSAINIVGSQAPGTSSGEAMQTMESIVNDDLPAGFGYDWSGMSYQEILAGNAATLLLVLSIVVV
FLCLAALYESWSIPVAVLLVVPLGVLGALGLSMLRGLPNDLFFKIGLITVIGLAAKNAILIVEFAVEQRAAGKNLRDATIEAAR
LRFRPILMTSFAFIMGVIPMAISTGAGANSRHAIGTGVIGGMLFATLLGLLMIPVFFVVVRRMLGDKLDEPSKEFMERQRDA
DAAHRPDR

>AmrBAdeB_S_maltophilia
MARFFIDRPVFAWVLAIFIILAGVLAIPRLAVERYPAVAPPSVSIYASYPGASPQTLNDAVVGLIERELSSVKHLLYFESSVDTS
GEASITATFKPGTNPELAQVDVQNRIKAIEPRLPRSVRQNGLFVEAADSGFLMLVGLRSPDASVSEAALGDFMARNIIEELR
RIDGVGRVQLFGAEQAMRVWLDPTRLTGYGLTMGDVAAAIEQQNLEISPGRIGDSPGVPGQRITVPLSADGQLSTPEQF
AAIVLRAGADGSRVVLGDVARVELGAQSYAWGTREDGHPATAAGVQLRPGANAVRTASAVRERMAELAPLLPRGVESS
IPFDTAPFVKISIQKVVQTLLEAMLLVFAVMYLFLQNWRYTLIPALVAPIALLGTFAVMLALGFSINVLTMFGMVLAIGIIVDD
AIVVVEGVERIMAEEGLPPREATIKAMRELTGAVIGITLVLTAVFIPMALASGSVGAIYRQFTVAMAVSILFSALLALSLTPALC
ATLLRPNTHGHHGRGGLFGAFNRGFERMTGRYQRGVASVLQRSGRVMGVFAALVAALLLGLHWLPGAFLPEEDQGYF
MTSIQLPAEATAERTLAVVEAYERHVASRPGIGSNQSILGYSFSGSGPSAALTYTMLKDWGERAGATAAGEVEAAQKAM
ATIAEGEVMSVMPPAIDSLGRSSGFSLALQARTGQSQAELRAALQQLLKLAEASPLLSEVHADGLPAGTSVRLDIDRAKAE
AMGVGFEQISSTLSAAMGSQYVNDFPNKGRLQQVILQADAPHRMELEDVLKLYVRNSEGGMVALSELVTPHWTEAPLQ
LQRYLGFPALNLSGAPASGVSTGQAMAEMERLAEKLPSGFALQWTSQSLQERESGAQAPWLLLLSMLVVFLVLAALYES
WSIPLAVMLVVPLGLLGAVAAVLLRGMPNDVFFKVGMITVIGLSAKNAILIVEFARQLQQQGRGLVEAALEAARLRLRPIV
MTSLAFALGVVPLMLAQGASKETQQAIGTGVFGGMVSATVLAVFFVPVFYVVVQGARQWIAQRLRRRRPMPEGSVDG
TGHR

>MexFQ_S_maltophilia
MDFSRFFIDRPIFAAVLSIVIFAAGLITIPILPISEYPEVVPPSVVVRTVYPGANPKVIAETVATPLEEAINGVEGMMYLKSVAGS
DGVLQMTVTFRPGTDPDAAAVKVQNRVAQAQARLPEDVRRQGVTTQKQSPTFLMVVHLTSPQGKYDTLYLRNYARLH
VKDALARIPGVGDAQIFGGGDYAMRAWLDPDKVASRGLTASDVVRAMREQNVQVSAGQLGAEPLPDSKFLTLINAQG
RLKTEKEFGDIVLKSGTDGEIVRLSDVARLELGAGDYTLRSQLDGKNAVGIGIFQSPGANALEIRDQVISTMDEMQKTMPA
DVKYEAVYDTTIFVRDSIKAVVSTLLEAIALVVLVVILFLQTWRASIIPLIAVPVSVVGTFAALYLLGFSINTLSLFGLVLAIGIVV
DDAIVVVENVERNIEEGLSPTAAAHQAMREVSGPIVAIALVLCAVFVPMAFLSGVTGQFYKQFAVTIAISTVISAINSLTLSP
ALAARLLKAHGAPKDGPSRLIDRLFGWVFRPFNRFFKSSSEKYERGVARILGRRGAVFVVYAILLVGTGVIFKLVPPGFIPTQ
DKLYLIAGVKLPEGSSIARTDEMLRKVAKIAQETDGVAHTISFPGLNALQFTNTPNTGVAFIPLKPFAERHGRTAAQINAEIN
QKIAGLQEGFAFAMMPPPILGLGNGNGYQMFIEDRGNLGYGALQNAVQAMQGTVAQTPGMAFPISSYQANVPQLDA
EVDRVKAKAQGVQLTELFDTLQTYLGSAYVNDFNQFGRTWQVIAQADGPFRETVEDIGKLRTRNDRGEMVPIGSMVTIK
QTFGPDPVLRFNGYPAADLAGEADPRLLSSAEAMTKLTEIAGKVLPVGMTTEWTDLSYQQATQGKAAFIVFPVAIMLAFL

VLAALYESWSLPLAVILIVPMTLLSALFGVWLTGGDNNVFVQVGLVVLMGLACKNAILIVEFARELEMGGKGIVEAALEAC
RLRLRPIVMTSIAFIAGTVPLVLSHGAGAEVRSVTGITVFAGMLGVTLFGLFLTPVFYVALRKFVTRNGGGQLVQHGEPTIH
H

>AcrBF_I_loihiensis
MAKFFINRPIFAWVIALLTMMAGAIAMLQLPIAQYPTIAPPAVEIQVSYPGANAETVSNTVTKVIEQNMTGIDNLLYFSSQS
NFGNGTVSLTFASGTDPDIAQVQVQNKLSQATPQLPQAVQAQGITVSKASSSFLMVAALVASDDRYSQTDLSDYIASNI
QDPIARTTGVGNVQLFGSPYSMRIWVDPSALVNYGMTMADLKSAIQAQNAQVAAGELGGTPAVEGQRLNATIIAQTR
MSDVSEFENITLKVLSDGSQVRLKDVAEVEKGAENYSIQANYNGKPATGLAINLQTGANALETADAVKARMAELEEFFPD
GMELVTAYDTTPFIKISISEVAQVLIEAIILVFLLMFVFLQNLRATLIPTLAIPVVILGTMGVMALAGFSINTLTMFGMVLSIGLL
VDDAIVVVENVERLMSEEGLSPKQATIKSMGQITGALVAIGVVMAAVFAPMAFFPGSTGAVYRQFSLTIITSMGLSVLVAI
VFSPALCATLLKPVKHDKSSGWGPLGWFNRTLEKWTDKYTNTIHSILRRTVRFGVLYLGIIGILVFLFMKLPAAFLPDEDRGV
FLTQMQLPVGSTIEQSVNTMDKIENYYLNQEDAVQSVFSVVGFSFSGRSQANGIAFVRLKDWSERDESQSVNAVIGRAF
GFFAGIKEAMIFAFNLPPIPELGVSSGFNLFLQDRGNLGHAGLLEARNQLLGMAGQNDKLVQVRPNGLEDAPQLRIDIDF
EKAQALGLTITNINETLSTAWGSSYVNDFIDRGRVKKVYLQGETESRMLPEDINEWYVKNNQGDMVPFSSFSSWEWTYG
AQRVESYNGVAAMEIQGQAAPGVSTGEAMIEMEKMIAKLPPGIGFEWTGISLQERQSGDFAPILYSVSLIVVFLFLAALYES
WSIPFSVMLVVPLGILGAVVAVMLRGISNDVYFQVGLLTTVGVSARNAILIVEFAKDLQAQGKELLDATLEAVRLRLRPILM
TSLAFIFGVMPLALSDGAGAASRNAIGTSVIGGMIGGTVLAIFFVPLFFYVVRRIFPPKQSEE

>AcrBF_P_arcticus
MSRFFINRPIFAWVLAVLVMLIGVISVLNLPIEQYPRIAPPTISVSASYPGANAQTVENSVIQIIEQRMKGLDGLMYMSSSSS
SNGSASVTLTFENGTDSDTAQVQVQNKLQAAMSSLPESVQRQGVNVNKSSSSFMMVQAFISEDGNMDRSDIGDYINS
NVLDAISRVEGVGEVQVFGSTYAMRVWLDPSRLRSYNMVPSDVVNAIRAQNTQVSAGQLGQAPADTDQQVINATVT
VQSYLQTPEEFQNILLKTDTAGAQVRLGDVADVEIGSENYSVVSLYNGQEAAGLGISLAAGANALETREAVGVRMAELEK
NFPAGLTSVVPYDTTPFVRLSIEQVVMTLIEAIVLVFIVMFLFLQNWRATIIPTLAVPVVLLGTFGVLYIAGFSINVLTMFAMV
LSIGLLVDDAIVVVENVERILEEDPHISIKDATVQSMGEISKIVIGIALILSAVFIPMAFFGGSTGVIYRQFSITLITSMVLSAMVA
LVFTPALCVTLLKRGKSHEKGNSENQKGFFGWFNRSFTKTSQSYEKFIGKSFRFKWLYLIVYAAIIGIMAVVFLRIPGSFLPEE
DQGIMFTLVQLPAGSTLDETQDVLDKIRNYYDTQESDNIASVFTIAGFSFAGQGQNMGLAFVRLSDWEERSGDENTAQA
VAGRAMGYFFTQLNEAQVFAIVPPAITELGNASGFNLMLQDSGNLGHDGLLEARNMLLGMAAQNEQVAGVRPNGQE
DSPQLKIDINQEQAAAYGLSLSNINSVISTAWGSSYVNDFVDRGRIKRVYVQGEASSRTNPDDIGKWYVRNDMNDMISF
DAFSSSEWQSGSPRLTRYNSLPSMNIQGSAAPGLSTGEAMSSMEAMIDKLPEGISYEWTGMSLEEQKSGAQAPILYALSIL
VVFLCLAALYESWSIPFSVLLVIPLGVLGAVIFTWLRGFANDIYLQVGLLTVVGLSAKNAILIIEFAKERQEAGYSLTEAVMTA
ARQRLRPIIMTSLAFGLGVVPLFLANGPGSGSQNAIGTSVIGGVITATLLGIFFIPMFYIWVRSAFPHKYENNKPNDKDDSN
GTPDSHNPMPSDNDQPASLGDNT

>MexDYB_Marinomonas
MLAHFFINRPVFAWVISIAIMLAGLASILNLPISQYPNVAPPTISISATYSGASAETVENSVTQILEQQLTGLDGLLYFSSSSSS
SGRTSINVTFEQGTDADIAQVQVQNKIQQITSNLPESVQRNGVTVRKSNSDFLMVAAIYDETDTDTANDISDFLVSSVQD
PVSRVDGVGSIQVFGAEYAMRIWLDPLKLASYKLMPSNVSSAINAQNTQVAAGSLGAYPIIKGQELNVTVTAQSKLQTAE
QFRDIILAYDDSGATVRLSDVAKVELGSESYGYIPRLNGHPASGMAVMLAPGANALSTSEAVKKVLNGLSSTLPDGYKLSF
PVDNTAFIRISIEEVVKTLFEAIVLVVIVMFIFLQNWRATLIPAIAVPVVLLGTFGVLEIFGFSINTLTMFGVVLSIGLLVDDAIVV
VENVERVMEEDKLSPKEATIKSMKEITSALVGIAVVLSAVFLPMAFFSGSTGVIYRQFSITIVASMTLSVIVALTLTPALCATLL
KANHGGNNKGLGGWFNRNFDRATDKYFGSVSSIIKKPVRWMLGYGVIVAGLAGLMVTLPSGFLPTEDQGRVMVMVSL
PEGAAMSRTDKVMRDVESYFMNQEKDNVEDIFTISGFNFMGSGQNAGMAFVALKDWSKRVGPANSASAIVGRSYGAF
AGIRDARIFSLIPPSIQGLGQSNGFTFQLQASGNTDRATLLEMRNALLQNANTSQLLTGVRLGSDSEAPQLHIDIDQEKAS
ALGLSMSDISSTLSSAWAGSYINDFIDRGRVKTVYMQGEAQYRSSPEDLQHWYVRGNDNTMTPFSAFAKSEWTYGPKSL
TRFNGLASYEIQGSGAPGVSSGAAMDELQNIADALPGGVTGSWSGLSYQERLSSGQTQILYAISILVVFLCLAALYESWTV

PFSVILVIPLGVIGSAAAAHLRGLENDVYFQVALLTTIGLSSKNAILIVEFAEAAYRRGLNVWDAAAQAARLRLRPIIMTSMA
FMVGTLPLALSSGAGANSRISIGSGIVGGTLTATVLAVFFVPLFFVVVRRIFPKRPEALSSNENAE

>AmrBAdeB_MexDYB_Marinomonas
MSNFFIARPNFAWVMAIFILLAGLLALPTLPVSQFPVVAPPQVEITATYPGASAQVLVDSVTSVIEEELNGAKGLLYFDSSS
NANGIAQITASFEPGTDPDLAQVDIQNRLKKAEARLPSSVIAQGVQVDQASSGFLMIYALTFKGDDSSKSIIALADYAARNI
NNNIRRVKGVGKLQYFGTEAAMRVWVSPEKLLSYGLSIGDINNAIRNQNVQVPAGSFGGRPADDAQELTASLVVKGFLE
DPEEFGNIVLRSNVDGSAVYLKDVARVELGKQDYNFETRLNGHKAIMGAVQLSPGANAIETVKAIKARAAELEANFPDDI
QISFPYDTSKFVSAAIEKVIYTLIEAIVLVFLVMLLFLQNLRYTLIPTIVVPVCILGTFAVMSLMGFSVNMMTMFGMVLAIGILV
DDAIVVVENVERIMVEEGLPPKDATIKAMGQVSGAIVGITLVLSAVFVPLAFMSGSVGIIYQQFSMSLAVSIMLSGILALTFT
PALCATLLKPINKEAHHEKGGFFGWFNRKFARLTHRYSILNGMLVNRSGRFMLVYLVLIIGLGYAYLRLPESFVPTEDQGYM
IVDLQLPAGATFSRTNETMKDAEKFLGQRDAVENVVTVMGFSFSGMGANAGLLFPTFTDWSDRQQSLFDETMAYNQF
AGSITDGRSMAVEPPSIEGMGNSGGFSLRLQDRSNLGREALTNARNIVLGQANASPIIAYAMMEGLEDAPQLRINIDRQK
ATALGVGFESINNAVSTAYGSASVNDFTNAGRLQRVVVQADVADRMTPERVLELQVLNDEGNLVPMRSFASSSWEIGP
VQVSRYNGYPTYKISGSPMPGHSSGEAMAEIERIISTLPKGIGYEWTGLSYQEKAAGSQAPMLLTISILVVFLLLVALYESWSI
PFSVILIIPIGALGAVAAVYLMGMSNDVYFKVGLITIIGLSAKNAILIVEFAKDLYQQGHSLKESAIQAAKLRFRPIIMTSMAFIL
GVVPLTIATGAGAASQHSLGVSVIGGMISATVLGVLFVPVFFTWVLGRFKASNKTV

>AmrBAdeB2_MexDYB_Marinomonas
MAQFFIHRPIFAWVLALFIILGGVLSIPQLPIARYPSIAPPSVSIMATYPGANPQTMNDAVLSLIERELSGVKNMLYFESSAD
GSGSASITVTFKPGTDPKLAQVDVQNKIKAVEARLPQAVRQNGLSVESATSSFLMIVGLKSDDGRFTETSLSDYMSRNLLE
EFRRIDGVGNVQLFGSEQAMRIWVDPNKLTSYGLTMADLTTAITQQNTQIAPGSVGASPSVIGQRVTVPLTVQGQLSSP
EEFASIVVRARTDGSKIVLSDVARVEIGAQSYGFSNRENGKVSTSAAIQLAPGANAVSTSNKVLARMEELGRTMPAGMSY
SIPFNTAPFVKVSIEKVIYTLLEAMALVFLVMFLFLQNIRYTLIPAIVAPIALLGTFSIMLLAGFSINVLTMFGMVLAIGIIVDDAI
VVVENVERIMAKEGLSPKDATVKAMRQITGAVVSITLVLTAVFIPMAFASGSVGVIYQQFSLSMAVSILFSAFLALTLTPALC
ATLLKPHTEKDFKKTGFFGWFNRTFDSMTKGYVSCVGKLVARTGRMMMIFIVIVAILILALRQLPTSFLPEEDQGYFMTTIQ
LPADATSERTLDVVEVFEEYIASRPAIASNLSILGFGFSGSGSNAAMAFTVLKDWKDREYTTLQEEVSLAQQAMVDISEGSA
MSLMPPAIDELDTSSGFSMRLQDRANRGYEALKEAETKLLTLAAQSKVVTGVYPDGLPSGVSIQLNINRLKAEAMGVSFSS
ISDTLSTAMGSTYVSDFPNAGRMQQVILQADASARMQIEDVLNLYVRNISGGMVPLSEVVTPVWTETPLQLTRYQGFPA
IRISGAAAAGFSSGDAMQEMERLVDQLPAGFAVAWSGQSLQERQSSSQAPLLMALSMLVVFLVLAALYESWSIPLSVML
VVPLGLLGAVIAVLLREMPNDVFFKVGMITVIGLSAKNAILIVEFAKQLREQGEGLIESAMIAARLRLRPILMTSLAFGLGVVP
LVIASGASAETQRAIGTGVLGGIISATVLAVFFVPVFFVFVIGLQERISRWRALD

>LpeB_lpp2880_L_pneumophila
MKLTSYFIKHPVITIILNAMIVVLGLLCLYNLSVREYPDINFPTITVSASYPNASPDLVETAITNVLEDRLAGIEGLETITSQSSA
GYAQITLMFRSGTSMDRALSTTQDAVGVAKALLPPEVRSPTVERQRKSNGLPFIGISLESTSKDFGELTHYANLNLKNVFRS
VQGVASVEVWGQPYTYNITLQPEKLFSFGVNVDEIVNALAKNRISLPAGNYRNKIPSTLNSELKTREDYENLLIKANSKHPIL
LKSLANVALETDNSQMRVRVNGHAGVVLSINKANEANPIEVSKEIRKVIKGLQQGLPKDLKINTIIDQSDFINASIKNIRSAI
GEAIFLVLIIVFLFLRNIKATIIPLITIPISLLGSLLFLKLFGFSINLMTLLAMVLAIGLVVDDAIIVLENIWRHIESGLSPLDAALKGA
REIGFAIIAMTFTLSSVYLPIAFIQGMLGQLFVEFAVALAGSVFISGIVALTLSPLMCANLLSASSKNWWPQFDNALEKLTKH
YSNILQFILKHQKITLLTALISVVACFGFYNLISHETAPKEDRGLIGIYIPPVAGEDIGMLDNKTGKLEKKLDAIPEANNRLTFIG
DWGGSIVLPLKPHAQRHRSANQIVEKLRPSFNHFPSMDPHVWSWDTGLPGVDDAGSGSELTLVISTPDHFRQLFDETEK
LKSVLDKSKLFAAVNYDLRLDTMGYNIDLDYNQLAKLGLTANQVAKTIEVFFSGDKSQTFEKDGVVYNISIKGDSSPWTLN
ELYLTTNEGKSVSLGAITKMQPKAQPATLDHYQQMRSTTLHVQLHKGDSMAKAIEKLWGTTKEVMPQHYKLTWIGAAK
AFHESSNSMLFLFFLSLAFIYAILCTQFENFTDPFIILFTVPLACSGALLFTYLFGQSLNIYTQVGLITLIGLISKHGILIVEFANKLH
KDGASLPEAIQKSAALRLRPILMTTGAMVFGAIPLVLSHDAGSESRHAIGTVLISGLGLGTLFTLFVLPAVYNIIKSKLIDMQK
TGGKKRCSKE
