# Supplementary material for: Ever-Adapting RND Efflux Pumps in Gram-Negative Multidrug-Resistant Pathogens: A Race against Time
Source: Antibiotics (Basel). 2021 Jun 25;10(7):774. doi: 10.3390/antibiotics10070774 (PMC8300642; doi:10.3390/antibiotics10070774)
Supplement: Supplementary file 1 [file antibiotics-10-00774-s001.zip › Supplementary Figures.pdf]

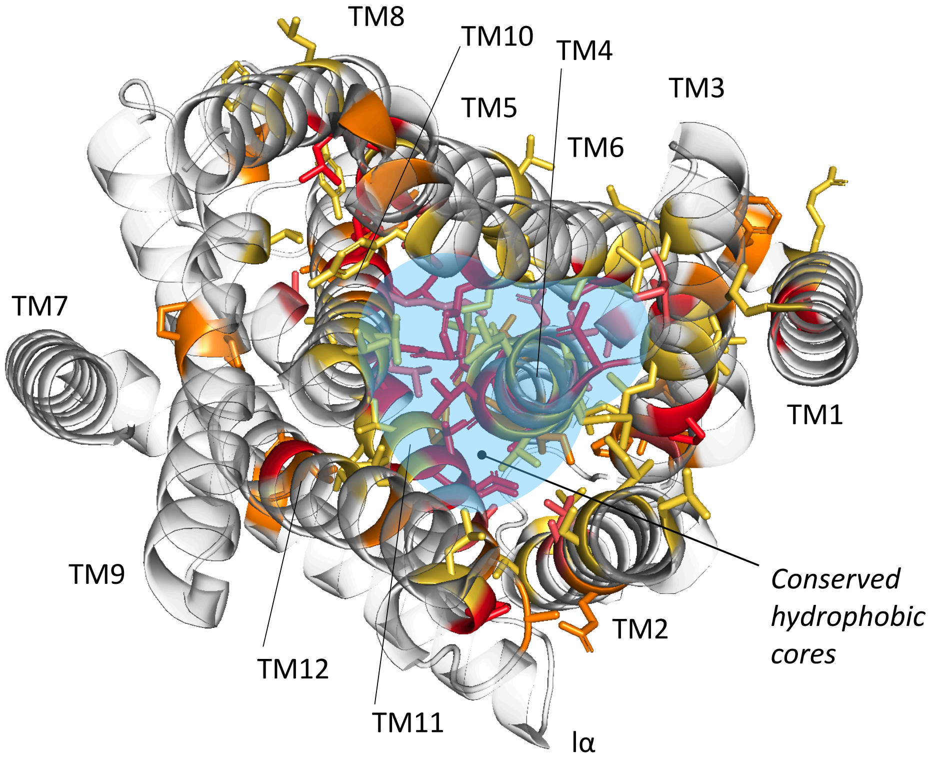


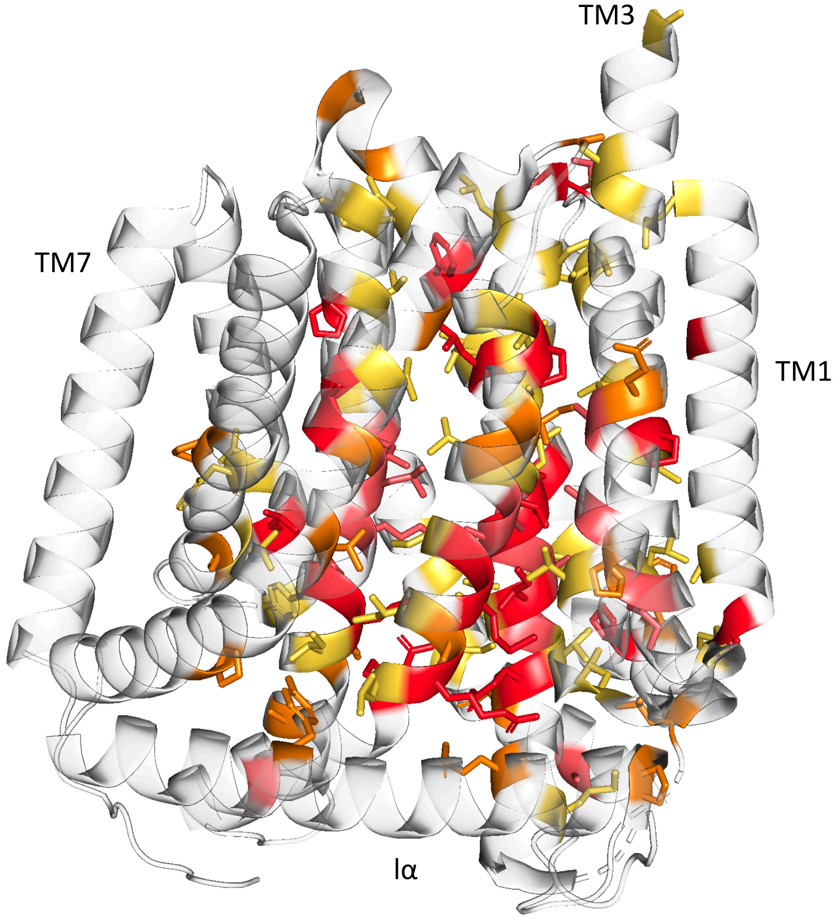


**Figure S1. Additional images of the TM-domain.** Top, top-down view; bottom, side-view. Coordinates from high resolution AcrB-Ec (using DARPin inhibitors), PDB accession code 4DX5 [54]. Red, conserved between 135 pumps; light red, conserved within 19 selected pumps and also highly conserved in all 135; orange, conserved in selected 19 pumps; yellow, highly conserved in all 135 pumps; blue, conserved hydrophobic clusters.

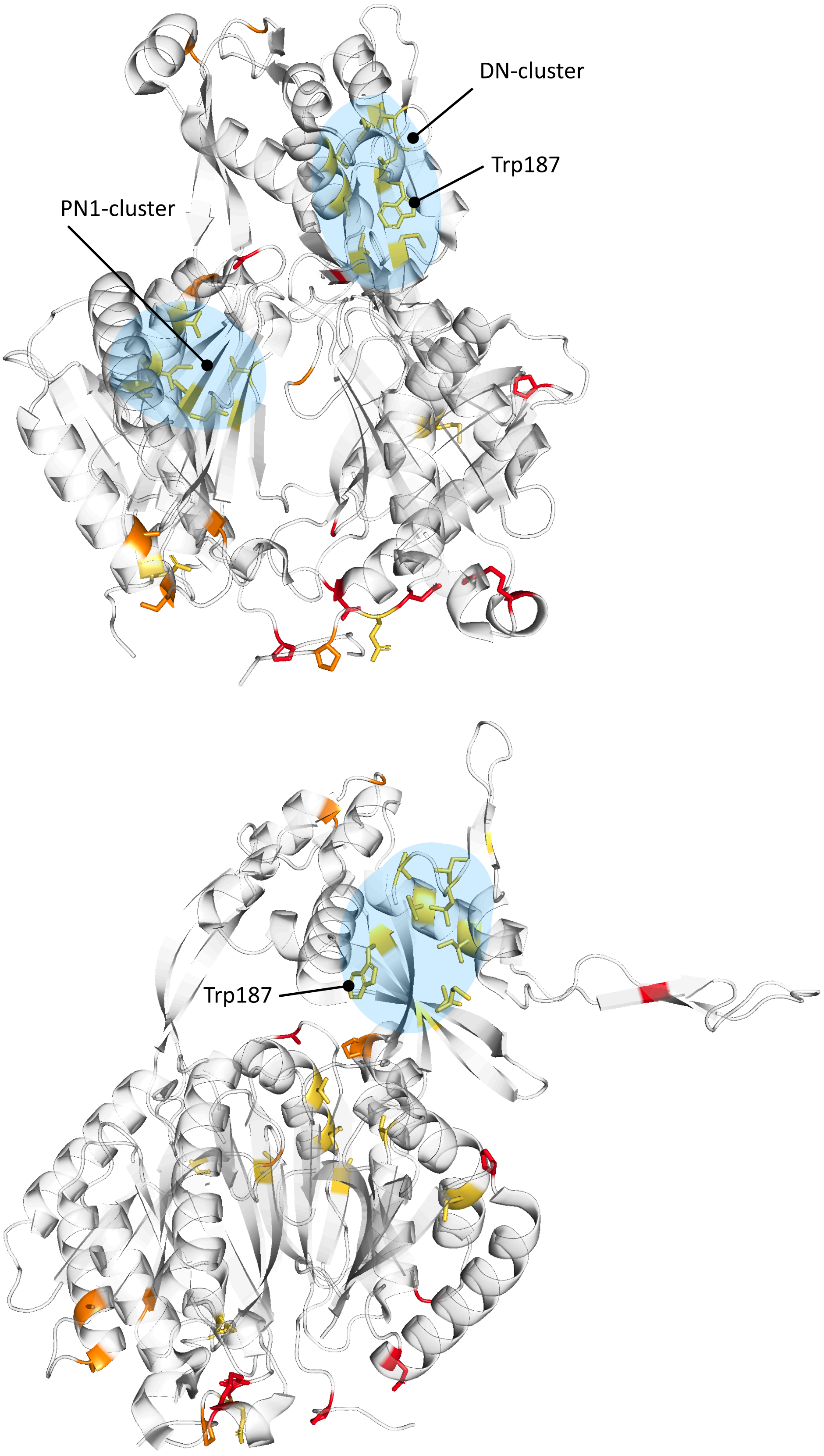


**Figure S2. Additional images of the porter domain.** Top, side-view; bottom, 90 degrees turned side-view. Coordinates from high resolution AcrB-Ec (using DARPin inhibitors), PDB accession code 4DX5 [54]. Red, conserved between 135 pumps; light red, conserved within 19 selected pumps and also highly conserved in all 135; orange, conserved in selected 19 pumps; yellow, highly conserved in all 135 pumps; blue, conserved hydrophobic clusters.

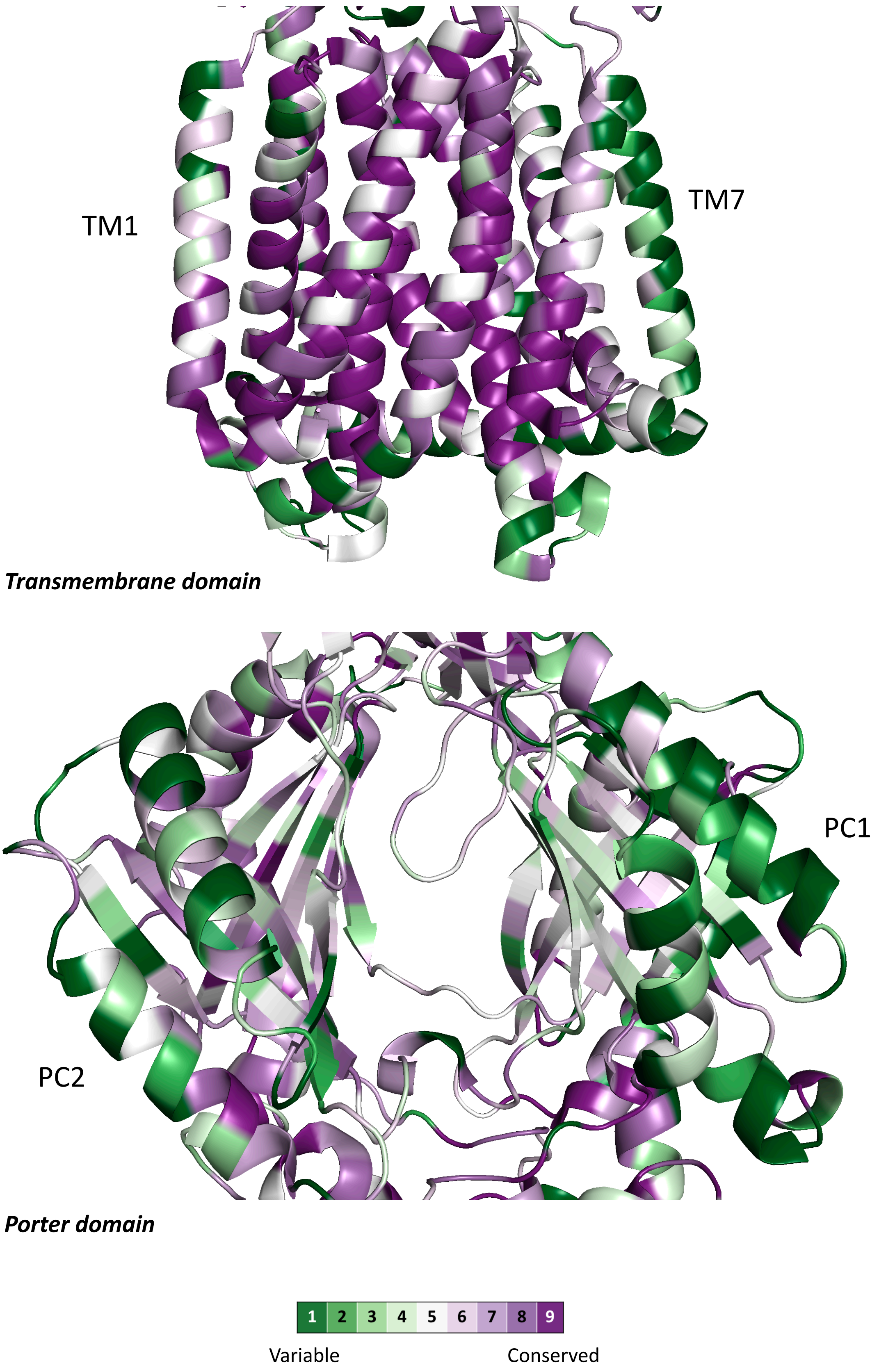


**Figure S3. Additional images of the ConSurf output.** Top, TM-domain; bottom, porter-domain. Coordinates from high resolution AcrB-Ec (using DARPin inhibitors), PDB accession code 4DX5 [54]. Side-view of AcrB-Ec showing conservation of the monomer based on 135 MDR-type RND pumps, analyzed using ConSurf [67,68]. Conservation is relative (most conserved "category 9" (dark purple) ranges from 50 to 100% conserved depending on the residue, Table 3 and S1).

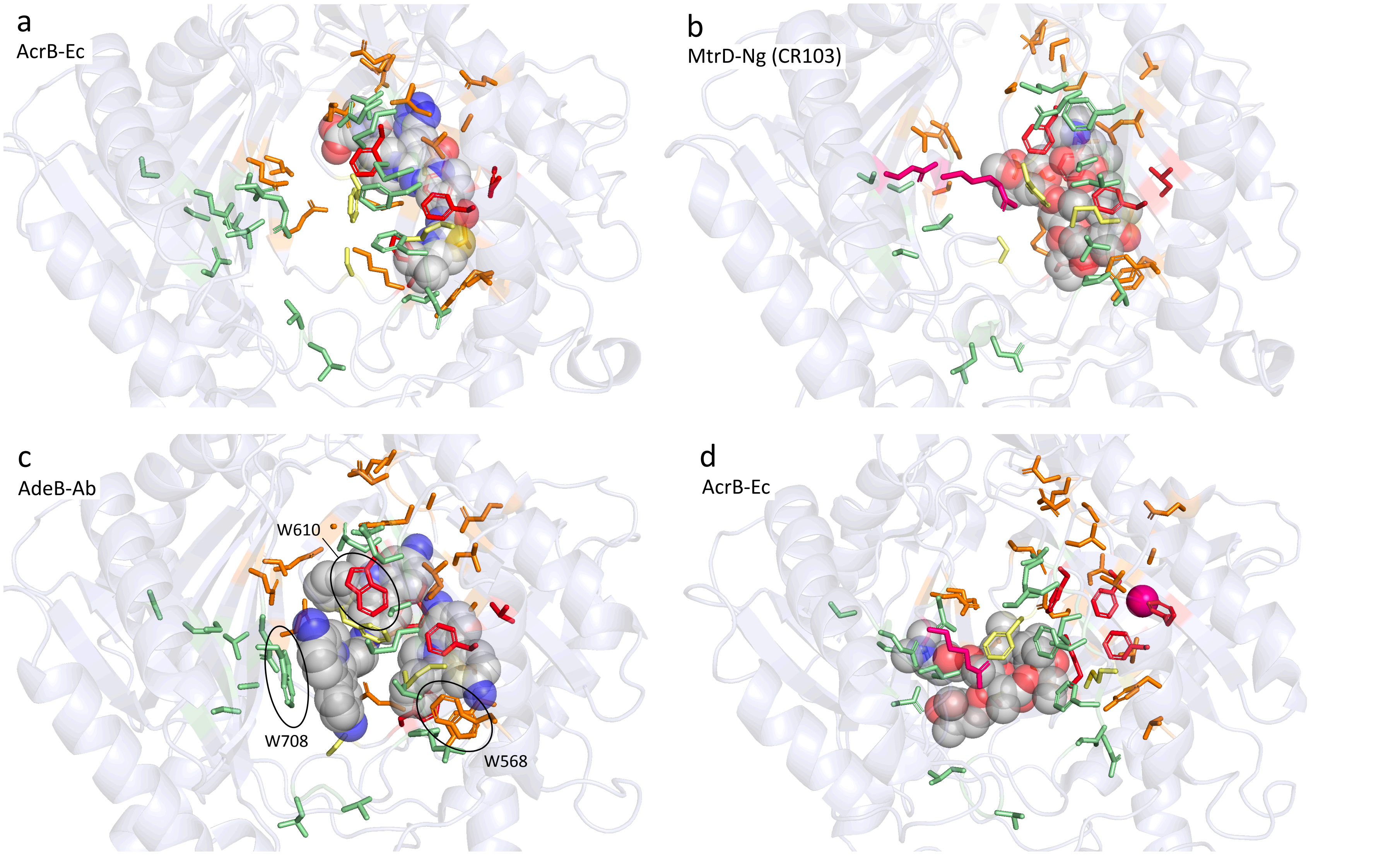


**Figure S4. Front view of the drug-bound structures of AcrB-Ec, MtrD-Ng and AdeB-Ab.** Green sticks show the PBP, orange sticks show the DBP and red sticks show the hydrophobic pit. (**A**) ABI-PP bound in the Binding monomer of AcrB-Ec (PDB accession code 3W9H [36]). (**B**) Erythromycin bound in the Binding monomer of "CR103" MtrD-Ng (PDB accession code 6VKT [43]). (**C**) Ethidium bound in the Binding monomer of AdeB-Ab (PDB accession code 7KGG [41]). (**D**) Erythromycin bound to the Access monomer of AcrB-Ec (PDB accession code 3AOC [51]).
