# Supplementary material for: Ever-Adapting RND Efflux Pumps in Gram-Negative Multidrug-Resistant Pathogens: A Race against Time
Source: Antibiotics (Basel). 2021 Jun 25;10(7):774. doi: 10.3390/antibiotics10070774 (PMC8300642; doi:10.3390/antibiotics10070774)
Supplement: Supplementary file 1 [file antibiotics-10-00774-s001.zip › Table S1.pdf]

Table S1. Overview of residue conservation based on 135 MDR-type RND pumps.

The table details the residue variety in % for each position in the query sequence (AcrB-Ec). Each column shows the % for that amino-acid, found in the position based on AcrB-Ec. Analysis after ClustalOmega [65] and output data by ConSurf [68]. Efflux pumps selected from Zwama *et al.* (2019) [17].
Colors are per Figure 3a of the main article, with green being most variable and purple being most conserved.

Position (#)	AA (%)	Percentage	ConSurf Category	A	C	D	E	F	G	H	I	K	L	M	N	P	Q	R	S	T	V	W	Y
1	M 54.815	54.815	8					31.111			6.667		6.667	54.815							0.741		
2	A 31.852	31.852	7	31.852									2.963	0.741		14.074			31.111	18.519	0.741		
3	R 25.185	25.185	5	1.481		18.519	2.963		0.741	4.444		17.037	0.741		16.296		11.111	25.185	0.741	0.741			
4	F 71.852	71.852	7					71.852			12.593	0.741	2.963			1.481				2.222	3.704		4.444
5	F 97.037	97.037	9		2.963			97.037															
6	I 88.889	88.889	9								88.889									0.741	10.37		
7	D 42.963	42.963	2	0.741		42.963	5.185			8.148	0.741	8.148			8.889		5.926	17.037	0.741	1.481			
8	R 93.333	93.333	9							3.704		2.222					0.741	93.333					
9	P 100.000	100	9													100							
10	I 56.296	56.296	8								56.296	3.704			2.222			0.741			37.037		
11	F 76.296	76.296	8					76.296			0.741		21.481	0.741							0.741		
12	A 96.296	96.296	9	96.296															2.963	0.741			
13	W 55.556	55.556	5	7.407				2.222	2.963		9.63		5.926	0.741	0.741				8.148	2.963	3.704	55.556	
14	V 92.593	92.593	9	0.741	0.741						0.741			0.741					3.704	0.741	92.593		
15	I 48.889	48.889	7	0.741				0.741			48.889		31.111	1.481							17.037		
16	A 54.815	54.815	9	54.815											2.963				41.481	0.741			
17	I 54.074	54.074	6	0.741				1.481			54.074		37.037	1.481					0.741	1.481	2.963		
18	L 32.593	32.593	4	2.222				13.333			28.889		32.593	4.444							18.519		
19	I 67.407	67.407	7	0.741				0.741			67.407		14.815	5.926						2.963	7.407		
20	M 37.037	37.037	6	1.481	6.667			6.667			8.148		16.296	37.037					4.444	5.926	13.333		
21	L 65.926	65.926	6	2.222				1.481			21.481		65.926	5.185							3.704		
22	A 50.370	50.37	4	50.37	1.481			2.222	7.407		4.444		17.778	0.741					1.481	9.63	4.444		
23	G 100.000	100	9						100														
24	L 25.926	25.926	1	25.185				1.481	13.333		11.111		25.926	2.222					2.963	5.185	11.852	0.741	
25	L 53.333	53.333	6	2.963			0.741	3.704			18.519		53.333	1.481	1.481		10.37	2.963			3.704	0.741	
26	A 65.185	65.185	6	65.185	0.741				2.222										28.889	1.481	1.481		
27	I 56.296	56.296	5	3.704				4.444	0.741		56.296		18.519	5.926							6.667	2.222	1.481
28	L 21.481	21.481	1	2.222			0.741	14.074	1.481	1.481	2.222	5.185	21.481	6.667	4.444	11.852	2.222	5.926	11.111	3.704	2.222	0.741	2.222
29	K 31.852	31.852	1	1.481		1.481	2.222	1.481	2.222		2.222	31.852	4.444		7.407		16.296	2.963	17.778	5.926	2.222		
30	L 90.370	90.37	8								2.963		90.37	6.667									
31	P 78.519	78.519	6	3.704		0.741	2.963		1.481						1.481	78.519	5.185		3.704	1.481	0.741		
32	V 54.074	54.074	6								40.741		4.444							0.741	54.074		
33	A 31.852	31.852	6	31.852		0.741	19.259						0.741	0.741	4.444		0.741	20	19.259	2.222			
34	Q 66.667	66.667	8	0.741			20.741	1.481		0.741				2.222			66.667	5.185	1.481				0.741
35	Y 93.333	93.333	8					5.926		0.741													93.333
36	P 100.000	100	9													100							
37	T 18.519	18.519	2	5.185		17.037	17.037					7.407	1.481	0.741	8.889		8.148	2.963	11.111	18.519	1.481		
38	I 41.481	41.481	7					0.741		0.741	41.481		11.111	10.37	1.481				1.481	0.741	31.852		
39	A 54.815	54.815	7	54.815		2.963	8.889					0.741			1.481		0.741		5.185	12.593	12.593		
40	P 76.296	76.296	7	2.222				0.741			1.481		0.741		11.852	76.296	0.741		2.963	2.963			
41	P 78.519	78.519	8	7.407						0.741					0.741	78.519			2.963	8.148	1.481		
42	A 23.704	23.704	6	23.704			0.741		0.741		1.481		2.963	0.741	5.926		11.852		14.815	22.222	14.815		
43	V 62.963	62.963	7	0.741							34.815		1.481								62.963		
44	T 28.148	28.148	4	5.926		0.741	4.444		1.481		0.741	0.741	1.481	1.481	5.926		8.889	8.889	19.259	28.148	11.852		
45	I 54.074	54.074	8								54.074		1.481								44.444		
46	S 37.778	37.778	4	4.444		0.741	2.222			0.741		2.222		1.481	4.444		2.963	8.889	37.778	28.889	0.741		4.444
47	A 66.667	66.667	8	66.667				0.741	1.481									0.741		24.444	5.926		
48	T 28.148	28.148	1	12.593		0.741	2.222	1.481	0.741		0.741		0.741	0.741	17.778		1.481	0.741	20.741	28.148	9.63		1.481
49	Y 92.593	92.593	8					5.185			0.741		0.741									0.741	92.593
50	P 82.963	82.963	6	2.222		0.741					2.963		0.741			82.963			2.963	2.963	1.481		2.963
51	G 98.519	98.519	9						98.519						0.741				0.741				
52	A 100.000	100	9	100																			
53	S 51.852	51.852	8			23.704									20	0.741			51.852	3.704			
54	A 77.037	77.037	8	77.037					0.741							17.778			4.444				
55	K 27.407	27.407	3	2.222		11.852	20.741		0.741			27.407			1.481		26.667	1.481	3.704	3.704			
56	T 51.852	51.852	7			1.481	0.741	0.741	0.741		1.481		16.296							51.852	26.667		
57	V 41.481	41.481	7	0.741							27.407		20.741	8.889						0.741	41.481		
58	Q 32.593	32.593	7	14.074		1.481	31.852		2.963		0.741	1.481			5.926		32.593	2.963	3.704		2.222		
59	D 32.593	32.593	5	2.963		32.593	17.037		16.296			2.222			20.741		4.444		2.222	1.481			
60	T 42.963	42.963	7	9.63				15.556					0.741		1.481		0.741		26.667	42.963			2.222
61	V 82.963	82.963	9								17.037										82.963		
62	T 62.222	62.222	8	20							2.963		1.481						1.481	62.222	11.852		
63	Q 51.111	51.111	7	6.667		1.481	0.741		1.481			0.741	0.741		0.741		51.111	2.222	18.519	14.815	0.741		
64	V 40.741	40.741	7	0.741							9.63	2.963	6.667			37.037	1.481	0.741			40.741		

65 I 64.444	64.444	7								64.444		31.852	0.741							2.963		
66 E 92.593	92.593	9	0.741			92.593										4.444			1.481	0.741		
67 Q 68.148	68.148	7	2.222		0.741	14.074					0.741			1.481		68.148	11.111	0.741	0.741			
68 N 25.926	25.926	6	23.704			17.778		0.741			2.222			25.926		17.778	2.222	9.63				
69 M 36.296	36.296	7	1.481							26.667		23.704	36.296			0.741				11.111		
70 N 44.444	44.444	8	15.556								5.926			44.444		0.741		12.593	20	0.741		
71 G 80.000	80	8	1.481			1.481		80								5.926		10.37	0.741			
72 V 31.852	31.852	8	20							25.926		20.741							1.481	31.852		
73 D 57.778	57.778	7			57.778	31.111					6.667			0.741	1.481	0.741		1.481				
74 N 48.148	48.148	4			6.667			34.815	7.407		2.222			48.148			0.741					
75 L 53.333	53.333	8					0.741			18.519		53.333	24.444							2.963		
76 L 34.074	34.074	5	0.741		20.741	1.481				5.185		34.074	26.667			0.741	6.667	1.481	0.741	1.481		
77 Y 95.556	95.556	9					1.481											0.741	2.222			95.556
78 M 60.000	60	7					15.556			11.852		6.667	60						0.741	3.704		1.481
79 S 54.815	54.815	6	2.222		2.963	13.333	2.222			0.741	4.444			2.222		2.963		54.815	12.593	1.481		
80 S 94.815	94.815	9	3.704															94.815	1.481			
81 T 25.926	25.926	4			2.963	2.963		0.741			2.963			9.63		22.222	1.481	24.444	25.926	6.667		
82 S 78.519	78.519	8	12.593	0.741				1.481					0.741	1.481				78.519	1.481	2.963		
83 D 27.481	27.481	5	6.107		27.481	2.29	0.763	4.58					0.763	8.397		3.053	2.29	23.664	14.504	3.817		2.29
84 S 66.038	66.038	7	8.491		3.774			5.66						7.547			0.943	66.038	7.547			
85 D 24.444	24.444	5	8.889		24.444	1.481	2.222	0.741				4.444	1.481	9.63	3.704	10.37		19.259	13.333			
86 G 94.074	94.074	9						94.074						2.963				2.963				
87 S 27.407	27.407	1	4.444		0.741	1.481	1.481		1.481	0.741	5.185	1.481	2.963	11.111		10.37	9.63	27.407	12.593	7.407		1.481
88 A 30.370	30.37	6	30.37	0.741			1.481	2.222		0.741		8.148	11.852					21.481	1.481	17.778		3.704
89 T 37.778	37.778	2	2.222		0.741	5.185	0.741			2.222	2.222	3.704	0.741	3.704		15.556	3.704	15.556	37.778	5.185		0.741
90 I 56.296	56.296	6								56.296		20	0.741						2.222	20.741		
91 T 69.630	69.63	6			0.741	1.481				2.963	1.481		2.963	8.148		2.222		8.889	69.63	1.481		
92 L 42.222	42.222	6	14.074							16.296		42.222								27.407		
93 T 66.667	66.667	7					2.963		2.222		4.444	0.741	0.741	3.704		0.741	3.704	8.148	66.667	1.481	0.741	3.704
94 F 80.741	80.741	9	2.222				80.741			1.481		5.926	9.63									
95 E 31.852	31.852	1	12.593		4.444	31.852			0.741	1.481	20.741	1.481		4.444		6.667	8.148		4.444	2.963		
96 L 25.926	25.926	4	15.556		0.741				0.741	8.148		25.926	0.741	1.481	14.815	10.37		15.556	3.704	1.481		0.741
97 G 77.778	77.778	8			4.444	0.741		77.778	1.481					15.556								
98 T 78.519	78.519	8	3.704			0.741	2.222	0.741		2.963							2.222	1.481	78.519	2.222		5.185
99 D 77.778	77.778	7			77.778	0.741			0.741					19.259				0.741	0.741			
100 P 60.741	60.741	1	14.815			0.741	0.741	0.741		0.741		7.407	0.741		60.741			7.407	2.222	2.963		0.741
101 D 71.852	71.852	7	1.481		71.852	5.926				1.481	1.481	0.741		8.148		1.481		2.222	4.444			0.741
102 I 34.074	34.074	4	2.963		0.741	9.63		2.963		34.074	5.185	11.852	5.185	3.704		8.148	4.444	0.741	7.407	2.222		0.741
103 A 95.556	95.556	9	95.556			0.741						2.963								0.741		
104 Q 63.704	63.704	7	5.185			0.741	2.963	0.741				12.593	3.704			63.704	1.481	0.741	1.481	6.667		
105 V 62.222	62.222	8	9.63							4.444			2.963	1.481		8.148		2.963	8.148	62.222		
106 Q 54.815	54.815	7	0.741		19.259	11.852					1.481	2.963	0.741	6.667		54.815	0.741		0.741			
107 V 82.222	82.222	8								11.111		4.444							2.222	82.222		
108 Q 77.778	77.778	8	0.741									8.148	3.704	2.963		77.778	2.222	1.481	2.963			
109 N 77.778	77.778	9	14.074		2.222									77.778				5.926				
110 K 58.519	58.519	8	0.741								58.519					8.889	31.111	0.741				
111 LV 44.444	44.444	8	2.222							7.407		44.444						0.741	0.741	44.444		
112 Q 39.259	39.259	6	11.852		0.741	1.481	0.741	1.481			11.111			17.037		39.259		13.333	2.963			
113 L 27.407	27.407	4	11.111			3.704		1.481		1.481	2.222	27.407				25.185	7.407	15.556	1.481	2.963		
114 A 62.222	62.222	7	62.222							5.926						1.481			3.704	26.667		
115 M 25.185	25.185	4	0.741			20				1.481	2.963	9.63	25.185	1.481		2.963	16.296	1.481	17.037	0.741		
116 P 42.222	42.222	4	15.556		1.481		0.741	2.963			0.741			5.185	42.222		7.407	17.778	2.222	0.741		2.963
117 R 30.370	30.37	5	1.481		2.963	2.222	1.481				13.333	25.185	0.741	2.222		13.333	30.37	2.963	2.222	1.481		
118 L 97.778	97.778	9										97.778	1.481		0.741							
119 P 99.259	99.259	9													99.259	0.741						
120 Q 47.407	47.407	4	6.667		1.481	17.778					2.222	1.481		2.222	5.185	47.407	3.704	8.889	2.963			
121 E 54.815	54.815	4	11.852		9.63	54.815		4.444			0.741		0.741	1.481	2.222	1.481	0.741	5.926	3.704	2.222		
122 V 77.037	77.037	9	12.593							4.444								4.444	1.481	77.037		
123 Q 53.333	53.333	7			2.963	11.852				1.481	0.741	1.481		2.222		53.333	17.037		6.667			2.222
124 Q 36.296	36.296	4	2.963		15.556	2.222					2.963	1.481	0.741	5.185	0.741	36.296	22.963	4.444	2.222	2.222		
125 Q 55.556	55.556	7			0.741	0.741				2.963		6.667	1.481	6.667	20.741	55.556	0.741	1.481		1.481		0.741
126 G 78.519	78.519	8	1.481					78.519		0.741								3.704	3.704	11.852		
127 V 46.667	46.667	6								36.296		15.556	1.481							46.667		
128 T 34.815	34.815	3			2.222	3.704	0.741	0.741	0.741	2.222	2.963	0.741	0.741	5.926		11.852	8.148	17.778	34.815	5.926		0.741
129 V 72.593	72.593	8	0.741		0.741					2.222	3.704	2.222	2.222				2.222	4.444	8.889	72.593		
130 E 27.407	27.407	3	4.444		8.889	27.407		1.481			3.704	1.481		4.444		11.852	8.148	14.815	11.111	2.222		
131 K 67.407	67.407	7	5.926								67.407					3.704	1.481	11.111	8.889	0.741	0.741	
132 S 34.074	34.074	5	24.444		0.741		0.741	13.333			4.444	0.741		0.741		4.444	3.704	34.074	10.37	1.481		0.741
133 S 51.111	51.111	6	6.667		8.889	1.481		11.852						3.704		6.667	1.481	51.111	5.185	2.963		

134 S 40.152	40.152	5	7.576		9.091	0.758		2.273		0.758	1.515		0.758	5.303	15.909	1.515	3.788	40.152	9.848	0.758		
135 S 31.852	31.852	5	2.963		13.333		3.704	11.111		0.741		1.481		16.296				31.852	17.778	0.741		
136 F 48.889	48.889	7	15.556				48.889	2.222		6.667		14.074	2.963	5.926	0.741			1.481	0.741			0.741
137 L 63.910	63.91	7	3.008				1.504			8.271		63.91	5.263			0.752		2.256	9.774	5.263		
138 M 56.391	56.391	6	5.263			0.752	0.752		0.752	1.504		27.82	56.391	2.256		3.759				0.752		
139 V 49.630	49.63	7			0.741		1.481	4.444		11.852		4.444	4.444						4.444	49.63		18.519
140 V 37.778	37.778	5	18.519						0.741	23.704		12.593	2.963			0.741			0.741	37.778		2.222
141 G 32.593	32.593	6	26.667					32.593	11.111				0.741	5.926		0.741	0.741	14.815	5.926	0.741		
142 F 49.630	49.63	6					49.63			2.222		35.556	3.704							8.889		
143 V 21.481	21.481	1	1.481		0.741	0.741	4.444		0.741	16.296	1.481	2.222		0.741		2.222	2.963	9.63	19.259	21.481	0.741	14.815
144 S 82.963	82.963	9	2.963		6.667		0.741							5.926				82.963				0.741
145 P 29.907	29.907	1	2.804		14.953	19.626		0.935			5.607				29.907	0.935	0.935	4.673	19.626			
146 D 56.190	56.19	2	0.952		56.19	3.81		2.857			3.81			17.143		1.905	0.952	7.619	4.762			
147 G 44.030	44.03	1	2.239		12.687	8.209		44.03			6.716			9.701	7.463	4.478	0.746	1.493	2.239			
148 S 27.407	27.407	1	2.222		2.222	7.407		2.963		2.222	11.111	0.741		5.185		6.667	18.519	27.407	12.593	0.741		
149 M 34.074	34.074	4	1.481		0.741	0.741	3.704	0.741	4.444	2.963	2.222	22.222	34.074	0.741		1.481	2.222	1.481	2.222	1.481		17.037
150 D 42.222	42.222	3	0.741		42.222	3.704		0.741	0.741		1.481			7.407	0.741	0.741		20.741	20	0.741		
151 Q 22.222	22.222	1	7.407		5.926	5.926	0.741	3.704		3.704	9.63	0.741	5.185	5.926	5.926	22.222	5.185	9.63	6.667	1.481		
152 L 14.074	14.074	1	7.407		9.63	10.37	2.222	1.481		3.704	1.481	14.074	0.741	5.926	5.926	9.63		11.852	9.63	5.185		0.741
153 D 44.444	44.444	7	10.37		44.444	8.148	5.926									14.815	0.741	0.741			0.741	14.074
154 I 53.333	53.333	7								53.333		37.778	2.963						0.741	5.185		
155 S 37.037	37.037	6	25.185		0.741			8.148			0.741			4.444			5.185	37.037	17.778			0.741
156 D 70.370	70.37	9			70.37	0.741			0.741					27.407				0.741				
157 Y 90.370	90.37	8					4.444			2.963											2.222	90.37
158 V 31.111	31.111	6	26.667				0.741			12.593		22.963	5.185						0.741	31.111		
159 A 31.852	31.852	3	31.852		2.222	6.667		8.148		1.481	0.741	4.444	0.741	5.926		0.741	0.741	11.111	9.63	14.815		0.741
160 S 31.111	31.111	7	11.852					0.741		3.704		17.037		1.481		1.481	30.37	31.111	2.222			
161 N 57.778	57.778	6	1.481						4.444	0.741	2.222	0.741		57.778		3.704	2.222	5.185	1.481	17.037		2.963
162 V 45.185	45.185	5								34.074		12.593	8.148							45.185		
163 K 37.037	37.037	5			0.741	2.963				4.444	37.037	6.667	1.481	2.963		30.37	5.926		2.222	5.185		
164 D 59.259	59.259	6			59.259	11.852		0.741						5.185	21.481	1.481						
165 P 33.333	33.333	4	11.111			22.222				1.481	5.926		1.481	0.741	33.333	14.074	4.444	1.481	2.222	1.481		
166 L 60.741	60.741	5					5.926			28.889		60.741	1.481							2.963		
167 S 48.148	48.148	6	18.519			2.963	5.926	1.481			4.444	2.222		0.741		3.704	10.37	48.148	0.741	0.741		
168 R 77.778	77.778	9	5.185					0.741			0.741					2.222	77.778	2.963	10.37			
169 V 34.074	34.074	4	1.481							25.185		25.926						0.741	12.593	34.074		
170 P 21.481	21.481	1	5.185		14.815	12.593	0.741	2.963			5.926			13.333	21.481	3.704	0.741	14.074	3.704			0.741
171 G 100.000	100	9						100														
172 V 89.630	89.63	9	0.741							6.667			2.222						0.741	89.63		
173 G 76.296	76.296	7	12.593					76.296						0.741		4.444		5.185	0.741			
174 D 34.815	34.815	2	3.704		34.815	14.074					15.556		0.741	5.185		7.407	6.667	10.37	1.481			
175 V 54.815	54.815	7	18.519				3.704			14.815		2.963							5.185	54.815		
176 Q 58.519	58.519	5	0.741		11.852	8.889		2.222				2.222	0.741	2.963		58.519	4.444	3.704	2.222	1.481		
177 L 43.704	43.704	4	6.667				1.481			16.296		43.704	0.741	1.481	1.481	2.222		1.481	2.222	21.481		0.741
178 F 69.630	69.63	7	0.741			0.741	69.63	0.741		1.481		11.111	1.481								2.222	11.852
179 G 97.778	97.778	9	0.741					97.778										1.481				
180 S 33.582	33.582	4	21.642		0.746			14.925			2.985	0.746	0.746	1.493	0.746	9.701	7.463	33.582	5.224			
181 Q 37.037	37.037	6			14.815	21.481		0.741			4.444			1.481	4.444	37.037	1.481	1.481	10.37	2.222		
182 Y 72.593	72.593	7	2.222				11.852		0.741		2.222	2.222			0.741	3.704	3.704					72.593
183 A 78.519	78.519	8	78.519					1.481										19.259	0.741			
184 M 87.407	87.407	9								0.741		10.37	87.407							0.741		0.741
185 R 97.778	97.778	9												2.222			97.778					
186 I 68.889	68.889	7	1.481							68.889		5.185								24.444		
187 W 98.519	98.519	9					0.741												0.741		98.519	
188 L 81.481	81.481	8								2.222		81.481	7.407		0.741					8.148		
189 D 74.815	74.815	7			74.815						2.963			16.296		1.481	3.704	0.741				
190 P 91.111	91.111	7	4.444					0.741							91.111			2.222	1.481			
191 N 20.741	20.741	1	13.333		20	12.593		0.741	4.444	0.741	2.963	2.222	0.741	20.741	0.741	8.148	2.222	2.222	5.185	2.222		0.741
192 K 68.889	68.889	6	3.704			2.963			0.741		68.889	4.444				2.222	15.556	0.741		0.741		
193 L 63.704	63.704	7								2.222		63.704	25.185						1.481	7.407		
194 A 40.741	40.741	4	40.741		0.741	1.481	0.741	0.741	1.481	2.222	1.481	2.222	0.741	27.407		4.444	1.481	2.222	6.667	5.185		
195 S 25.926	25.926	5	25.185		2.222	0.741		11.111		0.741	9.63			16.296		5.926	0.741	25.926	1.481			
196 Y 37.778	37.778	2					26.667		2.963			13.333	0.741	0.741		2.222	13.333			2.222		37.778
197 N 29.630	29.63	3	3.704		4.444	2.963		25.185	0.741		5.185			29.63		22.222	0.741	5.185				
198 L 74.815	74.815	6					0.741			5.185		74.815	9.63							9.63		
199 T 71.852	71.852	8									0.741		7.407	1.481				17.778	71.852	0.741		
200 P 40.000	40	4	28.148				1.481	1.481		3.704			3.704		40			2.963	11.111	7.407		
201 S 22.222	22.222	1	15.556		3.704	1.481		5.926			4.444	4.444	2.222	5.185	0.741	5.185		22.222	13.333	15.556		
202 D 81.481	81.481	7	1.481		81.481	9.63								0.741		5.926			0.741			

203 V 82.222	82.222	8								14.074		3.704								82.222		
204 V 20.000	20	1	11.852			2.222		1.481		14.815	2.222	6.667	2.963	4.444		5.185	5.926	8.148	13.333	20		0.741
205 N 22.222	22.222	1	18.519		7.407	2.963		2.222	0.741	0.741	0.741		0.741	22.222		10.37	3.704	17.037	12.593			
206 A 89.630	89.63	9	89.63							0.741	0.741					3.704			1.481	3.704		
207 I 56.296	56.296	7								56.296		28.889	2.222							12.593		
208 R 29.630	29.63	1	7.407			12.593		1.481		0.741	14.815	0.741		2.963		14.074	29.63	6.667	7.407	0.741		0.741
209 A 34.815	34.815	3	34.815		0.741	19.259		1.481		0.741	0.741			5.185		7.407	2.963	15.556	2.963	7.407		0.741
210 Q 75.556	75.556	9				1.481			1.481					18.519		75.556		0.741				2.222
211 N 98.519	98.519	9												98.519			0.741	0.741				
212 V 24.444	24.444	4	21.481		2.222		4.444			5.185	0.741	1.481	0.741	9.63		2.963	3.704	3.704	11.111	24.444		8.148
213 Q 79.259	79.259	8	0.741		0.741	5.185				2.963		7.407		0.741		79.259	0.741	1.481				0.741
214 V 45.185	45.185	6	10.37				5.926	0.741		23.704		2.963						6.667	3.704	45.185		0.741
215 A 67.407	67.407	7	67.407												5.926			22.963	3.704			
216 A 62.963	62.963	7	62.963				0.741	1.481		1.481		1.481	0.741		8.889			5.926	2.222	14.074		
217 G 100.000	100	9						100														
218 Q 46.667	46.667	4	4.444		0.741	8.148			0.741	0.741	8.148		0.741	3.704		46.667	7.407	14.074	3.704			0.741
219 L 57.037	57.037	6	2.963		2.222		2.963			13.333		57.037	0.741						8.889	11.111		0.741
220 G 76.296	76.296	7	1.481			1.481		76.296			11.111			3.704		0.741	2.963		1.481	0.741		
221 G 44.340	44.34	5	31.132		9.434	0.943		44.34				0.943		0.943		4.717		6.604	0.943			
222 T 28.302	28.302	1	10.377		1.887	8.491						17.925	3.774		9.434	7.547	1.887	8.491	28.302	0.943		0.943
223 P 99.057	99.057	9						0.943							99.057							
224 A 34.906	34.906	2	34.906		0.943		0.943	2.83	0.943	2.83		1.887	1.887	1.887	14.151	0.943		22.642	7.547	4.717		0.943
225 V 38.462	38.462	1	2.885		1.923	0.962	0.962	2.885		7.692	0.962	10.577	0.962	0.962	17.308		3.846	6.731	2.885	38.462		
226 P 23.301	23.301	1	7.767		13.592	6.796		4.854		0.971	19.417	0.971		4.854	23.301	2.913	1.942	9.709	1.942	0.971		
227 G 59.701	59.701	1	5.97		7.463	2.985		59.701			5.224			5.97	1.493	0.746	1.493	5.97	2.985			
228 Q 63.704	63.704	6	2.963		0.741	4.444	0.741			2.222	2.222			0.741	1.481	63.704		9.63	3.704	2.222		5.185
229 Q 33.582	33.582	3	11.194		5.224	10.448	5.97	2.239	0.746		2.239	1.493	0.746		1.493	33.582	7.463	4.478	0.746	1.493		10.448
230 L 46.269	46.269	5					17.91			9.701		46.269		0.746		2.985	1.493		3.731	14.925		2.239
231 N 40.299	40.299	6	1.493		1.493	2.985				1.493	0.746	7.463		40.299		14.179		4.478	20.896	4.478		
232 A 48.889	48.889	5	48.889				3.704			5.926		11.111	0.741					1.481	4.444	11.111		12.593
233 T 38.519	38.519	6	0.741		2.963				0.741		1.481	3.704		9.63	7.407		1.481	31.852	38.519			1.481
234 I 54.074	54.074	6	0.741					1.481		54.074		14.815		2.963				1.481		24.444		
235 N 26.667	26.667	5	1.481			1.481			0.741	20.741	6.667	0.741	1.481	26.667		3.704	2.222	8.148	19.259	5.926		0.741
236 A 54.815	54.815	6	54.815					6.667		0.741		0.741			0.741			5.185	8.889	22.222		
237 Q 48.889	48.889	5	0.741		5.926	6.667		0.741	0.741		16.296	2.963	2.222	5.926	2.222	48.889	3.704	2.222	0.741			
238 T 42.963	42.963	8			0.741	0.741		34.074						0.741				20.741	42.963			
239 R 56.296	56.296	7			10.37	2.222	0.741				2.222	7.407				14.815	56.296	0.741	4.444			0.741
240 L 86.667	86.667	8					2.222			2.222		86.667	2.222						2.963	3.704		
241 Q 19.259	19.259	2	1.481		1.481	7.407		2.963	2.963		13.333			11.111	0.741	19.259	0.741	14.815	18.519	5.185		
242 T 45.926	45.926	4			11.111	2.963		0.741			0.741			7.407			1.481	29.63	45.926			
243 P 40.000	40	1	16.296			8.889		0.741		0.741	1.481	2.963	0.741		40		0.741		7.407	20		
244 E 68.148	68.148	4	1.481		11.852	68.148					2.963					11.852	0.741	1.481	1.481			
245 E 57.037	57.037	5	7.407		5.185	57.037										30.37						
246 F 92.593	92.593	9					92.593					5.926								0.741		0.741
247 G 27.407	27.407	1	11.852			23.704		27.407			6.667	0.741		2.963		3.704	17.037	5.926				
248 N 34.815	34.815	2	8.148		19.259	6.667		0.741	1.481		14.074			34.815		9.63	2.222	2.963				
249 I 71.111	71.111	8								71.111		15.556	2.222						1.481	9.63		
250 I 34.815	34.815	7					0.741			34.815		21.481	0.741		2.963	0.741		0.741	12.593	25.185		
251 L 57.037	57.037	6								24.444		57.037	0.741							17.778		
252 K 47.407	47.407	6	2.963						0.741		47.407						44.444	3.704				0.741
253 V 34.074	34.074	5	19.259			2.222		0.741		0.741				5.185		1.481	0.741	20	14.074	34.074		1.481
254 N 41.667	41.667	2	3.788		15.152	3.788		14.394		0.758	0.758	1.515	0.758	41.667		3.03	4.545	4.545	3.788	1.515		
255 Q 17.778	17.778	1	12.593		5.926	5.926	0.741	8.148	0.741		2.222	0.741		6.667	12.593	17.778	2.222	9.63	13.333	0.741		
256 D 68.468	68.468	4	0.901		68.468	2.703		0.901	1.802				0.901	10.811		3.604		9.009	0.901			
257 G 88.889	88.889	7	2.222		5.926			88.889	0.741		0.741			0.741					0.741			
258 S 48.148	48.148	5	20		2.222	7.407		4.444	0.741		3.704			1.481		6.667	2.222	48.148	2.963			
259 QV 17.778	17.778	2	2.963			5.926	1.481			13.333	3.704	14.074	2.963	2.222	0.741	17.778	8.889	2.222	5.926	17.778		
260 V 68.889	68.889	7								17.037		5.185							8.889	68.889		
261 R 69.630	69.63	6					2.222		4.444		5.185	7.407				0.741	69.63		4.444	3.704		2.222
262 L 91.111	91.111	8								5.185		91.111	0.741							2.963		
263 K 31.111	31.111	1	4.444		0.741			22.963			31.111			2.222		4.444	23.704	9.63		0.741		
264 D 96.296	96.296	9			96.296				0.741					1.481		0.741		0.741				
265 V 76.296	76.296	8								21.481		2.222								76.296		
266 A 91.111	91.111	9	91.111					8.889														
267 R 55.556	55.556	3			5.926	5.926			2.222	1.481	13.333		0.741	2.963		1.481	55.556		8.889	0.741	0.741	
268 V 68.889	68.889	7	2.963							22.963		3.704	0.741		0.741					68.889		
269 E 81.481	81.481	7	5.185		0.741	81.481		4.444								2.222		2.963	0.741	2.222		
270 L 77.778	77.778	6					1.481			5.185	0.741	77.778	11.111				1.481	0.741		1.481		
271 G 87.407	87.407	7	2.963		2.222	3.704		87.407						2.222				1.481				

272 A 40.000	40	4	40	0.741	0.741		17.037			8.889	0.741	0.741	2.222	1.481	2.963	0.741	21.481	1.481			0.741
273 E 54.815	54.815	5	3.704	5.185	54.815		2.963			2.963			2.963		14.815	2.222	8.889	0.741			0.741
274 S 31.111	31.111	3	3.704	12.593	2.963	0.741	1.481	2.963		6.667			28.889		4.444	0.741	31.111	3.704			
275 Y 85.926	85.926	8		7.407		2.222											1.481	2.963			85.926
276 D 28.889	28.889	1	15.556	28.889	2.963		8.889		0.741	0.741	0.741		12.593	0.741	6.667		13.333	7.407	0.741		
277 V 16.296	16.296	1	5.185		0.741	14.074	0.741		9.63		14.815	3.704	0.741		0.741		12.593	10.37	16.296	0.741	9.63
278 R 18.519	18.519	1	3.704	2.963	4.444		2.963		15.556	0.741	9.63		5.185	0.741	2.963	18.519	15.556	5.926	11.111		
279 A 40.000	40	5	40				5.185		1.481		1.481	0.741	2.963	0.741			27.407	11.852	8.148		
280 R 39.259	39.259	1	2.222		3.704	0.741			2.222	9.63	12.593	1.481	2.222		8.889	39.259	5.185	7.407	0.741	0.741	2.963
281 L 30.370	30.37	5	6.667		5.185	22.963			6.667		30.37	2.963			0.741				1.481		22.963
282 N 81.481	81.481	8		11.852			2.222			0.741			81.481		0.741	0.741	2.222				
283 G 82.963	82.963	7		1.481			82.963			0.741			9.63		1.481	0.741	2.963				
284 K 41.481	41.481	1	1.481	2.222	2.963	0.741	1.481	8.889	0.741	41.481	0.741	3.704	2.963		21.481	3.704	1.481	2.963	2.222		0.741
285 P 62.222	62.222	1	5.185	7.407	8.148					2.963			2.963	62.222	1.481	0.741	4.444	3.704	0.741		
286 A 79.259	79.259	8	79.259				2.222										8.889	9.63			
287 V 31.111	31.111	6	24.444				1.481		4.444		0.741	0.741					20	17.037	31.111		
288 G 48.889	48.889	5	18.519		0.741	9.63	48.889	1.481	1.481		2.222	0.741		1.481			4.444		5.926		4.444
289 L 43.704	43.704	4	6.667			2.963	2.222		25.926		43.704	12.593							5.926		
290 G 42.963	42.963	5	36.296				42.963		0.741		0.741			8.148	2.222		5.185	0.741	2.963		
291 I 62.222	62.222	7							62.222		4.444	1.481						0.741	31.111		
292 K 32.593	32.593	4		1.481	4.444	16.296			0.741	32.593	1.481	5.185	6.667		11.111	4.444	2.222	3.704		2.222	7.407
293 L 57.778	57.778	6	4.444				0.741		0.741	0.741	57.778	2.222	0.741	11.111	17.778		1.481	0.741	1.481		
294 A 54.815	54.815	5	54.815				0.741				7.407	0.741	0.741		5.185	7.407	12.593	9.63	0.741		
295 P 52.593	52.593	6	3.704										2.222	52.593	0.741		14.815	25.926			
296 G 74.074	74.074	5	1.481	4.444	1.481		74.074						2.963				7.407	8.148			
297 A 87.407	87.407	9	87.407				4.444										7.407	0.741			
298 N 100.000	100	9											100								
299 A 71.111	71.111	9	71.111		7.407						1.481			16.296			2.222	1.481			
300 L 68.889	68.889	7							15.556		68.889	6.667						0.741	8.148		
301 D 37.778	37.778	1	11.111	37.778	20.741				0.741	0.741			7.407		7.407	3.704	2.963	5.185	2.222		
302 T 51.852	51.852	7	3.704						12.593		3.704						1.481	51.852	26.667		
303 A 62.222	62.222	7	62.222	2.222			2.222		5.185	0.741		2.222				1.481	17.778	0.741	5.185		
304 E 22.963	22.963	1	10.37	15.556	22.963		0.741	1.481		18.519			9.63		3.704	0.741	7.407	8.889			
305 A 43.704	43.704	2	43.704	4.444	8.148		8.148	0.741		2.963	14.074		5.926		5.926	5.926					
306 V 72.593	72.593	8	0.741						22.222		4.444								72.593		
307 R 46.667	46.667	3	0.741	1.481	0.741		1.481	1.481	2.963	30.37	5.185	2.222	3.704			46.667					2.963
308 A 42.222	42.222	1	42.222	7.407	14.815		1.481	0.741		10.37			4.444	2.963	5.185	1.481	2.963	4.444	1.481		
309 K 22.963	22.963	2	7.407	0.741	17.037	0.741			2.222	22.963	9.63	1.481	0.741			20	0.741	12.593	3.704		
310 L 47.407	47.407	5	1.481			1.481			10.37		47.407	28.889							7.407	0.741	2.222
311 A 40.741	40.741	1	40.741	14.815	12.593		1.481		0.741	8.889	0.741		3.704	10.37	1.481		1.481	2.963			
312 E 46.667	46.667	1	2.963	9.63	46.667		3.704		0.741	9.63	0.741		2.222		5.926	10.37	4.444	2.222	0.741		
313 L 66.667	66.667	6	1.481			2.963			12.593		66.667	13.333			1.481		0.741				0.741
314 E 26.667	26.667	1	14.074		26.667		0.741			16.296			1.481		17.037	4.444	16.296	2.222	0.741		
315 P 29.630	29.63	1	6.667	1.481	5.926		1.481	2.222		21.481	1.481		0.741	29.63	9.63	8.148	6.667	4.444			
316 F 25.185	25.185	1	0.741	5.926	2.222	25.185	2.222	1.481			0.741	0.741	11.852		8.148	5.926	9.63	8.889	1.481		14.815
317 F 66.667	66.667	7				66.667					21.481	8.148	0.741							0.741	2.222
318 P 100.000	100	9												100							
319 A 17.037	17.037	1	17.037	8.889	14.074			10.37		2.222			1.481	12.593	14.815	0.741	11.852	5.926			
320 G 77.037	77.037	5	1.481	8.889	0.741		77.037			0.741			2.963				6.667	1.481			
321 L 37.037	37.037	3				0.741			8.148		37.037	30.37							19.259		4.444
322 K 31.111	31.111	1	3.704	8.148	26.667		0.741	1.481		31.111			5.926		5.185	1.481	8.148	6.667			0.741
323 Y 29.630	29.63	3	11.111	0.741		1.481	4.444	0.741	9.63		1.481	3.704	0.741				3.704	0.741	25.926	5.926	29.63
324 V 29.630	29.63	1	5.185	6.667	8.148	2.222	0.741	1.481		13.333	2.222		5.185		2.963	2.222	16.296	2.963	29.63		0.741
325 I 33.333	33.333	4	3.704			5.185		0.741	33.333	1.481	7.407	0.741		0.741			0.741	2.963	18.519		24.444
326 P 51.852	51.852	5	22.963				1.481		0.741	0.741	5.926	1.481	1.481	51.852			1.481		11.852		
327 Y 81.481	81.481	7			1.481	11.852			0.741	2.963		0.741							0.741		81.481
328 D 85.926	85.926	8		85.926	10.37								2.963					0.741			
329 T 62.222	62.222	7	9.63				0.741		0.741	0.741			0.741	11.852	1.481	1.481	10.37	62.222			
330 T 77.778	77.778	9	7.407														14.815	77.778			
331 P 39.259	39.259	1	5.185	4.444	6.667				7.407	2.963	4.444			39.259	2.222	8.148	4.444	4.444	10.37		
332 F 81.481	81.481	8	6.667			81.481						0.741		0.741			0.741		2.963		6.667
333 V 66.667	66.667	8							30.37		0.741	1.481						0.741	66.667		
334 K 41.481	41.481	3	1.481	6.667	7.407			0.741		41.481			7.407		6.667	18.519	6.667	2.963			
335 I 29.630	29.63	4	22.963	5.926	6.667		6.667		29.63	0.741	6.667		0.741		0.741	2.222	7.407	0.741	8.889		
336 S 91.852	91.852	9	6.667														91.852	1.481			
337 I 91.852	91.852	9							91.852		4.444								3.704		
338 E 30.370	30.37	1	0.741	8.889	30.37	0.741	2.222	17.037	1.481	9.63		0.741	5.926		6.667	4.444	6.667	2.963			1.481
339 E 54.074	54.074	6	11.111	8.889	54.074		4.444			11.111			0.741		4.444		4.444	0.741			
340 V 95.556	95.556	9					0.741		2.963									0.741	95.556		

341 V 58.519	58.519	6	3.704				3.704	0.741		23.704		5.926	0.741				0.741			58.519		2.222
342 K 41.481	41.481	1	0.741		0.741	2.963		0.741	19.259	1.481	41.481	1.481	0.741	0.741		9.63	5.185	4.444	2.963	3.704		3.704
343 T 93.333	93.333	9	2.222															2.963	93.333	1.481		
344 L 85.185	85.185	8					2.222			11.111		85.185	0.741							0.741		
345 L 22.963	22.963	1	6.667				21.481	9.63		19.259		22.963						0.741	2.222	16.296		0.741
346 E 91.852	91.852	8			0.741	91.852				2.222		2.222				2.222				0.741		
347 A 94.074	94.074	9	94.074																5.926			
348 I 46.667	46.667	5	2.963				0.741	1.481		46.667		10.37	11.852					0.741	3.704	21.481		
349 V 30.370	30.37	1	11.852				3.704	2.222		21.481		28.148	2.222							30.37		
350 L 79.259	79.259	9	0.741	0.741						17.778		79.259								1.481		
351 V 99.259	99.259	9								0.741										99.259		
352 F 50.370	50.37	7					50.37	0.741		9.63		10.37						0.741	0.741	27.407		
353 L 55.556	55.556	3	8.148	1.481			2.222	0.741		11.111		55.556	0.741							20		
354 V 94.074	94.074	9								5.185		0.741								94.074		
355 M 57.037	57.037	9								16.296			57.037						2.963	23.704		
356 Y 45.185	45.185	4	0.741				28.889			9.63		5.185	0.741					0.741	2.222	5.926	0.741	45.185
357 L 88.148	88.148	7	0.741							2.963		88.148	2.222							5.926		
358 F 96.296	96.296	9		2.963			96.296					0.741										
359 L 96.296	96.296	9								2.222		96.296	1.481									
360 Q 74.815	74.815	9	0.741					21.481	1.481							74.815	1.481					
361 N 60.741	60.741	8	2.222		0.741		0.741		1.481		0.741			60.741			0.741	21.481	11.111			
362 F 37.037	37.037	1	0.741				37.037			9.63		17.778	3.704					0.741		6.667	23.704	
363 R 98.519	98.519	9									0.741			0.741			98.519					
364 A 74.815	74.815	8	74.815	0.741														11.111	2.963	0.741		9.63
365 T 65.926	65.926	9	2.963							2.222			0.741					14.074	65.926	14.074		
366 L 68.148	68.148	6	1.481				1.481			22.222		68.148	0.741					1.481		4.444		
367 I 94.074	94.074	9								94.074										5.926		
368 P 96.296	96.296	9	0.741												96.296				1.481	1.481		
369 T 45.185	45.185	7	13.333	0.741						9.63		14.815	0.741					2.963	45.185	12.593		
370 I 57.778	57.778	5	2.963	0.741						57.778		20.741	3.704						1.481	12.593		
371 A 66.667	66.667	8	66.667																20	13.333		
372 V 65.185	65.185	9	5.185							29.63										65.185		
373 P 100.000	100	9													100							
374 V 74.815	74.815	9								11.852		13.333								74.815		
375 S 43.704	43.704	9	8.889	3.704														43.704	0.741	42.963		
376 L 85.926	85.926	8								6.667		85.926	4.444							2.963		
377 L 54.074	54.074	8					0.741			28.889		54.074	5.926							10.37		
378 G 97.778	97.778	9	2.222					97.778														
379 T 76.296	76.296	8	8.148					2.222										1.481	76.296	11.852		
380 F 76.296	76.296	7	4.444	2.963			76.296	0.741		3.704		7.407	1.481							2.963		
381 A 50.370	50.37	5	50.37	0.741			11.852	20		0.741		2.222	5.185					4.444	3.704	0.741		
382 V 50.370	50.37	4	5.185				7.407	5.926		17.778		11.111	0.741						1.481	50.37		
383 L 48.889	48.889	8								3.704		48.889	47.407									
384 A 21.481	21.481	2	21.481			1.481	0.741	1.481	5.926		2.222	17.037		1.481		14.815		12.593	0.741	0.741	1.481	17.778
385 A 42.963	42.963	1	42.963				0.741	0.741		7.407		24.444	11.111		0.741			0.741	1.481	8.889		0.741
386 F 51.852	51.852	4	7.407	2.963			51.852			2.222		23.704	9.63					0.741	0.741	0.741		
387 G 97.037	97.037	9			2.222			97.037						0.741								
388 F 63.704	63.704	7					63.704					1.481	0.741							1.481		32.593
389 S 91.111	91.111	9																91.111	8.889			
390 I 68.889	68.889	7	2.222							68.889		18.519	0.741							8.889	0.741	
391 N 100.000	100	9												100								
392 T 52.593	52.593	7	5.185							1.481		22.222	4.444			1.481		1.481	52.593	10.37		0.741
393 L 86.667	86.667	8								2.963		86.667	8.889							1.481		
394 T 85.185	85.185	9												0.741				14.074	85.185			
395 M 49.630	49.63	9								0.741		48.889	49.63							0.741		
396 F 77.037	77.037	9	0.741				77.037					21.481										0.741
397 AG 48.889	48.889	7	48.889					48.889										2.222				
398 M 68.889	68.889	8		0.741			0.741			7.407		21.481	68.889							0.741		
399 V 92.593	92.593	9								7.407										92.593		
400 L 100.000	100	9										100										
401 A 88.148	88.148	9	88.148	0.741														9.63		1.481		
402 I 94.815	94.815	9								94.815		1.481								3.704		
403 G 98.519	98.519	9						98.519											1.481			
404 L 62.963	62.963	9								35.556		62.963	0.741							0.741		
405 LV 47.407	47.407	9								5.185		47.407								47.407		
406 V 100.000	100	9																		100		
407 D 100.000	100	9			100																	
408 D 98.519	98.519	9			98.519									1.481								
409 A 99.259	99.259	9	99.259															0.741				

410 I 100.000	100	9								100												
411 V 92.593	92.593	9								6.667		0.741								92.593		
412 V 93.333	93.333	9								2.222			4.444							93.333		
413 V 85.926	85.926	9								4.444		8.148	1.481							85.926		
414 E 100.000	100	9				100																
415 N 97.778	97.778	9						1.481	0.741					97.778								
416 V 88.148	88.148	9	0.741	0.741						9.63			0.741							88.148		
417 E 79.259	79.259	8	0.741		5.926	79.259			8.889		0.741					0.741			0.741		0.741	2.222
418 R 97.778	97.778	9														0.741	97.778		1.481			
419 V 29.630	29.63	7							22.963	21.481		11.852		13.333					0.741	29.63		
420 M 61.481	61.481	8								31.111		5.926	61.481							1.481		
421 A 30.769	30.769	1	30.769			3.846		1.282	5.128				3.846			3.846	6.41	25.641	10.256	8.974		
422 E 73.563	73.563	4			3.448	73.563	1.149	1.149	1.149		4.598			1.149		5.747	3.448	1.149	3.448			
423 E 68.889	68.889	5	2.963		9.63	68.889		2.222			6.667					5.185	2.222	1.481		0.741		
424 G 77.037	77.037	5	2.222		2.222			77.037	2.222		8.148			2.963		2.222	1.481	1.481				
425 L 72.593	72.593	6		0.741		9.63				2.222	5.926	72.593	4.444			1.481	1.481		0.741	0.741		
426 S 44.444	44.444	4	2.963		4.444	2.222		1.481	0.741		3.704			2.222	25.185			44.444	12.593			
427 P 87.313	87.313	6	5.224					0.746		2.239	0.746				87.313		2.239			1.493		
428 K 33.582	33.582	2	1.493				14.925		0.746	2.239	33.582	11.94	1.493				22.388		0.746	7.463		2.985
429 E 54.815	54.815	2	8.889		14.074	54.815			0.741	0.741	2.222			1.481	1.481	8.148	5.926	0.741	0.741			
430 A 98.519	98.519	9	98.519															1.481				
431 T 65.185	65.185	9	31.852															1.481	65.185	1.481		
432 R 31.852	31.852	1	0.741			5.185			2.963	20.741	11.111	9.63	1.481			5.185	31.852	3.704	1.481	4.444		1.481
433 K 57.037	57.037	5	0.741			3.704			0.741	9.63	57.037	5.185		1.481		9.63	8.889		0.741	2.222		
434 S 43.704	43.704	9	34.074					20										43.704	2.222			
435 M 77.037	77.037	9	12.593	0.741								0.741	77.037					2.222	5.926	0.741		
436 R 28.889	28.889	1	2.222		8.148	4.444		26.667	0.741		11.111			0.741		5.926	28.889	7.407	3.704			
437 E 50.370	50.37	8				50.37			0.741							48.148	0.741					
438 I 68.148	68.148	8								68.148		6.667	1.481							23.704		
439 T 28.889	28.889	7	17.778				0.741	2.222		1.481	0.741	0.741				26.667		19.259	28.889	1.481		
440 G 75.556	75.556	5					3.704	75.556			0.741	1.481	1.481	0.741	0.741		0.741	5.926	2.222	5.926	0.741	
441 A 57.778	57.778	8	57.778							0.741					40					1.481		
442 L 42.963	42.963	8								29.63		42.963								27.407		
443 V 54.074	54.074	8								43.704		1.481	0.741							54.074		
444 G 53.333	53.333	8	35.556					53.333										9.63	1.481			
445 I 63.704	63.704	9								63.704			22.963					2.222	8.889	2.222		
446 A 48.148	48.148	8	48.148					3.704										2.222	42.963	2.963		
447 L 55.556	55.556	5	2.222				0.741			11.111		55.556	19.259					2.222	2.963	5.926		
448 V 74.815	74.815	9	2.963							0.741									21.481	74.815		
449 L 98.519	98.519	9								0.741		98.519	0.741									
450 S 42.963	42.963	6	21.481	8.889				1.481		4.444		3.704	2.222					42.963	5.185	9.63		
451 A 94.815	94.815	9	94.815															5.185				
452 V 100.000	100	9																		100		
453 F 77.778	77.778	9					77.778					1.481										20.741
454 V 34.074	34.074	4	14.074							29.63		17.037						2.963	2.222	34.074		
455 P 100.000	100	9													100							
456 M 57.778	57.778	8	2.222							20		3.704	57.778					2.222	6.667	7.407		
457 A 82.222	82.222	9	82.222					16.296										0.741		0.741		
458 F 80.741	80.741	8	0.741				80.741			0.741		14.815	2.963									
459 F 41.481	41.481	7	3.704				41.481	1.481		6.667		14.815	23.704			4.444		0.741	0.741	2.222		
460 G 52.593	52.593	5	0.741		0.741	2.222		52.593							8.148	3.704		28.148	3.704			
461 G 99.259	99.259	9						99.259						0.741								
462 S 42.963	42.963	7	5.185				0.741			11.111		24.444	1.481			0.741		42.963	7.407	5.926		
463 T 75.556	75.556	8	3.704									0.741				1.481		3.704	75.556	14.815		
464 G 97.778	97.778	9	2.222					97.778														
465 A 32.593	32.593	5	32.593		0.741	8.148		0.741		3.704	0.741			2.222		13.333	1.481	7.407	4.444	24.444		
466 I 57.037	57.037	9					13.333			57.037		26.667	2.222							0.741		
467 Y 77.778	77.778	9					22.222															77.778
468 R 57.778	57.778	6	0.741							0.741	26.667					10.37	57.778		2.963	0.741		
469 Q 79.259	79.259	9				20										79.259		0.741				
470 F 100.000	100	9					100															
471 S 50.370	50.37	9	44.444	0.741				0.741										50.37	3.704			
472 I 34.074	34.074	5	1.481				9.63			34.074		32.593	1.481						0.741	20		
473 T 88.889	88.889	9	2.222															7.407	88.889	1.481		
474 I 60.741	60.741	9								60.741		30.37	7.407							1.481		
475 A 48.889	48.889	8	48.889	0.741						2.963								5.185	2.222	40		
476 S 34.074	34.074	5	11.852				2.222	17.037		14.815		0.741	0.741					34.074	5.185	13.333		
477 A 61.481	61.481	8	61.481															37.037	1.481			
478 M 45.185	45.185	9	0.741							11.111			45.185						13.333	29.63		

479 V 29.630	29.63	4	21.481				8.148	3.704		16.296		12.593	0.741					2.222	5.185	29.63		
480 L 47.407	47.407	8					11.852			34.815		47.407								5.926		
481 S 100.000	100	9																100				
482 V 40.741	40.741	8	27.407					25.926				0.741						4.444	0.741	40.741		
483 L 38.519	38.519	5					18.519			28.148		38.519	2.222							12.593		
484 V 60.000	60	8	1.481							7.407		14.074		17.037						60		
485 A 85.926	85.926	9	85.926															14.074				
486 L 85.185	85.185	8								4.444		85.185	10.37									
487 T 58.519	58.519	8		0.741				0.741		28.148		0.741						5.926	58.519	5.185		
488 L 88.889	88.889	8					9.63			0.741		88.889	0.741									
489 T 57.778	57.778	9	0.741								0.741							40.741	57.778			
490 P 100.000	100	9													100							
491 A 77.037	77.037	9	77.037							0.741		0.741	17.778						1.481	2.222		
492 L 77.778	77.778	9								0.741		77.778	20.741			0.741						
493 C 73.333	73.333	8	11.852	73.333				0.741										14.074				
494 A 74.074	74.074	7	74.074					2.222								0.741	0.741	17.037	0.741	4.444		
495 T 48.148	48.148	5	3.704					0.741		2.222	11.111	14.815	1.481	0.741			5.185	3.704	48.148	6.667		1.481
496 L 61.481	61.481	4					5.926			8.148		61.481	20							3.704	0.741	
497 L 97.037	97.037	9								1.481		97.037	0.741					0.741				
498 K 77.778	77.778	6			0.741			1.481	0.741		77.778	0.741		1.481	0.741	1.481	10.37	2.963	1.481			
499 P 58.955	58.955	1	10.448	0.746	2.239	4.478		2.985	5.224		2.985			0.746	58.955	2.985	2.239	3.731	2.239			
500 H 18.797	18.797	1	6.015		1.504	6.015		9.023	18.797	18.045	1.504	7.519		6.015	2.256	1.504	3.759	3.008	3.008	12.03		
501 A 15.625	15.625	1	15.625		14.844	7.031		7.031	14.062	1.562	9.375			1.562	8.594	4.688		9.375	5.469	0.781		
502 K 36.082	36.082	1	21.649		2.062	8.247		6.186	6.186	1.031	36.082		1.031	2.062	5.155	1.031	1.031	4.124	1.031	3.093		
503 G 55.128	55.128	1	1.282		11.538	11.538		55.128	3.846		3.846		1.282	2.564		2.564	1.282	1.282	3.846			
504 E 20.779	20.779	1	7.792		15.584	20.779		10.39	16.883	1.299	11.688	1.299	1.299	1.299	2.597	1.299		6.494		1.299		
505 H 38.889	38.889	2			3.968	6.349	0.794	2.381	38.889		13.492	0.794		6.349	11.111	7.143	1.587	4.762	1.587			0.794
506 H 22.689	22.689	1	1.681		1.681	5.882	0.84	16.807	22.689		15.966			5.042	17.647	1.681		6.723	3.361			
507 E 19.512	19.512	1	6.504		13.008	19.512		14.634	1.626	3.252	8.943	0.813		8.13	0.813	2.439	2.439	9.756	1.626	4.065	2.439	
508 L 20.000	20	1	15		2.5		7.5	12.5	2.5	7.5	10	20		5	7.5				2.5	2.5	5	
509 K 42.500	42.5	1			1.25	2.5		1.25	1.25	7.5	42.5	8.75	3.75		1.25	12.5	8.75	1.25	5	2.5		
510 K 23.333	23.333	1	3.333		13.333	1.111		8.889	1.111		23.333			6.667	1.111		16.667	3.333	20		1.111	
511 G 74.444	74.444	4	1.111			1.111		74.444			6.667			1.111	2.222		8.889	4.444				
512 F 65.649	65.649	5					65.649	0.763		3.817		16.031	3.817		1.527					3.817	3.053	1.527
513 F 69.403	69.403	7	6.716			7.463	69.403	1.493				5.97		0.746				2.985	2.985	0.746		1.493
514 G 45.185	45.185	1	10.37			1.481		45.185	2.963	2.963	3.704	0.741		2.222	0.741	4.444	17.778	3.704	1.481	1.481	0.741	
515 W 46.667	46.667	1	6.667	1.481			1.481	8.889		3.704	2.963	2.963		0.741	11.111	0.741	5.185	0.741	4.444	2.222	46.667	
516 F 77.612	77.612	8	2.239				77.612			4.478		6.716						0.746		8.209		
517 N 74.815	74.815	7	2.222		10.37	10.37		0.741	1.481					74.815								
518 R 62.963	62.963	3	2.963			1.481		0.741	5.185		7.407	0.741	0.741	3.704		2.963	62.963	2.222	5.926		2.222	0.741
519 GL 14.074	14.074	1	5.185				11.852	14.074	0.741	4.444	2.963	14.074	12.593	2.963	1.481		5.926	4.444	10.37	7.407		1.481
520 F 78.519	78.519	8					78.519			2.222		11.111	0.741							1.481		5.926
521 D 34.815	34.815	1	4.444		34.815	28.148		3.704	1.481		1.481	1.481		14.074		0.741	0.741	4.444	4.444			
522 R 40.741	40.741	1	2.963					4.444	3.704		28.148			1.481	0.741	3.704	40.741	8.148	3.704	0.741	1.481	
523 S 26.667	26.667	3	9.63				4.444	2.963		5.185		22.963	8.148	5.926				26.667	5.926	6.667	1.481	
524 T 38.519	38.519	5	13.333	0.741		1.481		0.741			5.926			4.444	0.741	3.704	4.444	19.259	38.519	5.926	0.741	
525 H 20.741	20.741	1	3.704		7.407	11.111		8.889	20.741		3.704	2.222		14.074		8.148	11.111	4.444	2.963	0.741		0.741
526 R 27.407	27.407	1	5.926	0.741			2.222	25.926	18.519		6.667	1.481				0.741	27.407	3.704	1.481	2.222	0.741	2.222
527 Y 96.296	96.296	9	0.741				1.481					0.741							0.741			96.296
528 T 20.000	20	1	3.704		0.741	17.037	0.741	8.148	0.741	0.741	2.222	1.481	0.741	0.741		18.519	2.963	10.37	20	11.111		
529 R 24.444	24.444	1	5.926		11.852	2.222		12.593	1.481	0.741	11.111	2.963		11.111		2.222	24.444	6.667	1.481		0.741	4.444
530 G 22.222	22.222	1	7.407	0.741		0.741	3.704	22.222	0.741	4.444	1.481	11.111	6.667			1.481	6.667	20	5.185	5.926	1.481	
531 V 63.704	63.704	8	0.741	0.741		0.741				6.667		23.704		2.222					1.481	63.704		
532 G 29.630	29.63	1	19.259		5.926	2.963		29.63	6.667		2.963	1.481		2.963		2.222	11.111	11.111	2.963	0.741		
533 K 18.519	18.519	1	4.444			1.481	8.148	13.333	7.407		18.519	2.963	0.741	10.37		2.222	17.037	5.926	0.741	2.222	0.741	3.704
534 I 34.815	34.815	3	7.407				1.481			34.815		18.519	8.148					3.704	5.926	19.259	0.741	
535 L 61.481	61.481	5					2.963			13.333		61.481	6.667	1.481				0.741	1.481	11.852		
536 R 35.556	35.556	1	5.926		2.963	2.222		10.37	8.889		12.593	0.741	0.741	6.667		4.444	35.556	3.704	4.444			0.741
537 R 34.815	34.815	1	2.222		0.741		2.222	7.407	11.111		11.111	0.741	2.222	4.444		2.222	34.815	14.815	2.963	1.481	0.741	0.741
538 RS 24.444	24.444	4	3.704				0.741	6.667			8.148	2.222			5.926	2.222	24.444	24.444	20.741	0.741		
539 G 37.778	37.778	1	5.185			0.741	2.963	37.778	0.741	2.963	3.704	10.37	1.481		8.148		3.704	1.481	2.963	10.37	6.667	0.741
540 R 45.926	45.926	3	6.667	0.741			1.481	0.741		8.889	0.741	11.852	1.481		4.444	1.481	45.926	3.704		11.852		
541 Y 27.407	27.407	3	7.407				7.407	2.963		4.444		0.741	12.593					2.963	3.704	20.741	9.63	27.407
542 L 44.444	44.444	4		0.741			5.185	6.667		8.889		44.444	20.741					1.481		8.148	0.741	2.963
543 L 37.037	37.037	1	9.63				3.704	6.667		7.407		37.037	4.444					0.741	2.222	28.148		
544 I 31.111	31.111	2	8.148				13.333	1.481		31.111		20.741	1.481						1.481	21.481		0.741
545 Y 60.741	60.741	6	15.556				11.111	2.222		0.741		2.222							0.741	3.704	2.963	60.741
546 L 31.852	31.852	1	20.741	0.741			2.963	11.852		5.926		31.852	1.481						0.741	23.704		
547 L 31.111	31.111	1	14.074				1.481	15.556		14.815		31.111						0.741	1.481	20.741		

548 L 37.778	37.778	4	2.963				2.963			34.074		37.778	4.444					0.741	0.741	16.296		
549 V 36.296	36.296	3	4.444	5.185			6.667	0.741		8.889		28.148	3.704					2.963	2.963	36.296		
550 V 30.370	30.37	1	20.741	2.963			0.741	28.148		7.407		7.407	0.741					1.481		30.37		
551 G 34.074	34.074	1	12.593	0.741				34.074		5.185		20	2.222					9.63	2.222	13.333		
552 M 31.111	31.111	4	5.926	5.185				3.704		4.444		23.704	31.111					0.741	19.259	5.926		
553 A 24.444	24.444	1	24.444	1.481			4.444	20.741		5.185		2.222			11.111				0.741	18.519	3.704	7.407
554 F 22.963	22.963	1	2.963				22.963	3.704	0.741	5.926	1.481	19.259	0.741	0.741	2.222				0.741	17.778	11.111	9.63
555 L 57.778	57.778	2	5.185			0.741	3.704	11.111		4.444		57.778	10.37			1.481	0.741	2.222		2.222		
556 F 65.926	65.926	5	2.963				65.926			0.741		8.148	2.222					1.481	0.741	0.741	0.741	16.296
557 L 17.778	17.778	1	2.222		0.741	0.741	1.481	1.481	1.481	6.667	8.889	17.778	12.593	3.704		5.185	1.481	11.111	11.852	8.889	2.963	0.741
558 R 44.444	44.444	1	4.444			0.741	4.444	0.741	1.481	0.741	6.667	4.444	2.222	3.704		6.667	44.444	12.593	3.704		0.741	2.222
559 L 51.111	51.111	6	0.741							11.111		51.111	6.667		1.481			6.667	11.111	10.37	0.741	
560 P 85.185	85.185	6	2.222						0.741		3.704			0.741	85.185	2.963		3.704	0.741			
561 T 35.556	35.556	2	3.704		0.741	2.222		13.333	0.741		8.148		0.741	2.222	1.481	1.481	2.222	25.926	35.556	1.481		
562 S 38.519	38.519	6	18.519		0.741	20.741		20.741		0.741								38.519				
563 F 75.556	75.556	8					75.556					21.481							1.481	0.741		0.741
564 L 55.556	55.556	8	16.296			0.741				8.889		55.556	0.741						2.222	15.556		
565 P 99.259	99.259	9													99.259		0.741					
566 DE 20.741	20.741	1	7.407		20.741	20.741		1.481		0.741	2.222	5.926	1.481	6.667	3.704	9.63	0.741	2.222	14.074	2.222		
567 E 86.667	86.667	9			0.741	86.667										12.593						
568 D 100.000	100	9			100																	
569 Q 68.889	68.889	8								0.741	17.778	1.481	0.741			68.889	9.63	0.741				
570 G 84.444	84.444	8	2.963					84.444				2.963				7.407		0.741				1.481
571 V 28.889	28.889	3	5.185		2.222	0.741	9.63	0.741		12.593		1.481	5.185				0.741	0.741	4.444	28.889	0.741	26.667
572 L 34.815	34.815	4	2.963				30.37			8.148		34.815	5.185					0.741	1.481	14.815		1.481
573 M 23.704	23.704	5	0.741		0.741		20.741	0.741		12.593		22.222	23.704	0.741		0.741			4.444	11.852		0.741
574 T 34.074	34.074	4	17.037				1.481	7.407		2.963		1.481	2.963	2.963				8.148	34.074	20.741		0.741
575 M 25.926	25.926	5	7.407		5.185	0.741	6.667	2.963		10.37		5.185	25.926	1.481		16.296	0.741	13.333	1.481	1.481		0.741
576 V 43.704	43.704	4	21.481				2.222	4.444		16.296		1.481	1.481					2.963	1.481	43.704		4.444
577 Q 60.741	60.741	5	0.741		0.741	2.222	0.741			2.222	7.407	2.963	2.222	4.444	0.741	60.741	1.481	3.704	9.63			
578 L 69.630	69.63	7	12.593					3.704				69.63	4.444	0.741	0.741	0.741		3.704	3.704			
579 P 94.815	94.815	8	1.481		0.741										94.815	0.741		1.481		0.741		
580 A 30.370	30.37	4	30.37		14.074	6.667								2.222	11.852	6.667		17.037	4.444	6.667		
581 G 62.222	62.222	6	10.37		3.704	0.741		62.222	2.222					8.889	2.222			3.704	4.444			1.481
582 A 67.407	67.407	7	67.407			0.741												25.185	2.963	3.704		
583 T 53.333	53.333	8	1.481		0.741			0.741						10.37	0.741			32.593	53.333			
584 L 35.556	35.556	3	7.407		1.481	2.222	0.741		0.741	5.926	4.444	35.556	3.704	0.741		24.444	3.704	3.704	0.741	2.963		1.481
585 E 36.296	36.296	2	7.407		25.926	36.296		2.963		0.741			0.741	4.444		10.37		5.926	3.704	1.481		
586 R 62.963	62.963	7				1.481			0.741				0.741	0.741		14.815	62.963	1.481	0.741			16.296
587 T 77.037	77.037	8	2.963		0.741				0.741	5.185	0.741	4.444	0.741	0.741				2.963	77.037	3.704		
588 Q 24.444	24.444	1	3.704		13.333	19.259		2.222			2.222	14.074	2.963	6.667		24.444	3.704	3.704		2.963		0.741
589 A 26.667	26.667	1	26.667		7.407	10.37	0.741	2.222	3.704	1.481	23.704	1.481		5.926		7.407	3.704	0.741	1.481	2.963		
590 V 65.926	65.926	6	4.444				3.704	0.741	1.481	1.481	0.741	1.481	1.481	2.222		0.741		1.481	6.667	65.926		7.407
591 L 25.185	25.185	3	6.667				1.481	4.444		14.074		25.185	11.852	2.222			1.481	2.963	8.148	21.481		
592 D 25.926	25.926	1	9.63		25.926	17.037		2.222	1.481	0.741	14.074	0.741		5.926		7.407	11.111	3.704				
593 Q 27.407	27.407	1	8.148		4.444	21.481			0.741	0.741	22.222				2.963	27.407	5.926	0.741	3.704	1.481		
594 V 35.556	35.556	3	6.667				8.889			11.111		10.37	20.741						1.481	35.556		5.185
595 E 32.593	32.593	2	3.704		4.444	32.593		1.481		2.963	0.741		0.741	8.889		3.704	8.148	12.593	17.778	1.481		0.741
596 D 22.963	22.963	1	9.63		22.963	11.111		2.963	6.667		11.852			11.852	1.481	13.333	4.444	2.963	0.741			
597 Y 39.259	39.259	2	2.222		0.741	1.481	3.704		8.889	20.741	2.963	2.963	2.963	2.222		1.481	0.741	0.741	2.963	4.444	1.481	39.259
598 F 23.704	23.704	2	11.852				23.704	2.963		8.148		20	2.222	1.481					0.741	5.926		22.963
599 L 47.407	47.407	1	8.889		3.704	3.704	3.704	2.222			7.407	47.407	10.37	1.481		2.222	1.481	3.704	2.963	0.741		
600 T 18.519	18.519	1	6.667		5.185	16.296		1.481	0.741		11.111			14.815		7.407	2.222	14.815	18.519	0.741		
601 Q 18.182	18.182	1	3.306		8.264	9.917	6.612		9.091	5.785	10.744	3.306		12.397	0.826	18.182			7.438	4.132		
602 E 94.545	94.545	9				94.545										5.455						
603 K 63.768	63.768	3	5.797			2.899		5.797	1.449	1.449	63.768			1.449		2.899	11.594	2.899				
604 P 39.552	39.552	1	11.94		26.866	9.701		1.493	1.493		2.239			3.731	39.552	1.493		1.493				
605 N 28.148	28.148	4	16.296		0.741	20		22.963				2.222		28.148	0.741		0.741	2.222	3.704	2.222		
606 V 68.148	68.148	7	0.741					1.481		12.593	0.741	1.481	2.222	0.741			1.481	0.741	5.185	68.148		4.444
607 E 28.148	28.148	1	19.259		9.63	28.148		3.704	0.741		8.889	2.963	2.963	4.444		2.963	2.222	4.444	4.444	2.963		2.222
608 S 51.111	51.111	4	3.704		11.111	2.222	4.444	5.185	3.704		0.741	0.741		8.889			0.741	51.111	3.704	0.741		2.963
609 V 44.444	44.444	5	7.407		0.741		1.481	0.741		11.852		1.481	2.963	6.667			0.741	8.889	10.37	44.444		2.222
610 F 49.630	49.63	5	0.741				49.63			11.111		7.407	5.185	0.741		3.704			4.444	11.852	2.963	2.222
611 T 33.333	33.333	5	23.704							6.667	0.741	2.222	2.222			8.889		16.296	33.333	5.926		
612 V 44.444	44.444	6	0.741				17.778			22.963		4.444	0.741			0.741			7.407	44.444		0.741
613 N 22.222	22.222	3	13.333		1.481			0.741		4.444		8.148	0.741	22.222	11.852	2.222		17.778	9.63	7.407		
614 G 93.333	93.333	8			0.741	0.741	2.222	93.333				0.741	1.481					0.741				
615 F 59.848	59.848	6	3.788		0.758		59.848	1.515		3.03		12.121				0.758		7.576	3.788	1.515	1.515	3.788
616 S 42.308	42.308	6	0.769		6.154	0.769	0.769	27.692					0.769	13.846	4.615			42.308	1.538		0.769	

617 F 52.344	52.344	5	3.125				52.344	9.375		8.594		5.469	0.781	3.125	8.594			2.344	1.562	4.688		
618 S 26.357	26.357	4	22.481		2.326		0.775	7.752		2.326	0.775	11.628	0.775	13.178	2.326			26.357	3.101	5.426		0.775
619 G 72.222	72.222	7	6.481					72.222						0.926		0.926		1.852	12.037	5.556		
620 N 29.630	29.63	5	6.481		0.926			1.852		0.926		2.778	1.852	29.63		14.815	18.519	19.444	0.926	0.926		0.926
621 G 67.290	67.29	6	5.607					67.29		0.935	1.869	1.869				1.869	1.869	14.019	3.738	0.935		
622 Q 56.410	56.41	7	1.709		1.709	1.709		0.855						5.128	21.368	56.41		7.692	3.419			
623 N 84.252	84.252	8	3.937		0.787			0.787						84.252				8.661	0.787	0.787		
624 A 29.323	29.323	3	29.323				0.752	7.519					6.015	3.759		5.263		21.805	14.286	11.278		
625 G 60.902	60.902	6	27.82		0.752			60.902				2.256	0.752		0.752			4.511		2.256		
626 I 25.373	25.373	4	1.493				13.433	0.746		25.373		18.657	18.657	0.746		0.746	7.463	2.239	4.478	5.97		
627 A 42.424	42.424	4	42.424				0.758	17.424		6.061		5.303	5.303	1.515				6.061	1.515	13.636		
628 F 74.074	74.074	8	0.741				74.074	11.852	0.741	8.148							0.741	0.741		1.481		1.481
629 V 41.481	41.481	5	7.407				6.667			21.481		3.704	5.185		2.963				11.111	41.481		
630 S 20.741	20.741	2	4.444			0.741		2.222		5.926	6.667	4.444	8.889	8.148	6.667		11.111	20.741	14.815	5.185		
631 L 87.407	87.407	8					5.926					87.407	4.444			0.741				1.481		
632 K 77.037	77.037	6	4.444		3.704	1.481					77.037	0.741		2.963	0.741		2.222	2.222	2.222	2.222		
633 D 49.630	49.63	4	1.481		49.63	0.741		1.481	1.481					4.444	35.556	0.741		2.963	0.741	0.741		
634 W 81.481	81.481	8	0.741				15.556		0.741			0.741			0.741						81.481	
635 S 35.556	35.556	2	8.889		22.222	14.815		5.926			5.926			4.444		2.222		35.556				
636 E 60.000	60	3	2.963		10.37	60		0.741			3.704	1.481		0.741		17.037		0.741	0.741	1.481		
637 R 100.000	100	9															100					
638 E 15.556	15.556	1	0.741		11.852	15.556		2.963	2.963		14.074	0.741		5.185	13.333	5.185	2.963	9.63	13.333	0.741		0.741
639 G 34.615	34.615	1	8.462		7.692	6.154	0.769	34.615	0.769	1.538	5.385	0.769			2.308	2.308	16.154	6.923	3.846	0.769		1.538
640 S 24.444	24.444	1	12.593		9.63	11.852		1.481	0.741	0.741	7.407	0.741	2.222	1.481	14.815	1.481	2.963	24.444	7.407			
641 E 24.603	24.603	1	9.524		10.317	24.603		7.937	0.794	1.587	3.175			1.587	1.587	15.079	1.587	7.937	11.111	3.175		
642 N 29.921	29.921	2	9.449		5.512	2.362	3.15	5.512		0.787	3.15	11.811	4.724	29.921		13.386	0.787	7.874	0.787	0.787		
643 S 26.446	26.446	2	4.959		8.264	12.397		7.438	1.653	0.826	13.223			1.653	0.826	9.091		26.446	11.57	1.653		
644 A 38.000	38	5	38		1				1	4		7	1					12		34	1	1
645 F 16.346	16.346	1	15.385		4.808	13.462	16.346	6.731		0.962	0.962	0.962	2.885	7.692	6.731	10.577	1.923	4.808	3.846	1.923		
646 A 61.905	61.905	4	61.905		4.762	5.714	0.952	2.857			1.905			1.905	1.905	5.714		10.476	1.905			
647 I 57.031	57.031	5	1.562			7.031	2.344			57.031		13.281	0.781				0.781			17.188		
648 A 31.298	31.298	3	31.298			0.763				19.847	0.763	8.397	1.527	3.053		5.344		3.053	9.16	16.031		0.763
649 A 25.000	25	1	25		4.545	12.121		20.455	2.273	0.758	5.303	0.758	3.03	3.03	8.333	9.848	0.758	2.273	1.515			
650 R 53.030	53.03	3	9.091		4.545	9.848			1.515	1.515	6.061	3.788		1.515	0.758	4.545	53.03	0.758	2.273	0.758		
651 A 48.507	48.507	5	48.507				1.493	1.493		11.194		26.119	2.985					0.746	0.746	5.97		0.746
652 N 26.119	26.119	4	2.985		0.746		1.493	2.239		0.746	0.746		17.164	26.119		22.388	1.493	4.478	17.164			2.239
653 G 23.485	23.485	1	21.97		0.758	2.273		23.485			11.364	0.758	3.788	2.273	3.788	17.424	6.061	1.515	2.273	2.273		
654 A 54.217	54.217	2	54.217			2.41	3.614		8.434	1.205	1.205	1.205	6.024			2.41	6.024	2.41	1.205	2.41		7.229
655 F 38.843	38.843	2	2.479		0.826	4.132	38.843	1.653		1.653	21.488	10.744	0.826	1.653		4.959	6.612	3.306				0.826
656 S 22.901	22.901	3	10.687				16.031	2.29		5.344		16.794	8.397	5.344		0.763	1.527	22.901	1.527	3.817		4.58
657 Q 26.667	26.667	1	9.63		8.148	5.926		9.63	2.222	1.481	8.148	0.741		3.704		26.667	2.963	11.852	8.148	0.741		
658 I 66.667	66.667	6					3.704			66.667	0.741	8.889	3.704	1.481	0.741			2.222	0.741	9.63		1.481
659 K 28.148	28.148	1	6.667		2.963	2.963		2.963	0.741		28.148	0.741		4.444	18.519	8.889	14.074	3.704	2.222	2.963		
660 D 39.259	39.259	4	2.222		39.259	36.296		17.037			0.741			0.741		1.481		2.222				
661 A 54.074	54.074	5	54.074				0.741	23.704		2.222		6.667	2.963			0.741	0.741	2.963		4.444		0.741
662 FM 15.556	15.556	2	3.704		2.222	2.222	15.556			2.963	3.704	8.148	15.556	6.667		14.815	6.667	6.667	5.185	0.741		5.185
663 V 51.111	51.111	4	10.37					1.481		23.704		2.963	2.222	1.481	0.741			1.481	4.444	51.111		
664 F 48.889	48.889	4	10.37				48.889	2.222	0.741	7.407		2.963	11.111	0.741				2.963	4.444	3.704		4.444
665 A 50.370	50.37	5	50.37					2.222		6.667					11.852			13.333	2.963	12.593		
666 F 55.556	55.556	5	0.741				55.556			6.667		7.407	7.407					6.667	1.481	12.593		1.481
667 N 31.111	31.111	3	7.407			0.741		1.481		1.481		5.185	6.667	31.111	15.556	7.407		11.111	4.444	7.407		
668 P 60.150	60.15	4	0.752				3.759			3.008		23.308	5.263		60.15		1.504	0.752		1.504		
669 P 93.333	93.333	8	4.444												93.333		0.741	1.481				
670 A 45.926	45.926	6	45.926		0.741	5.926		0.741							27.407			15.556	3.704			
671 I 57.778	57.778	8	0.741				0.741			57.778		17.037	0.741							22.963		
672 P 28.148	28.148	1	0.741		11.111	2.963		0.741		5.185		5.926	3.704	2.222	28.148	10.37	3.704	7.407	5.185	12.593		
673 G 57.037	57.037	7	0.741		2.963	31.852		57.037						1.481		0.741		2.222	2.963			
674 L 71.852	71.852	7	2.963				0.741	8.148		2.222		71.852	4.444					6.667	2.222			0.741
675 G 88.372	88.372	7			0.775	3.101		88.372					0.775	0.775				4.651	0.775	0.775		
676 T 36.567	36.567	4	0.746		1.493	2.985		10.448		0.746		1.493	0.746	17.164		10.448	1.493	12.687	36.567	2.985		
677 A 32.836	32.836	6	32.836				2.239	20.149		5.224		0.746	1.493	1.493		2.239		24.627	8.955			
678 S 23.134	23.134	5	7.463		1.493		2.985	21.642				11.194	1.493	6.716	3.731			23.134	20.149			
679 G 78.519	78.519	8	0.741			1.481		78.519						1.481	17.037	0.741						
680 F 74.815	74.815	7			0.741		74.815		0.741	3.704		3.704	0.741							11.852		3.704
681 D 36.296	36.296	5	2.963		36.296	13.333		0.741		0.741	5.926			4.444		12.593	5.185	8.889	8.148	0.741		
682 F 35.780	35.78	3	1.835		0.917		35.78	1.835		0.917		34.862	17.431							4.587		1.835
683 E 28.302	28.302	1	0.943			28.302	13.208		0.943		0.943		2.83	0.943		23.585	12.264			0.943	4.717	10.377
684 L 80.189	80.189	8								14.151		80.189								5.66		
685 Q 45.185	45.185	7				11.852	13.333			15.556	2.963	5.185	5.185			45.185				0.741		

686 D 68.148	68.148	8	6.667		68.148		0.741			0.741		1.481		2.963						19.259		
687 R 35.556	35.556	4	0.741			1.481	0.741		5.185	12.593	3.704	8.889		5.185		17.037	35.556	2.963	2.222	2.222		1.481
688 G 31.111	31.111	1	30.37			2.222		31.111				2.963	0.741	3.704			0.741	16.296	11.111	0.741		
689 G 51.111	51.111	2	9.63		4.444			51.111		0.741				20.741	5.926			0.741	6.667			
690 L 37.037	37.037	1	11.852		2.222	0.741	0.741	6.667		0.741	2.963	37.037	2.222	4.444		11.111	8.148	0.741	4.444	5.926		
691 G 67.407	67.407	5			12.593	2.963		67.407	0.741					2.963	2.222	1.481		6.667	2.963			
692 H 41.481	41.481	5	2.222			1.481	7.407		41.481	0.741	0.741	2.222	0.741		5.926	1.481	7.407		2.963	2.222		22.963
693 E 36.296	36.296	1	13.333		18.519	36.296		3.704	0.741		2.963			1.481	3.704	5.185	3.704	4.444	5.185	0.741		
694 A 33.333	33.333	1	33.333		4.444	14.074		2.963			20			0.741		8.889	2.222	5.926	3.704	3.704		
695 L 91.852	91.852	8	0.741				0.741			4.444		91.852	1.481							0.741		
696 T 17.037	17.037	1	11.111		1.481	1.481	5.926			2.963	4.444	16.296	16.296	0.741		5.926	0.741	2.222	17.037	5.185		8.148
697 A 28.889	28.889	1	28.889		6.667	14.074		2.222			7.407	0.741		6.667		25.185	2.963	2.222	2.222	0.741		
698 A 59.259	59.259	6	59.259		0.741	3.704	1.481			2.222		2.963	2.963	0.741	0.741	2.963		1.481	4.444	14.074		2.222
699 R 48.148	48.148	6	20			1.481		1.481		1.481		2.222	5.926			2.963	48.148	2.222	8.148	5.926		
700 N 42.963	42.963	4	3.704		16.296	8.889	0.741	2.222				0.741	0.741	42.963		15.556	0.741	2.963	3.704		0.741	
701 Q 45.926	45.926	3	3.704		3.704	11.111		2.222		2.222	8.889		1.481	5.185		45.926	5.926	0.741	7.407	1.481		
702 L 59.259	59.259	5	1.481				9.63			10.37		59.259	8.148							11.111		
703 L 52.593	52.593	4				0.741	2.963			15.556	5.185	52.593	5.185			4.444		2.963	1.481	8.889		
704 G 34.815	34.815	1	17.778		2.963	7.407	2.963	34.815		0.741	5.185		2.963	3.704		12.593	0.741	1.481	5.185			1.481
705 M 25.185	25.185	2	13.333			9.63				0.741	17.778	13.333	25.185	1.481		5.185	8.148	2.222	2.222	0.741		
706 A 71.852	71.852	7	71.852					1.481		8.148		4.444	3.704					2.963	0.741	6.667		
707 A 35.556	35.556	1	35.556		2.222	1.481	0.741	3.704	1.481		7.407	0.741	5.926	12.593		8.148	8.889	6.667	2.222	0.741	1.481	
708 Q 44.444	44.444	3	7.407		1.481	8.889		2.963	0.741		21.481		0.741	5.185		44.444	0.741	2.222	3.704			
709 S 33.333	33.333	4	1.481		11.111	3.704		0.741	14.815		1.481	0.741		14.074		0.741	6.667	33.333	4.444	2.222		4.444
710 P 60.741	60.741	2			1.481	2.222		19.259	0.741		8.889				60.741	1.481	1.481	3.704				
711 D 16.296	16.296	1	6.667		16.296	13.333		5.926	0.741	5.926	11.852	8.148	2.222	3.704		7.407	6.667	3.704	0.741	6.667		
712 L 29.167	29.167	1							4.167			29.167	25			4.167		4.167	16.667	16.667		
713 L 62.963	62.963	7	0.741				23.704			3.704		62.963	2.963						0.741	5.185		
714 TV 30.263	30.263	1	11.842			1.316			1.316			3.947	2.632			2.632		14.474	30.263	30.263		1.316
715 G 24.444	24.444	1	5.185		2.963	1.481	14.815	24.444			0.741		1.481	8.889	0.741	2.222	17.778	6.667	0.741	2.963		8.889
716 V 63.704	63.704	6	4.444							5.185		9.63	4.444		5.185	0.741		3.704	2.963	63.704		
717 R 48.889	48.889	5	0.741		10.37	0.741	10.37	0.741	1.481	2.963	0.741	0.741	5.185	2.963		2.963	48.889	0.741	2.222	0.741	0.741	6.667
718 P 35.556	35.556	4	7.407			1.481	0.741	1.481	6.667	4.444	1.481	8.148	2.222	2.222	35.556			17.037	8.889	0.741		1.481
719 N 42.963	42.963	7	2.222		21.481	3.704	0.741	2.222				0.741		42.963				14.815	11.111			
720 G 48.148	48.148	5	1.481		1.481		8.148	48.148		0.741		17.778	0.741					2.963	3.704	2.222	1.481	11.111
721 L 40.000	40	4	3.704		5.926	0.741		0.741			11.111	40	5.926	2.222		17.778	8.148	2.222	1.481			
722 E 30.370	30.37	4	13.333		7.407	30.37	8.889	0.741		5.926		0.741		3.704	7.407	0.741	2.222	2.963	1.481	6.667		7.407
723 D 58.519	58.519	7	1.481		58.519	5.185		1.481						20.741	2.963	4.444		5.185				
724 T 23.704	23.704	5	11.852			4.444		5.185		2.222	5.185	1.481	4.444	2.963		2.963	0.741	17.037	23.704	17.778		
725 P 80.000	80	6	6.667		0.741	0.741						0.741	0.741		80	2.963	0.741	2.963	2.222	1.481		
726 Q 70.370	70.37	7			0.741	6.667		0.741	0.741	0.741	1.481	0.741		1.481		70.37	3.704	3.704	5.185	3.704		
727 L 35.556	35.556	3	2.222				15.556			5.926		35.556	6.667					0.741	2.222	12.593		18.519
728 KQ 21.481	21.481	1	2.222		9.63	5.926	0.741		3.704		21.481	1.481		5.926		21.481	15.556	1.481	4.444	2.222		3.704
729 L 37.037	37.037	4	8.889				0.741			31.111		37.037	0.741							20.741	0.741	
730 D 41.481	41.481	1	2.222		41.481	12.593			5.185	3.704	7.407	1.481		5.926	0.741	2.963	2.222	7.407	2.963	2.963		0.741
731 I 69.630	69.63	7					0.741			69.63		5.926								23.704		
732 D 90.370	90.37	8			90.37						0.741			8.889								
733 R 52.593	52.593	7	0.741		5.185		0.741		2.222		5.185	2.222		1.481	0.741	24.444	52.593	0.741	0.741			2.963
734 E 42.963	42.963	2	12.593		12.593	42.963			0.741	0.741	0.741	0.741		5.926		3.704	9.63	1.481	3.704	4.444		
735 K 80.741	80.741	7	2.222							0.741	80.741	2.222				8.148	5.926					
736 A 88.148	88.148	8	88.148							1.481		2.963	0.741							6.667		
737 Q 22.963	22.963	3	14.815			11.852	0.741	10.37		0.741	16.296	2.222	0.741	2.963		22.963	7.407	6.667	1.481	0.741		
738 A 59.259	59.259	5	59.259		5.185	1.481				0.741	0.741	2.963				5.926	1.481	10.37	8.889	2.963		
739 L 61.481	61.481	6	1.481						3.704		0.741	61.481	14.074			10.37		0.741				7.407
740 G 91.111	91.111	7	0.741		1.481			91.111			0.741			1.481		3.704		0.741				
741 V 64.444	64.444	6	0.741							12.593		20.741	1.481							64.444		
742 S 50.370	50.37	4	7.407		4.444	1.481		2.222			0.741			5.185	11.111	1.481	5.185	50.37	10.37			
743 I 28.148	28.148	4	0.741				9.63			28.148		26.667	17.037	1.481	0.741	1.481			0.741	11.852		1.481
744 S 29.630	29.63	3	14.074		13.333	5.185		2.222	0.741		2.222	0.741		6.667		14.815	1.481	29.63	8.889			
745 D 59.259	59.259	3	5.185		59.259	8.148								5.926		9.63	1.481	5.185	5.185			
746 I 65.185	65.185	7	1.481							65.185		8.148								25.185		
747 N 42.222	42.222	6	3.704		0.741		20.741	13.333						42.222			0.741	11.852	2.963	0.741		2.963
748 S 23.704	23.704	1	8.148		16.296	11.111	0.741	4.444	0.741	1.481	0.741			10.37		9.63		23.704	11.852	0.741		
749 T 77.037	77.037	7	6.667		3.704					1.481				0.741		0.741		5.926	77.037	3.704		
750 L 76.296	76.296	7					1.481			10.37		76.296	7.407							4.444		
751 S 35.556	35.556	6	12.593		0.741	1.481		17.037			1.481			0.741		27.407	1.481	35.556	1.481			
752 T 39.259	39.259	3	21.481					1.481		10.37		2.222		1.481				11.111	39.259	11.852		0.741
753 A 51.852	51.852	6	51.852				3.704	1.481				8.889	10.37	2.222		1.481		2.222	3.704			14.074
754 L 43.704	43.704	5					10.37			0.741		43.704	11.111							3.704	28.889	1.481

755 G 84.444	84.444	7	4.444					84.444										11.111				
756 S 48.148	48.148	7	3.704		2.963	5.185		37.037							1.481	0.741		48.148	0.741			
757 S 29.630	29.63	1	10.37		2.222	0.741		8.889	1.481	0.741		13.333	2.963	6.667		2.222	1.481	29.63	12.593	4.444		2.222
758 Y 84.444	84.444	7				2.222	2.222		0.741	0.741	2.222			1.481	0.741		0.741	0.741	2.222	1.481		84.444
759 V 67.407	67.407	6	5.185							21.481		2.963						0.741	1.481	67.407	0.741	
760 N 87.407	87.407	8	0.741		0.741	0.741		2.963						87.407		0.741		2.963	3.704			
761 D 76.296	76.296	8			76.296	0.741						0.741		1.481		0.741	17.037		1.481		0.741	0.741
762 F 88.148	88.148	8					88.148			2.222		1.481							0.741	4.444		2.963
763 I 30.370	30.37	6	1.481		6.667	0.741	1.481	1.481	0.741	30.37		2.222	5.185	21.481	7.407	0.741		3.704	9.63	6.667		
764 D 40.000	40	5	2.963		40	0.741	0.741		1.481	5.185	2.963	11.852	5.185	11.111		4.444	8.148	0.741		1.481		2.963
765 R 34.815	34.815	3	6.667		9.63	7.407	17.778	2.963	1.481		4.444	0.741	0.741	6.667		5.185	34.815					1.481
766 G 93.333	93.333	8						93.333						3.704				2.222				
767 R 88.148	88.148	8					0.741		0.741		5.926					3.704	88.148			0.741		
768 V 45.926	45.926	7	4.444							2.963		4.444	6.667	2.222		1.481		15.556	16.296	45.926		
769 K 43.704	43.704	8					3.704				43.704					10.37	2.222				5.185	34.815
770 K 45.185	45.185	5			2.222	3.704			0.741		45.185			1.481	2.222	18.519	25.185		0.741			
771 V 96.296	96.296	9								2.963		0.741								96.296		
772 Y 40.000	40	5					3.704			28.148		1.481	4.444	7.407		2.222	0.741	0.741	3.704	6.667	0.741	40
773 V 42.222	42.222	5	11.852							10.37		13.333	6.667		12.593			2.222	0.741	42.222		
774 Q 91.791	91.791	9								0.746			5.97			91.791	0.746	0.746				
775 A 52.985	52.985	7	52.985					16.418				2.239	0.746	1.493				11.194		14.925		
776 D 41.791	41.791	3	8.955		41.791	34.328				1.493	4.478	1.493		1.493		2.239	0.746	1.493	0.746	0.746		
777 A 46.269	46.269	5	46.269		2.985	2.239		8.209			0.746	0.746	1.493		5.224	7.463	16.418	3.731	2.239	2.239		
778 P 24.444	24.444	1	3.704		11.852	11.111		3.704	0.741	1.481	14.074	0.741		2.222	24.444	14.815	1.481	5.926	2.222	1.481		
779 Y 31.852	31.852	1	10.37		3.704	3.704	30.37	1.481	1.481		1.481			1.481		4.444		7.407	0.741	0.741	0.741	31.852
780 R 97.037	97.037	9			0.741							2.222					97.037					
781 M 47.407	47.407	6	3.704		5.926	2.222	0.741	0.741	1.481			17.778	47.407	0.741	0.741	2.963		9.63	2.963	2.963		
782 L 25.185	25.185	3	2.222		3.704	4.444		2.963	1.481		2.963	25.185	0.741	15.556		13.333	2.963	13.333	9.63	1.481		
783 P 71.852	71.852	3	5.185			0.741	1.481			3.704	1.481	5.185			71.852	1.481		2.963	0.741	5.185		
784 E 47.407	47.407	1	6.667		22.963	47.407		4.444			2.222			2.222		5.926	0.741	5.926	0.741		0.741	
785 D 63.704	63.704	6	5.185		63.704	0.741			1.481		0.741		0.741	2.222		11.852	0.741	8.148	1.481	0.741	2.222	
786 I 48.889	48.889	6	0.741				2.222			48.889		37.037	0.741							10.37		
787 N 22.963	22.963	1	5.185		8.148	5.185		16.296			5.926	15.556		22.963		6.667	5.185	5.926	0.741	0.741		1.481
788 N 25.185	25.185	1	0.741		8.148	3.704		2.963	5.926		20.741	7.407	0.741	25.185		8.889	8.889	4.444	1.481			0.741
789 W 37.778	37.778	6					3.704		0.741	7.407		33.333	1.481			1.481				2.222	37.778	11.852
790 Y 60.000	60	5			0.741		13.333		3.704		10.37			0.741		2.963	4.444	2.222	1.481			60
791 V 85.185	85.185	8	0.741							5.926		1.481						0.741	5.926	85.185		
792 R 74.074	74.074	6	0.741						0.741		8.889	0.741	0.741	1.481	4.444		74.074	1.481	6.667			
793 N 54.815	54.815	7	8.148					14.815				0.741		54.815				14.815	6.667			
794 AS 18.519	18.519	1	18.519		10.37	2.963		3.704		1.481	7.407	1.481		17.037	3.704	4.444	4.444	18.519	4.444	1.481		
795 N 18.462	18.462	1	11.538		14.615	6.923		2.308	0.769		6.923	2.308	0.769	18.462	0.769	10	6.154	15.385	2.308			0.769
796 G 88.889	88.889	7			2.963	0.741		88.889						5.185	0.741			0.741	0.741			
797 E 30.370	30.37	1	2.222		11.852	30.37		11.852			6.667	0.741		3.704		22.222		3.704	4.444	2.222		
798 M 72.593	72.593	8	2.222									17.037	72.593			0.741	0.741	6.667				
799 V 71.852	71.852	7								18.519		4.444	0.741						4.444	71.852		
800 P 89.630	89.63	6	1.481											0.741	89.63		1.481	6.667				
801 L 50.370	50.37	8					41.481			5.926		50.37	0.741							1.481		
802 S 68.148	68.148	7	7.407		1.481			11.111			1.481			3.704			2.963	68.148	3.704			
803 A 45.185	45.185	6	45.185			5.185								3.704		1.481		23.704	20.741			
804 F 55.556	55.556	5					55.556			3.704		22.963	2.963							14.815		
805 A 36.296	36.296	5	36.296							13.333		3.704						13.333	5.185	28.148		
806 T 34.815	34.815	2	2.222		3.704	3.704			0.741		11.111		0.741	5.185		3.704	5.185	28.889	34.815			
807 S 19.259	19.259	1	10.37		0.741	1.481	1.481	7.407		15.556	0.741	8.889	2.963		4.444	0.741		19.259	9.63	15.556	0.741	
808 R 20.000	20	1	1.481		3.704	17.778		1.481	10.37		7.407			2.963	1.481	8.148	20	11.111	11.111	2.963		
809 W 57.037	57.037	3			6.667	4.444		0.741	1.481	1.481	0.741	4.444		0.741	8.889	4.444	2.963		2.222	2.222	57.037	1.481
810 ET 25.926	25.926	1	1.481		0.741	25.926		1.481		7.407	5.926	0.741		1.481		2.963	3.704	11.852	25.926	9.63		0.741
811 Y 35.556	35.556	2	6.667		0.741	5.185	2.222	0.741		1.481	1.481	3.704	0.741	1.481	0.741	1.481		13.333	14.815	9.63		35.556
812 G 68.889	68.889	6	7.407			8.889		68.889						0.741		5.185	2.222	2.222	1.481	2.963		
813 P 56.296	56.296	7	3.704			0.741				0.741					56.296	1.481		36.296	0.741			
814 P 40.000	40	4	3.704		14.815	6.667		1.481			0.741	2.963		4.444	40	4.444	8.889	4.444	2.963	3.704		0.741
815 R 40.000	40	5	5.185			0.741	1.481			2.222	9.63	2.963	0.741	2.222	2.963	15.556	40	11.852	2.963	1.481		
816 L 69.630	69.63	7	2.222				0.741			6.667		69.63	2.222				2.222	0.741		14.815		0.741
817 E 34.815	34.815	4	2.222		1.481	34.815		1.481		3.704	1.481	1.481	8.889	8.889	6.667	2.963		8.889	11.852	4.444	0.741	
818 R 73.333	73.333	8							15.556			0.741		0.741		7.407	73.333	0.741	1.481			
819 Y 68.148	68.148	7					30.37						1.481									68.148
820 N 80.741	80.741	9	0.741		2.222							0.741	0.741	80.741		12.593		2.222				
821 G 66.667	66.667	8						66.667	0.741	1.481		3.704	0.741	1.481		21.481	0.741	2.222		0.741		
822 L 37.037	37.037	3					15.556			2.222	0.741	37.037	2.963	0.741		5.185	0.741	2.222		6.667		25.926
823 P 42.222	42.222	5	5.926		0.741	1.481				2.222	0.741	2.222		17.778	42.222	2.222	4.444	17.037	2.222	0.741		

824 S 51.852	51.852	7	42.963															51.852	5.185			
825 A 29.630	29.63	5	29.63				2.222	0.741		5.926		8.148	25.926					1.481	0.741	21.481		3.704
826 E 42.963	42.963	4	2.222		8.148	42.963				2.222	8.889			7.407	1.481	2.963	6.667	3.704	11.111	2.222		
827 I 69.630	69.63	7					0.741			69.63		22.222	0.741							6.667		
828 Q 20.000	20	4	2.963		0.741	2.963	0.741	2.963	0.741	2.222		18.519	1.481	8.889		20		18.519	6.667	11.852		0.741
829 G 84.444	84.444	8	13.333					84.444		0.741		0.741								0.741		
830 E 23.704	23.704	2	11.111		8.148	23.704	1.481	5.185		2.963			1.481	3.704		14.815	0.741	11.111	1.481	14.074		
831 A 50.000	50	2	50					3.906		0.781		4.688	2.344	0.781	31.25	0.781		1.562	0.781	3.125		
832 A 74.803	74.803	6	74.803		3.15				0.787			2.362	6.299	0.787			0.787	6.299	3.15	1.575		
833 P 84.211	84.211	4	5.263		0.752	0.752	0.752				0.752				84.211	0.752	0.752	3.008	3.008			
834 G 89.552	89.552	7	0.746		0.746			89.552		2.985			0.746				2.985		2.239			
835 V 29.104	29.104	1	2.239		0.746	0.746	4.478	1.493	3.731	3.731	14.925	2.239	0.746			1.493	2.239	2.239	5.224	29.104		24.627
836 S 84.444	84.444	8	4.444											1.481	2.222	0.741		84.444	6.667			
837 S 43.704	43.704	7	0.741			0.741				0.741	0.741	6.667	8.148	0.741				43.704	37.037	0.741		
838 G 88.148	88.148	7	2.222		5.185	0.741		88.148										2.963	0.741			
839 E 37.037	37.037	3	6.667		16.296	37.037					1.481			2.222		25.185	0.741		10.37			
840 A 93.333	93.333	9	93.333					2.222		1.481								0.741		2.222		
841 M 60.741	60.741	8				2.963				13.333	1.481	11.111	60.741			4.444			0.741	5.185		
842 A 44.444	44.444	1	44.444		17.037	6.667				0.741	3.704	2.222	0.741	6.667		5.185	0.741	4.444	5.185	2.222		
843 A 24.444	24.444	2	24.444			17.778	6.667			3.704	2.222	16.296	3.704			1.481	1.481	0.741	5.926	6.667	5.185	3.704
844 M 51.852	51.852	7	0.741				1.481			14.074		22.222	51.852							9.63		
845 E 74.815	74.815	5	2.963		2.963	74.815		0.741		0.741	2.963			0.741		8.889	0.741	1.481	2.222		0.741	
846 E 17.778	17.778	1	9.63		5.926	17.778		4.444			15.556		1.481	2.963		12.593	17.037	5.926	5.185	1.481		
847 L 53.333	53.333	3	4.444			4.444	1.481		0.741	16.296	1.481	53.333	5.185	0.741		2.222			2.963	6.667		
848 A 58.647	58.647	5	58.647							5.263		10.526	1.504					1.504	1.504	21.053		
849 A 28.571	28.571	1	28.571		6.015	3.759		5.263	0.752		16.541			6.767	3.008	3.759	6.767	15.789	0.752	2.256		
850 K 25.000	25	1	2.273		3.03	12.121		0.758	0.758	6.061	25	3.03		3.788		22.727		4.545	6.061	9.091		0.758
851 L 90.370	90.37	8	2.222			0.741	3.704	0.741				90.37	2.222									
852 P 92.593	92.593	7	0.741		0.741			2.222							92.593	0.741		1.481	1.481			
853 S 15.909	15.909	1	15.152		3.788	7.576		3.788		0.758	5.303	1.515		12.121	11.364	12.121	3.788	15.909	4.545	2.273		
854 G 88.889	88.889	7	2.222		5.185	0.741		88.889	0.741					1.481				0.741				
855 I 31.111	31.111	5	1.481				24.444			31.111		1.481	11.111			0.741		0.741	5.185	5.926		17.778
856 G 42.963	42.963	3	8.889		1.481	1.481		42.963	1.481	0.741	2.963	1.481	1.481	4.444		2.963	1.481	16.296	11.852			
857 Y 42.963	42.963	1					17.778	1.481	5.926	9.63		12.593				0.741		0.741	0.741	5.926	1.481	42.963
858 E 44.444	44.444	6	9.63		33.333	44.444		1.481			0.741			0.741		2.222		6.667	0.741			
859 W 80.000	80	7					5.185					0.741									80	14.074
860 T 65.926	65.926	7	5.926					2.222		1.481	2.222	0.741	2.222	1.481		2.222		15.556	65.926			
861 G 74.815	74.815	7	5.185		8.889	5.926		74.815										5.185				
862 M 30.370	30.37	5	4.444		1.481	14.815				2.963	0.741	27.407	30.37			11.111		1.481	2.222	2.963		
863 S 71.111	71.111	8	16.296							0.741			0.741					71.111	10.37	0.741		
864 Y 57.037	57.037	6	0.741				3.704		0.741		2.963	14.074					19.259	0.741			0.741	57.037
865 Q 86.667	86.667	9	0.741		0.741	11.852										86.667						
866 E 62.222	62.222	7			1.481	62.222	6.667				0.741	10.37				11.852						6.667
867 R 44.444	44.444	4	2.963			0.741			0.741	10.37	14.815	3.704	1.481			4.444	44.444	1.481	2.222	12.593		
868 L 42.222	42.222	3	3.704		1.481	4.444				2.963	3.704	42.222	2.963			22.222	4.444	1.481	6.667	3.704		
869 S 51.852	51.852	7	20.741			17.037		0.741								4.444		51.852	3.704	1.481		
870 G 80.741	80.741	7	6.667			0.741		80.741						0.741		1.481		8.889	0.741			
871 N 45.185	45.185	6	13.333		4.444		0.741	12.593	0.741		1.481			45.185		0.741		20.741				
872 Q 55.556	55.556	7	16.296				0.741	0.741			2.222	2.963				55.556		8.148	13.333			
873 A 62.222	62.222	7	62.222			0.741	2.222	2.963				16.296	5.926					2.963	5.185	1.481		
874 P 45.185	45.185	1	7.407				0.741	2.963		8.889		12.593	3.704		45.185	1.481			4.444	7.407	1.481	3.704
875 A 30.370	30.37	1	30.37				11.852	1.481		8.148		14.074	6.667		2.963	0.741		6.667	1.481	6.667	2.963	5.926
876 L 57.037	57.037	7	10.37							10.37		57.037							11.852	10.37		
877 Y 42.963	42.963	7					41.481			2.222		8.148	3.704							1.481		42.963
878 A 47.407	47.407	3	47.407				4.444	10.37		2.222		10.37	0.741		11.852			1.481	5.185	5.926		
879 L 50.370	50.37	6	4.444				5.185			29.63		50.37	6.667							3.704		
880 S 58.519	58.519	8	28.889	5.185				3.704										58.519	0.741	2.963		
881 L 51.852	51.852	5	2.222				1.481	0.741		20		51.852	4.444					0.741		18.519		
882 L 54.074	54.074	5	5.185	0.741						23.704		54.074	1.481							14.815		
883 V 48.889	48.889	7	1.481				14.074			14.074		14.815	6.667							48.889		
884 V 66.667	66.667	9	9.63							23.704										66.667		
885 F 83.704	83.704	8					83.704														0.741	15.556
886 L 97.037	97.037	9	0.741									97.037	2.222									
887 C 44.444	44.444	8	1.481	44.444			5.926			6.667		2.222						0.741		38.519		
888 L 100.000	100	9										100										
889 A 89.630	89.63	9	89.63	0.741														2.963		6.667		
890 A 91.852	91.852	9	91.852					0.741		5.185								0.741	0.741	0.741		
891 L 59.259	59.259	9										59.259	0.741			40						
892 Y 82.222	82.222	9					17.778															82.222

893 E 94.074	94.074	9				94.074		0.741						5.185								
894 S 94.815	94.815	9									0.741			1.481			2.963	94.815				
895 W 77.778	77.778	7	0.741				11.852			2.963		5.926								0.741	77.778	
896 S 60.741	60.741	7	2.963				0.741	1.481		0.741	0.741	2.222					17.037	60.741	10.37	2.963		
897 I 42.963	42.963	8			21.481					42.963		19.259							0.741	15.556		
898 P 99.259	99.259	9													99.259			0.741				
899 F 47.407	47.407	5	2.222	0.741			47.407			2.963		37.037	2.222					0.741	2.222	4.444		
900 S 42.222	42.222	7	33.333					1.481		8.889								42.222	0.741	13.333		
901 V 75.556	75.556	9								23.704			0.741							75.556		
902 M 45.926	45.926	7	2.963							22.222		27.407	45.926							1.481		
903 L 72.593	72.593	6	2.222				0.741	1.481		5.185		72.593	6.667		0.741			0.741	2.222	7.407		
904 V 51.852	51.852	8	8.889	0.741						16.296								14.815	7.407	51.852		
905 V 88.148	88.148	9	2.963							7.407		1.481								88.148		
906 P 97.778	97.778	9										0.741			97.778					1.481		
907 L 69.630	69.63	8					6.667			2.963		69.63	18.519							2.222		
908 G 58.519	58.519	8	17.778	3.704				58.519										10.37	9.63			
909 V 41.481	41.481	6	2.222	0.741						28.889		21.481	3.704						0.741	41.481	0.741	
910 L 34.074	34.074	3	2.963	6.667			14.074	1.481		22.222		34.074						6.667		11.852		
911 G 86.667	86.667	9	1.481					86.667										11.852				
912 A 91.852	91.852	8	91.852					0.741										4.444	1.481	1.481		
913 L 59.259	59.259	5	3.704				0.741			9.63		59.259	4.444					0.741	0.741	20.741		
914 L 44.444	44.444	1	12.593	0.741			2.963	2.222		16.296		44.444	3.704					0.741	5.185	10.37	0.741	
915 A 52.593	52.593	5	52.593				11.852	18.519		0.741		2.222	1.481		6.667			2.963	1.481	0.741	0.741	
916 V 26.667	26.667	6	22.963							4.444		19.259	1.481	1.481	0.741			2.222	20.741	26.667		
917 T 21.642	21.642	1	5.97				5.97	1.493	0.746	4.478	2.239	6.716	5.97	6.716			1.493	10.448	21.642	2.239	18.657	5.224
918 L 60.448	60.448	4	1.493				9.701	0.746		6.716		60.448	10.448					1.493	0.746	4.478	3.731	
919 R 75.949	75.949	5	1.266				1.266	3.797			1.266	5.063	1.266		1.266		75.949	3.797	3.797	1.266		
920 G 73.333	73.333	4	2.963		2.963	0.741	3.704	73.333			0.741	2.963	2.222	3.704				0.741		2.963	2.222	0.741
921 L 45.185	45.185	6	8.148				3.704	11.852		0.741	0.741	45.185	12.593			2.963		5.185	5.926	1.481		1.481
922 T 22.963	22.963	5	4.444		15.556	14.815					0.741		1.481	8.148	13.333			17.778	22.963	0.741		
923 N 75.556	75.556	8								2.222		11.852	6.667	75.556			0.741	1.481		1.481		
924 D 60.000	60	9			60									39.259				0.741				
925 V 53.333	53.333	8								42.963		2.963	0.741							53.333		
926 Y 74.815	74.815	8					24.444						0.741									74.815
927 F 58.519	58.519	8	4.444				58.519					1.481						8.889	22.222	4.444		
928 Q 80.000	80	9			0.741	0.741					16.296	0.741	0.741			80				0.741		
929 V 71.852	71.852	8								28.148										71.852		
930 G 91.852	91.852	8	6.667					91.852										1.481				
931 L 88.148	88.148	8					2.963			2.222		88.148	5.926							0.741		
932 L 45.185	45.185	8					0.741			28.889		45.185	1.481						0.741	22.963		
933 T 78.519	78.519	9	0.741									4.444	1.481						78.519	14.815		
934 L 45.185	45.185	8								8.148		45.185						0.741	33.333	12.593		
935 I 70.370	70.37	8	0.741							70.37		1.481	6.667							20.741		
936 G 95.556	95.556	9	4.444					95.556														
937 L 97.778	97.778	9										97.778	0.741							1.481		
938 S 46.667	46.667	8	31.111							17.778								46.667	0.741	3.704		
939 A 61.481	61.481	7	61.481	7.407														19.259	11.852			
940 K 97.778	97.778	9									97.778						2.222					
941 N 81.481	81.481	9							16.296					81.481				0.741	1.481			
942 A 77.037	77.037	9	77.037					22.963														
943 I 100.000	100	9								100												
944 L 100.000	100	9										100										
945 I 89.630	89.63	9								89.63		1.481	7.407							1.481		
946 V 85.185	85.185	9	1.481	5.185			0.741			5.926									1.481	85.185		
947 E 97.037	97.037	9	0.741		0.741	97.037										1.481						
948 F 91.852	91.852	9					91.852													7.407		0.741
949 A 97.778	97.778	9	97.778							0.741								1.481				
950 K 48.148	48.148	7	1.481			5.185				0.741	48.148	1.481	1.481	21.481			15.556	0.741		3.704		
951 E 37.037	37.037	4	6.667		24.444	37.037		0.741	5.185	1.481	5.926	0.741	2.222	0.741		12.593	0.741	0.741	0.741			
952 L 57.778	57.778	4	5.926			6.667			0.741	2.222	6.667	57.778	5.926	2.963		5.185	5.185					0.741
953 Q 27.612	27.612	5				8.209	0.746		7.463	2.239	1.493	0.746	17.164	6.716		27.612	14.179		0.746	2.239	1.493	8.955
954 E 35.577	35.577	1	9.615		15.385	35.577		1.923	2.885		0.962	11.538		1.923		11.538	4.808	0.962		2.885		
955 K 31.111	31.111	2	4.444		2.222	27.778			5.556	2.222	31.111	6.667	5.556	4.444		4.444	1.111	2.222	2.222			
956 E 33.871	33.871	3	3.226		3.226	33.871		1.613	7.258		14.516	1.613		1.613		24.194	8.065		0.806			
957 G 93.333	93.333	8				0.741		93.333			0.741			3.704		0.741	0.741					
958 K 29.630	29.63	1	5.926			4.444	0.741	0.741	8.148	0.741	29.63	20.741	14.074	0.741		1.481	7.407			1.481	0.741	2.963
959 S 26.667	26.667	3	5.926		16.296	2.222		23.704	0.741					9.63	2.963		0.741	26.667	11.111			
960 L 48.889	48.889	3	4.444						1.481	8.148	9.63	48.889	1.481		5.185		9.63	1.481	3.704	5.926		
961 I 22.222	22.222	1	2.963	0.741		0.741	5.185	0.741	0.741	22.222	1.481	16.296	8.889		1.481	1.481	11.111	1.481	1.481	19.259	1.481	2.222

962 E 52.593	52.593	2	14.074		20	52.593			0.741		4.444			1.481		2.963	1.481	1.481	0.741			
963 A 94.815	94.815	9	94.815															5.185				
964 A 33.333	33.333	6	33.333							14.815		0.741	1.481					0.741	31.852	17.037		
965 L 50.370	50.37	1	1.481			5.926			0.741	9.63	2.222	50.37	6.667			2.222	2.963	1.481	3.704	12.593		
966 E 51.111	51.111	1	3.704		5.185	51.111			6.667	3.704	5.185		1.481	0.741		8.148	3.704	2.963	6.667			0.741
967 A 88.148	88.148	8	88.148					5.926										5.926				
968 A 51.852	51.852	7	51.852	16.296				0.741										11.111	0.741	19.259		
969 R 65.926	65.926	5	5.926					2.222	0.741		11.852		1.481			2.963	65.926	4.444	2.222	1.481		0.741
970 L 38.519	38.519	6			0.741	0.741				11.111		38.519	25.926			10.37	0.741	0.741	5.926	5.185		
971 R 100.000	100	9															100					
972 L 86.667	86.667	9					13.333					86.667										
973 R 100.000	100	9															100					
974 P 93.333	93.333	9	6.667												93.333							
975 I 86.667	86.667	9								86.667										13.333		
976 L 71.852	71.852	6								13.333		71.852	4.444	0.741						9.63		
977 M 100.000	100	9											100									
978 T 100.000	100	9																	100			
979 S 75.556	75.556	9	5.185															75.556	19.259			
980 L 59.259	59.259	7	14.815				6.667	0.741		10.37		59.259	3.704							3.704	0.741	
981 A 91.852	91.852	9	91.852															5.926	2.222			
982 F 78.519	78.519	9					78.519					0.741	16.296						4.444			
983 I 59.259	59.259	6	2.222					6.667		59.259		0.741	5.185					0.741	5.926	19.259		
984 L 45.185	45.185	5	19.259	2.222			17.778			2.222		45.185	9.63						0.741	2.963		
985 G 97.037	97.037	9	2.222					97.037										0.741				
986 V 74.074	74.074	8	2.963	1.481		0.741			3.704	0.741		7.407	3.704					0.741	4.444	74.074		
987 V 33.333	33.333	3	2.222				5.185			15.556		28.889	11.852					0.741	2.222	33.333		
988 P 100.000	100	9													100							
989 L 89.630	89.63	9										89.63	10.37									
990 V 37.037	37.037	6	31.852				0.741			7.407		13.333	5.185						2.963	37.037		1.481
991 I 36.296	36.296	1					17.778	0.741		36.296		27.407	2.222						6.667	4.444		4.444
992 S 47.407	47.407	7	46.667					2.222										47.407	0.741	2.963		
993 S 40.741	40.741	3	2.222		0.741	1.481			5.926	0.741	0.741		1.481	5.185		2.222	0.741	40.741	37.037	0.741		
994 G 98.519	98.519	9			0.741			98.519										0.741				
995 A 89.630	89.63	8	89.63					0.741							8.148				0.741	0.741		
996 G 89.630	89.63	9	0.741					89.63										9.63				
997 A 54.815	54.815	7	54.815		0.741	0.741			0.741		0.741					0.741		41.481				
998 G 34.815	34.815	4	15.556			22.222		34.815		2.222		1.481	0.741	8.889		1.481		4.444		8.148		
999 S 48.889	48.889	8	26.667					4.444					10.37			1.481		48.889	5.185	2.963		
1000 R 52.593	52.593	9									0.741	0.741				45.926	52.593					
1001 H 31.111	31.111	5	0.741			0.741	14.074	1.481	31.111	2.963				26.667		8.889	6.667	1.481	0.741	3.704		0.741
1002 A 60.741	60.741	7	60.741		5.185	1.481		2.963		1.481		0.741		0.741		1.481		21.481		3.704		
1003 I 48.889	48.889	8								48.889		11.852	11.111						2.963	25.185		
1004 G 99.259	99.259	9	0.741					99.259														
1005 T 53.333	53.333	7					1.481			17.778		8.889	2.222	0.741				2.222	53.333	11.852	1.481	
1006 G 50.370	50.37	7	12.593					50.37		2.222					0.741			5.926	7.407	20.741		
1007 V 74.815	74.815	9								19.259		5.926								74.815		
1008 F 21.481	21.481	5	5.926				21.481			18.519		11.852	20.741					2.222	1.481	17.778		
1009 G 64.444	64.444	6	11.111	0.741			1.481	64.444				0.741						15.556	3.704		1.481	0.741
1010 G 100.000	100	9						100														
1011 M 76.296	76.296	8								1.481		12.593	76.296						5.926	3.704		
1012 L 39.259	39.259	3	5.185				0.741	2.222		31.852		39.259	0.741					5.185	2.222	12.593		
1013 S 23.704	23.704	6	14.074	0.741			2.963	14.074		16.296		0.741	0.741					23.704	20.741	5.926		
1014 A 54.074	54.074	8	54.074					27.407										4.444		14.074		
1015 T 94.815	94.815	9	1.481									0.741	0.741					2.222	94.815			
1016 L 31.852	31.852	1	4.444	1.481			14.815	0.741		12.593		31.852	0.741					1.481	2.222	29.63		
1017 L 59.398	59.398	8	0.752				36.09			2.256		59.398								1.504		
1018 A 49.630	49.63	8	49.63					25.926										3.704	20	0.741		
1019 I 46.667	46.667	7								46.667		36.296	0.741						2.222	14.074		
1020 F 73.333	73.333	6					73.333			3.704		11.111								6.667		5.185
1021 F 57.778	57.778	8					57.778			1.481		15.556	0.741							20	2.963	1.481
1022 V 62.963	62.963	8								9.63		13.333							14.074	62.963		
1023 P 100.000	100	9													100							
1024 V 44.444	44.444	6	6.667	2.222				0.741		4.444		31.111	1.481					3.704	4.444	44.444		0.741
1025 F 74.815	74.815	7	2.963	1.481			74.815			8.889		3.704	2.963							4.444		0.741
1026 FY 49.630	49.63	7					49.63		0.741													49.63
1027 V 64.444	64.444	6	0.741				0.741		0.741	2.222	1.481	7.407	3.704	0.741		0.741	0.741	5.185	8.148	64.444		2.963
1028 L 37.037	37.037	2	7.407				5.926		0.741	2.222		37.037	0.741			0.741			2.222	28.889	6.667	7.407
1029 V 52.593	52.593	5	0.741				1.481			22.963		20	1.481							52.593		0.741
1030 R 43.704	43.704	5	17.037	0.741		2.963	0.741	4.444		2.222	3.704	3.704	4.444			7.407	43.704	4.444	2.222	1.481		0.741

1031 R 48.889	48.889	1	2.222		2.222	2.963		7.407	1.481		15.556			1.481		1.481	48.889	9.63	5.926			0.741
1032 R 27.407	27.407	1	2.963	0.741	5.926	2.963	8.148			7.407	5.185	19.259	3.704	2.963	1.481	0.741	27.407		2.222	5.185	2.222	1.481
1033 F 43.704	43.704	5	3.704		1.481	0.741	43.704	1.481	14.074	0.741	4.444	4.444		0.741		3.704	7.407	4.444		8.889		
1034 KS 14.925	14.925	1	4.478		2.985	14.179		11.194	2.239	1.493	14.925	2.239		1.493	11.94	4.478	6.716	14.925	6.716			
1035 R 32.090	32.09	1	1.493		4.478	2.239	0.746	6.716	2.239		17.91	6.716		0.746	8.209	1.493	32.09	4.478	0.746	3.731	4.478	1.493
1036 K 34.091	34.091	1	1.515		2.273	3.788	3.03	2.273	4.545	3.03	34.091	11.364	0.758	2.273	1.515	2.273	16.667	5.303	1.515	3.03		0.758
1037 P 13.077	13.077	1	6.923		3.846	9.231	0.769	4.615	5.385		11.538	3.846	1.538	6.923	13.077	7.692	5.385	10.769	4.615	3.077		0.769
1038 E 19.380	19.38	1	14.729		6.202	19.38	0.775	3.876	3.876	0.775	10.853	1.55		1.55	5.426	3.101	13.178	3.876	5.426	4.651		0.775
1039 D 12.000	12	1	8		12	8	7.2	2.4	4.8	1.6	8.8	2.4		1.6	9.6	4.8	10.4	5.6	5.6	4	3.2	
1040 A 15.238	15.238	1	15.238		5.714	11.429		5.714	0.952	6.667	7.619	3.81	1.905	2.857	3.81	5.714	7.619	6.667	5.714	7.619		0.952
1041 E 20.000	20	1	10		4	20	2	2	3	7	6	6		6	5	4	9	3	5	8		
1042 AEHPS 10.784	10.784	1	10.784		5.882	10.784	0.98	3.922	10.784	0.98	5.882	0.98	1.961	3.922	10.784	5.882	4.902	10.784	4.902	3.922		1.961
1043 E 18.367	18.367	1	4.082	1.02	4.082	18.367		3.061	7.143	1.02	4.082	10.204	4.082		12.245	3.061	3.061	17.347	5.102	2.041		
1044 H 17.778	17.778	1	11.111		5.556	12.222		2.222	17.778	1.111	10	6.667	1.111	3.333		6.667	2.222	6.667	5.556	7.778		
1045 P 16.279	16.279	1	13.953		5.814	10.465		3.488	2.326	3.488	8.14	4.651	1.163	1.163	16.279	3.488	3.488	10.465	9.302	2.326		
1046 SV 14.286	14.286	1	10.39		2.597	11.688		3.896	2.597	1.299	6.494	6.494		3.896	6.494	9.091	2.597	14.286	3.896	14.286		
1047 E 25.000	25	1	6.818		22.727	25		9.091	9.091	2.273	2.273	2.273			2.273	2.273		4.545	6.818			4.545
1048 H 26.316	26.316	1	5.263		5.263	13.158			26.316	2.632	13.158			5.263	5.263	5.263		13.158	5.263			
1049 H 43.243	43.243	1	10.811			5.405	2.703	5.405	43.243		2.703	8.108			2.703	8.108	2.703		2.703	5.405		
